# Ru-Catalyzed Asymmetric Addition of Arylboronic Acids to Aliphatic Aldehydes via *P*-Chiral Monophosphorous Ligands

**DOI:** 10.3390/molecules27123898

**Published:** 2022-06-17

**Authors:** Rui Miao, Yanping Xia, Yifei Wei, Lu Ouyang, Renshi Luo

**Affiliations:** 1 School of Pharmacy, Gannan Medical University, Ganzhou 341000, China; miaorui202205@163.com (R.M.); xyp110220@163.com (Y.X.); gnyxyghwyf@163.com (Y.W.); 2 College of Chemistry and Environmental Engineering, Shaoguan University, Shaoguan 512005, China

**Keywords:** Ru-catalyzed, asymmetric addition, *P*-chiral monophosphorous ligands, chiral alcohols

## Abstract

Chiral alcohols are among the most widely applied in fine chemicals, pharmaceuticals and agrochemicals. Herein, the Ru-monophosphine catalyst formed in situ was found to promote an enantioselective addition of aliphatic aldehydes with arylboronic acids, delivering the chiral alcohols in excellent yields and enantioselectivities and exhibiting a broad scope of aliphatic aldehydes and arylboronic acids. The enantioselectivities are highly dependent on the monophosphorous ligands. The utility of this asymmetric synthetic method was showcased by a large-scale transformation.

## 1. Introduction

Chiral alcohols are widely present in biologically active substances and pharmaceuticals [1,2,3,4,5,6,7]. Of these, chiral aryl alkyl alcohols are one important class of alcohols. The ubiquity of aryl alkyl alcohols as high-ranking intermediates makes them appealing precursors [8,9,10]. Thus, the invention of methods for the preparing of such chiral aryl alkyl alcohols features an important role in organic synthesis. Classic methods for constructing such chiral molecules include asymmetric hydrogenation of ketones [11,12,13], asymmetric hydrogen transfer reduction of ketones or unsaturated ketones [14,15,16,17,18,19,20,21], along with the asymmetric addition of organometallic reagents to aldehydes [22,23,24]. As an alternative, strategies via the asymmetric homologation reaction [25,26,27,28], tandem Michael–MPV reaction and subsequent reductive desulfurization [29], as well as the asymmetric addition of aldehydes with arylboronic acids were also established [30,31]. It is worth noting that the tandem *α*-alkylation/asymmetric reactions of ketones with alcohols [32] and the asymmetric Guerbet reaction of alcohols [33,34] are green and high-efficient strategies.

Similar to the organometallic reagents, the addition of organoborons to carbonyl compounds allows for the generation of chiral alcohols in direct and effective ways. Compared with the disadvantages of organometallic reagents [22,23,24], the organoborons feature the advantages of easy manipulation, low toxicity and good functional group tolerance [35], which has attracted great attention [36,37,38] since Miyaura reported the first Rh-catalyzed asymmetric addition of arylboronic acid to aldehydes in 1998 [39]. For example, metals including Rh [40,41,42,43,44,45,46,47,48], Ru [49], Ni [50], Co [51] and Pd [52,53,54] have been intensively explored in this regard recently. Additionally, miscellaneous carbonyl compounds, such as asisatins [55,56,57,58], ketoesters [59,60,61,62,63], diketones [64,65,66], trifluoroacetophenones [67,68,69], inactive ketones [70,71,72] and aldehydes [73,74,75] were successfully employed to couple with organoborons reagents.

Despite the fact that these remarkable achievements were created in the asymmetric addition of carbonyl compounds with organoboron reagents, efforts have been mainly devoted to the active ketones [55,56,57,58,59,60,61,62,63,64,65,66,67,68,69], inactive ketones [70,71,72], and aromatic aldehydes [73,74,75], but studies on aliphatic aldehydes have been less reported [30]. In this regard, the development of new, high-efficient catalytic systems for the enantioselective addition of aliphatic aldehydes with arylboronic acids still is desirable.

**Scheme 1:**
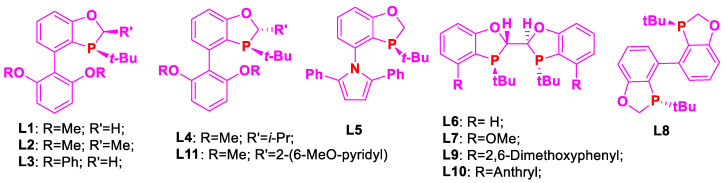
Chiral phosphorous ligands developed by Tang’s group.

**Scheme 2:**
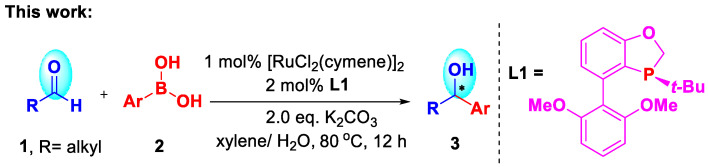
Asymmetric addition of aliphatic aldehydes with arylboronic acids.

**L1**~**L8** (Scheme 1) represent one kind of phosphorus ligand featured with a *P*-chiral center, which is successfully utilized in asymmetric synthesis [76,77,78,79,80,81,82]. For example, the ligands **L3** and **L9** were discovered to realize the asymmetric addition of aromatic aldehydes and active ketones with arylboronic acids with excellent enantioselectivities [67,83]. Based on this, we envisioned to further extend the utility of these chiral phosphorous ligands in the asymmetric catalysis. Herein, we reported the Ru-catalyzed enantioselective addition of aliphatic aldehydes with arylboronic acids via *P*-chiral monophosphorous ligands to access chiral secondary alcohols (Scheme 2), delivering the desired aryl alkyl alcohols in excellent yields and enantioselectivities. The application for gram-scale synthesis of **3da** was also disclosed.

## 2. Results and Discussions

At the outset of the research, initial experimental results show that the monophosphine ligand **L1** effectively promoted the Ru (II)-catalyzed asymmetric addition of phenylpropanal **1a** with 4-methylphenylboronic acid **2a** to access chiral alcohol **3aa** in excellent yields and with good enantioselectivity; only a trace amount of **4aa** was obtained as a byproduct (Table 1, entry 1). Further ligand investigations indicated that the ligands had a remarkable influence on enantioselectivity (Table 1, entries 2~5 and entry 11). For instance, good enantioselectivities can be afforded by using **L3** or **L5** as chiral phosphorous ligands (Table 1, entries 3 and 5), while a racemic or nearly racemic product was formed by employing **L2**, **L4** and **L11** (Table 1, entries 2, 4 and 11), indicating that the substituents on the carbon between the phosphorus and oxygen atom appear to have a negative influence on enantioselectivity.

Similar trends were also observed by using chiral bisphosphine ligands **L6**~**L7** or **L9**~**L10**,which were connected via *α*-position of a *P*-chiral center (Table 1, entries 6~7 and 9~10). For example, only low enantioselectivity was shown when bisphosphine ligands **L6**~**L7** and **L9**~**L10** were utilized in the reaction, while a moderate enantioselective product could be afforded by using **L8** as a bisphosphine ligand, which was coupled at the *β*-position (Table 1, entry 8). In general, the ruthenium precursors have no influence on the yields and enantioselectivities of this reaction (Table 1, entries 12 and 13). However, only low yields and enantioselectivities are accomplished using rhodium and iridium complexes as precursors (Table 1, entries 14 and 15).

The reaction was further assessed under various reaction conditions (Table 2). Solvent screening showcased that xylene/H_2_O was the optimal reaction medium, providing the corresponding asymmetric addition product **3aa** in full conversion and 91:9 *er* (Table 2, entry 7). Interestingly, full conversion and 93:7 *er* were obtained even when the reaction was conducted within 4 h (Table 2, entry 8). Corresponding various bases also delivered the desired chiral alcohol product **3aa** in good enantioselectivities (Table 2, entries 9~16), while organic bases, such as 1, 4-diazabicyclo [2.2.2] octane (DABCO), afforded a lower yield than inorganic bases (Table 2, entry 17).

**Scheme 3:**
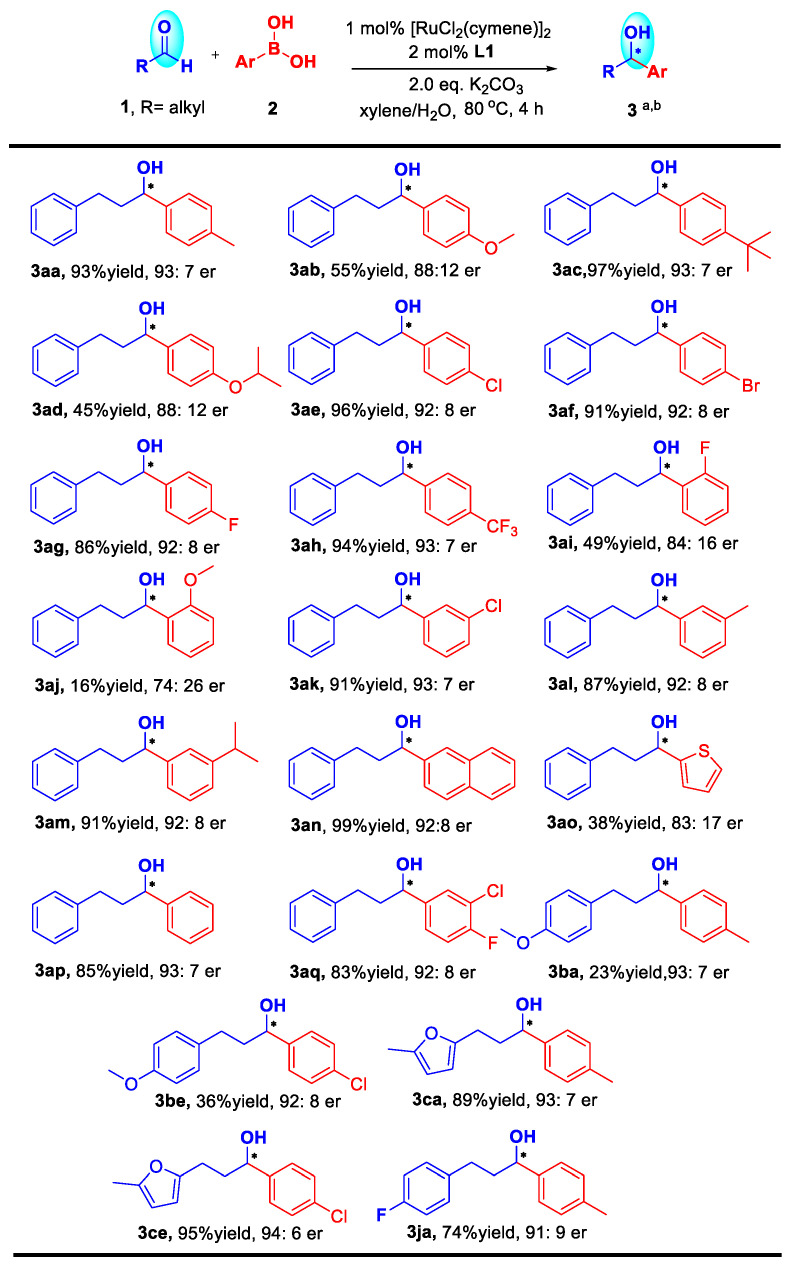
Substrates of 3-arylpropanal for asymmetric addition with arylboronic acids *^a^*^,*b*^. *^a^* Performed with **1** (0.5 mmol), **2** (2.0 eq.), [RuCl_2_(cymene)]_2_ (1.0 mol%), **L1** (2 mol%), K_2_CO_3_ (2.0 eq.), xylene (1.5 mL) and H_2_O (0.5 mL), at 80 °C under N_2_ atmosphere for 4 h. *^b^* The yield is isolated yield and *ee* is determined by CHIRAL column.

Having established the optimal conditions, we next investigated the scope of the reaction substrates (Scheme 3).

Generally, *para*substituted arylboronic acids bearing electron-donating or electron-withdrawing groups (**2a**~**2h**) reacted with phenyl propanal (**1a**) smoothly to form the relevant alcohol products (**3aa**, **3ac** and **3ae**~**3ah**) in excellent yields and enantioselectivities. However, only moderate yields and good enantioselectivities were observed when arylboronic acids bearing alkoxy substituents in the *para* position were introduced (**3ab** and **3ad**). On the other hand, the position of the substituent onaryl boronic acid has significant influence on enantioselectivity and yield. For instance, arylboronic acids bearing substituents in the *ortho* position resulted in low yields and enantioselectivities (**3ai** and **3aj**), presumably due to *ortho* effects [83]. Gratifyingly, the *meta*substituted phenylboronic acids are also well compatible under the standard conditions, giving the desired addition products excellent enantioselectivities and yields (**3ak**~**3am**). In addition, except for thiophene boronic acid (**3ao**), other different boronic acids, including 2-naphthalene boronic acid, phenyl boronic acid and disubstituted boronic acid, worked well in this catalytic system to yield the alcohols in excellent yields and enantioselectivities (**3an**, **3ap** and **3aq**). Unlike aryl boronic acid bearing alkoxy, phenyl propanal bearing 4-methoxy substituent on the phenyl group resulted in a low yield but with excellent enantioselectivities (**3ba** and **3be**). To our satisfaction, excellent yields and enantioselectivities resulted when furan-substituted propionaldehyde was loaded in this Ru-monophosphine catalytic system (**3ca** and **3ce**). Interestingly, aldehydes with electron-withdrawing substituents (**1j**) are also compatible with this catalytic system, giving desirable product **3ja** in the yield of 74% and 91:9 *er*.

**Scheme 4:**
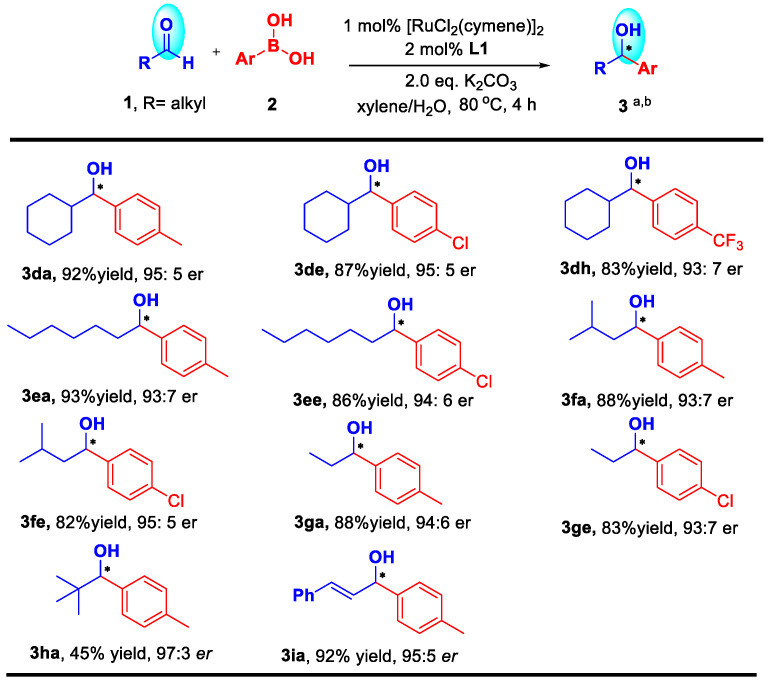
Substrates of alkyl aldehyde for asymmetric addition with arylboronic acids *^a^*^,*b*^. *^a^* Performed with **1** (0.5 mmol), **2** (2.0 eq.), [RuCl_2_(cymene)]_2_ (1.0 mol%), **L1** (2 mol%), K_2_CO_3_ (2.0 eq.), xylene (1.5 mL) and H_2_O (0.5 mL), at 80 °C under N_2_ atmosphere for 4 h. *^b^* The yield is isolated yield and *ee* is determined by CHIRAL column.

We next investigated the utility of this catalytic system for the substrate scope of aliphatic aldehydes bearing no aryl substituents (Scheme 4). Various common aliphatic aldehydes including cyclohexylformaldehyde, n-heptanal, isovaleraldehyde and propionaldehyde were also well tolerated in this catalytic system. Notably, the electron-withdrawing and electron-donating aryl boronic acids could be used as nucleophiles, generating chiral alcohols in 82%~93% yields and 93:7~95:5 *er*s (**3da**~**3ge**). Due to the steric hindrance, only a moderate yield and good enantioselectivity were afforded (**3ha**) when *t*-BuCHO was employed as a substrate under standard conditions. In addition, cinnamaldehyde can also react with **2a**, giving **3ia** in 90% *ee*.

**Scheme 5:**
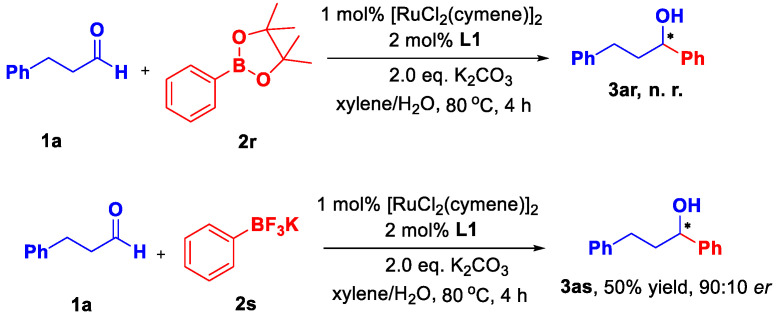
Substrates and other organoboron reagents tested in the system.

Additionally, in order to gain more insight into the versatility of nucleophilic reagents, other organoboron reagents were utilized under standard conditions. To our delight, potassium trifluoro(phenyl)borate (**2s**) could be compatible with this catalytic system, providing the desirable product in a moderate yield and with good *ee*. However, no desired product **3ar** was afforded when 4,4,5,5-tetramethyl-2-phenyl-1,3,2-dioxaborolane (**2r**) was severed as an organoboron reagent in this system (Scheme 5).

**Scheme 6:**
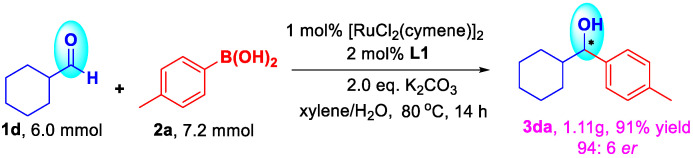
Large-scale experiment.

In order to illustrate the practicability of the catalytic system, we carried out a large-scale reaction of cyclohexyl formaldehyde (**1d**) and *p*-methylphenyl boronic acid (**2a**). Indeed, the corresponding addition product **3da** was successfully afforded on a 6.0 mmol scale from **1d** in a 91% yield and 94:6 *er* (Scheme 6).

**Scheme 7:**
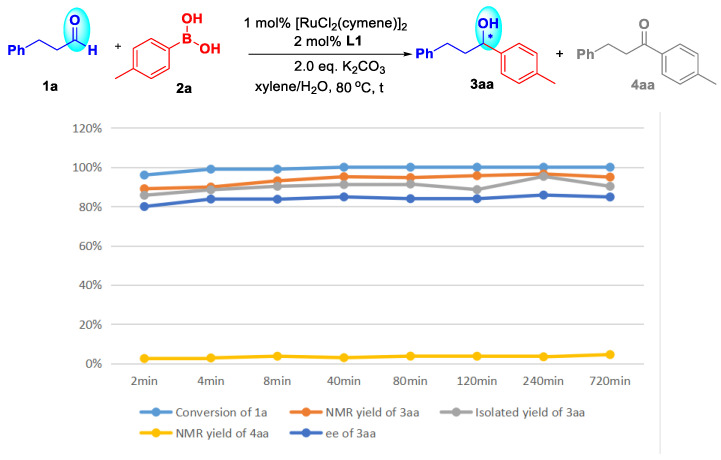
Plots for relationship of time, yield and enantioselectivity.

**Scheme 8:**
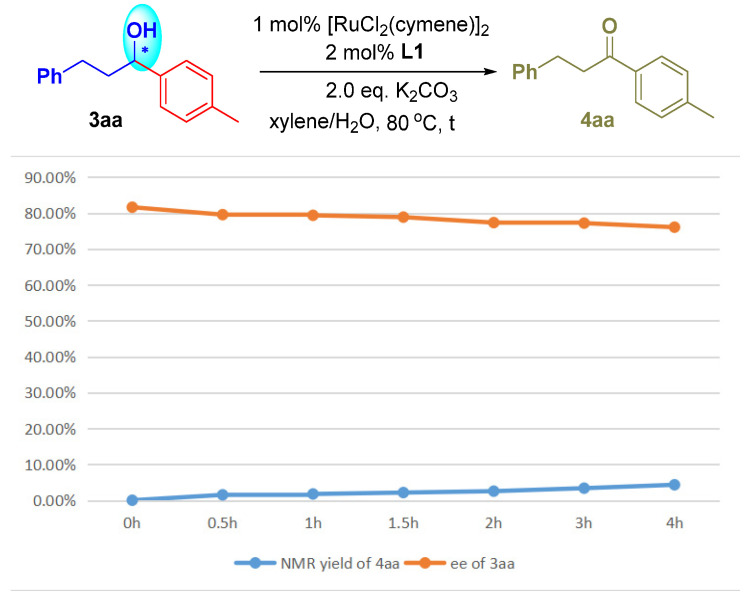
Plots for relationship of time, enantioselectivity and ketone formation.

The model reaction was measured over time to know about details of this reaction (Scheme 7). To our surprise, the treatment of **1a** with **2a** under standard conditions within 4 min afforded the desirable alcohol product **3aa** in the yield of 96% and 92:8 *er*, albeit with trace amounts of a ketone byproduct. The best enantioselectivity of product **3aa** was observed in 4 h, after which the enantioselectivities of alcohol decreased slightly, and the byproduct ketone increased slightly as time went by. In addition, the racemization of enantioenriched product **3aa** was monitored over time under the standard system, which showcased that the yield of **4aa** increased gradually, and a slight loss of enantioselectivity was observed (Scheme 8).

**Scheme 9:**
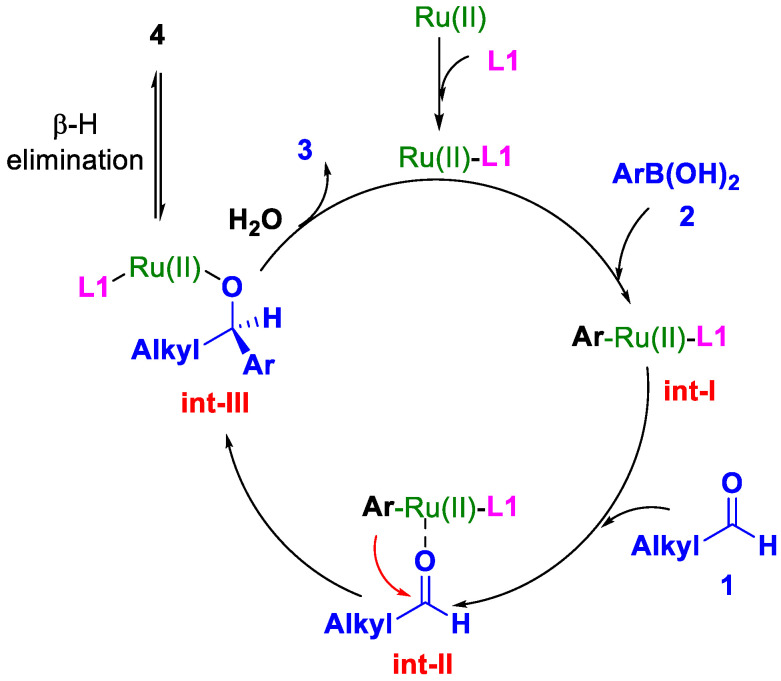
Proposed mechanism.

Based on the experimental results, we proposed a possible mechanistic cycle described in Scheme 9 [84]. Firstly, the catalyst **Ru-L1** was generated in situ with an Ru precursor and **L1**, followed by a transmetalation with aryl boronic acid to form **Int-I** under the condition of base. Subsequently, there was the coordination of aliphatic aldehydes with **Int-I** to generate the **Int-II**, followed by carbonyl insertion to produce **Int-III**. The *β*-H elimination of **Int-III** produced byproduct **4**. Finally, the addition of product **3** was released, and the catalyst was regenerated under the action of water and aryl boronic acid.

In summary, we described a Ru-catalyzed asymmetric addition of aliphatic aldehydes with aryl boronic acids based on a monophosphorous ligand, providing the corresponding alcohol products in excellent yields and enantioselectivities. A large-scale experiment showcased the utility of this catalytic system, which provides a supplementary method on acquiring chiral aryl alky alcohols.

## 3. Materials and Methods

### 3.1. General Information

Aliphatic aldehyde, arylboronic acids, dioxane, H_2_O, MTBE, THF, xylene and toluene were commercially acquired and used directly without further purifications. Reactions were carried out under a nitrogen atmosphere. ^1^H and ^13^C NMR spectra were recorded using a 400 MHz NMR spectrometer (CDCl_3_, *δ*_H_ = 7.26 ppm, *δ*_C_ = 77.23 ppm). The melting point was determined by a WRR melting point apparatus. The progress of the reaction was monitored by thin layer chromatography (TLC) or Agilent GC-7900. HPLC analyses were performed with an Agilent 1100 instrument using Chiralcel OD-H or OJ-H or Chiralpak AD-H, AS-H, IA or IB columns (0.46 cm diameter × 25 cm length). Optical rotations and MS spectra were recorded on a Perkin Elmer polarimeter (Model 341) and an ESI-ion trap mass spectrometer (Shimadzu LCMS-IT-TOF) separately.

### 3.2. General Procedure for Asymmetric Addition of Aliphatic Aldehydes

A mixture of aliphatic aldehydes (0.5 mmol), boric acids (1.0 mmol, 2.0 equiv), [RuCl_2_(cymene)]_2_ (3.1 mg, 1.0 mol%), **L1** (3.3 mg, 2.0 mol%) and K_2_CO_3_ (138.2 mg, 2.0 equiv) in *p*-xylene (1.5 mL)/H_2_O (0.5 mL) was added to a 25.0 mL Schlenk tube successively. Then, the reaction was stirred at 80 °C under N_2_ for 4h after which the reaction was diluted with H_2_O (15.0 mL), neutralized with HCl, and extracted with EtOAc (10.0 mL × 3). The organic layer was washed with brine (10.0 mL × 3) and dried over anhydrous MgSO_4_ after removal of the solvent under vacuum to afford the crude product, which was purified by column chromatography on silica gel with hexanes or petroleum ether/ethyl acetate (5:1 to 20:1) to deliver the desired product **3**. The enantioselectivities were determined by OD-H, AD-H, OJ-H, AS-H, IA or IB columns.

*3-phenyl-1-(p-tolyl)propan-1-ol* (**3aa**) [85]

${\left[a\right]}_{D}^{20}$ = +17.5 (*c* 0.29, CH_2_Cl_2_). Yield: 93% (105.1 mg) as yellow solid; m.p. 48–53 °C; ^1^H NMR (400 MHz, CDCl_3_) *δ* 7.66–6.66 (m, 9H), 4.60 (dd, *J* = 7.7, 5.5, 1H), 2.94–2.49 (m, 2H), 2.32 (s, 3H) and 2.21–1.86 (m, 3H).^13^C NMR (100 MHz, CDCl_3_) *δ* 141.94, 141.67, 137.33, 129.24, 128.51, 128.43, 125.98, 125.87, 73.75, 40.42, 32.15 and 21.18. The enantiomeric excess was determined by HPLC using Daicel Chiralpak OJ-H column, hexane/*i*-PrOH 95:5, flow rate 1.0 mL/min, UV detection at 220 nm, *t_minor_* = 13.758 min, *t_major_* = 16.774 min, and 93:7 *er*.

*1-(4-methoxyphenyl)-3-phenylpropan-1-ol* (**3ab**) [85]

${\left[a\right]}_{D}^{20}$ = +19.3 (*c* 0.66, CH_2_Cl_2_). Yield: 55% (66.6 mg) as a yellow oil; ^1^H NMR (400 MHz, CDCl_3_) *δ* 7.35–7.08 (m, 7H), 6.86 (d, *J* = 8.3, 2H), 4.59 (dd, *J* = 6.7, 1H), 3.77 (s, 3H), 2.78–2.51 (m, 2H) and 2.19–1.89 (m, 3H). ^13^C NMR (100 MHz, CDCl_3_) *δ* 159.11, 141.91, 136.77, 128.49, 128.42, 127.27, 125.87, 113.92, 73.49, 55.32, 40.39 and 32.16. The enantiomeric excess was determined by HPLC using Daicel Chiralpak AD-H column, hexane/*i*-PrOH 95:5, flow rate 1.0 mL/min, UV detection at 220 nm, *t_minor_* = 19.320 min, *t_major_* = 21.773 min and 88:12 *er*.

*1-(4-(tert-butyl)phenyl)-3-phenylpropan-1-ol* (**3ac**) [86]

${\left[a\right]}_{D}^{20}$ = +10.5 (*c* 0.32, CH_2_Cl_2_). Yield: 97% (130.2 mg) as a yellow oil; ^1^H NMR (400 MHz, CDCl_3_) *δ* 7.54–6.94 (m, 9H), 4.63 (dd, *J* = 7.9, 5.4, 1H), 2.99–2.54 (m, 2H), 2.25–1.98 (m, 2H), 1.95 (s, 1H) and 1.31 (s, 9H). ^13^C NMR (100 MHz, CDCl_3_) *δ* 150.64, 141.94, 141.62, 128.50, 128.41, 125.85, 125.74, 125.46, 73.71, 40.32, 34.58, 32.18 and 31.42. The enantiomeric excess was determined by HPLC using Daicel Chiralpak OJ-H column, hexane/*i*-PrOH 95:5, flow rate 1.0 mL/min, UV detection at 220 nm, *t_minor_* = 10.092 min, *t_major_* = 14.461 min and 93:7 *er*.

*1-(4-isopropoxyphenyl)-3-phenylpropan-1-ol* (**3ad**)

${\left[a\right]}_{D}^{20}$ = +12.1 (*c* 0.22, CH_2_Cl_2_). Yield: 45% (60.7 mg) as a yellow oil; ^1^H NMR (400 MHz, CDCl_3_) *δ* 7.53–7.03 (m, 7H), 6.86 (d, *J* = 8.3, 2H), 4.61 (dd, *J* = 6.7, 1H), 4.53 (p, *J* = 6.0, 1H), 2.78-2.57 (m, 2H), 2.19-1.93 (m, 2H), 1.87 (s, 1H) and 1.33 (d, *J* = 6.0, 6H). ^13^C NMR (100 MHz, CDCl_3_) *δ* 157.45, 141.90, 136.51, 128.47, 128.39, 127.25, 125.83, 115.86, 73.55, 69.95, 40.32, 32.17 and 22.09. HRMS-ESI (*m*/*z*): calculated for C_18_H_23_O_2_, [M+H]: 271.1698, found 271.1701. The enantiomeric excess was determined by HPLC using Daicel Chiralpak AD-H column, hexane/*i*-PrOH 95:5, flow rate 1.0 mL/min, UV detection at 220 nm, *t_minor_* = 14.219 min, *t_major_* = 16.039 min and 88:12 *er*.

*1-(4-chlorophenyl)-3-phenylpropan-1-ol* (**3ae**) [87]

${\left[a\right]}_{D}^{20}$ = +13.1 (*c* 1.17, CH_2_Cl_2_). Yield: 96% (118.3 mg) as a yellow oil; ^1^H NMR (400 MHz, CDCl_3_) *δ* 7.53–6.94 (m, 9H), 4.60 (dd, *J* = 7.8, 5.3, 1H), 2.81–2.52 (m, 2H), 2.15 (s, 1H) and 2.12–1.88 (m, 2H).^13^C NMR (100 MHz, CDCl_3_) *δ* 143.06, 141.54, 133.26, 128.67, 128.50, 128.46, 127.37, 126.02, 73.15, 40.51 and 31.95. The enantiomeric excess was determined by HPLC using Daicel Chiralpak IB column, hexane/*i*-PrOH 95:5, flow rate 0.5 mL/min, UV detection at 220 nm, *t_minor_* = 20.451 min, *t_major_* = 23.591 min and 92:8 *er*.

*1-(4-bromophenyl)-3-phenylpropan-1-ol* (**3af**) [88]

${\left[a\right]}_{D}^{20}$ = +10.0 (*c* 1.31, CH_2_Cl_2_). Yield:91% (132.4 mg) as a yellow oil; ^1^H NMR (400 MHz, CDCl_3_) *δ* 7.44 (d, *J* = 7.9, 2H), 7.35–7.03 (m, 7H), 4.59 (dd, *J* = 6.6, 1H), 2.75–2.56 (m, 2H), 2.12 (s, 1H) and 2.08–1.89 (m, 2H). ^13^C NMR (100 MHz, CDCl_3_) *δ* 143.58, 141.51, 131.62, 128.50, 128.46, 127.71, 126.02, 121.37, 73.18, 40.47 and 31.93. The enantiomeric excess was determined by HPLC using Daicel Chiralpak IB column, hexane/*i*-PrOH 95:5, flow rate 0.5 mL/min, UV detection at 220 nm, *t_minor_* = 22.304 min, *t_major_* = 25.606 min and 92:8 *er*.

*1-(4-fluorophenyl)-3-phenylpropan-1-ol* (**3ag**) [33]

${\left[a\right]}_{D}^{20}$ = +22.1 (*c* 0.99, CH_2_Cl_2_). Yield: 86% (98.8 mg) as a yellowish oil; ^1^H NMR (400 MHz, CDCl_3_) *δ* 7.60–7.09 (m, 7H), 7.01 (t, *J* = 8.6, 2H), 4.63 (dd, *J* = 7.8, 5.4, 1H), 2.77–2.57 (m, 2H) and 2.16–1.90 (m, 3H). ^13^C NMR (100 MHz, CDCl_3_) *δ* 162.24 (d, *J* = 245.4), 141.61, 140.33 (d, *J =* 3.1), 128.47, 128.44, 127.60 (d, *J* = 8.0), 125.97, 115.33 (d, *J =* 21.3), 73.22, 40.57 and 32.02. The enantiomeric excess was determined by HPLC using Daicel Chiralpak IB column, hexane/*i*-PrOH 95:5, flow rate 0.5 mL/min, UV detection at 220 nm, *t_minor_* = 20.004 min, *t_major_* = 22.949 min and 92:8 *er*.

*3-phenyl-1-(4-(trifluoromethyl)phenyl)propan-1-ol* (**3ah**) [33]

${\left[a\right]}_{D}^{20}$ = +17.1 (*c* 1.32, CH_2_Cl_2_). Yield: 94% (131.9 mg) as a yellow oil; ^1^H NMR (400 MHz, CDCl_3_) *δ* 7.56 (d, *J =* 8.0, 2H), 7.39 (d, *J =* 8.0, 2H), 7.34–6.99 (m, 5H), 4.68 (dd, *J =* 7.9, 5.2, 1H), 2.77–2.57 (m, 2H), 2.36 (s, 1H) and 2.13–1.91 (m, 2H). ^13^C NMR (100 MHz, CDCl_3_) *δ* 148.58, 141.38, 129.78 (q, *J =* 32.3), 128.54, 128.45, 126.21, 126.09, 125.46 (q, *J =* 3.8), 124.32 (q, *J =* 272.0), 73.17, 40.56 and 31.86. The enantiomeric excess was determined by HPLC using Daicel Chiralpak IB column, hexane/*i*-PrOH 95:5, flow rate 0.5 mL/min, UV detection at 210 nm, *t_minor_* = 21.798 min, *t_major_* = 24.446 min and 93:7 *er*.

*1-(2-fluorophenyl)-3-phenylpropan-1-ol* (**3ai**) [33]

${\left[a\right]}_{D}^{20}$ = +20.8 (*c* 0.53, CH_2_Cl_2_). Yield:49% (52.7 mg) as a yellow oil; ^1^H NMR (400 MHz, CDCl_3_) *δ* 7.45 (t, *J =* 7.6, 1H), 7.39-6.89 (m, 8H), 5.01 (t, *J =* 6.5, 1H), 2.90–2.58 (m, 2H) and 2.19–1.98 (m, 3H). ^13^C NMR (100 MHz, CDCl_3_) *δ* 159.87 (d, *J =* 245.5), 141.64, 131.50 (d, *J =* 13.2), 128.93 (d, *J =* 8.3), 128.44, 128.43, 127.34 (d, *J =* 4.7), 125.92, 124.35 (d, *J =* 3.6), 115.37 (d, *J =* 21.9), 68.01 (d, *J =* 2.5), 39.42 and 32.02. The enantiomeric excess was determined by HPLC using Daicel Chiralpak IB column, hexane/*i*-PrOH 95:5, flow rate 0.5 mL/min, UV detection at 220 nm, *t_minor_* = 17.583 min, *t_major_* = 19.992 min and 84:16 *er*.

*1-(2-methoxyphenyl)-3-phenylpropan-1-ol* (**3aj**) [89]

${\left[a\right]}_{D}^{20}$ = +18.5 (*c* 0.19, CH_2_Cl_2_). Yield: 16% (19.4 mg) as a yellow oil; ^1^H NMR (400 MHz, CDCl_3_) *δ* 7.38–7.11 (m, 7H), 6.96 (t, *J =* 7.4, 1H), 6.88 (d, *J =* 8.2, 1H), 4.88 (dd, *J =* 8.0, 5.1, 1H), 3.84 (s, 3H), 2.89–2.57 (m, 3H) and 2.22–2.02 (m, 2H). ^13^C NMR (100 MHz, CDCl_3_) *δ* 156.65, 142.20, 132.27, 128.48, 128.39, 128.31, 127.02, 125.72, 120.80, 110.58, 70.71, 55.27, 38.68 and 32.38. The enantiomeric excess was determined by HPLC using Daicel Chiralpak IB column, hexane/*i*-PrOH 95:5, flow rate 0.5 mL/min, UV detection at 220 nm, *t_minor_* = 19.673 min, *t_major_* = 24.260 min and 74:26 *er*.

*1-(3-chlorophenyl)-3-phenylpropan-1-ol* (**3ak**) [88]

${\left[a\right]}_{D}^{20}$ = +15.0 (c 0.55, CH_2_Cl_2_). Yield: 91% (111.7 mg) as a yellow oil; ^1^H NMR (400 MHz, CDCl_3_) *δ* 7.46–6.98 (m, 9H), 4.59 (dd, *J =* 7.9, 5.2, 1H), 2.85–2.53 (m, 2H), 2.26 (s, 1H) and 2.10–1.90 (m, 2H). ^13^C NMR (100 MHz, CDCl_3_) *δ* 146.74, 141.51, 134.43, 129.83, 128.51, 128.48, 127.73, 126.17, 126.03, 124.11, 73.20, 40.48, 31.94. The enantiomeric excess was determined by HPLC using Daicel Chiralpak IB column, hexane/*i*-PrOH 95:5, flow rate 0.5 mL/min, UV detection at 220 nm, *t_minor_* = 21.487 min, *t_major_* = 27.495 min and 93:7 *er*.

*3-phenyl-1-(m-tolyl)propan-1-ol* (**3al**) [88]

${\left[a\right]}_{D}^{20}$ = +21.0 (*c* 0.98, CH_2_Cl_2_). Yield: 87% (98.3 mg) as a yellow oil; ^1^H NMR (400 MHz, CDCl_3_) *δ* 7.63–6.82 (m, 9H), 4.59 (dd, *J =* 7.8, 5.5, 1H), 2.84–2.53 (m, 2H), 2.33 (s, 3H) and 2.18–1.91 (m, 3H). ^13^C NMR (100 MHz, CDCl_3_) *δ* 144.64, 141.93, 138.19, 128.52, 128.47, 128.44, 128.42, 126.70, 125.89, 123.08, 73.94, 40.47, 32.17 and 21.53. The enantiomeric excess was determined by HPLC using Daicel Chiralpak IB column, hexane/*i*-PrOH 95:5, flow rate 0.5 mL/min, UV detection at 220 nm, *t_minor_* = 17.873 min, *t_major_* = 20.271 min and 92:8 *er*.

*1-(3-isopropylphenyl)-3-phenylpropan-1-ol* (**3am**)

${\left[a\right]}_{D}^{20}$ = +18.3 (*c* 1.14, CH_2_Cl_2_). Yield:91% (102.8 mg) as a yellow oil; ^1^H NMR (400 MHz, CDCl_3_) *δ* 7.35–7.02 (m, 9H), 4.62 (dd, *J =* 7.9, 5.3, 1H), 2.89 (hept, *J =* 6.9, 1H), 2.80–2.59 (m, 2H), 2.16–1.88 (m, 3H) and 1.24 (d, *J =* 7.0, 6H). ^13^C NMR (100 MHz, CDCl_3_) *δ* 149.23, 144.65, 141.96, 128.55, 128.45, 125.90, 125.77, 124.17, 123.47, 74.11, 40.51, 34.24, 32.23 and 24.12 (d, *J =* 3.0). HRMS-ESI (*m*/*z*): calculated for C_18_H_22_NaO, [M+Na]: 277.1568, found 277.1574. The enantiomeric excess was determined by HPLC using Daicel Chiralpak IB column, hexane/*i*-PrOH 95:5, flow rate 0.5 mL/min, UV detection at 220 nm, *t_minor_* = 13.494 min, *t_major_* = 16.836 min and 92:8 *er*.

*1-(naphthalen-2-yl)-3-phenylpropan-1-ol* (**3an**) [90]

${\left[a\right]}_{D}^{20}$ = +13.3 (*c* 1.31, CH_2_Cl_2_). Yield: 99% (130.2 mg) as a light-yellow solid; m.p. 81-83 °C; ^1^H NMR (400 MHz, CDCl_3_) *δ* 7.86–7.62 (m, 4H), 7.42 (dd, *J =* 12.0, 7.9, 3H), 7.23 (d, *J =* 7.1, 2H), 7.14 (d, *J =* 8.0, 3H), 4.74 (t, *J =* 6.6, 1H), 2.82–2.48 (m, 2H), 2.30 (s, 1H) and 2.22–1.98 (m, 2H). ^13^C NMR (100 MHz, CDCl_3_) *δ* 141.99, 141.86, 133.36, 133.10, 128.56, 128.50, 128.44, 128.04, 127.80, 126.26, 125.97, 125.95, 124.79, 124.17, 74.00, 40.38 and 32.12. The enantiomeric excess was determined by HPLC using Daicel Chiralpak OD-H column, hexane/*i*-PrOH 90:10, flow rate 1.0 mL/min, UV detection at 220 nm, *t_minor_* = 18.230 min, *t_major_* = 23.050 min and 92:8 *er*.

*3-phenyl-1-(thiophen-2-yl)propan-1-ol* (**3ao**) [91]

${\left[a\right]}_{D}^{20}$ = +17.2 (*c* 0.30, CH_2_Cl_2_). Yield: 38% (38.4 mg) as a colorless oil; ^1^H NMR (400 MHz, CDCl_3_) *δ* 7.38–7.12 (m, 6H), 6.97 (d, *J =* 5.3, 2H), 4.92 (t, *J =* 6.7, 1H), 2.83–2.64 (m, 2H), 2.40–2.11 (m, 2H) and 2.05 (s, 1H).^13^C NMR (100 MHz, CDCl_3_) *δ* 148.53, 141.47, 128.52, 128.47, 126.70, 125.99, 124.72, 123.96, 69.54, 40.72 and 32.03. The enantiomeric excess was determined by HPLC using Daicel Chiralpak OD-H column, hexane/*i*-PrOH 95:5, flow rate 1.0 mL/min, UV detection at 220 nm, *t_minor_* = 21.015 min, *t_major_* = 31.268 min and 83:17 *er*.

*1,3-diphenylpropan-1-ol* (**3ap**) [85]

${\left[a\right]}_{D}^{20}$ = +21.3 (*c* 0.29, CH_2_Cl_2_). Yield: 85% (90.2 mg) as a yellow solid; m.p. 41–42 °C; ^1^H NMR (400 MHz, CDCl_3_) *δ* 7.52–6.98 (m, 10H), 4.64 (dd, *J =* 7.8, 5.5, 1H), 2.87–2.46 (m, 2H) and 2.18–1.94 (m, 3H). ^13^C NMR (100 MHz, CDCl_3_) *δ* 144.62, 141.85, 128.56, 128.50, 128.45, 127.68, 126.00, 125.91, 73.90, 40.50 and 32.10. The enantiomeric excess was determined by HPLC using Daicel Chiralpak OJ-H column, hexane/*i*-PrOH 95:5, flow rate 1.0 mL/min, UV detection at 220 nm, *t_minor_* = 16.095 min, *t_major_* = 19.016 min and 93:7 *er*.

*1-(3-chloro-4-fluorophenyl)-3-phenylpropan-1-ol* (**3aq**)

${\left[a\right]}_{D}^{20}$ = +15.2 (*c* 1.10, CH_2_Cl_2_). Yield: 83% (110.2 mg) as a yellow oil; ^1^H NMR (400 MHz, CDCl_3_) *δ* 7.72–6.90 (m, 8H), 4.60 (dd, *J =* 7.9, 5.2, 1H), 2.77–2.58 (m, 2H), 2.12 (s, 1H) and 2.12-1.88 (m, 2H). ^13^C NMR (100 MHz, CDCl_3_) *δ* 157.40 (d, *J =* 248.3), 141.70 (d, *J =* 3.7), 141.31, 128.53, 128.43, 128.18, 126.08, 125.62 (d, *J =* 7.2), 121.00 (d, *J =* 17.8), 116.54 (d, *J =* 21.1), 72.66, 40.56 and 31.89. HRMS-ESI (*m*/*z*): calculated for C_15_H_15_ClFO, [M+H]: 265.0795, found 265.0791. The enantiomeric excess was determined by HPLC using Daicel Chiralpak IB column, hexane/*i*-PrOH 95:5, flow rate 0.5 mL/min, UV detection at 220 nm, *t_minor_* = 23.081 min, *t_major_* = 26.696 min and 92:8 *er*.

*3-(4-methoxyphenyl)-1-(p-tolyl)propan-1-ol* (**3ba**) [92]

${\left[a\right]}_{D}^{20}$ = +15.0 (*c* 0.28, CH_2_Cl_2_). Yield: 23% (29.5 mg) as a colorless oil; ^1^H NMR (400 MHz, CDCl_3_) *δ* 7.48–6.97 (m, 6H), 6.82 (d, 2H), 4.63 (t, *J =* 6.7, 1H), 3.78 (s, 3H), 2.74–2.54 (m, 2H), 2.34 (s, 3H), 2.18–1.95 (m, 2H) and 1.80 (s, 1H). ^13^C NMR (100 MHz, CDCl_3_) *δ* 157.79, 141.65, 137.32, 133.88, 129.33, 129.20, 125.92, 113.81, 73.72, 55.27, 40.60, 31.18 and 21.13. The enantiomeric excess was determined by HPLC using Daicel Chiralpak IA column, hexane/*i*-PrOH 95:5, flow rate 1.0 mL/min, UV detection at 220 nm, *t_minor_* = 14.914 min, *t_major_* = 16.938 min and 93:7 *er*.

*1-(4-chlorophenyl)-3-(4-methoxyphenyl)propan-1-ol* (**3be**) [92]

${\left[a\right]}_{D}^{20}$ = +6.9 (*c* 0.49, CH_2_Cl_2_). Yield: 36% (49.8 mg) as a colorless oil; ^1^H NMR (400 MHz, CDCl_3_) *δ* 7.29 (q, *J =* 8.3, 4H), 7.09 (d, *J =* 8.2, 2H), 6.82 (d, *J =* 8.3, 2H), 4.64 (dd, *J =* 7.9, 5.3, 1H), 3.78 (s, 3H), 2.75–2.54 (m, 2H) and 2.12–1.88 (m, 3H). ^13^C NMR (100 MHz, CDCl_3_) *δ* 157.87, 143.11, 133.51, 133.21, 129.32, 128.63, 127.32, 113.89, 73.13, 55.28, 40.74 and 31.01. The enantiomeric excess was determined by HPLC using Daicel Chiralpak IA column, hexane/*i*-PrOH 95:5, flow rate 0.5 mL/min, UV detection at 220 nm, *t_minor_* = 30.043 min, *t_major_* = 32.492 min and 92:8 *er*.

*3-(5-methylfuran-2-yl)-1-(p-tolyl)propan-1-ol* (**3ca**)

${\left[a\right]}_{D}^{20}$ = +14.4 (*c* 1.02, CH_2_Cl_2_). Yield: 89% (102.3 mg) as a yellow oil; ^1^H NMR (400 MHz, CDCl_3_) *δ* 7.29–7.09 (m, 4H), 5.83 (d, *J =* 5.1, 2H), 4.64 (t, *J =* 6.7, 1H), 2.63 (h, *J =* 8.6, 8.1, 2H), 2.33 (s, 3H), 2.23 (s, 3H) and 2.12–1.94 (m, 3H). ^13^C NMR (100 MHz, CDCl_3_) *δ* 153.78, 150.39, 141.48, 137.30, 129.19, 125.92, 105.86, 105.60, 73.62, 37.24, 24.56, 21.15 and 13.54. HRMS-ESI (*m*/*z*): calculated for C_15_H_19_O_2_, [M + H]: 231.1385, found 231.1385. The enantiomeric excess was determined by HPLC using Daicel Chiralpak IB column, hexane/*i*-PrOH 95:5, flow rate 0.5 mL/min, UV detection at 220 nm, *t_minor_* = 13.845 min, *t_major_* = 14.888 min and 93:7 *er*.

*1-(4-chlorophenyl)-3-(5-methylfuran-2-yl)propan-1-ol* (**3ce**)

${\left[a\right]}_{D}^{20}$ = +9.4 (*c* 1.19, CH_2_Cl_2_). Yield: 95% (118.9 mg) as a yellow oil; ^1^H NMR (400 MHz, CDCl_3_) *δ* 7.26 (q, *J =* 8.4, 4H), 5.84 (d, *J =* 3.5, 2H), 4.65 (dd, *J =* 7.7, 5.5, 1H), 2.62 (t, *J =* 7.7, 2H), 2.28 (s, 1H), 2.23 (s, 3H) and 2.10–1.90 (m, 2H). ^13^C NMR (100 MHz, CDCl_3_) *δ* 153.36, 150.53, 142.87, 133.22, 128.62, 127.31, 105.91, 105.81, 73.02, 37.36, 24.37 and 13.53. HRMS-ESI (*m*/*z*): calculated for C_14_H_16_ClO_2_, [M + H]: 251.0839, found 251.0836. The enantiomeric excess was determined by HPLC using Daicel Chiralpak OD-H column, hexane/*i*-PrOH 95:5, flow rate 1.0 mL/min, UV detection at 220 nm, *t_minor_* = 17.860 min, *t_major_* = 19.360 min and 94:6 *er*.

*3-(4-fluorophenyl)-1-(p-tolyl)propan-1-ol* (**3ja**)

${\left[a\right]}_{D}^{20}$ = +20.4 (*c* 0.86, CH_2_Cl_2_). Yield: 74% (90.5 mg) as a yellow oil; ^1^H NMR (400 MHz, CDCl_3_) *δ* 7.24–7.05 (m, 6H), 6.93 (t, *J =* 8.8, 2H), 4.63–4.55 (m, 1H), 2.74-2.53 (m, 2H), 2.33 (s, 3H), 2.13–2.00 (m, 2H) and 1.94 (ddt, *J =* 13.6, 9.8, 6.0, 1H).^13^C NMR (101 MHz, CDCl_3_) *δ* 161.28 (d, *J =* 243.3), 141.53, 137.48 (d, *J =* 3.1), 137.43, 129.78 (d, *J =* 7.7), 129.26, 125.92, 115.10 (d, *J =* 21.1), 73.61, 40.50, 31.29 and 21.16. The enantiomeric excess was determined by HPLC using Daicel Chiralpak OJ-H column, hexane/*i*-PrOH 95:5, flow rate 1.0 mL/min, UV detection at 220 nm, *t_minor_* = 14.161 min, *t_major_* = 17.151 min and 91:9 *er*. HRMS-ESI (*m*/*z*): calcd for C_1__6_H_1__8_OF, [M+H]: 245.1342, found 245.1335.

*cyclohexyl(p-tolyl)methanol* (**3da**) [93]

${\left[a\right]}_{D}^{20}$ = +27.8 (*c* 0.93, CH_2_Cl_2_). Yield: 92% (93.9 mg) as a light-yellow oil; ^1^H NMR (400 MHz, CDCl_3_) *δ* 7.15 (q, *J =* 7.9, 4H), 4.30 (d, *J =* 7.3, 1H), 2.33 (s, 3H), 1.99 (d, *J =* 12.9, 1H), 1.87 (s, 1H), 1.75 (d, *J =* 12.9, 1H), 1.70–1.52 (m, 3H), 1.35 (d, *J =* 12.8, 1H) and 1.24–0.85 (m, 5H). ^13^C NMR (100 MHz, CDCl_3_) *δ* 140.71, 137.02, 128.89, 126.60, 79.26, 44.93, 29.33, 28.98, 26.47, 26.13, 26.05 and 21.14. The enantiomeric excess was determined by HPLC using Daicel Chiralpak IB column, hexane/*i*-PrOH 95:5, flow rate 0.5 mL/min, UV detection at 220 nm, *t_minor_* = 9.882 min, *t_major_* = 10.925 min and 95:5 *er*.

*(4-chlorophenyl)(cyclohexyl)methanol* (**3de**) [93]

${\left[a\right]}_{D}^{20}$ = +21.9 (*c* 0.96, CH_2_Cl_2_). Yield: 87% (97.7 mg) as a yellow oil; ^1^H NMR (400 MHz, CDCl_3_) *δ* 7.44–7.09 (m, 4H), 4.33 (d, *J =* 7.0, 1H), 2.03 (s, 1H), 1.91 (d, *J =* 12.9, 1H), 1.82–1.47 (m, 4H), 1.36 (d, *J =* 12.6, 1H) and 1.29–0.85 (m, 5H). ^13^C NMR (100 MHz, CDCl_3_) *δ* 142.03, 132.99, 128.29, 127.99, 78.61, 45.00, 29.19, 28.65, 26.37, 26.05 and 25.97. The enantiomeric excess was determined by HPLC using Daicel Chiralpak IA column, hexane/*i*-PrOH 98:2, flow rate 0.5 mL/min, UV detection at 220 nm, *t_minor_* = 25.489 min, *t_major_* = 27.163 min and 95:5 *er*.

*cyclohexyl(4-(trifluoromethyl)phenyl)methanol* (**3dh**) [94]

${\left[a\right]}_{D}^{20}$ = +16.5 (*c* 1.06, CH_2_Cl_2_). Yield: 83% (107.1 mg) as a white soild; m.p. 55–57 °C; ^1^H NMR (400 MHz, CDCl_3_) *δ* 7.49 (dd, *J =* 73.7, 8.0, 4H), 4.44 (d, *J =* 6.7, 1H), 2.09 (s, 1H), 1.88 (d, *J =* 12.4, 1H), 1.81–1.53 (m, 4H), 1.39 (d, *J =* 12.6, 1H) and 1.27-0.93 (m, 5H). ^13^C NMR (100 MHz, CDCl_3_) *δ* 147.51, 129.54 (q, *J =* 32.3), 126.90, 125.08 (q, *J =* 3.9), 124.22 (q, *J =* 271.9), 78.63, 45.03, 29.22, 28.36, 26.31, 26.04 and 25.94.The enantiomeric excess was determined by HPLC using Daicel Chiralpak IA column, hexane/*i*-PrOH 98:2, flow rate 0.5 mL/min, UV detection at 220 nm, *t_minor_* = 21.107 min, *t_major_* = 23.564 min and 93:7 *er*.

*1-(p-tolyl)heptan-1-ol* (**3ea**) [92]

${\left[a\right]}_{D}^{20}$ = +21.7 (*c* 0.95, CH_2_Cl_2_). Yield: 93% (95.7 mg) as a yellow solid; m.p. 34–35 °C; ^1^H NMR (400 MHz, CDCl_3_) *δ* 7.44-6.94 (m, 4H), 4.57 (t, *J =* 6.7, 1H), 2.33 (s, 3H), 2.04 (s, 1H), 1.83–1.59 (m, 2H), 1.43–1.15 (m, 8H) and 0.86 (t, *J =* 6.6, 3H). ^13^C NMR (100 MHz, CDCl_3_) *δ* 142.07, 137.07, 129.10, 125.91, 74.53, 39.07, 31.82, 29.26, 25.88, 22.66, 21.13 and 14.11. The enantiomeric excess was determined by HPLC using Daicel Chiralpak AS-Hcolumn, hexane/*i*-PrOH 99:1, flow rate 1.0 mL/min, UV detection at 220 nm, *t_minor_* = 8.920 min, *t_major_* = 11.107 min and 93:7 *er*.

*1-(4-chlorophenyl)heptan-1-ol* (**3ee**) [95]

${\left[a\right]}_{D}^{20}$ = +21.1 (*c* 0.97, CH_2_Cl_2_). Yield: 86% (97.4 mg) as a yellow solid; m.p. 50–52 °C; ^1^H NMR (400 MHz, CDCl_3_) *δ* 7.47–7.12 (m, 4H), 4.61 (t, *J =* 6.7, 1H), 2.13 (s, 1H), 1.81–1.57 (m, 2H), 1.42–1.15 (m, 8H) and 0.87 (t, *J =* 6.7, 3H). ^13^C NMR (100 MHz, CDCl_3_) *δ* 143.39, 133.04, 128.53, 127.30, 73.97, 39.16, 31.76, 29.16, 25.67, 22.60 and 14.08. The enantiomeric excess was determined by HPLC using Daicel Chiralpak OJ-Hcolumn, hexane/*i*-PrOH 99:1, flow rate 0.5 mL/min, UV detection at 220 nm, *t_minor_* = 28.980 min, *t_major_* = 32.669 min and 94:6 *er*.

*3-methyl-1-(p-tolyl)butan-1-ol* (**3fa**) [96]

${\left[a\right]}_{D}^{20}$ = +35.0 (*c* 0.78, CH_2_Cl_2_). Yield: 88% (78.3 mg) as a yellow solid; m.p. 40–43 °C; ^1^H NMR (400 MHz, CDCl_3_) *δ* 7.40–7.04 (m, 4H), 4.67 (dd, *J =* 7.9, 5.5, 1H), 2.33 (s, 3H), 1.93 (s, 1H), 1.76–1.59 (m, 2H), 1.47 (td, *J =* 11.2, 10.0, 5.5, 1H) and 0.93 (dd, *J =* 6.5, 3.1, 6H).^13^C NMR (100 MHz, CDCl_3_) *δ* 142.30, 137.14, 129.16, 125.88, 72.60, 48.28, 24.84, 23.10, 22.36 and 21.13. The enantiomeric excess was determined by HPLC using Daicel Chiralpak AS-H column, hexane/*i*-PrOH 95:5, flow rate 1.0 mL/min, UV detection at 220 nm, *t_minor_* = 4.584 min, *t_major_* = 5.628 min and 93:7 *er*.

*1-(4-chlorophenyl)-3-methylbutan-1-ol* (**3fe**) [97]

${\left[a\right]}_{D}^{20}$ = +37.9 (*c* 0.81, CH_2_Cl_2_). Yield: 82% (81.4 mg) as a light-yellow oil; ^1^H NMR (400 MHz, CDCl_3_) *δ* 7.27 (q, *J =* 8.5, 4H), 4.69 (dd, *J =* 8.1, 5.3, 1H), 2.08 (s, 1H), 1.73–1.57 (m, 2H), 1.50–1.38 (m, 1H) and 0.93 (dd, *J =* 6.4, 2.0, 6H). ^13^C NMR (100 MHz, CDCl_3_) *δ* 143.70, 133.06, 128.58, 127.27, 72.09, 48.39, 24.75, 23.09 and 22.22. The enantiomeric excess was determined by HPLC using Daicel Chiralpak AS-H column, hexane/*i*-PrOH 99:1, flow rate 0.5 mL/min, UV detection at 210 nm, *t_minor_* = 24.966 min, *t_major_* = 28.431 min and 95:5 *er*.

*1-(p-tolyl)propan-1-ol* (**3ga**) [98]

${\left[a\right]}_{D}^{20}$ = +37.2 (*c* 0.44, CH_2_Cl_2_). Yield: 88% (66.0 mg) as a light-yellow oil; ^1^H NMR (400 MHz, CDCl_3_) *δ* 7.21 (d, *J =* 8.1, 2H), 7.14 (d, *J =* 7.4, 2H), 4.53 (t, *J =* 6.7, 1H), 2.34 (s, 3H), 1.94 (s, 1H), 1.88–1.64 (m, 2H) and 0.90 (t, *J =* 6.4, 3H). ^13^C NMR (100 MHz, CDCl_3_) *δ* 141.67, 137.14, 129.09, 125.96, 75.89, 31.81, 21.12 and 10.20. The enantiomeric excess was determined by HPLC using Daicel Chiralpak AS-H column, hexane/*i*-PrOH 98:2, flow rate 1.0 mL/min, UV detection at 220 nm, *t_minor_* = 10.091 min, *t_major_* = 12.376 min and 94:6 *er*.

*1-(4-chlorophenyl)propan-1-ol* (**3ge**) [98]

${\left[a\right]}_{D}^{20}$ = +32.9 (*c* 0.54, CH_2_Cl_2_). Yield: 83% (70.5 mg) as a light-yellow oil; ^1^H NMR (400 MHz, CDCl_3_) *δ* 7.39–7.14 (m, 4H), 4.53 (t, *J =* 6.7, 1H), 2.29 (s, 1H), 1.81–1.61 (m, 2H) and 0.87 (t, *J =* 8.3, 3H). ^13^C NMR (100 MHz, CDCl_3_) *δ* 143.03, 133.05, 128.49, 127.37, 75.24, 31.91 and 9.97. The enantiomeric excess was determined by HPLC using Daicel Chiralpak OD-H column, hexane/*i*-PrOH 98:2, flow rate 1.0 mL/min, UV detection at 210 nm, *t_minor_* = 13.033min, *t_major_* = 14.609 min and 93:7 *er*.

*2,2-dimethyl-1-(p-tolyl)propan-1-ol* (**3ha**) [30]

${\left[a\right]}_{D}^{20}$ = +30.0 (*c* 0.23, CH_2_Cl_2_). Yield: 45% (40.1 mg) as a light-yellow oil; ^1^H NMR (400 MHz, CDCl_3_) *δ* = 7.20 (d, *J =* 8.1, 2H), 7.13 (d, *J =* 8.0, 2H), 4.37 (d, *J =* 2.2, 1H), 2.35 (s, 3H), 1.83 (s, 1H) and 0.92 (s, 9H). ^13^C NMR (101 MHz, CDCl_3_) δ = 139.25, 136.89, 128.27, 127.51, 82.30, 35.62, 25.94 and 21.11. The enantiomeric excess was determined by HPLC using Daicel Chiralpak AS-H column, hexane/*i*-PrOH 95:5, flow rate 1.0 mL/min, UV detection at 220 nm, *t_minor_* = 4.778min, *t_major_* = 6.400 min and 97:3 *er*.

*(E)-3-phenyl-1-(p-tolyl)prop-2-en-1-ol* (**3ia**) [99]

${\left[a\right]}_{D}^{20}$ = +33.9 (*c* 1.03, CH_2_Cl_2_). Yield: 92% (102.7 mg) as a yellow solid; m.p. 65–68 °C; ^1^H NMR (400 MHz, CDCl_3_) *δ* 7.54–6.99 (m, 9H), 6.62 (d, *J* = 15.8, 1H), 6.33 (dd, *J* = 15.8, 6.4, 1H), 5.28 (d, *J* = 6.4, 1H), 2.35 (s, 1H) and 2.32 (s, 3H). ^13^C NMR (100 MHz, CDCl_3_) *δ* 139.98, 137.55, 136.71, 131.79, 130.32, 129.37, 128.62, 127.77, 126.69, 126.43, 74.97 and 21.23. The enantiomeric excess was determined by HPLC using Daicel Chiralpak OD-H column, hexane/*i*-PrOH 90:10, flow rate 1.0 mL/min, UV detection at 254 nm, *t_minor_* = 16.002 min, *t_major_* = 22.736 min and 95:5 *er*.

*4′-Methyl-3-phenylpropiophenone* (**4aa**) [100]

Yellow oil; ^1^H NMR (400 MHz, CDCl_3_) *δ* 7.85 (d, *J* = 8.3, 2H), 7.35–7.15 (m, 7H), 3.26 (t, 2H), 3.05 (t, *J* = 7.8, 2H) and 2.39 (s, 3H). ^13^C NMR (100 MHz, CDCl_3_) *δ* 198.98, 143.92, 141.45, 134.36, 129.34, 128.57, 128.49, 128.22, 126.16, 40.42, 30.23 and 21.72.

*Ru-L1*

Yield: 85% (35 mg) as a red solid. ^1^H NMR (400 MHz, CDCl_3_) *δ* 7.36 (t, *J* = 7.9 Hz, 1H), 7.19 (t, *J* = 8.3 Hz, 1H), 6.93 (dd, *J* = 7.6, 1.1 Hz, 1H), 6.88-6.77 (m, 3H), 5.38 (d, *J* = 10.9 Hz, 1H), 4.49 (d, *J* = 10.9 Hz, 1H), 3.96 (s, 3H), 3.77 (s, 3H) and 0.87 (d, *J* = 14.0 Hz, 9H). ^13^C NMR (100 MHz, CDCl_3_) *δ* 161.80, 157.76, 157.71, 136.47, 131.00, 127.61, 125.59, 124.43, 118.32, 110.68, 108.49, 70.53, 63.47, 55.94, 36.42 and 27.25. ^31^P NMR (162 MHz, CDCl_3_) *δ* 72.32. HRMS-ESI (*m*/*z*): calculated for C_38_H_47_O_6_Cl_2_RuP_2_, [M+H]: 833.1268, found 833.1254.

## Figures and Tables

**Table 1:** Ligand and precursor screening of metal-catalyzed asymmetric additions of aliphatic aldehydes *^a^*.

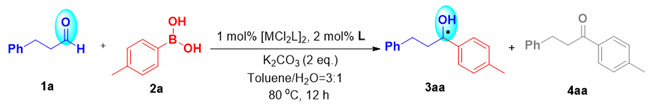						
Entry	Metal	Ligands	Conv. (%) *^b^*	NMR Yield of 3aa/4aa (%) *^b^*	Isolated Yield of 3aa (%)	*er* of 3aa (%) *^c^*
1	[RuCl_2_(cymene)]_2_	**L1**	>99	97/4	92	89:11
2	[RuCl_2_(cymene)]_2_	**L2**	>99	91/4	80	50:50
3	[RuCl_2_(cymene)]_2_	**L3**	>99	93/3	83	83:17
4	[RuCl_2_(cymene)]_2_	**L4**	16	12/4	10	59:41
5	[RuCl_2_(cymene)]_2_	**L5**	78	64/4	54	83:17
6	[RuCl_2_(cymene)]_2_	**L6**	--	Trace	--	26:74
7	[RuCl_2_(cymene)]_2_	**L7**	53	29/3	12	30:70
8	[RuCl_2_(cymene)]_2_	**L8**	66	41/4	16	77:23
9	[RuCl_2_(cymene)]_2_	**L9**	69	39/2	18	61:39
10	[RuCl_2_(cymene)]_2_	**L10**	65	30/2	14	48:52
11	[RuCl_2_(cymene)]_2_	**L11**	70	36/3	14	57:43
12	[RuCl_2_(benzene)]_2_	**L1**	>99	94/5	86	89:11
13	[RuCl_2_Cp*]_2_	**L1**	>99	100/0	96	87:13
14	[Rh(CH_2_CH_2_)_2_Cl]_2_	**L1**	>99	93/8	80	49:51
15	[Ir(coe)_2_Cl]_2_	**L1**	--	Trace	--	61:39

*^a^*Performed with **1a** (0.5 mmol), **2a** (2.0 eq.), metal (1 mol%), ligand (2 mol%), K_2_CO_3_ (2.0 eq.), toluene (1.5 mL), H_2_O (0.5 mL), at 80 °C under N_2_ atmosphere for 12 h. *^b^* Determined by NMR with dimethyl terephthalate as internal standard. *^c^* Determined by Chiral OJ-H column.

**Table 2:** Optimization of reaction conditions *^a^*.

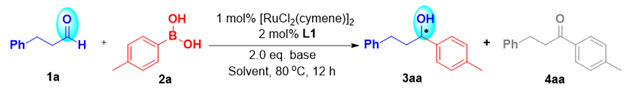						
Entry	Solvent	Solvent	Conv. (%) *^b^*	NMR Yield of 3aa/4aa (%) *^b^*	Isolated Yield of 3aa (%)	*er* of 3aa (%) *^c^*
1	H_2_O	K_2_CO_3_	>99	91/4	90	86:14
2	toluene	K_2_CO_3_	>99	94/3	89	87:13
3	toluene/H_2_O (3:1)	K_2_CO_3_	>99	96/4	92	89:11
4	dioxane	K_2_CO_3_	>99	95/3	86	90:10
5	dioxane/H_2_O (3:1)	K_2_CO_3_	>99	92/2	76	89:11
6	MTBE/H_2_O (5:1)	K_2_CO_3_	>99	80/8	67	90:10
7	xylene/H_2_O (3:1)	K_2_CO_3_	>99	95/5	91	91:9
8 *^d^*	xylene/H_2_O (3:1)	K_2_CO_3_	>99	96/3	95	93:7
9	THF	K_2_CO_3_	85	64/4	53	76:24
10	xylene/H_2_O (3:1)	K_3_PO_4_	>99	93/4	90	89:11
11	xylene/H_2_O (3:1)	Na_2_CO_3_	>99	96/4	88	90:10
12	xylene/H_2_O (3:1)	Li_2_CO_3_	>99	95/5	82	90:10
13	xylene/H_2_O (3:1)	Cs_2_CO_3_	>99	96/5	80	89:11
14	xylene/H_2_O (3:1)	KOH	>99	87/3	91	89:11
15	xylene/H_2_O (3:1)	KO*t*Bu	>99	80/3	55	92:8
16	xylene/H_2_O (3:1)	NaOMe	89	77/2	67	91:9
17	xylene/H_2_O (3:1)	DABCO	88	78/5	51	90:10

*^a^* Performed with **1a** (0.5 mmol), **2a** (2.0 eq.), [RuCl_2_(cymene)]_2_ (1.0 mol%), **L1** (2 mol%), base (2.0 eq.), solvent (2.0 mL), at 80 °C under N_2_ atmosphere for 12 h. *^b^* Determined by NMR with dimethyl terephthalate as internal standard. *^c^* Determined by Chiral OJ-H column. *^d^* reaction time: 4 h.

## Data Availability

The data presented in this study and in the Supplementary Materials are available on request from the corresponding author.

## Supplementary Materials

The following are available online: https://www.mdpi.com/article/10.3390/molecules27123898/s1. ^1^H, ^13^C and ^31^P NMR spectra, HRMS, and HPLC data of compounds **3aa–3ia**, **4aa** and Ru-L1.
