# Supplementary material for: Ru-Catalyzed Asymmetric Addition of Arylboronic Acids to Aliphatic Aldehydes via P-Chiral Monophosphorous Ligands
Source: Molecules. 2022 Jun 17;27(12):3898. doi: 10.3390/molecules27123898 (PMC9231018; doi:10.3390/molecules27123898)
Supplement: Supplementary file 1 [file molecules-27-03898-s001.zip › molecules-1751762-supplementaryproofed.pdf]

Supporting Information

Ru-Catalyzed Asymmetric Addition of Arylboronic Acids to Aliphatic Aldehydes via *P*-Chiral Monophosphorous Ligands

Rui Miao[a#], YanPing Xia[a#], YiFei Wei[a], Lu Ouyang[a]*, Renshi Luo[a,b]*

[a] *School of Pharmacy, Gannan Medical University, Ganzhou, 341000, Jiangxi Province, P. R. China.*

[b] *College of Chemistry and Environmental Engineering, Shaoguan University, Shaoguan, 512005, China.*

Table of Contents

A. General Procedure for asymmetric addition of aliphatic aldehydes................................ S2

B. General Procedure for racemic mixture of 3.. S2

C. General Procedure for NMR yields of 3aa and 4aa.. S2

D. General Procedure for Ru-L1.. S3

E. The original data of Scheme 5 and Scheme 6.. S3

F. Experiments for the amount of ruthenium catalyst...S5

G. NMR Spectra of products 3..S6

H. HPLC Spectra of products 3 ....S42

I. HRMS-ESI spectra for new compounds S75

A. General Procedure for asymmetric addition of aliphatic aldehydes

[$RuCl_2$(cymeme)]$_2$ (1 mol%), **L1** (2 mol%), as well as *p*-xylene (0.5 mL) were added in a 25.0 mL Schlenk tube successively under an N_2 atmosphere, which wasstirred at room temperature for 30 min to form the **Ru-L1** catalyst. Subsequently, a mixture of **1** (0.5 mmol), **2** (1.0 mmol), K_2CO_3 (2.0 equiv), *p*-xylene (1.0 mL), and H_2O (0.5 mL) were added to the Schlenk tube. Then the above reaction mixture was heated to 80ºC and stirred for 4h, after which the reaction was diluted with H_2O (15.0 mL), neutralized with HCl, and extracted with EtOAc (10.0 mL×3). The organic layer was washed with brine (10.0 mL×3) and dried over anhydrous $MgSO_4$. After removal of the solvent under vacuum to afford the crude product, which was purified by column chromatography on silica gel with hexanes or petroleum ether/ethyl acetate (5:1 to 20:1) to deliver the desired products **3**.

B. General Procedure for racemic mixture of 3

[$RuCl_2$(cymeme)]$_2$ (1 mol%), *rac*-**L1** (2 mol%), as well as *p*-xylene (0.5 mL) were added in a 25.0 mL Schlenk tube successively under an N_2 atmosphere, which was stirred at room temperature for 30 min to form the Ru-rac-**L1** catalyst. Subsequently, a mixture of **1** (0.5 mmol), **2** (1.0 mmol), K_2CO_3 (2.0 equiv), *p*-xylene (1.0 mL), and H_2O (0.5 mL) were added to the Schlenk tube. Then the above reaction mixture was heated to 80 ºC and stirred for 4h, after which the reaction was diluted with H_2O (15.0 mL), neutralized with HCl, and extracted with EtOAc (10.0 mL×3). The organic layer was washed with brine (10.0 mL×3) and dried over anhydrous $MgSO_4$. After removal of the solvent under vacuum to afford the crude product, which was purified by column chromatography on silica gel with hexanes or petroleum ether/ethyl acetate (5:1 to 20:1) to deliver the racemic product **3**.

C. General Procedure for NMR yields of 3aa and 4aa

When reaction was finished, an exact amount of internal standarddimethyl terephthalate (99.93%purity, 14.4-16.3 mg) was added in the Schlenk tube, which was diluted with H_2O (15.0 mL), neutralized with HCl, and extracted with EtOAc (10.0 mL×3).The combined organic layer was washed with brine (10.0 mL×3) and dried over

anhydrous $MgSO_4$. After removal of the solvent under vacuum to afford the crude product.The NMR yields of **3aa** and **4aa** in Table 1, Table 2, Scheme5 and Scheme 6 were measured with substrate **1a** (99.2% purity, 0.5 mmol) as benchmark.

D. General Procedure for Ru-L1

Ru-L1: To a Schlenk flask was charged **L1** (33.3 mg, 0.1 mmol) and 1,2-dichloroethane (2 mL) followed by $[RuCl_2(cymene)]_2$ (30.0 mg, 0.05 mmol), and the resulting dark red solution was stirred at reflux under nitrogen for 18 h. Solvent was removed under vacuum to yield a red solid. The crude product was purified by
flash chromatography (eluent, ethyl acetate/hexanes 2:1) to yield Ru-L1.

E. The original data of Schem 7 and Schem 8

$[RuCl_2(cymeme)]_2$ (1 mol%), **L1** (2 mol%), as well as *p*-xylene (0.5 mL) were added in a 25.0 mL Schlenk tube successively under an N_2 atmosphere, which was stirred at room temperature for 30 min to form the **Ru-L1** catalyst. Subsequently, a mixture of **1a** (66 μl, 0.5 mmol), **2a** (136 mg, 1.0 mmol), K_2CO_3 (138.2 mg, 1.0 mol, 2.0 equiv), *p*-xylene (1.0 mL), and H_2O (0.5 mL) were added, which was then stired at 80 °C in oil bath, the "stopwatch" was started.

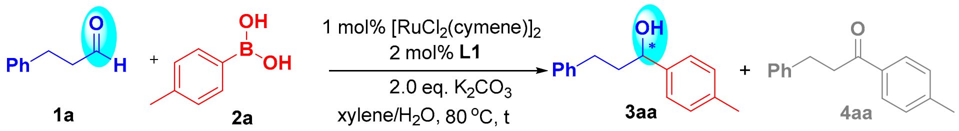

t/min	Conversion of **1a** (%)	NMR yield of **3aa** (%)	Isolated yield of **3aa** (%)	NMR yield of **4aa** (%)	*ee*of **3aa** (%)
2	96	89.03	85.7	2.49	79.95
4	99	89.89	88.49	2.79	83.76
8	99	93.05	90.26	3.67	83.67
40	100	95.15	91.15	2.92	84.87
80	100	94.71	91.41	3.71	83.96
120	100	95.68	88.58	3.67	83.94
240	100	96.55	95.32	3.3	85.8
720	100	94.97	90.26	4.5	84.83

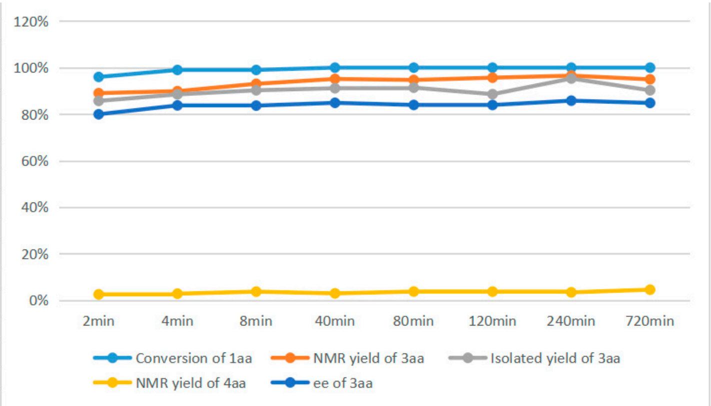

Scheme S1. Plots for relationship of time, yield and enantioselectivity.

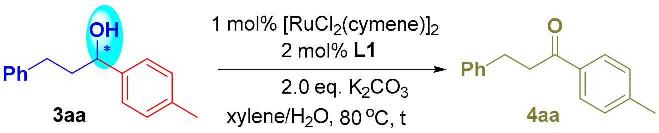

t/h	NMR yield of **4aa** （%）	*ee* of **3aa** (%)
0.0	0.00	81.64
0.5	1.51	79.57
1.0	1.75	79.38
1.5	2.14	78.88
2.0	2.53	77.35
3.0	3.36	77.21
4.0	4.31	76.08

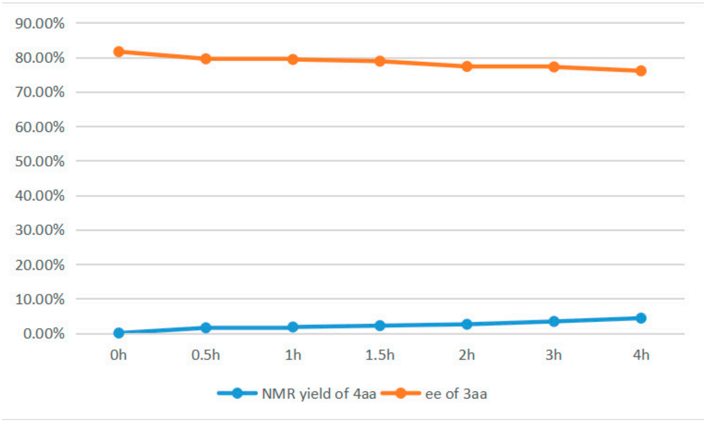

Scheme S2. Plots for relationship of time, enantioselectivity and ketone formation.

F. Experiments for the amount of ruthenium catalyst

Table S1. Variation of the amount of ruthenium catalyst.[a]

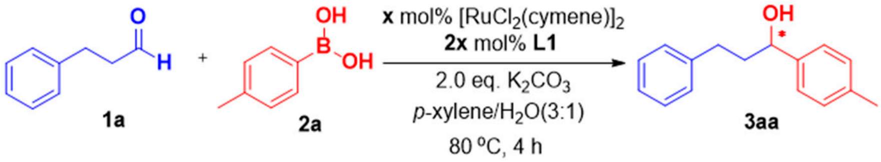

Entry	x mol(%)	Conv.(%)	Isolate yield of **3aa**(%)	*er* of **3aa**(%)[b]
1	1.0	>99	95.5	93:7
2	0.5	>99	95.3	92:8
3	0.1	>99	95.4	92:8
4	0.01	90	84.7	92:8

[a] Performed with **1a** (0.5 mmol), **2a** (2.0 eq.), $[RuCl_2(cymene)]_2$ (**x** mol%), **L1** (**2x** mol%), K_2CO_3 (2.0 eq.), *P*-xylene/H_2O (3:1) (2.0 mL), at 80°C under N_2 atmosphere for 4h. [b] Determined by Chiral OJ-H column.

G. NMR Spectra of products 3

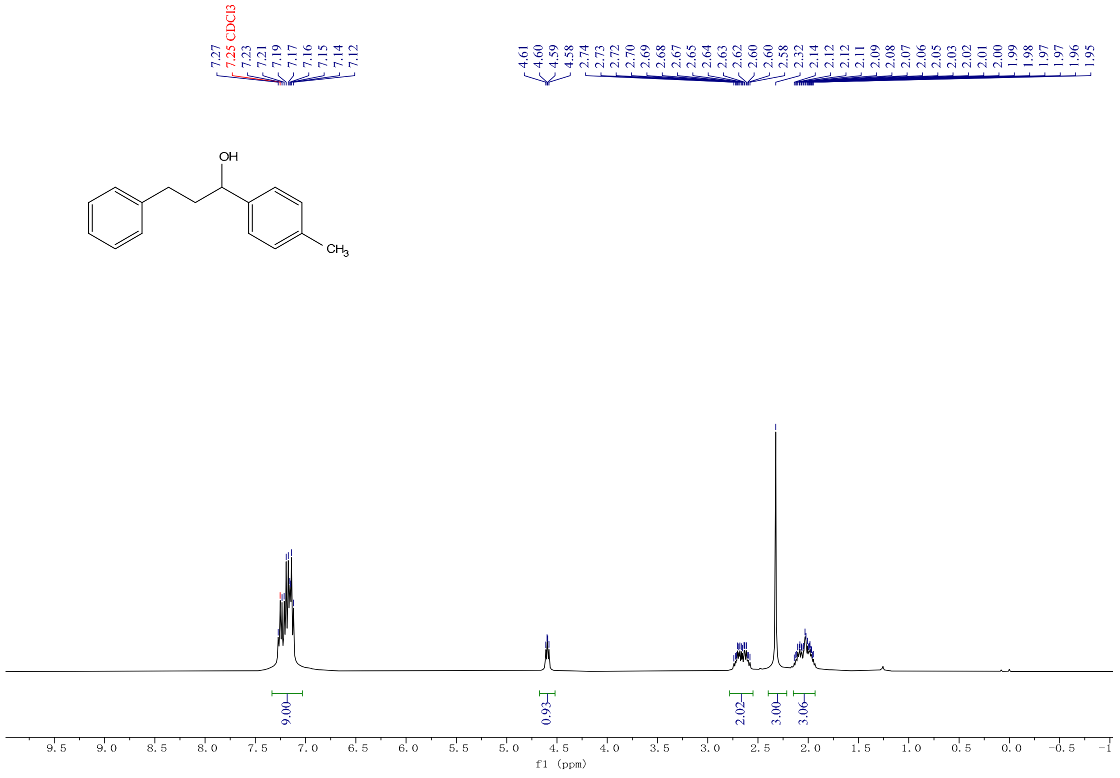

Figure S1: ^{1}H NMR spectrum of 3-phenyl-1-(p-tolyl)propan-1-ol (3aa)

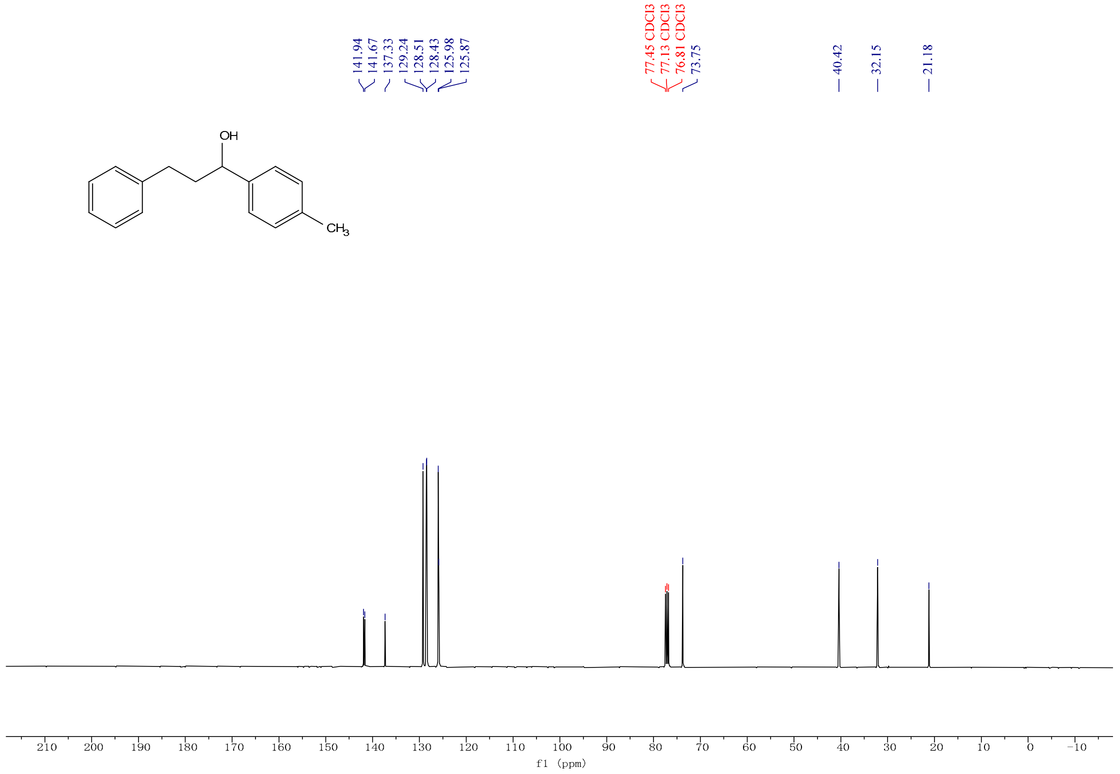

Figure S2:^{13}CNMR spectrum of 3-phenyl-1-(p-tolyl)propan-1-ol (3aa)

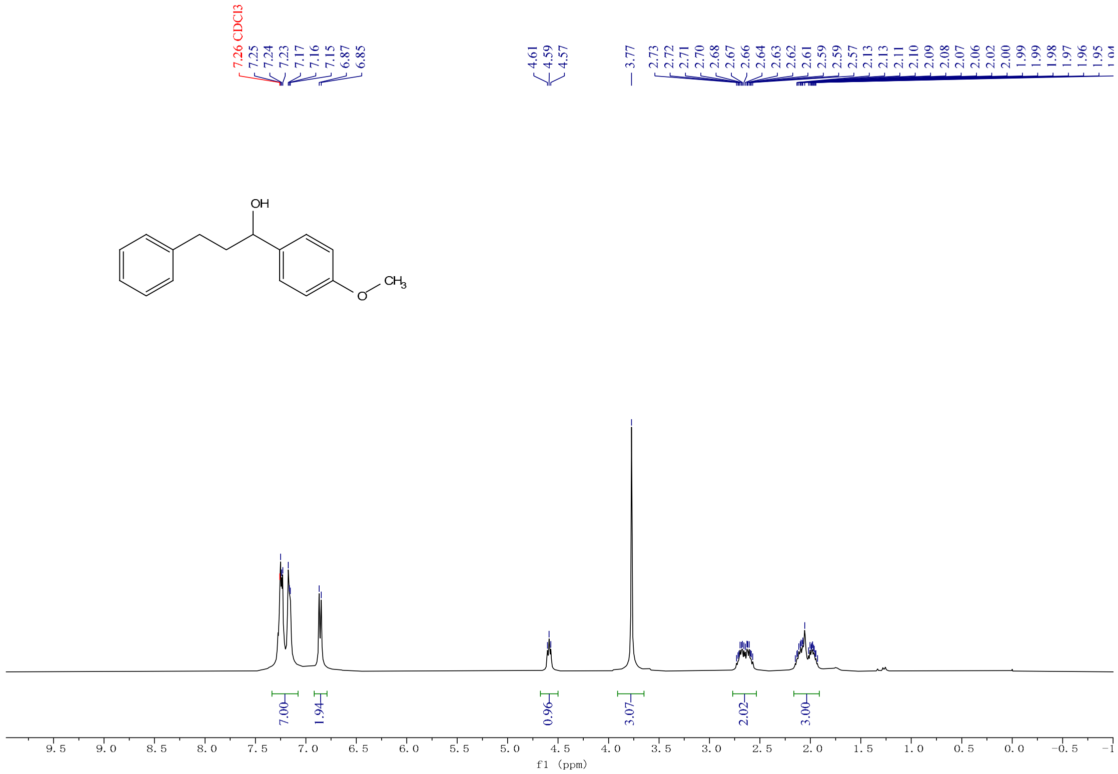

Figure S3: ^{1}H NMR spectrum of 1-(4-methoxyphenyl)-3-phenylpropan-1-ol (3ab)

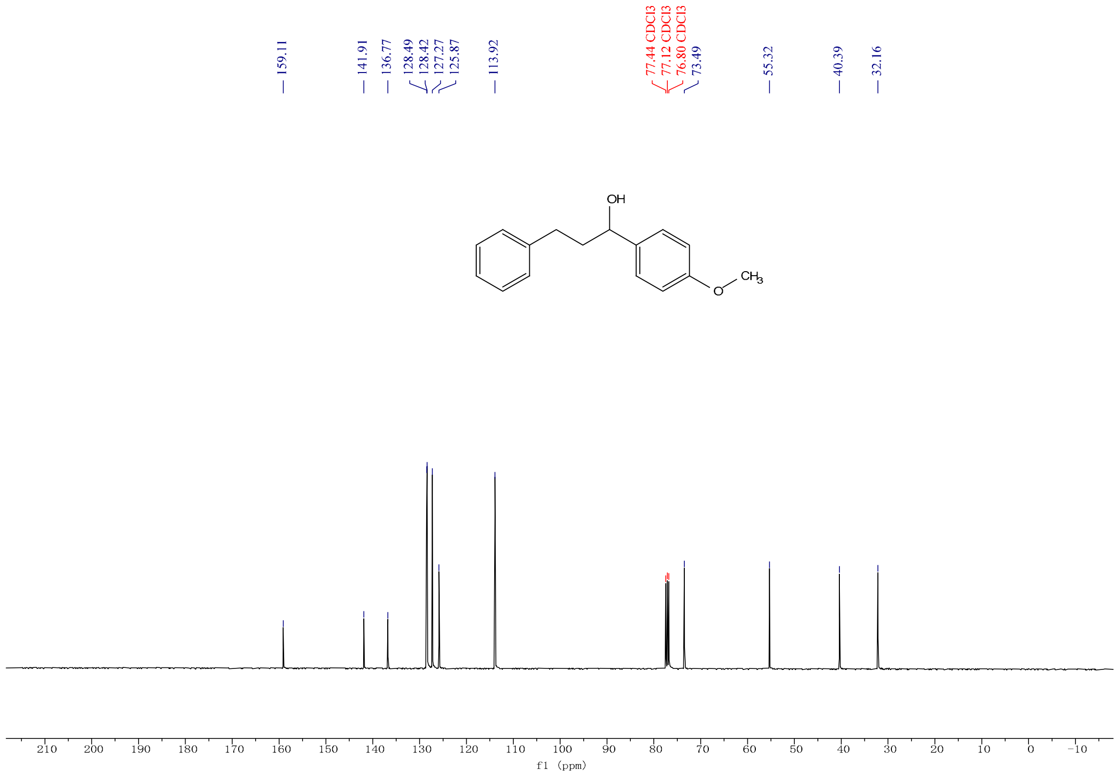

Figure S4: ^{13}CNMR spectrum of 1-(4-methoxyphenyl)-3-phenylpropan-1-ol (3ab)

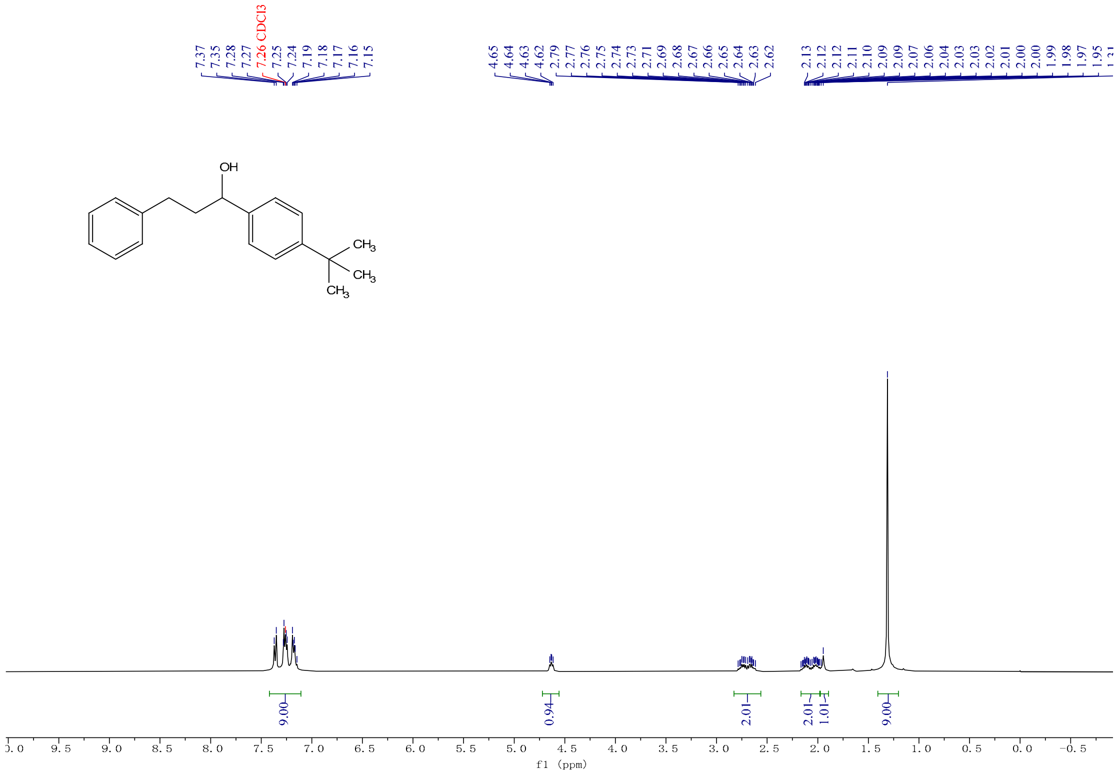

Figure S5: ^{1}H NMR spectrum of 1-(4-(tert-butyl)phenyl)-3-phenylpropan-1-ol (3ac)

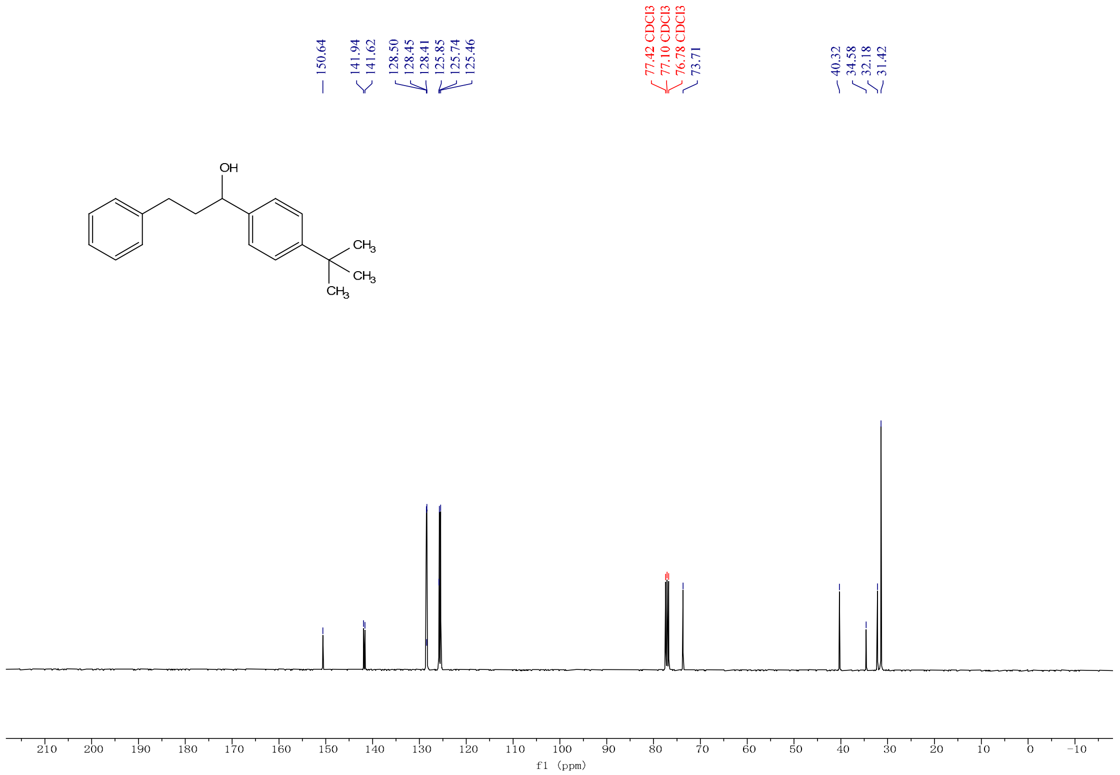

Figure S6: ^{13}CNMR spectrum of 1-(4-(tert-butyl)phenyl)-3-phenylpropan-1-ol (3ac)

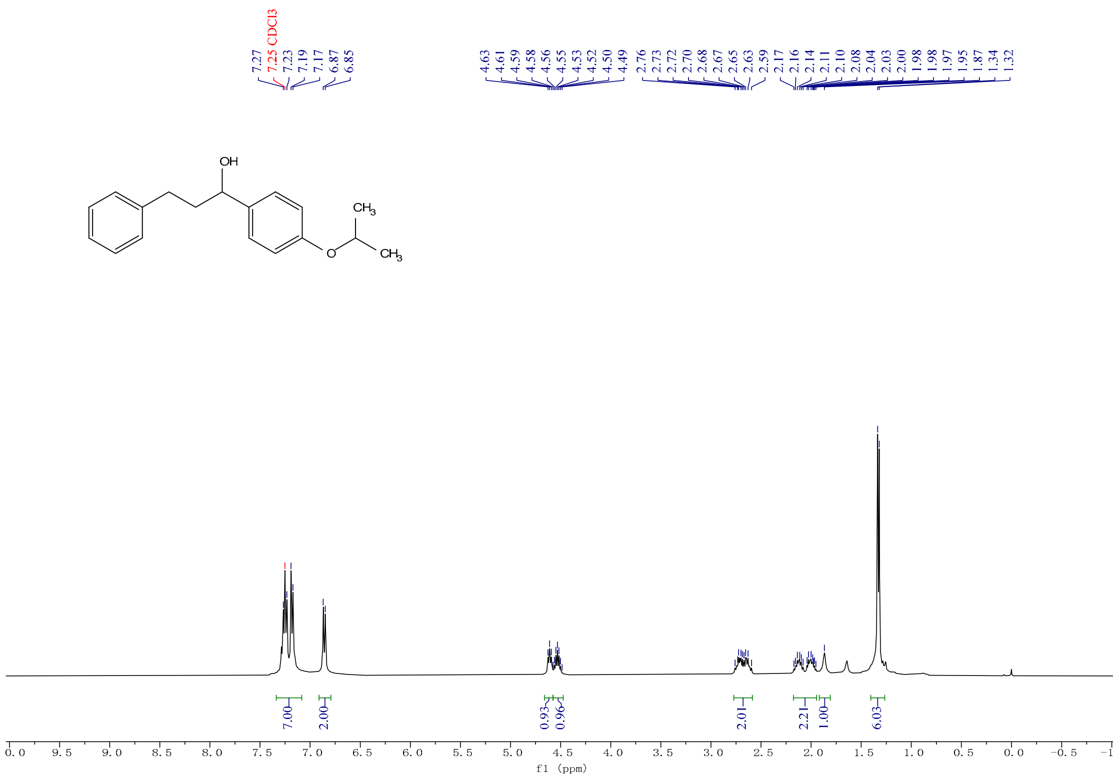

Figure S7: ¹H NMR spectrum of 1-(4-isopropoxyphenyl)-3-phenylpropan-1-ol (3ad)

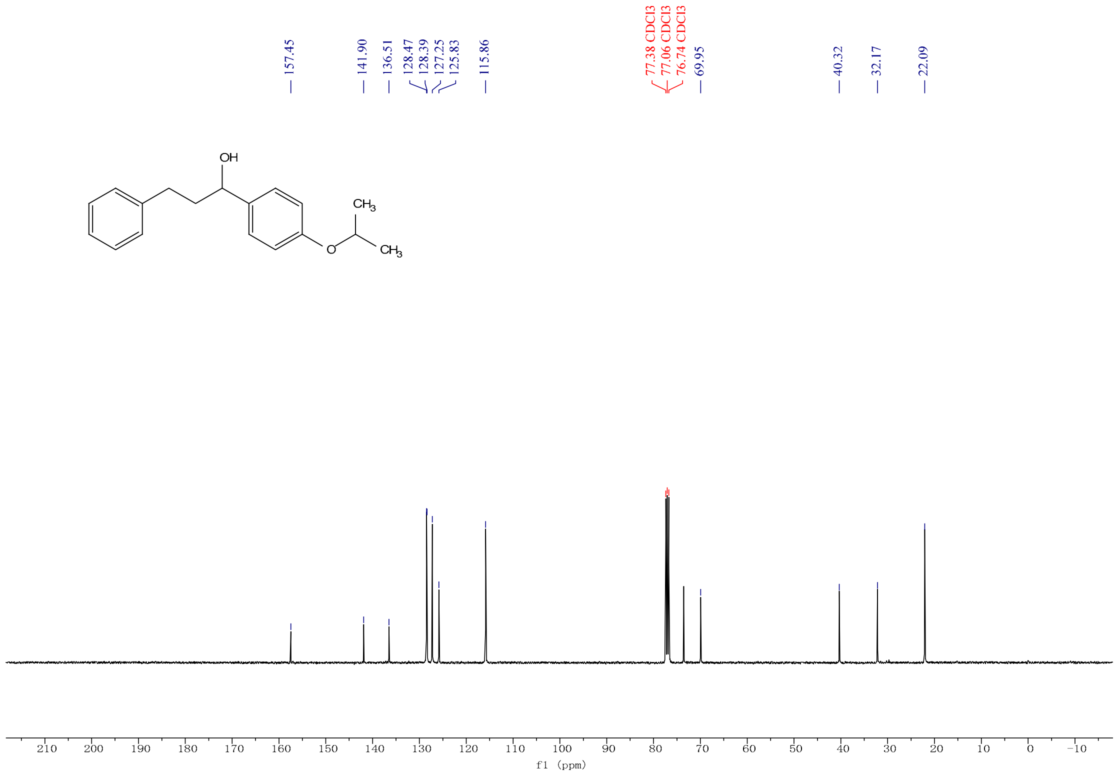

Figure S8: ¹³CNMR spectrum of 1-(4-isopropoxyphenyl)-3-phenylpropan-1-ol (3ad)

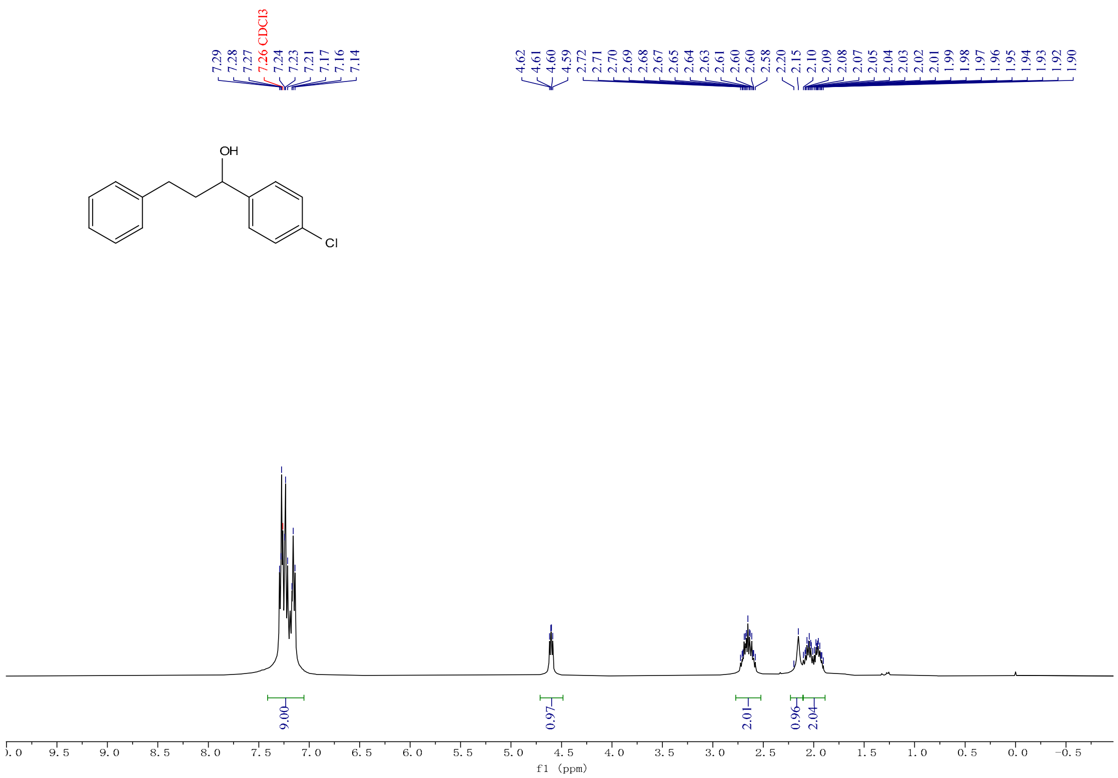

Figure S9: ^{1}H NMR spectrum of 1-(4-chlorophenyl)-3-phenylpropan-1-ol (3ae)

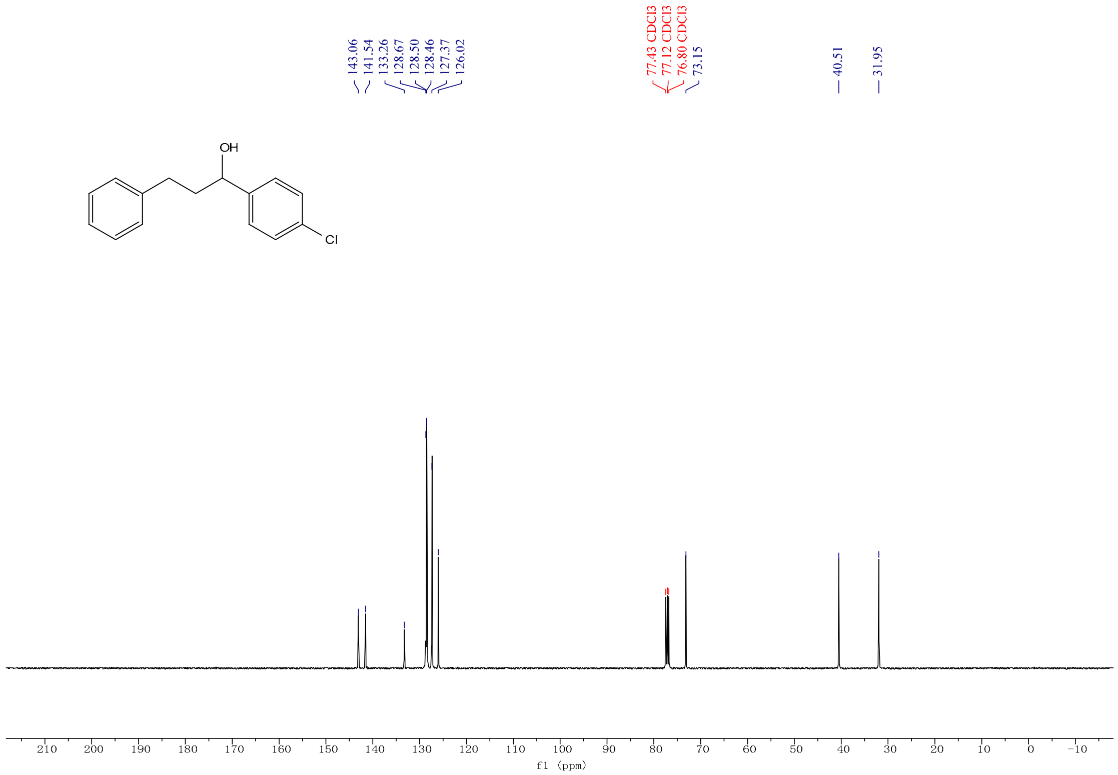

Figure S10: ^{13}CNMR spectrum of 1-(4-chlorophenyl)-3-phenylpropan-1-ol (3ae)

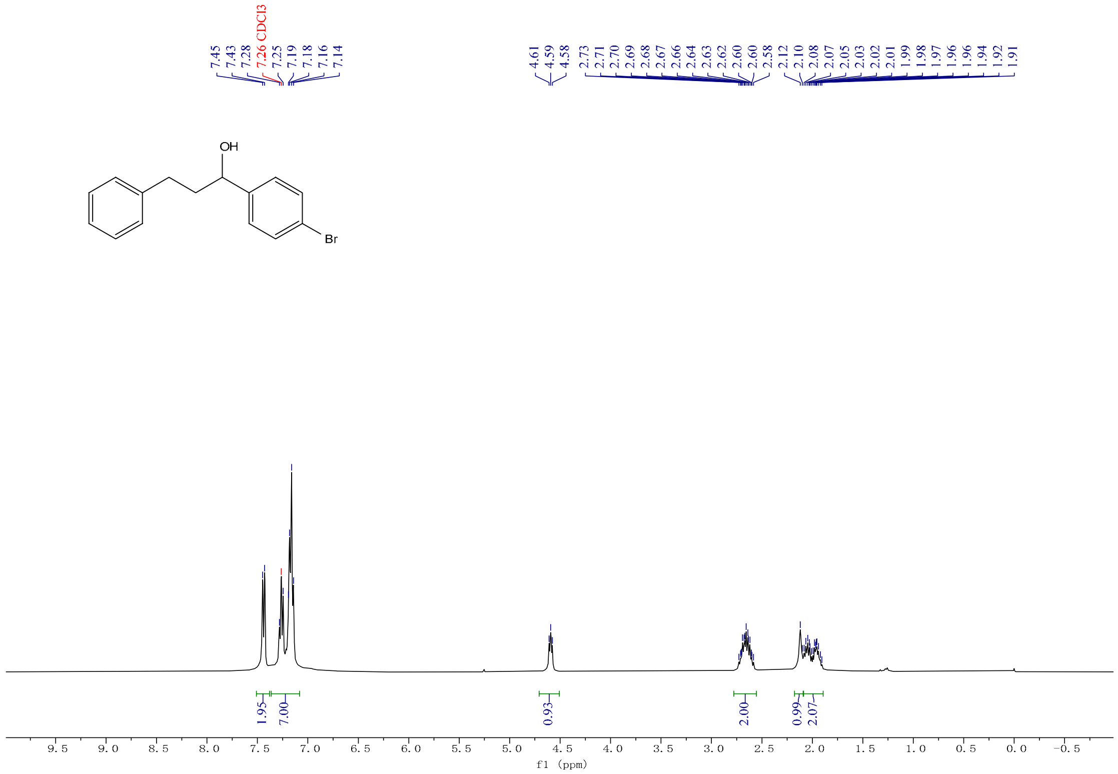

Figure S11: ^{1}H NMR spectrum of 1-(4-bromophenyl)-3-phenylpropan-1-ol (3af)

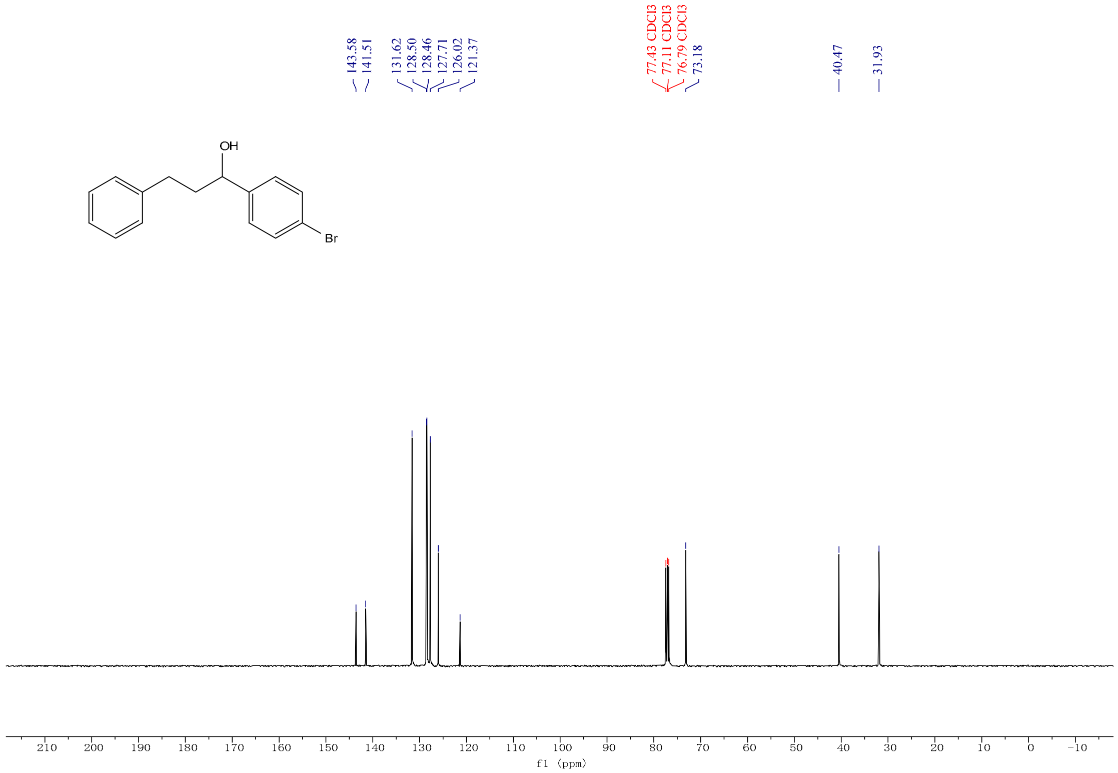

Figure S12: ^{13}CNMR spectrum of 1-(4-bromophenyl)-3-phenylpropan-1-ol (3af)

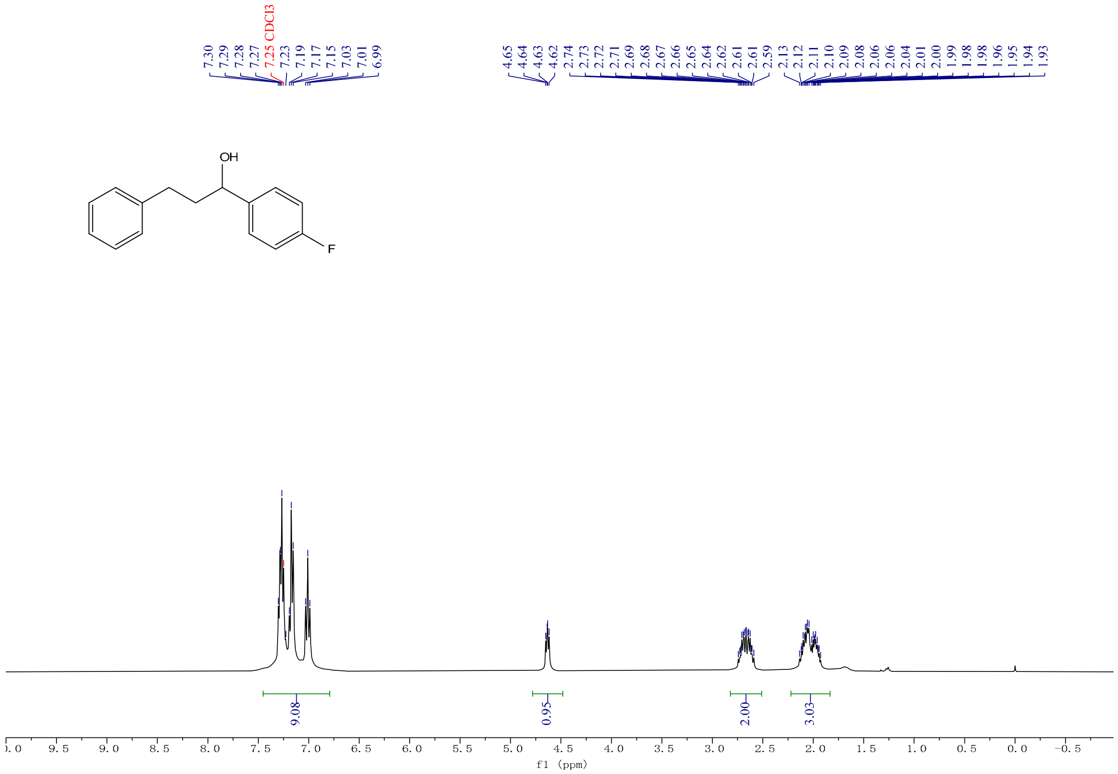

Figure S13: ^{1}H NMR spectrum of 1-(4-fluorophenyl)-3-phenylpropan-1-ol (3ag)

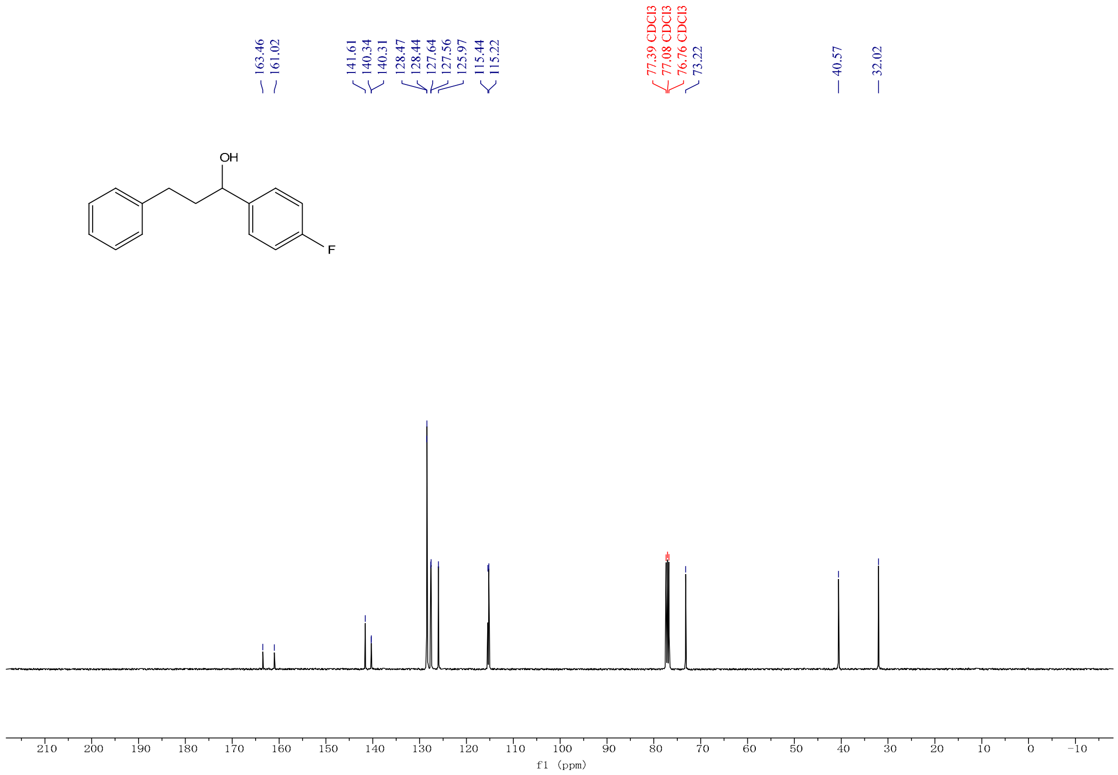

Figure S14: ^{13}CNMR spectrum of 1-(4-fluorophenyl)-3-phenylpropan-1-ol (3ag)

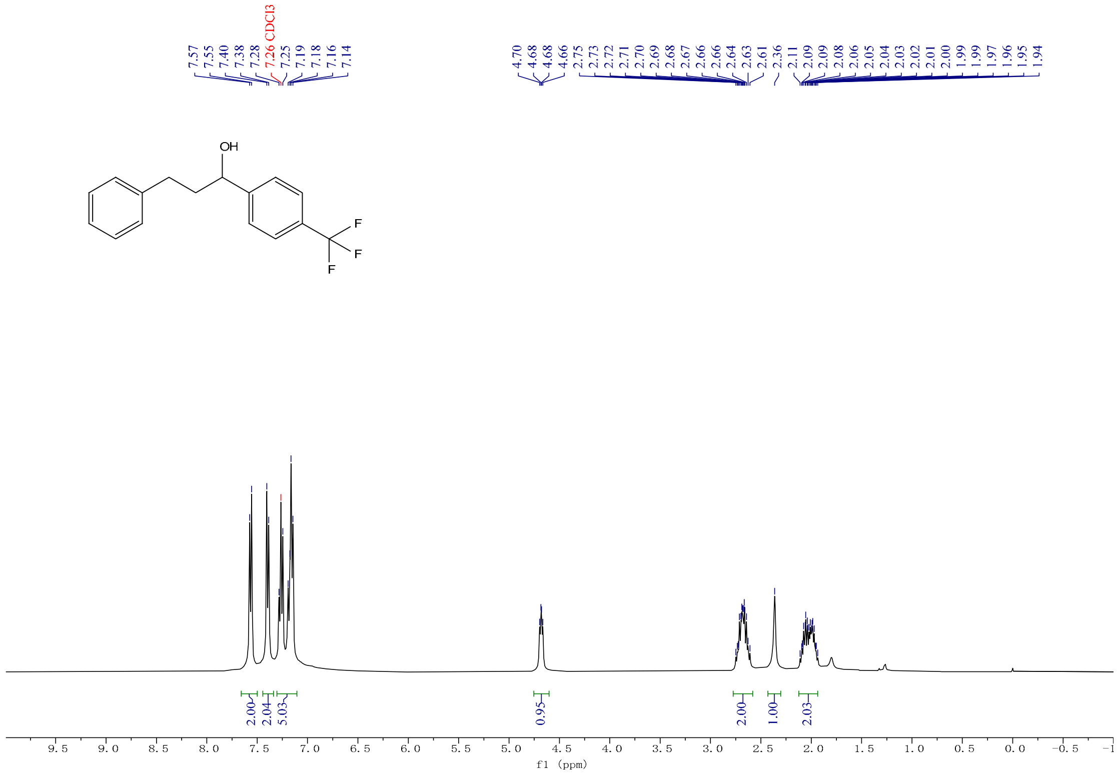

Figure S15: ^{1}H NMR spectrum of 3-phenyl-1-(4-(trifluoromethyl)phenyl)propan-1-ol (3ah)

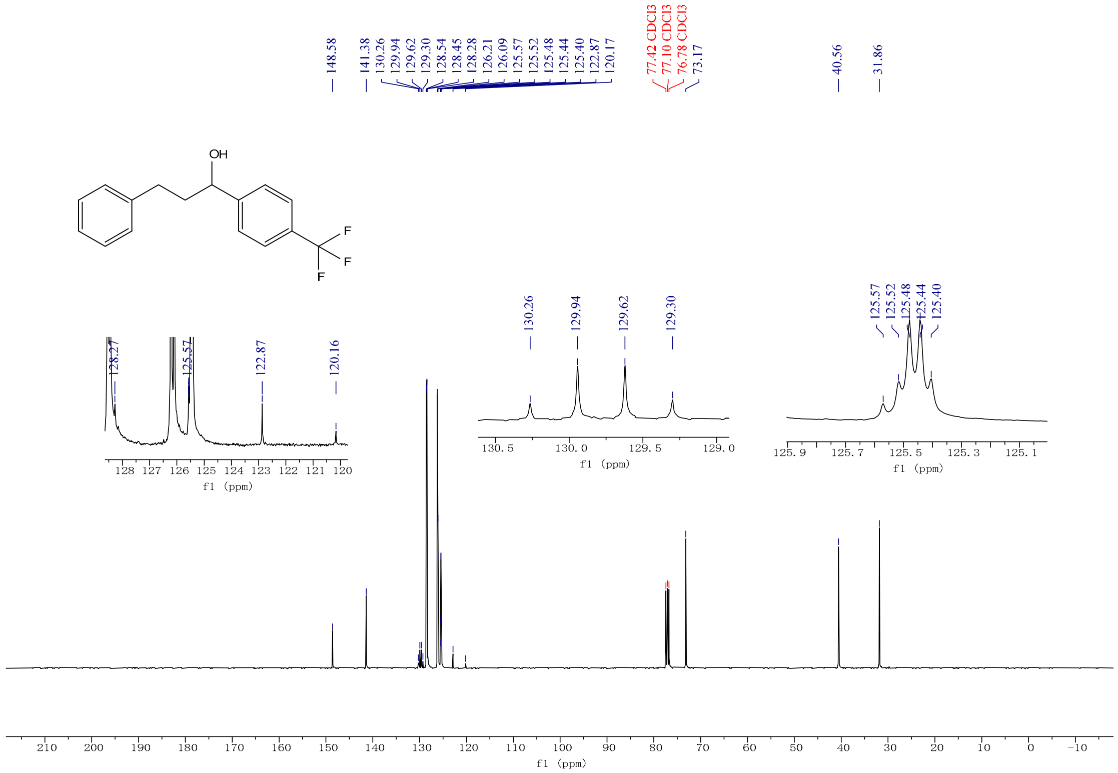

Figure S16: ^{13}CNMR spectrum of 3-phenyl-1-(4-(trifluoromethyl)phenyl)propan-1-ol (3ah)

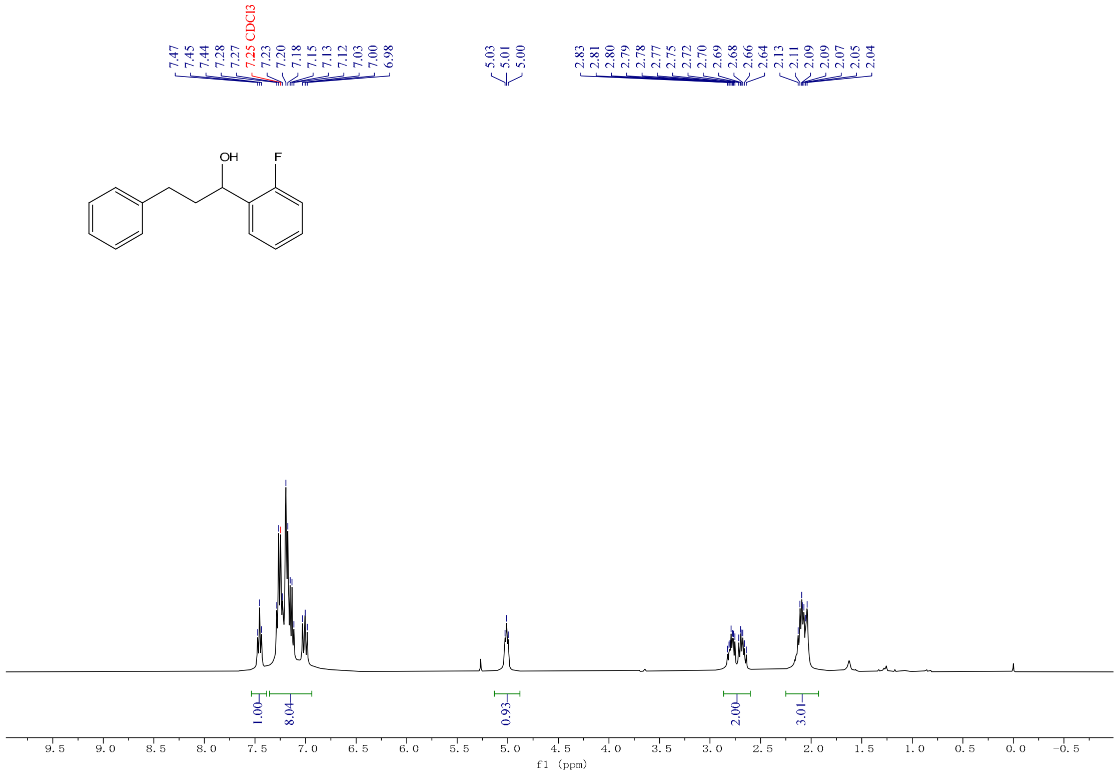

Figure S17: ^{1}H NMR spectrum of 1-(2-fluorophenyl)-3-phenylpropan-1-ol (3ai)

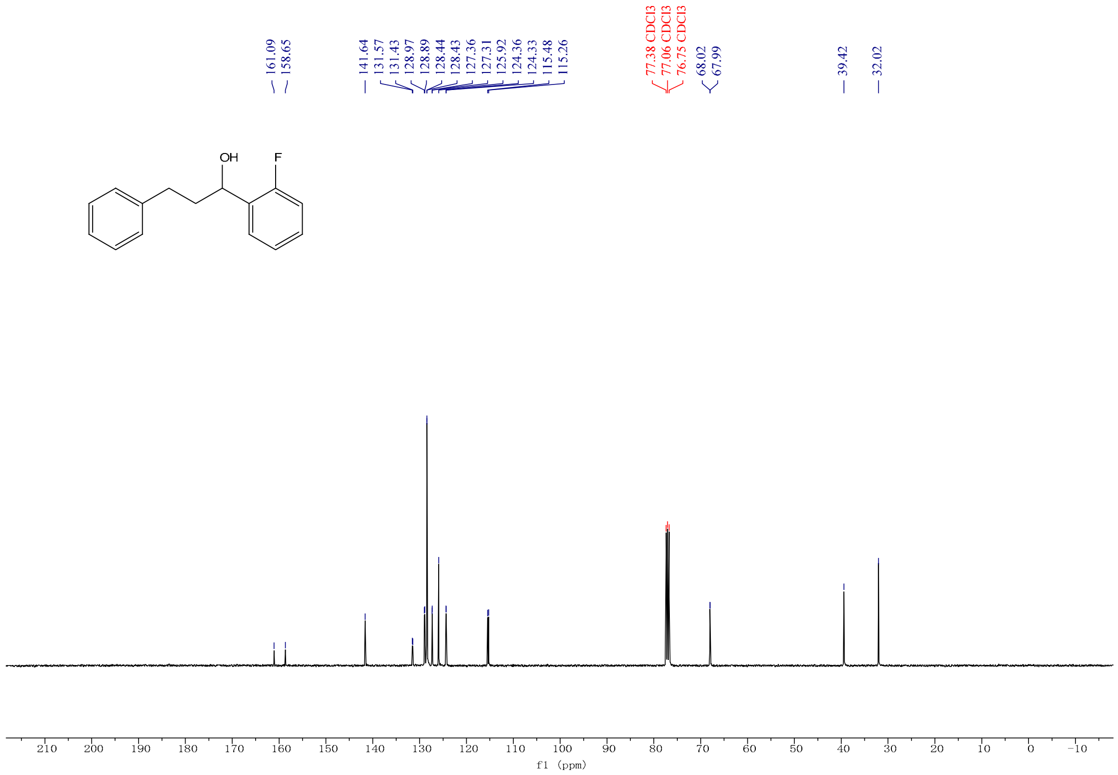

Figure S18: ^{13}CNMR spectrum of 1-(2-fluorophenyl)-3-phenylpropan-1-ol (3ai)

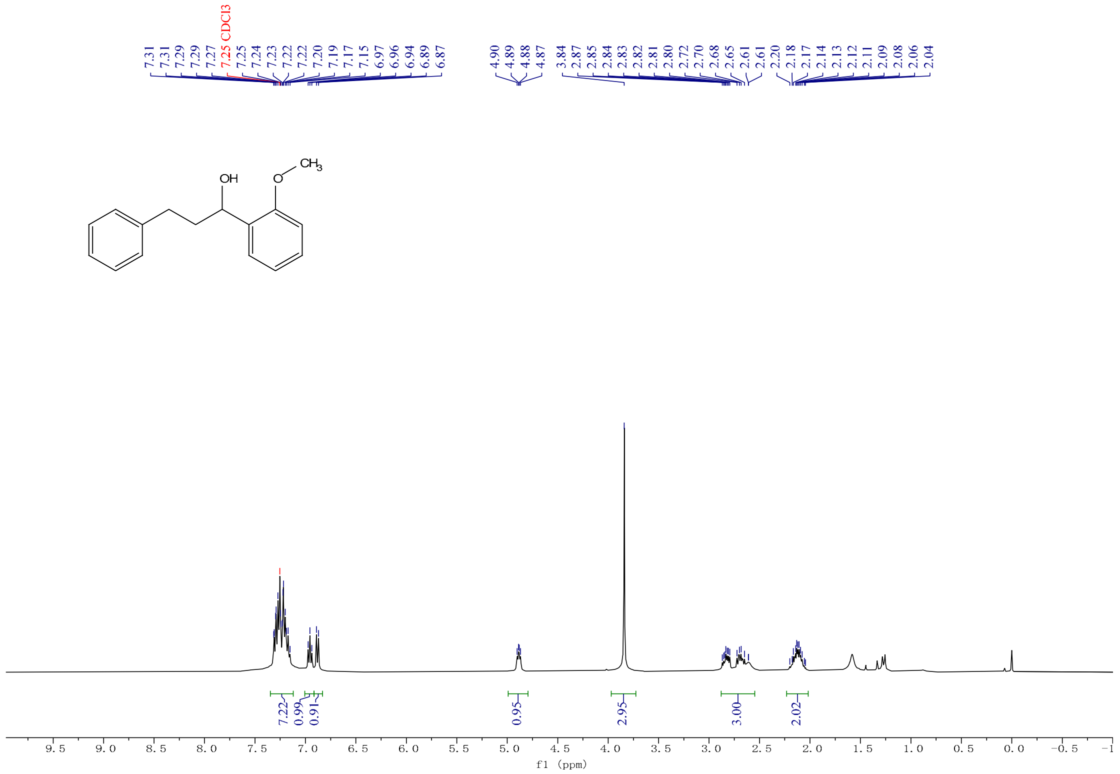

Figure S19: ^{1}H NMR spectrum of 1-(2-methoxyphenyl)-3-phenylpropan-1-ol (3aj)

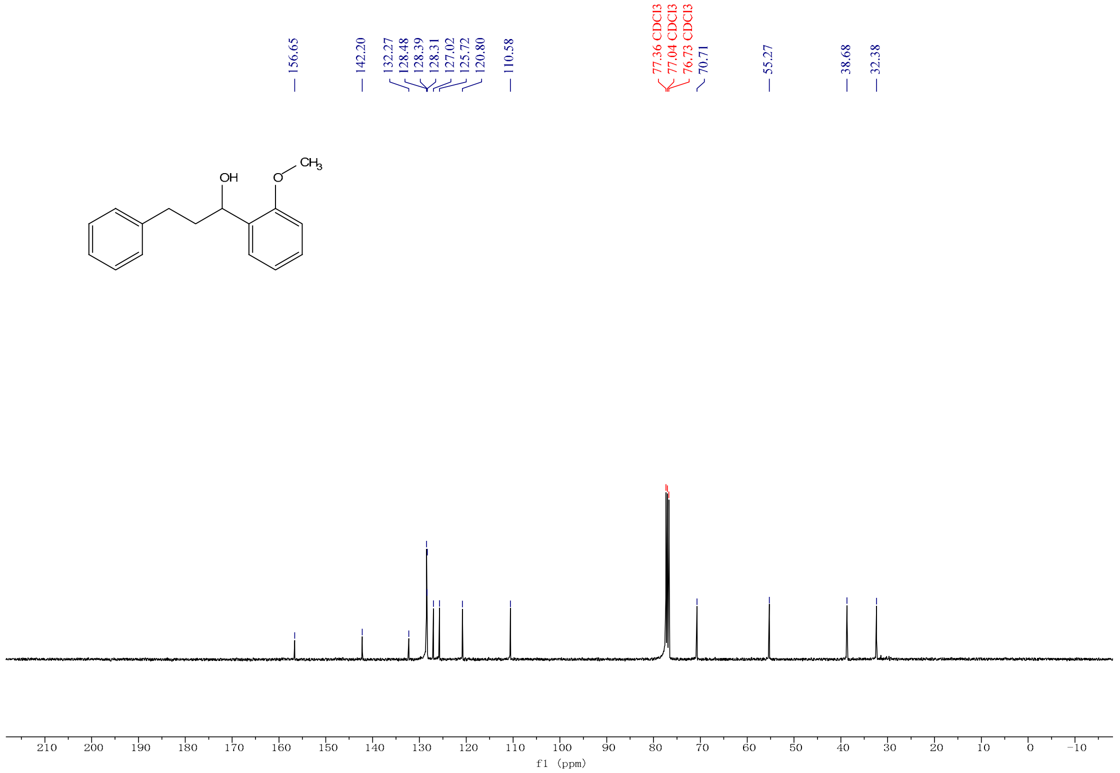

Figure S20: ^{13}CNMR spectrum of 1-(2-methoxyphenyl)-3-phenylpropan-1-ol (3aj)

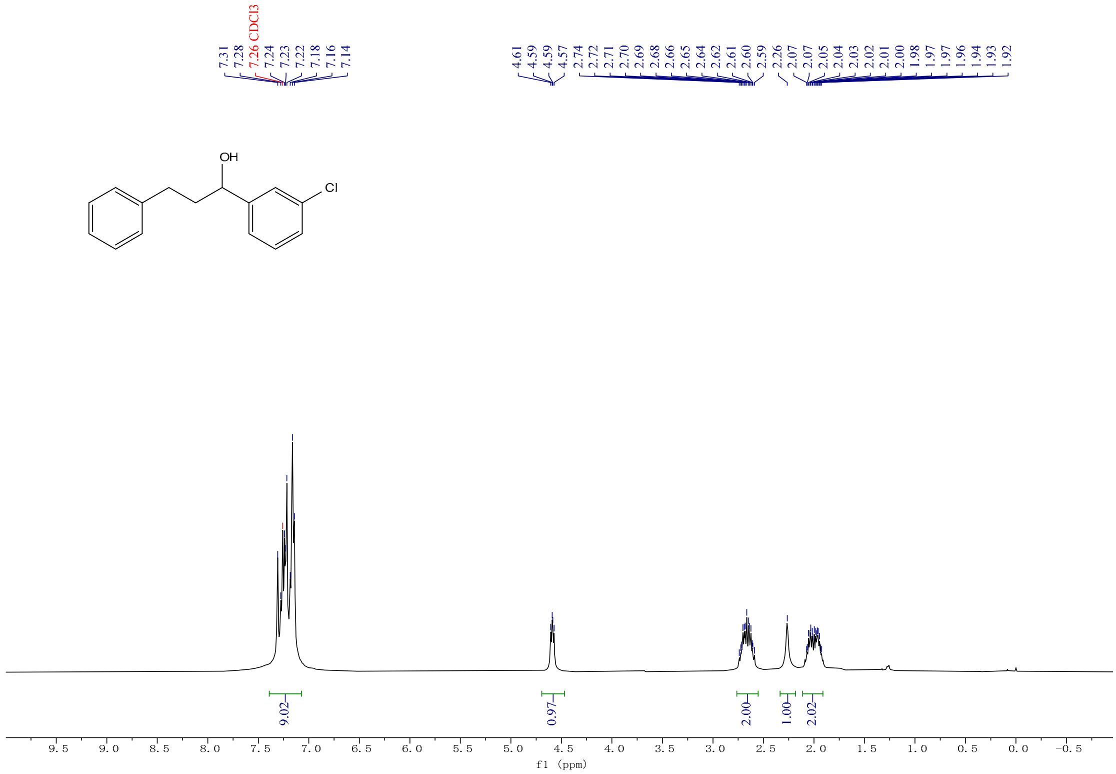

Figure S21: ^{1}H NMR spectrum of 1-(3-chlorophenyl)-3-phenylpropan-1-ol (3ak)

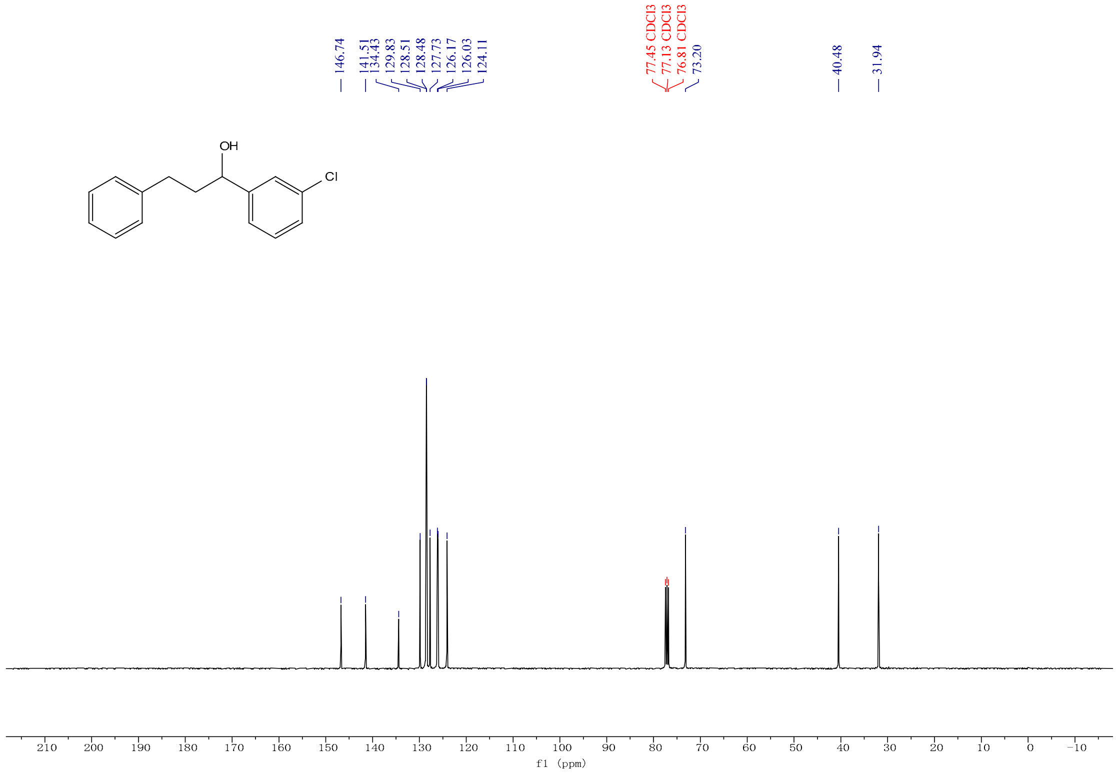

Figure S22: ^{13}CNMR spectrum of 1-(3-chlorophenyl)-3-phenylpropan-1-ol (3ak)

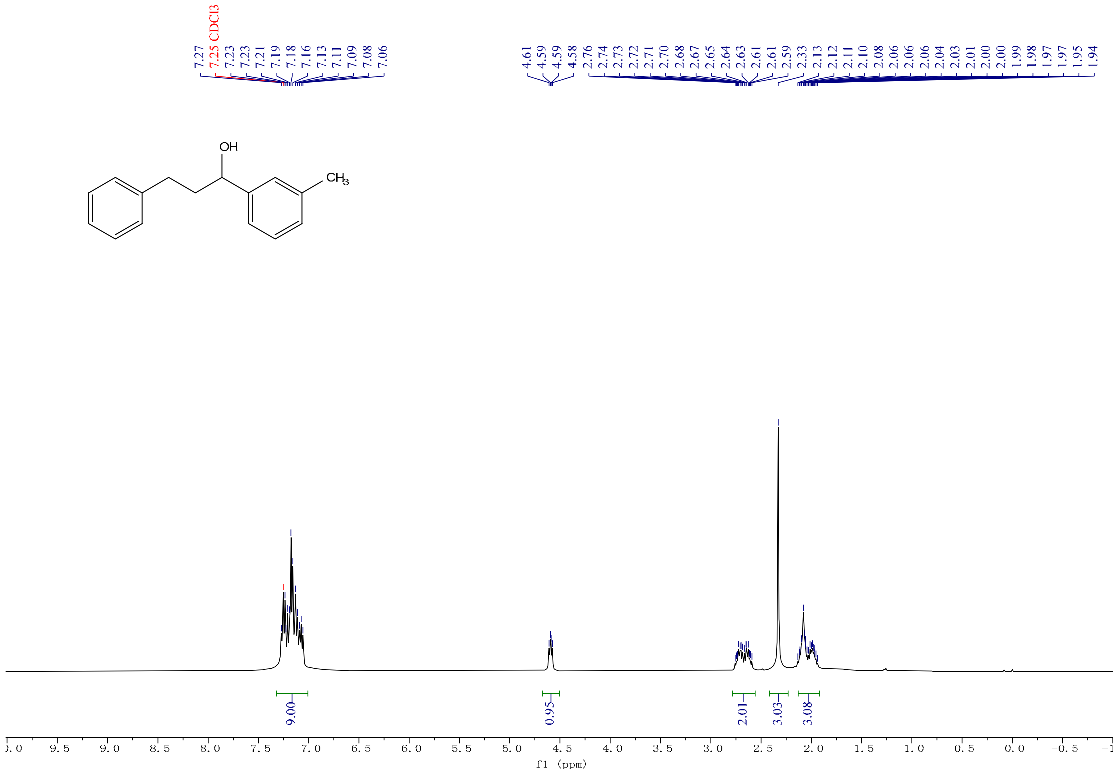

Figure S23: ^{1}H NMR spectrum of 3-phenyl-1-(m-tolyl)propan-1-ol (3al)

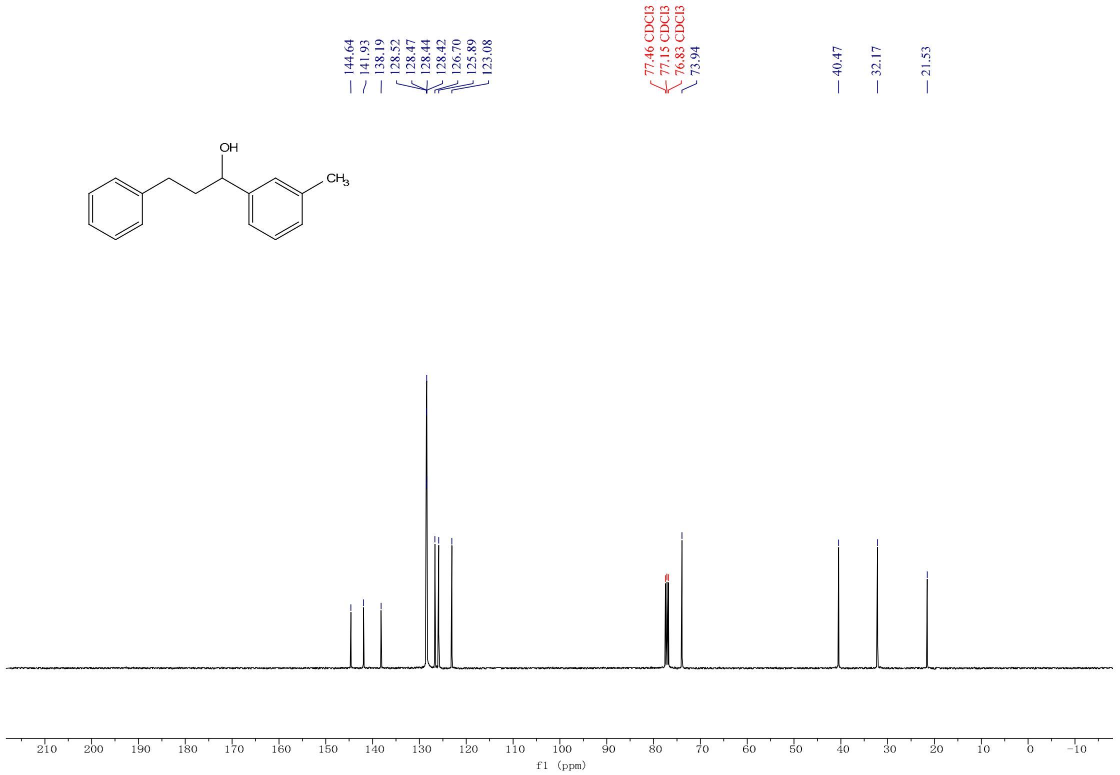

Figure S24: ^{13}CNMR spectrum of 3-phenyl-1-(m-tolyl)propan-1-ol (3al)

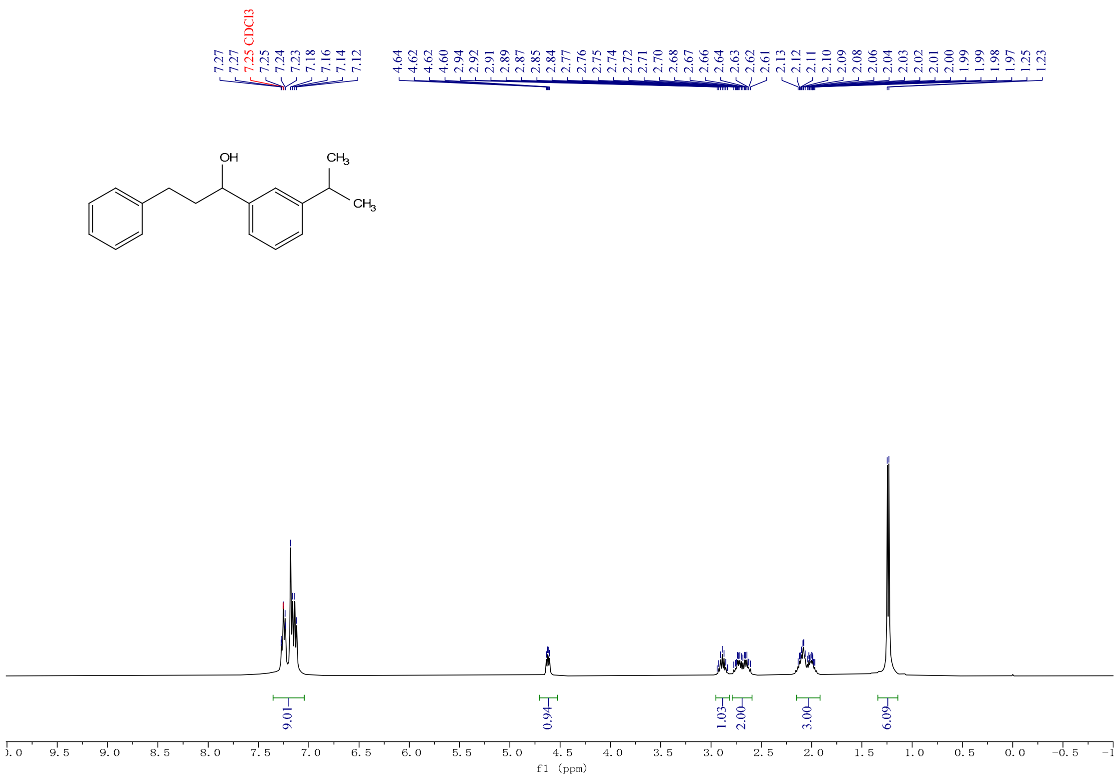

Figure S25: ^{1}H NMR spectrum of 1-(3-isopropylphenyl)-3-phenylpropan-1-ol (3am)

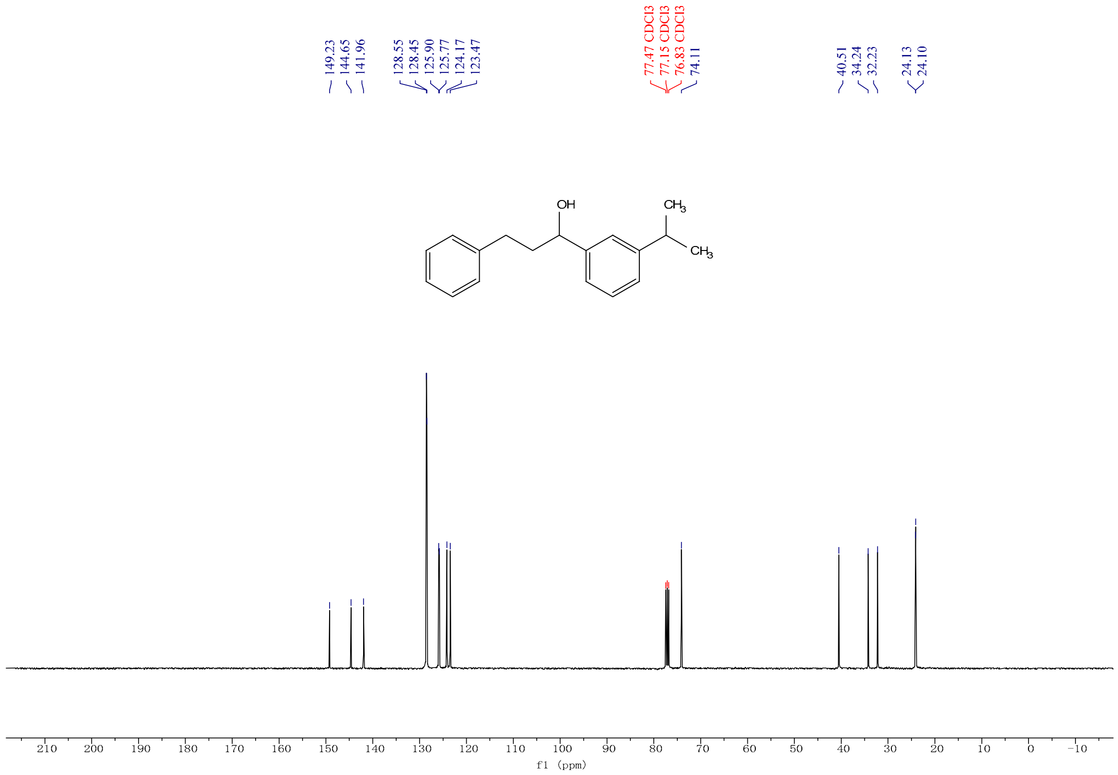

Figure S26: ^{13}CNMR spectrum of 1-(3-isopropylphenyl)-3-phenylpropan-1-ol (3am)

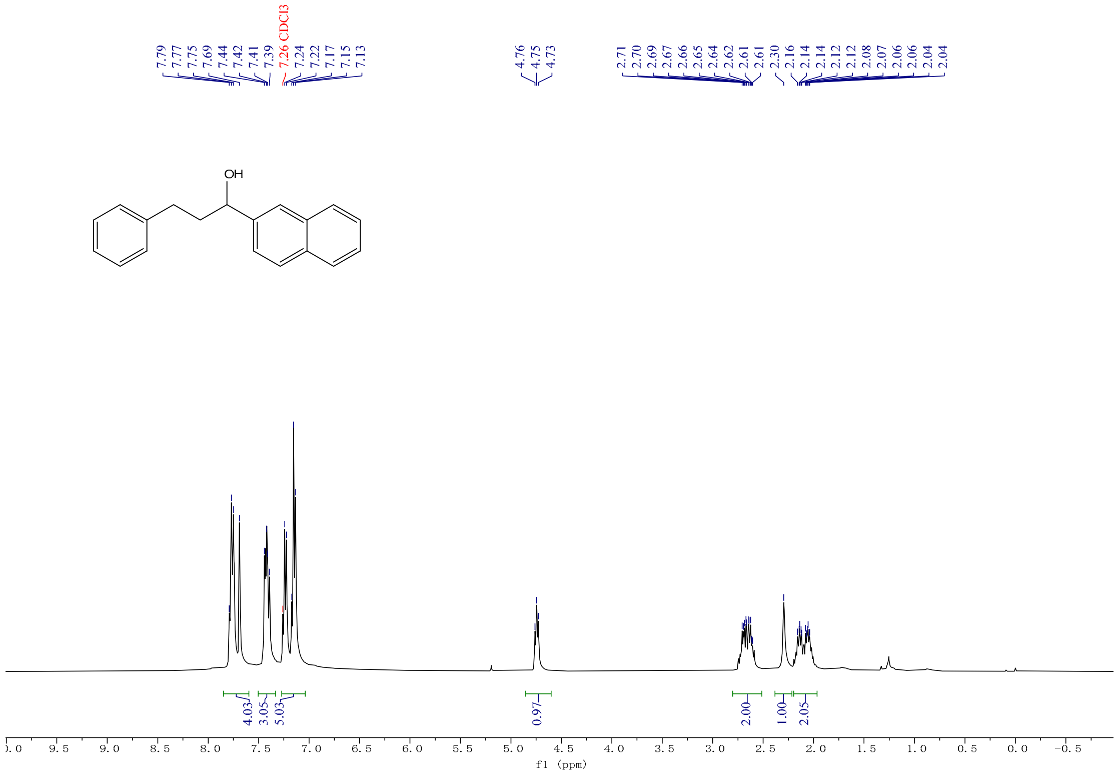

Figure S27: ^{13}CNMR spectrum of 1-(naphthalen-2-yl)-3-phenylpropan-1-ol (3an)

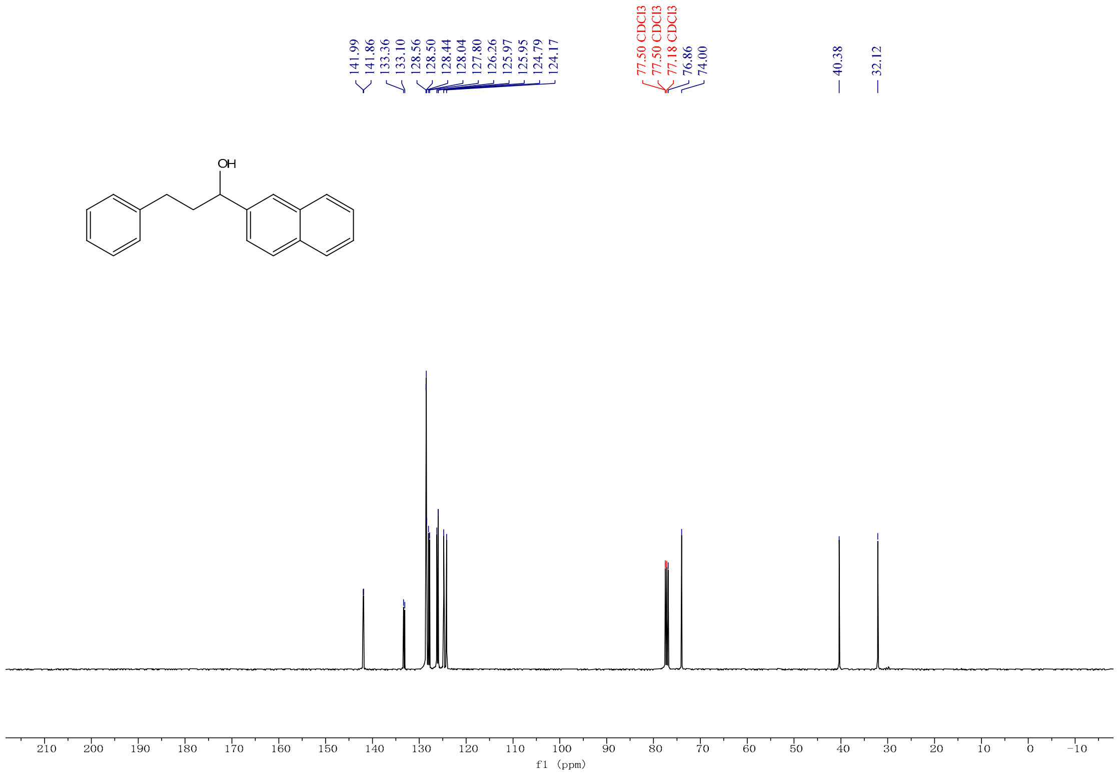

Figure S28: ^{1}H NMR spectrum of 1-(naphthalen-2-yl)-3-phenylpropan-1-ol (3an)

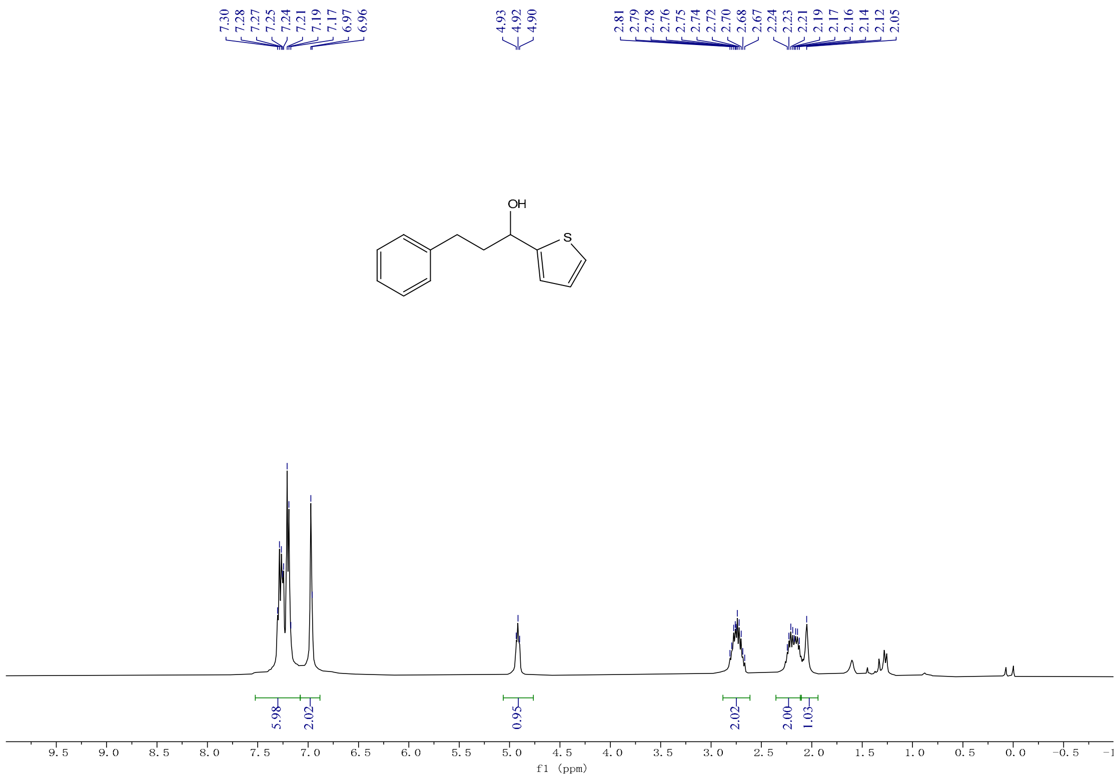

Figure S29: ^{1}H NMR spectrum of 3-phenyl-1-(thiophen-2-yl)propan-1-ol (3ao)

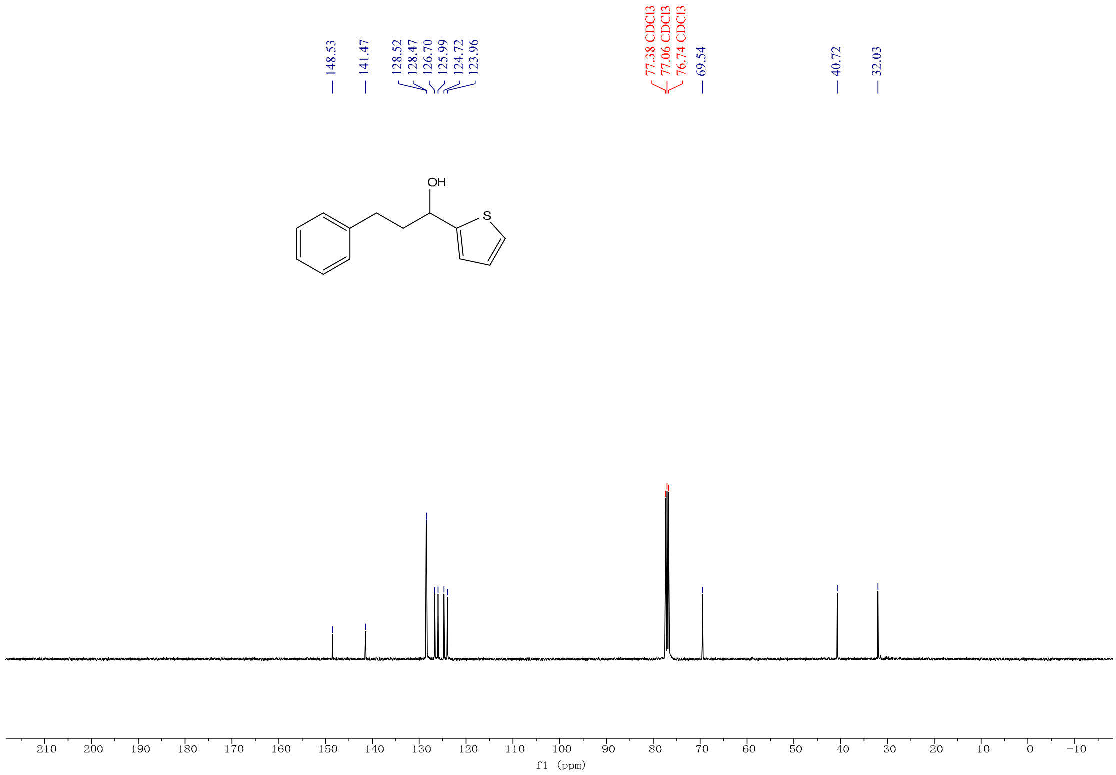

Figure S30: ^{13}CNMR spectrum of 3-phenyl-1-(thiophen-2-yl)propan-1-ol (3ao)

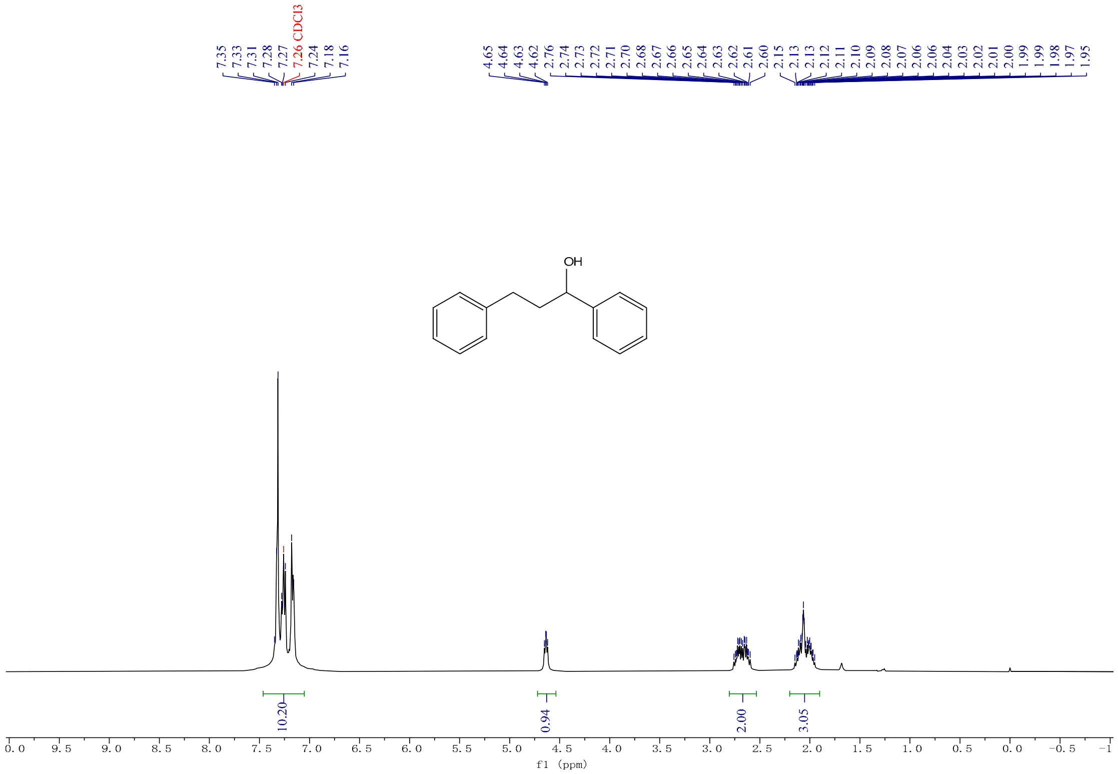

Figure S31: ^{1}H NMR spectrum of 1,3-diphenylpropan-1-ol (3ap)

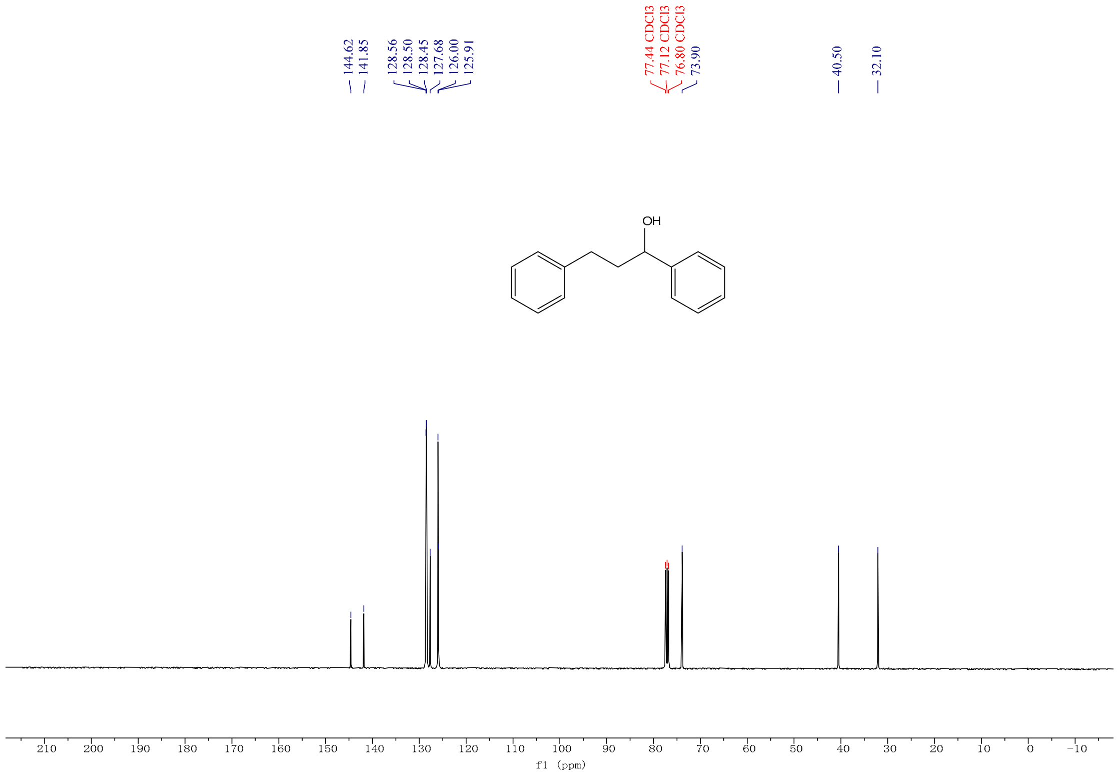

Figure S32: ^{13}CNMR spectrum of 1,3-diphenylpropan-1-ol (3ap)

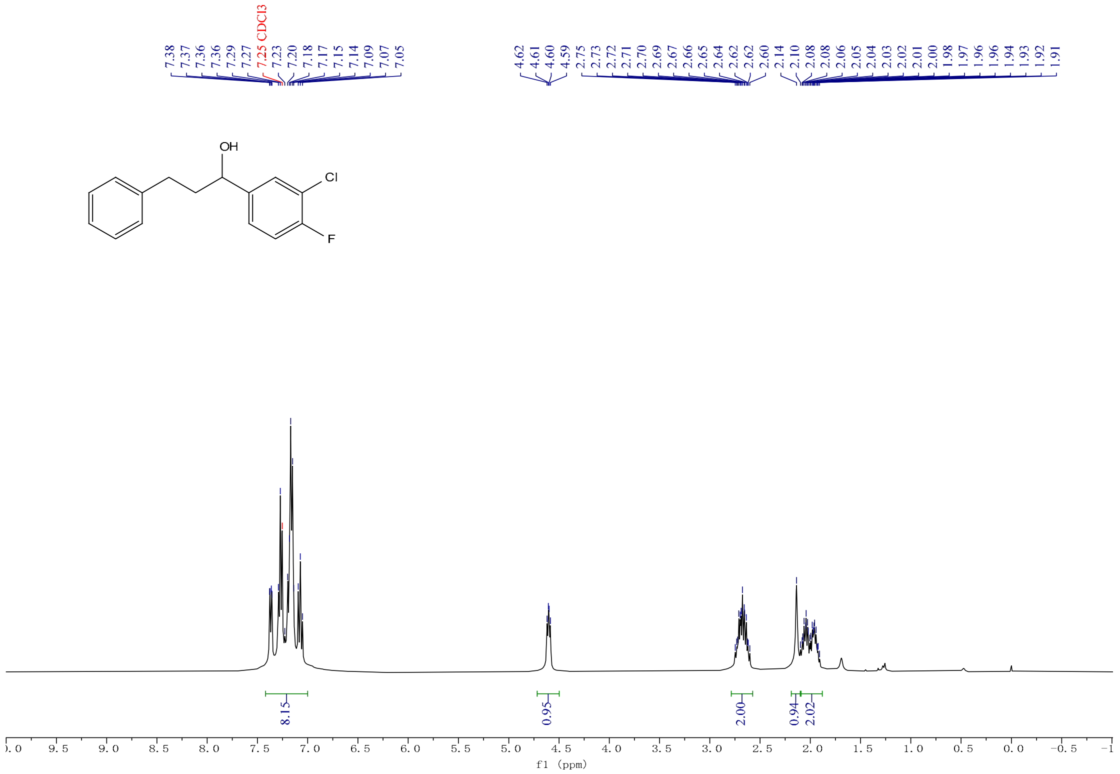

Figure S33: ^{1}H NMR spectrum of 1-(3-chloro-4-fluorophenyl)-3-phenylpropan-1-ol (3aq)

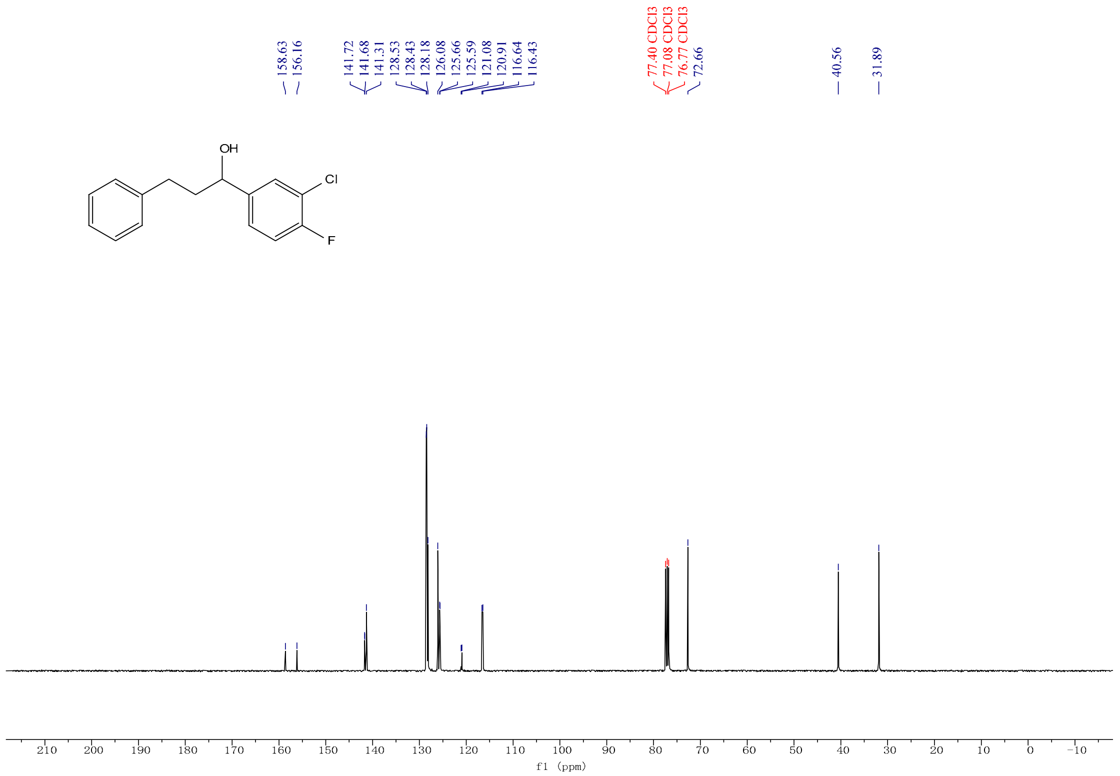

Figure S34: ^{13}CNMR spectrum of 1-(3-chloro-4-fluorophenyl)-3-phenylpropan-1-ol (3aq)

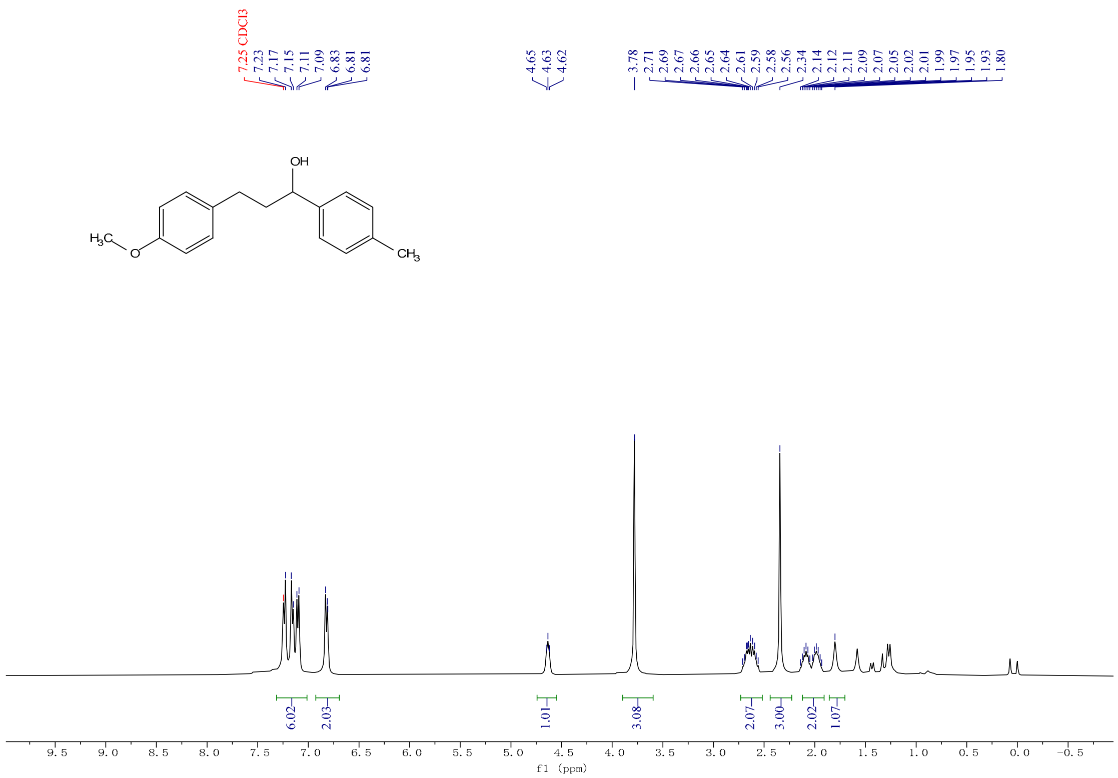

Figure S35: ^{1}H NMR spectrum of 3-(4-methoxyphenyl)-1-(p-tolyl)propan-1-ol (3ba)

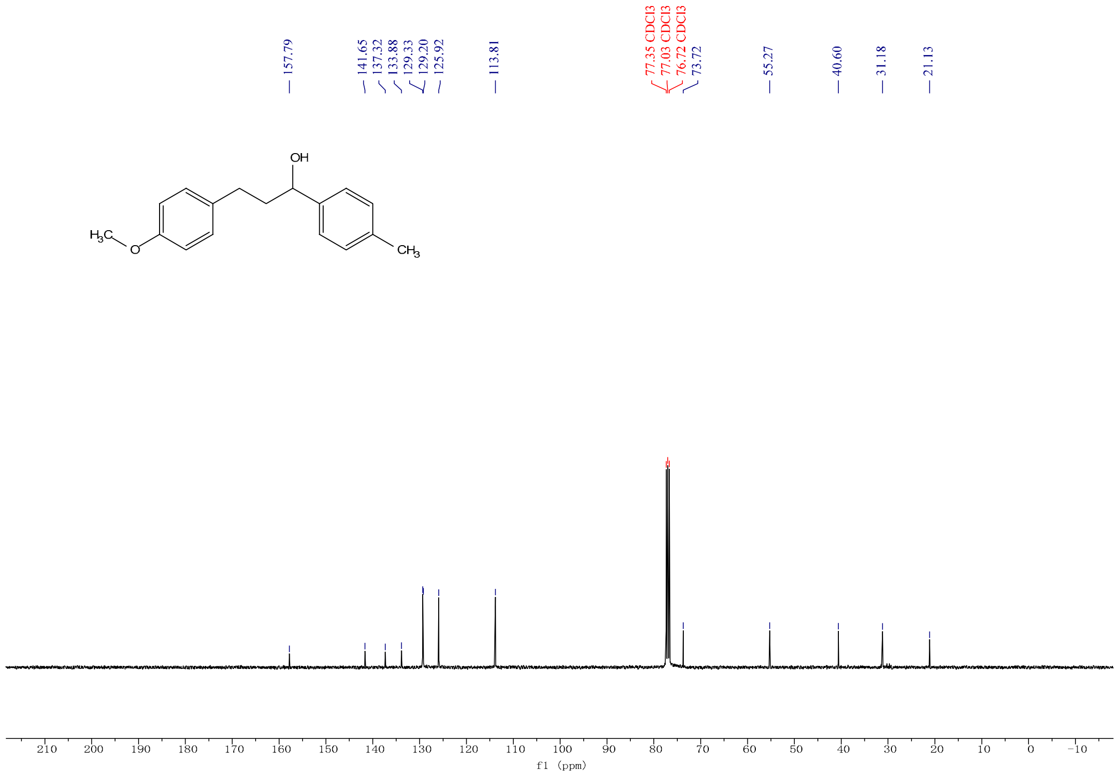

Figure S36: ^{13}CNMR spectrum of 3-(4-methoxyphenyl)-1-(p-tolyl)propan-1-ol (3ba)

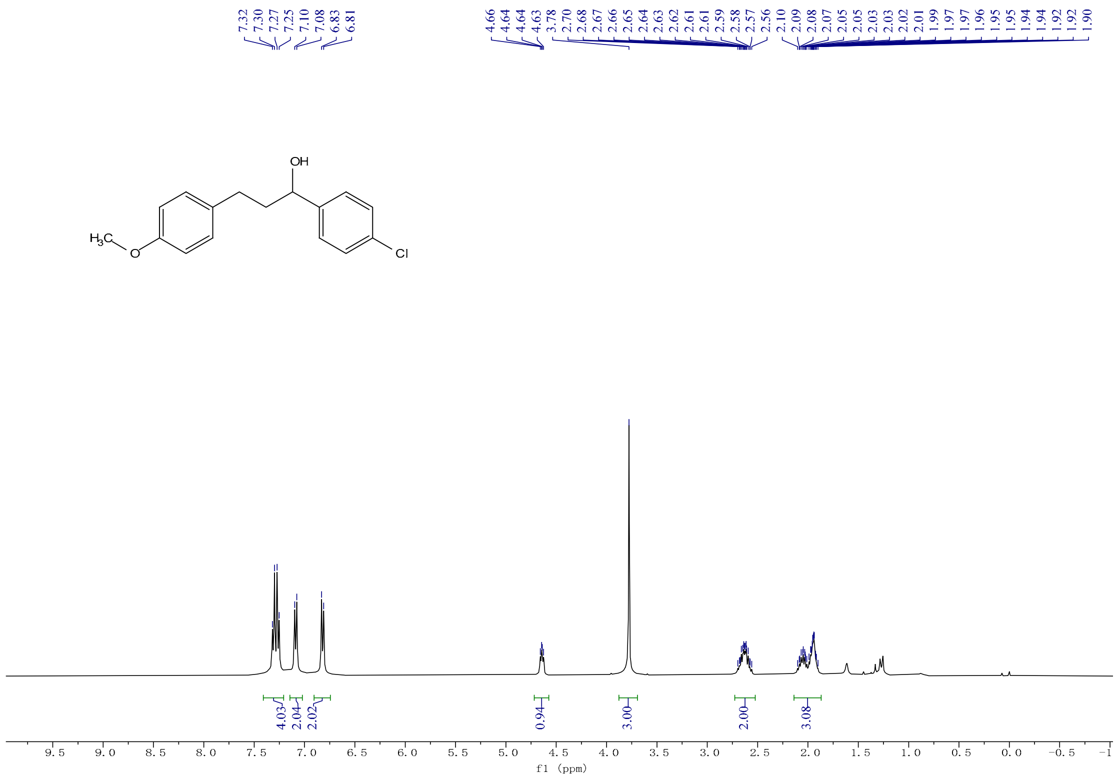

Figure S37: ^{1}H NMR spectrum of 1-(4-chlorophenyl)-3-(4-methoxyphenyl)propan-1-ol (3be)

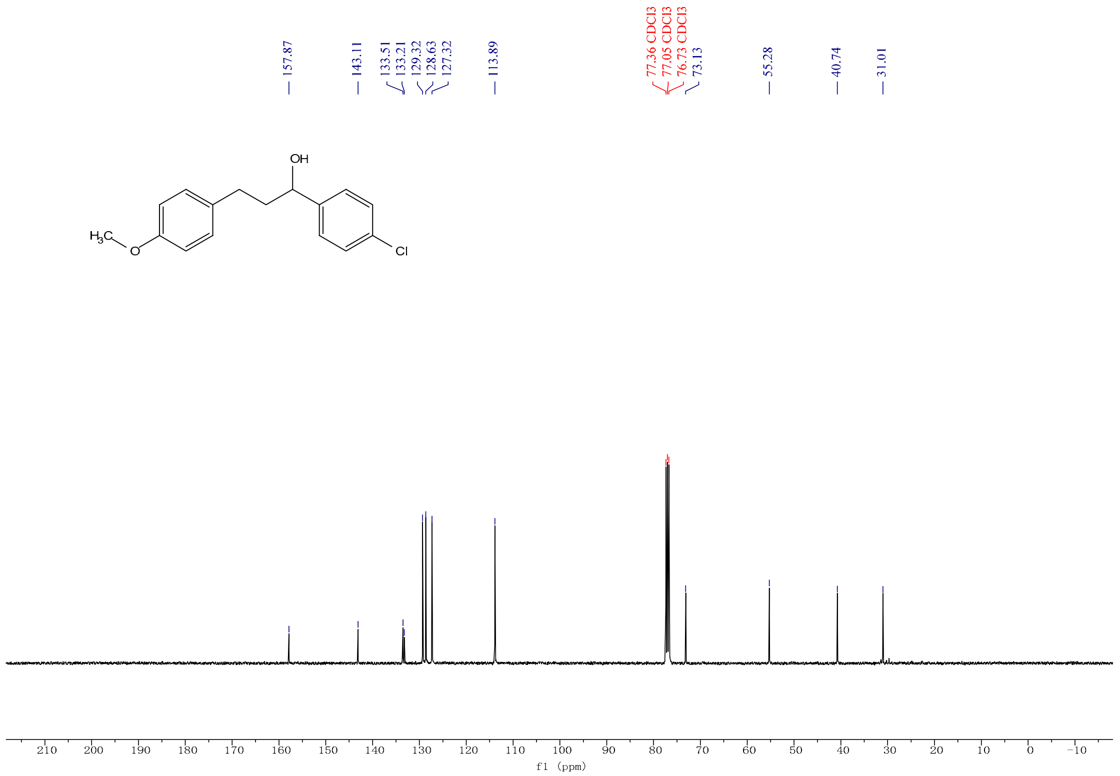

Figure S38:^{13}CNMR spectrum of 1-(4-chlorophenyl)-3-(4-methoxyphenyl)propan-1-ol (3be)

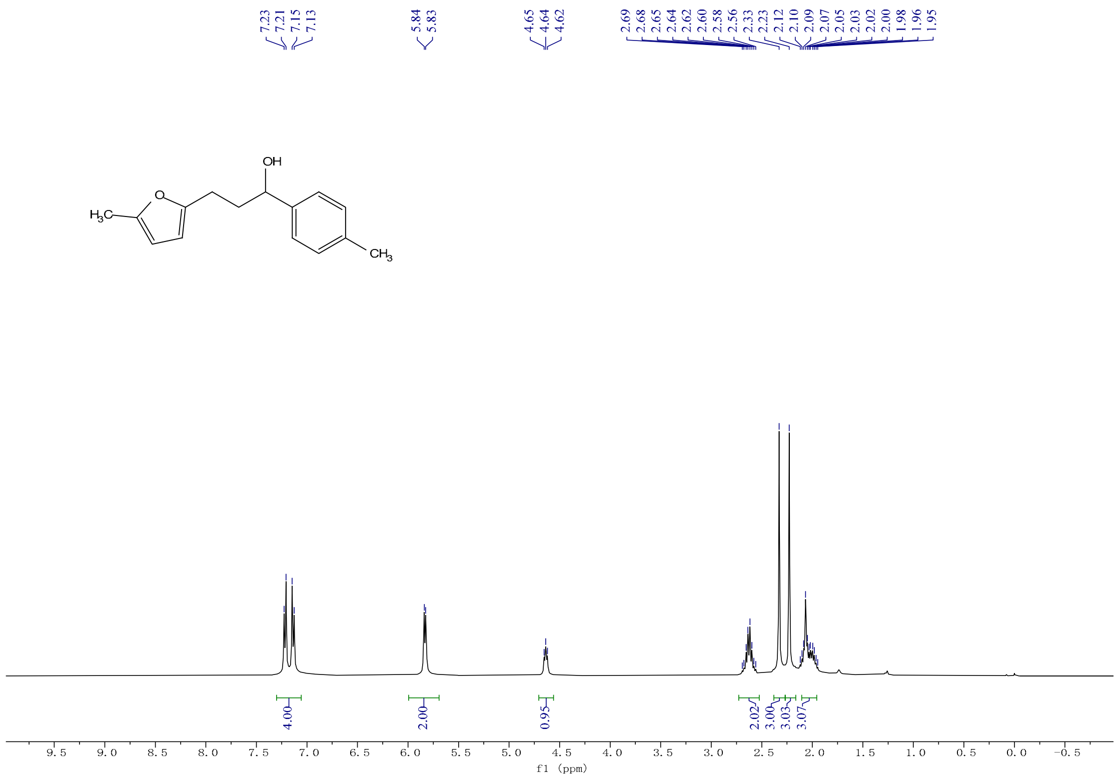

Figure S39: ^{1}H NMR spectrum of 3-(5-methylfuran-2-yl)-1-(p-tolyl)propan-1-ol (3ca)

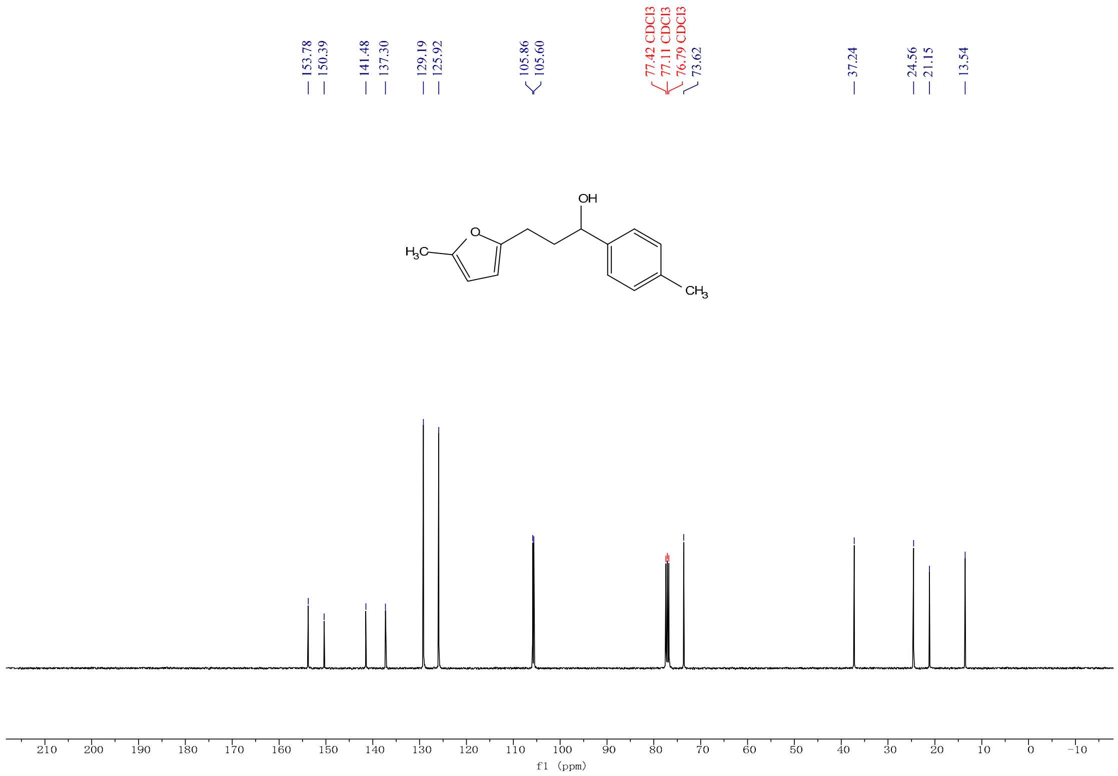

Figure S40: ^{13}CNMR spectrum of 3-(5-methylfuran-2-yl)-1-(p-tolyl)propan-1-ol (3ca)

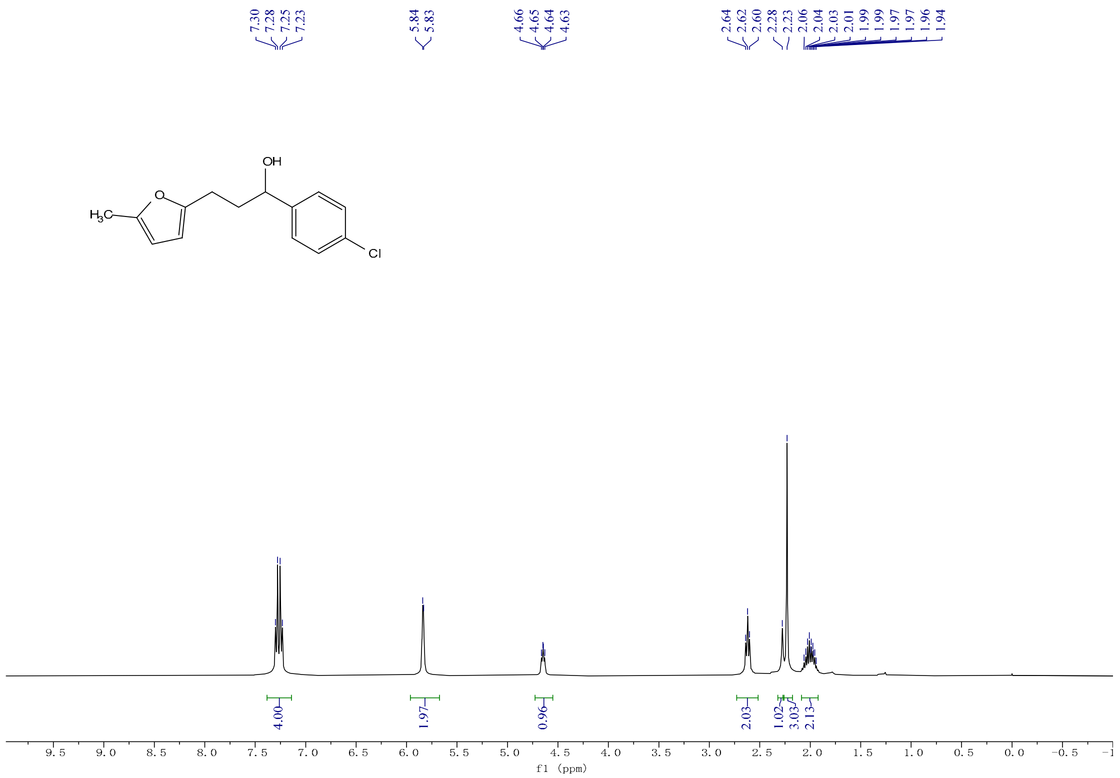

Figure S41: ^{1}H NMR spectrum of 1-(4-chlorophenyl)-3-(5-methylfuran-2-yl)propan-1-ol (3ce)

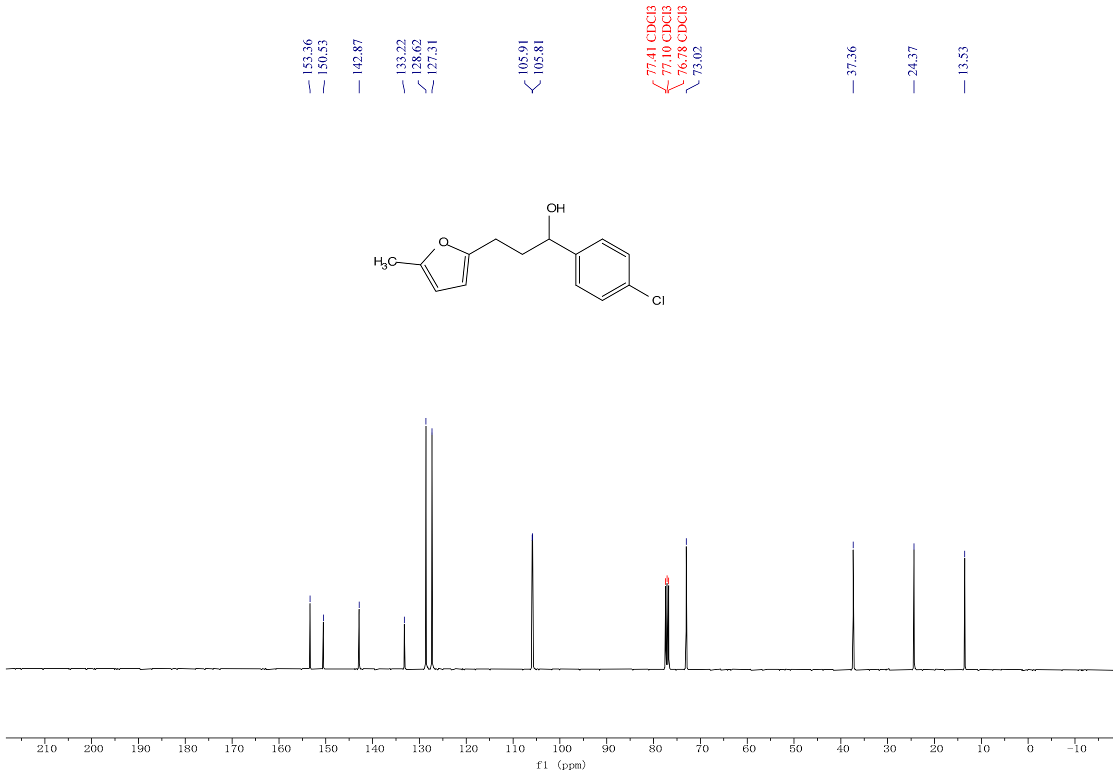

Figure S42: ^{13}CNMR spectrum of 1-(4-chlorophenyl)-3-(5-methylfuran-2-yl)propan-1-ol (3ce)

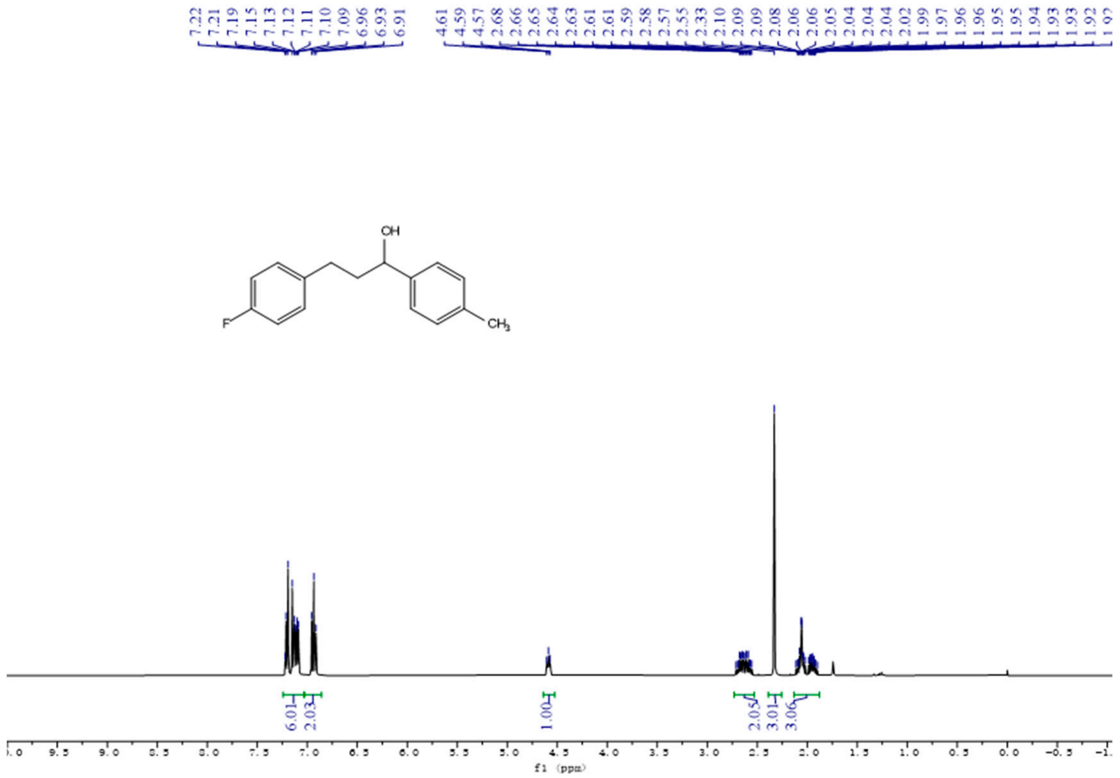

Figure S43: ^{1}H NMR spectrum of 3-(4-fluorophenyl)-1-(*p*-tolyl)propan-1-ol (3ja)

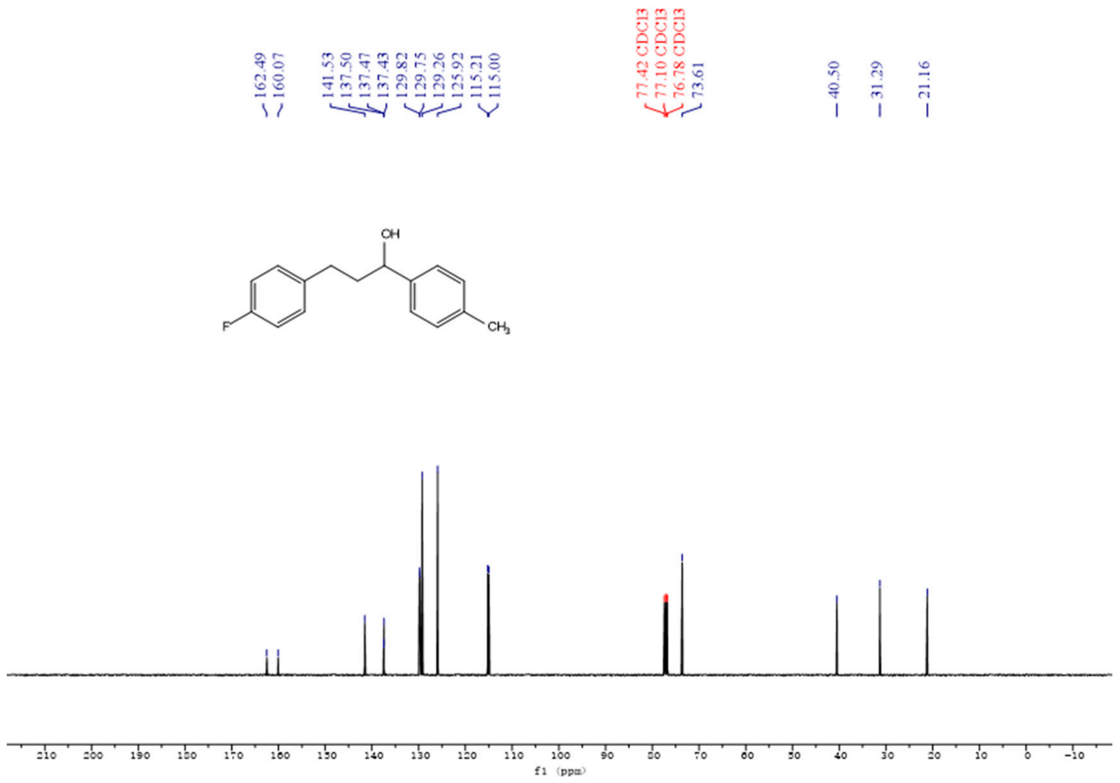

Figure S44: ^{13}C NMR spectrum of 3-(4-fluorophenyl)-1-(*p*-tolyl)propan-1-ol (3ja)

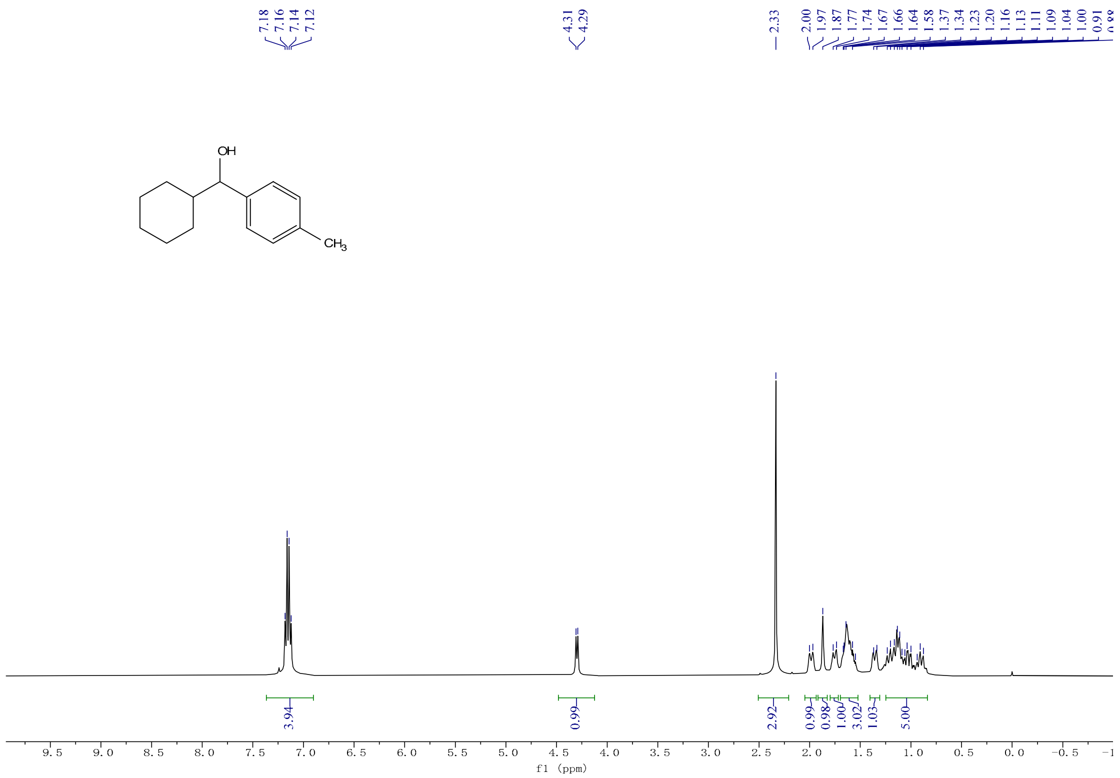

Figure S45: ^{1}H NMR spectrum of cyclohexyl(p-tolyl)methanol (3da)

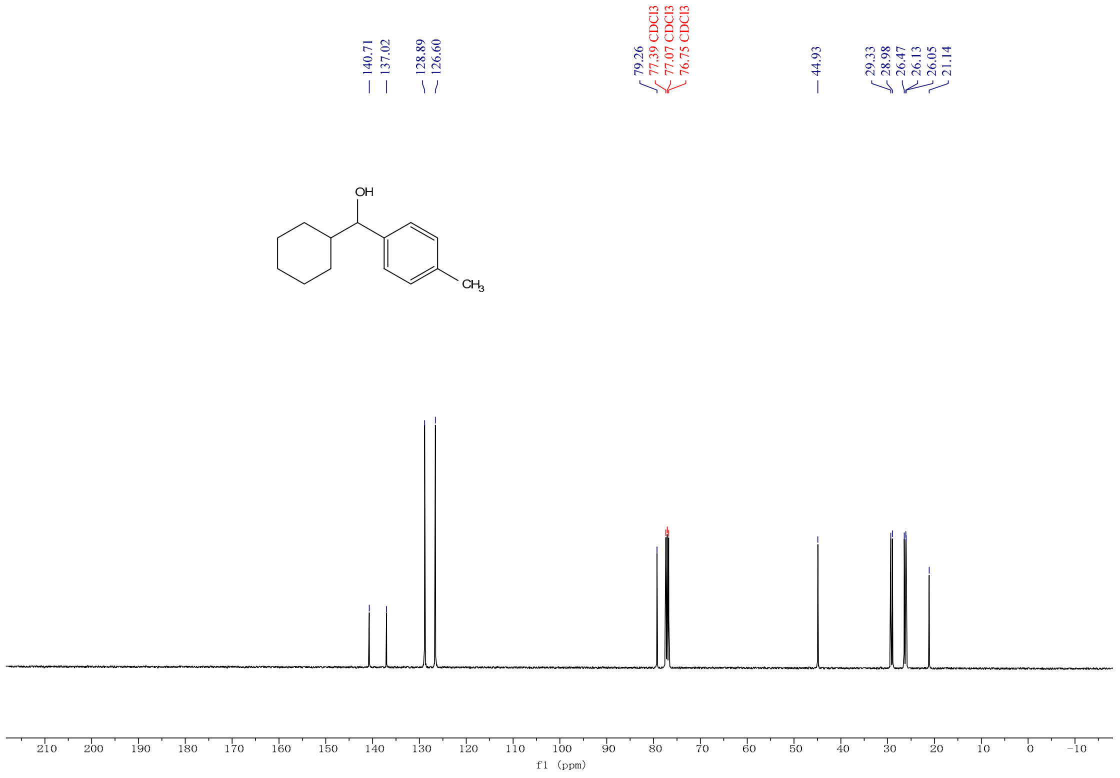

Figure S46: ^{13}CNMR spectrum of cyclohexyl(p-tolyl)methanol (3da)

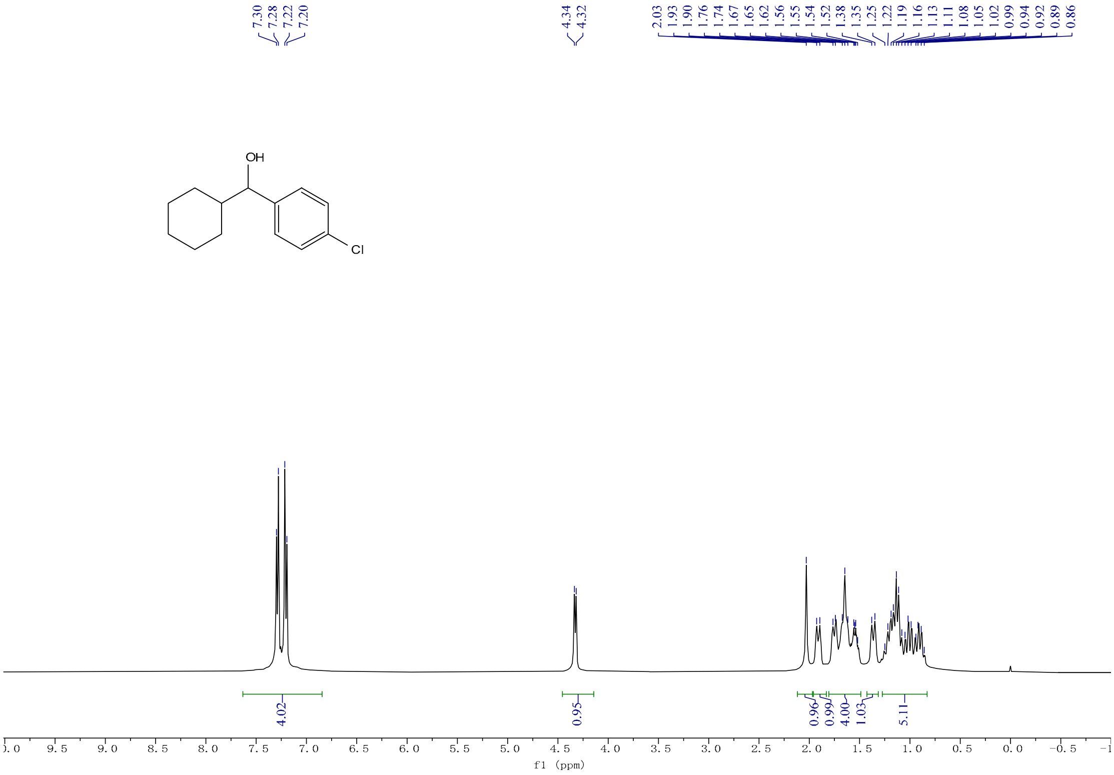

Figure S47: ^{1}H NMR spectrum of (4-chlorophenyl)(cyclohexyl)methanol (3de)

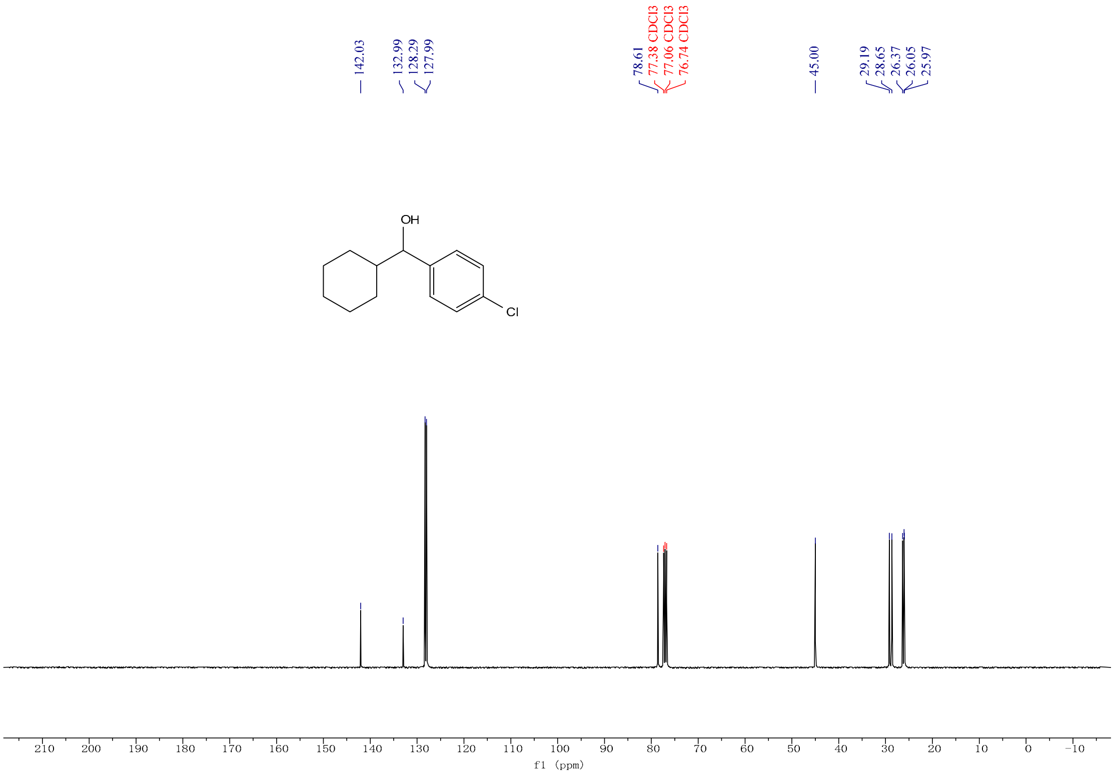

Figure S48: ^{13}CNMR spectrum of (4-chlorophenyl)(cyclohexyl)methanol (3de)

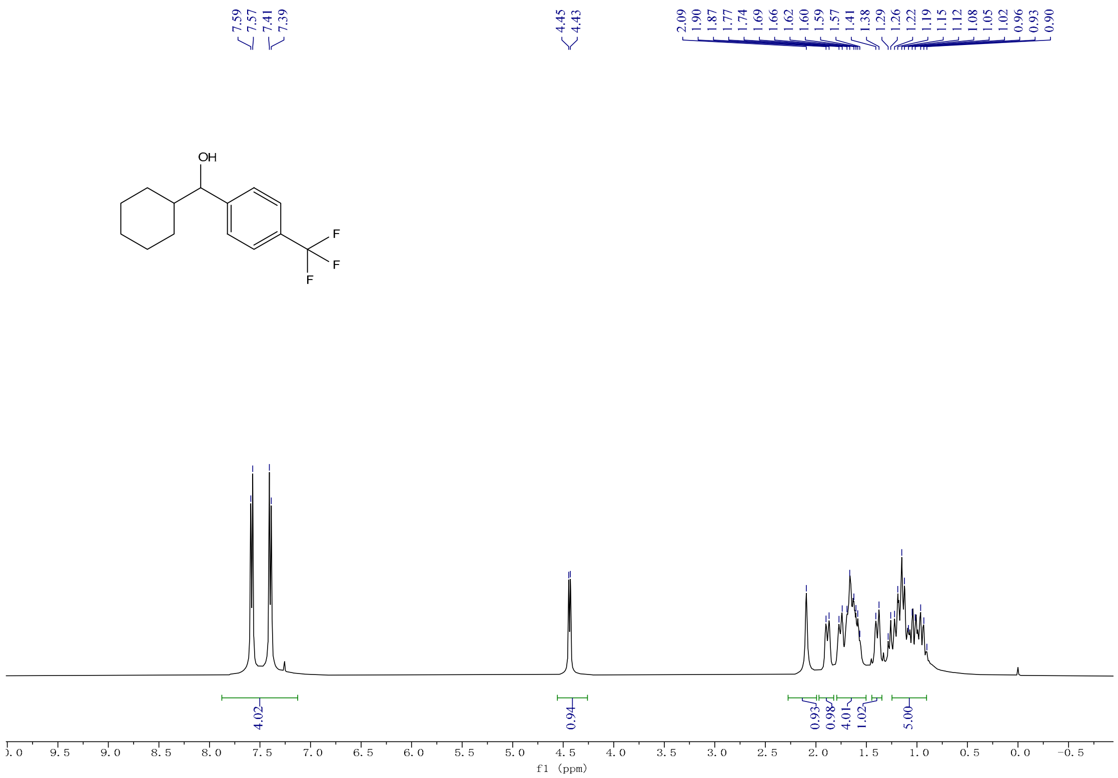

Figure S49: ^{1}H NMR spectrum of cyclohexyl(4-(trifluoromethyl)phenyl)methanol (3dh)

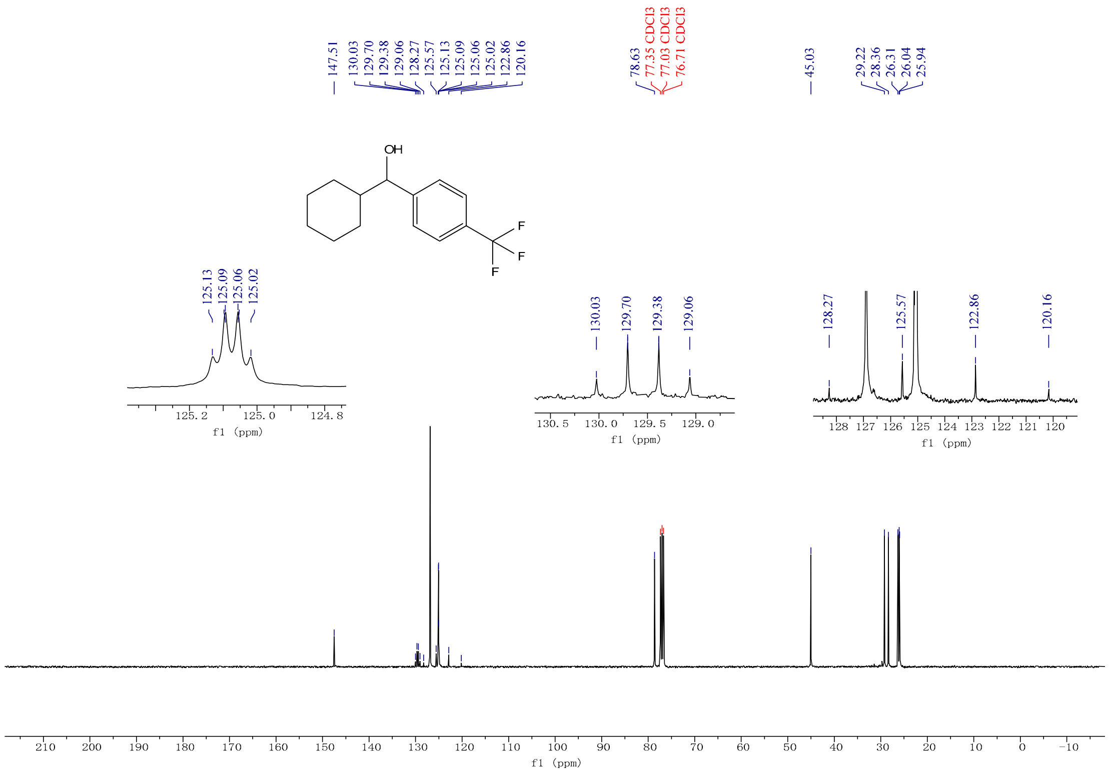

Figure S50: ^{13}CNMR spectrum of cyclohexyl(4-(trifluoromethyl)phenyl)methanol (3dh)

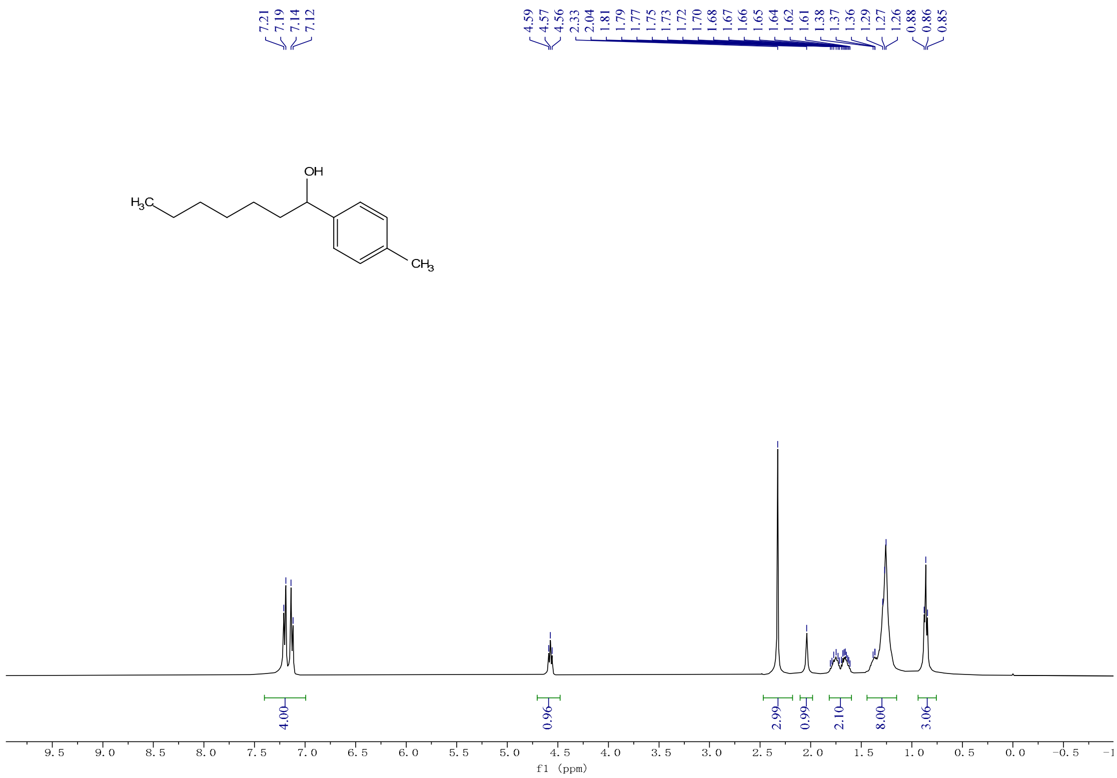

Figure S51: ^{1}H NMR spectrum of 1-(p-tolyl)heptan-1-ol (3ea)

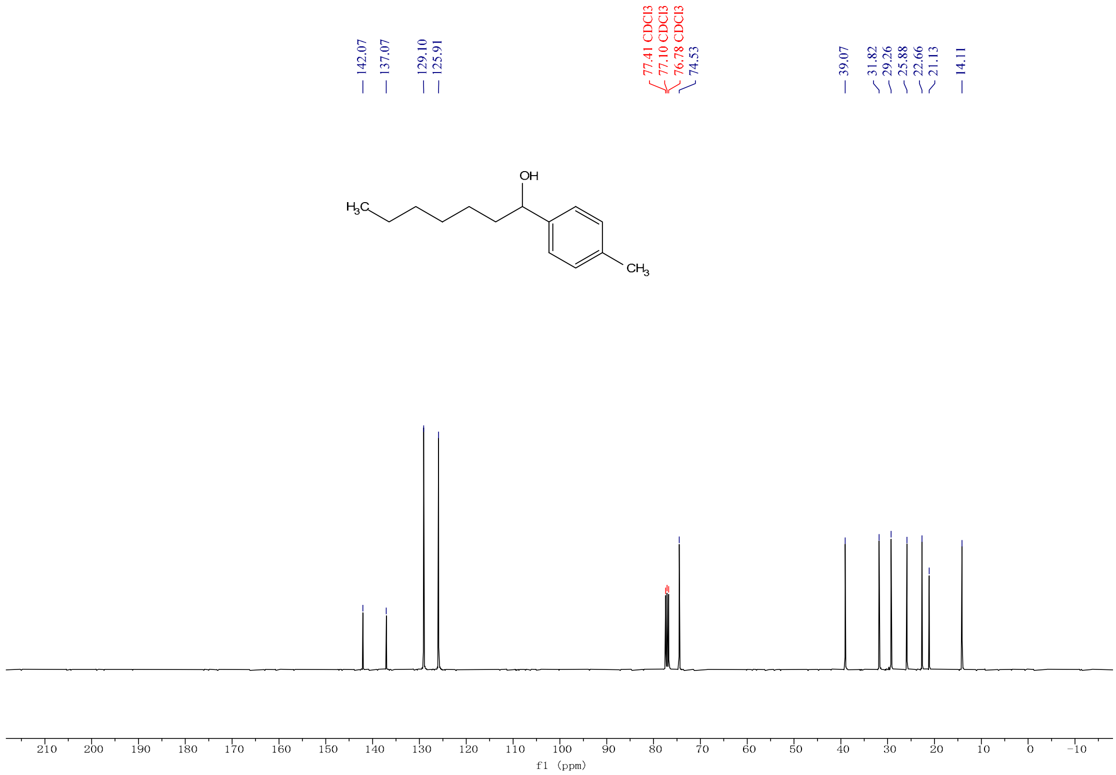

Figure S52: ^{13}CNMR spectrum of 1-(p-tolyl)heptan-1-ol (3ea)

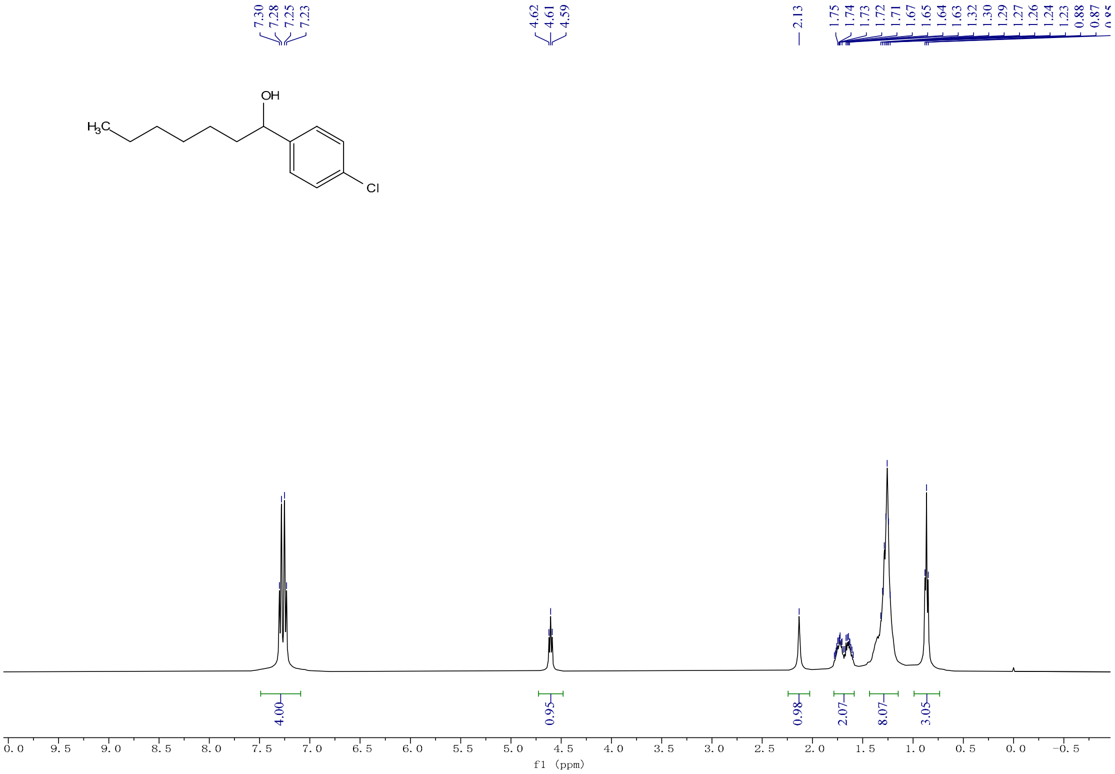

Figure S53: ^{1}H NMR spectrum of 1-(4-chlorophenyl)heptan-1-ol (3ee)

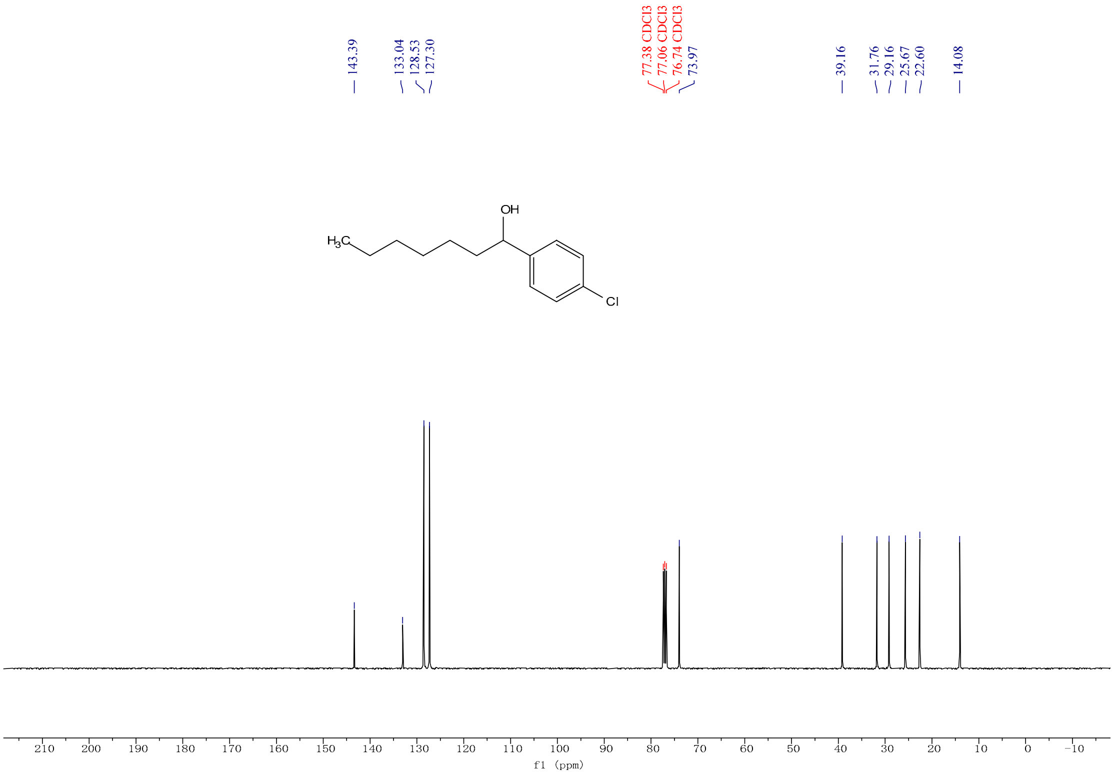

Figure S54: ^{13}CNMR spectrum of 1-(4-chlorophenyl)heptan-1-ol (3ee)

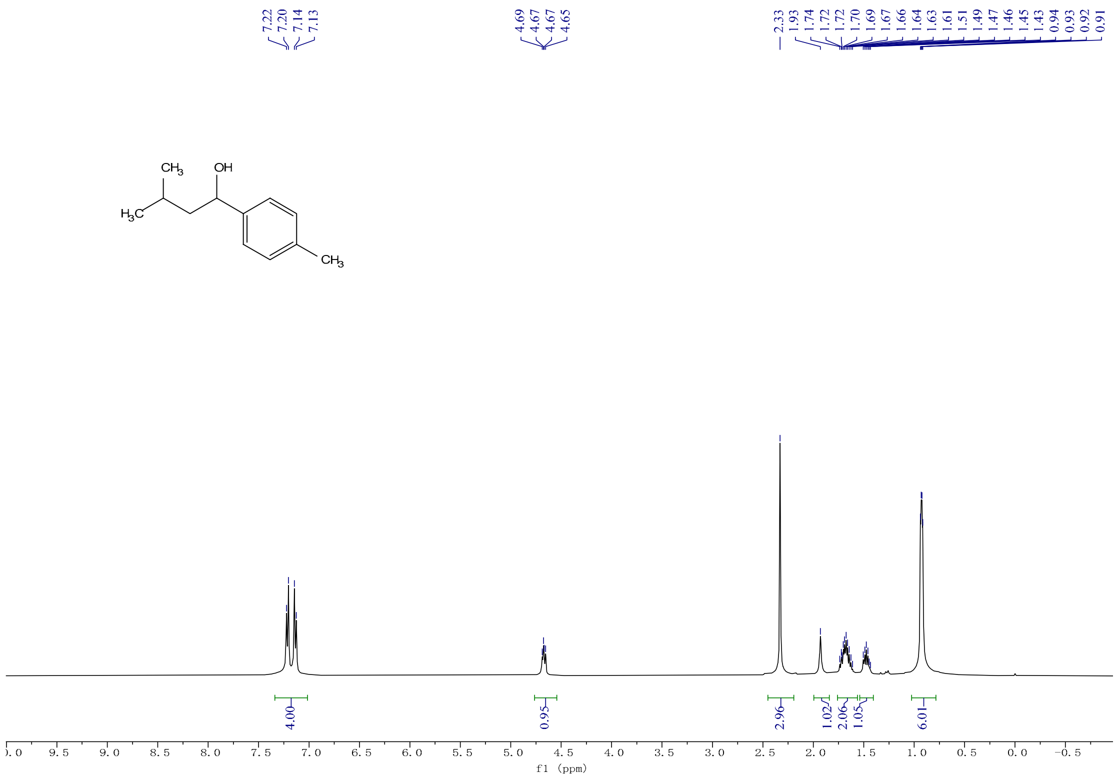

Figure S55:^{1}H NMR spectrum of 3-methyl-1-(p-tolyl)butan-1-ol (3fa)

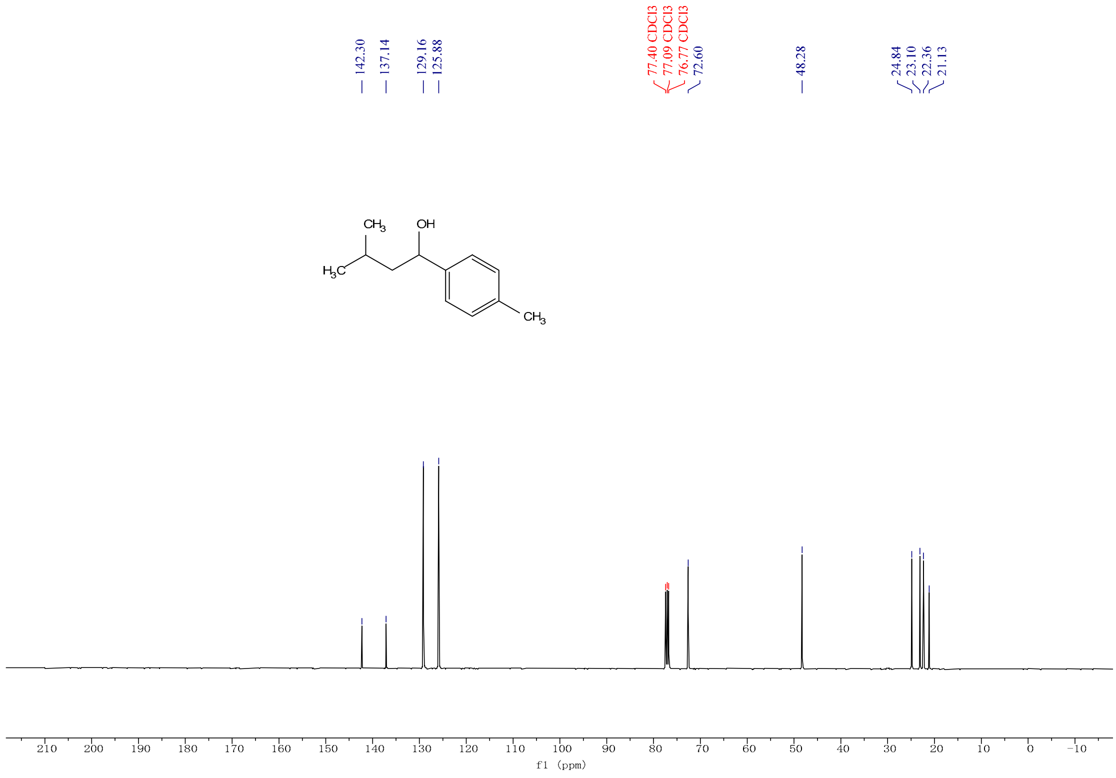

Figure S56: ^{13}CNMR spectrum of 3-methyl-1-(p-tolyl)butan-1-ol (3fa)

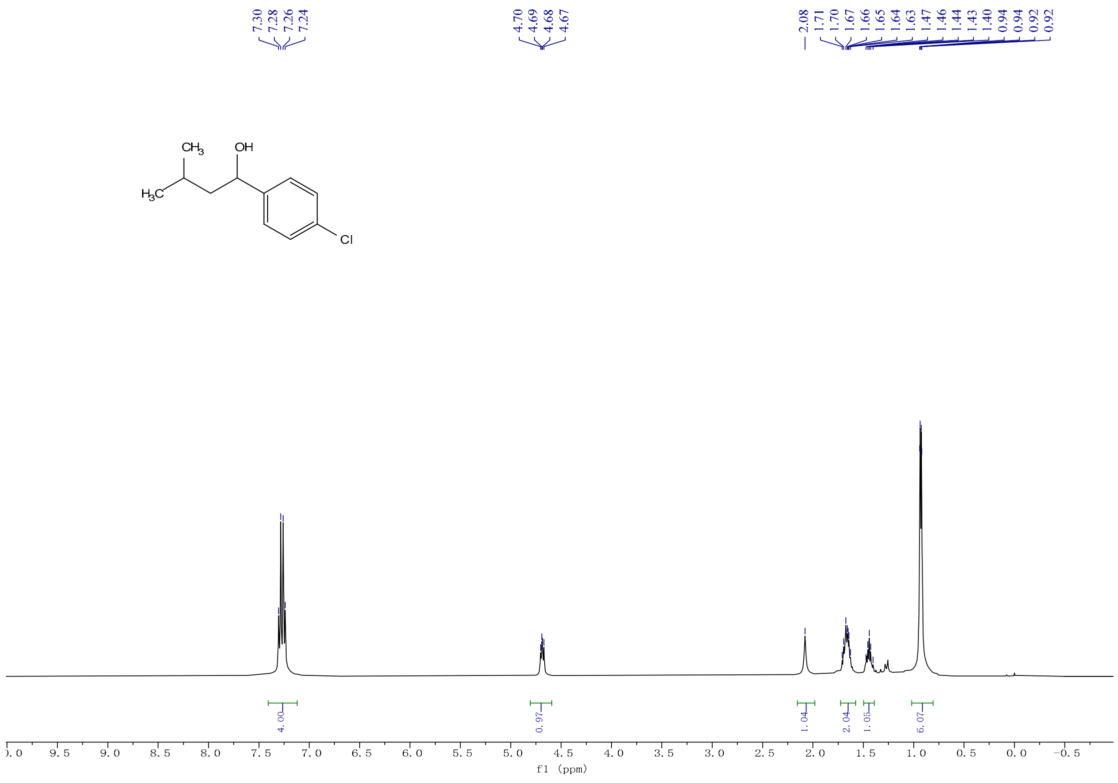

Figure S57: ^{1}H NMR spectrum of 1-(4-chlorophenyl)-3-methylbutan-1-ol (3fe)

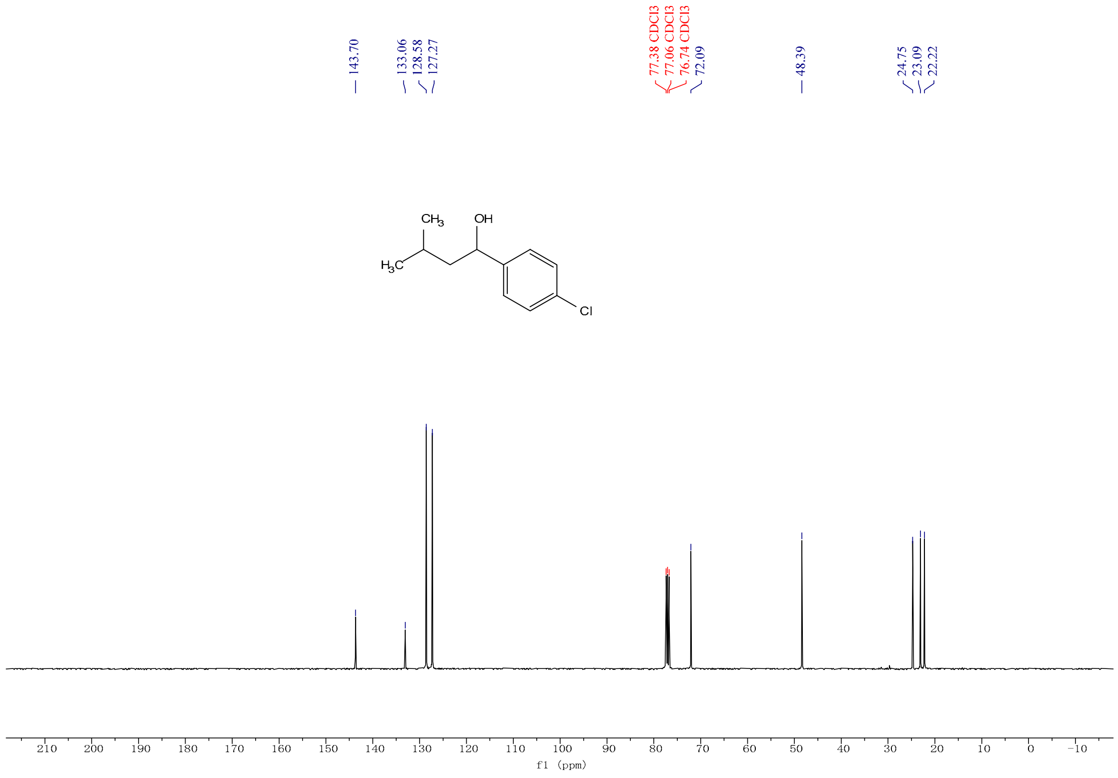

Figure S58: ^{13}CNMR spectrum of 1-(4-chlorophenyl)-3-methylbutan-1-ol (3fe)

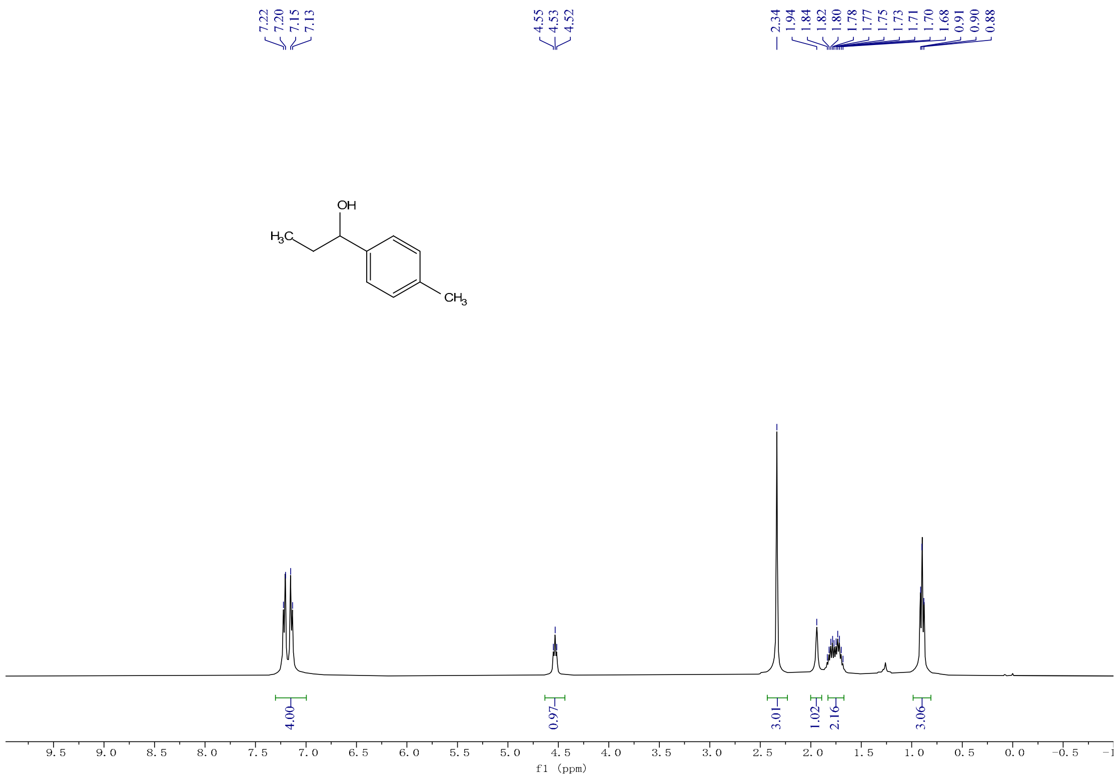

Figure S59: ^{1}H NMR spectrum of 1-(p-tolyl)propan-1-ol (3ga)

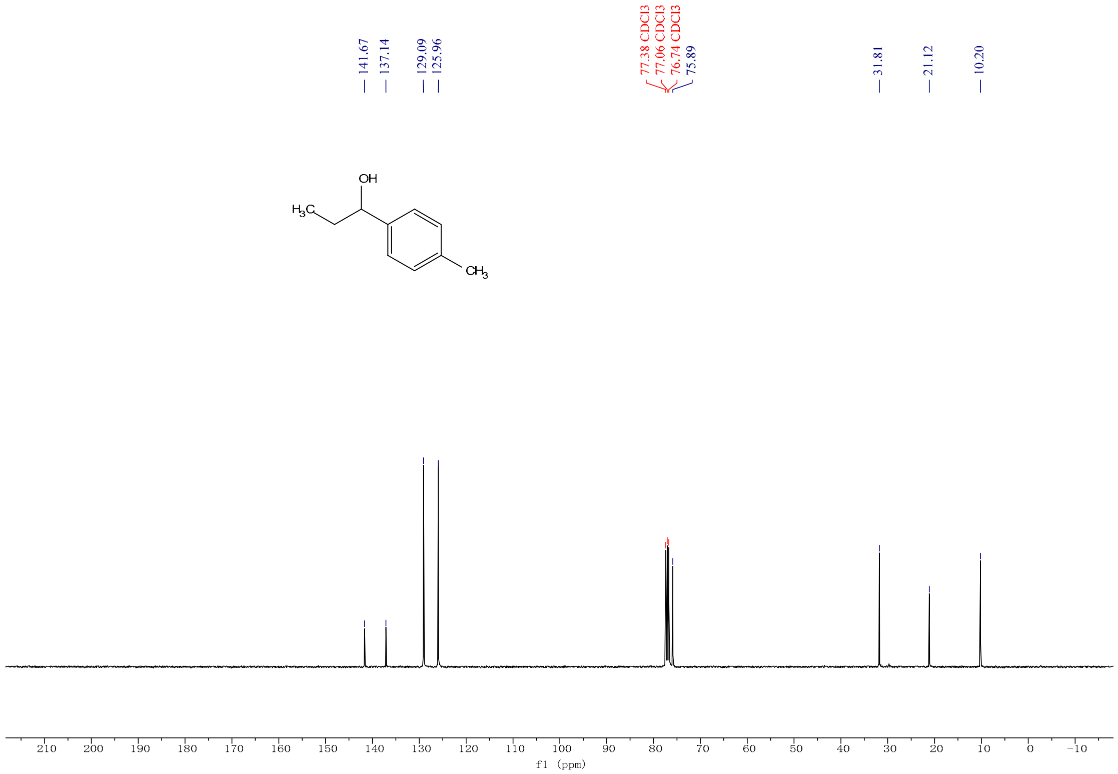

Figure S60: ^{13}CNMR spectrum of 1-(p-tolyl)propan-1-ol (3ga)

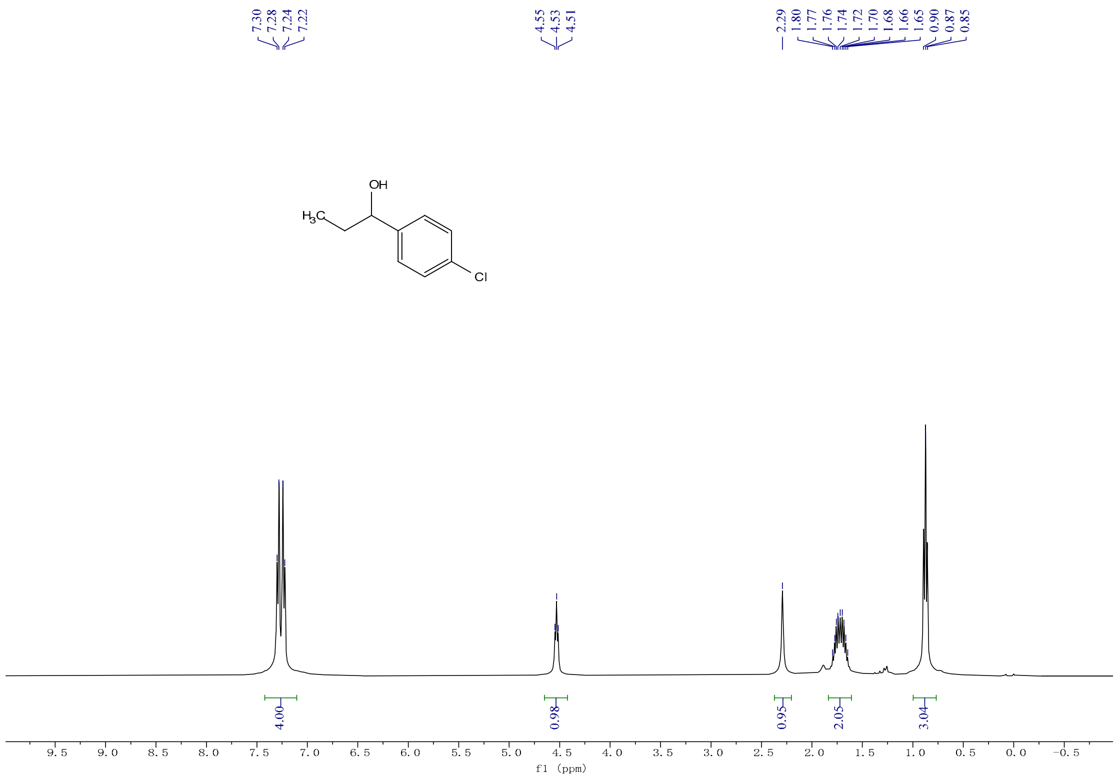

Figure S61: ^{1}H NMR spectrum of 1-(4-chlorophenyl)propan-1-ol (3ge)

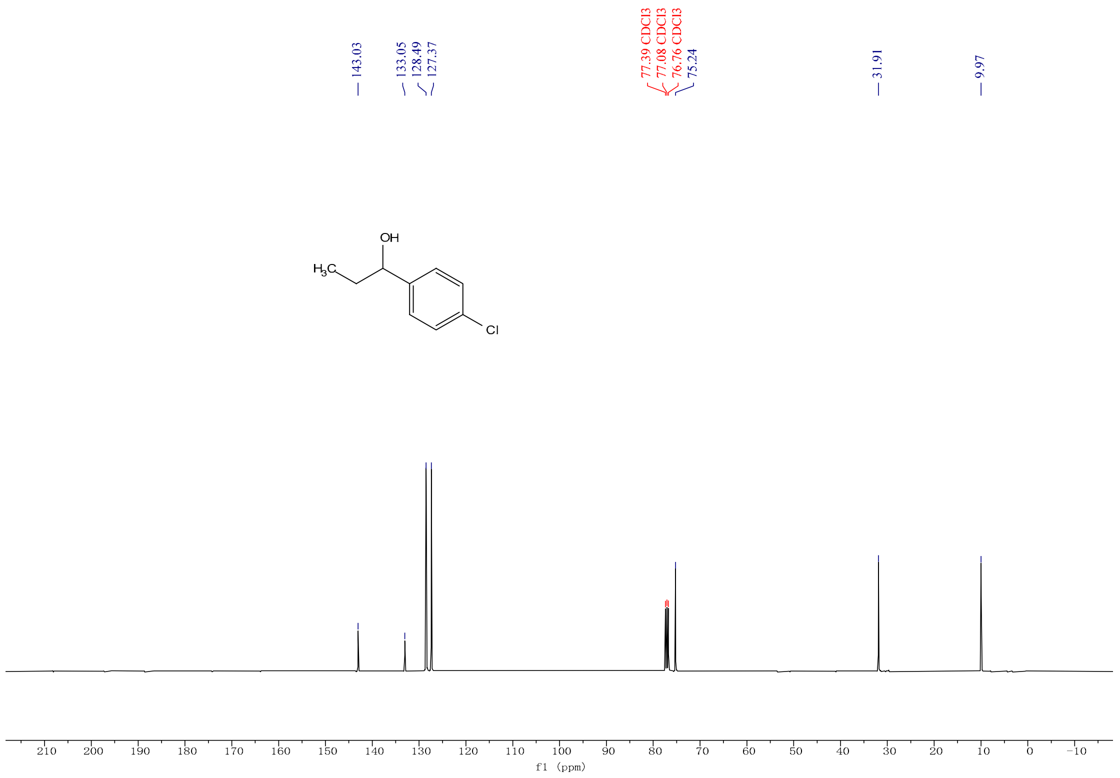

Figure S62: ^{13}CNMR spectrum of 1-(4-chlorophenyl)propan-1-ol (3ge)

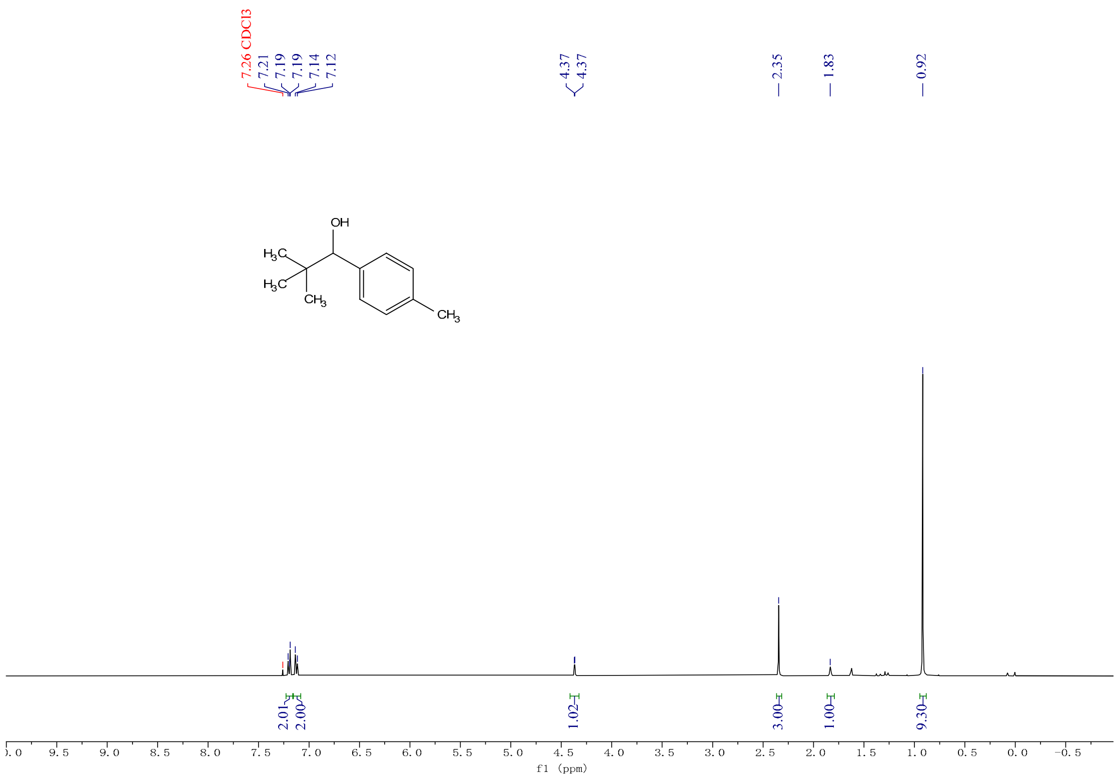

Figure S63: ^{1}H NMR spectrum of 2,2-dimethyl-1-(p-tolyl)propan-1-ol (3ha)

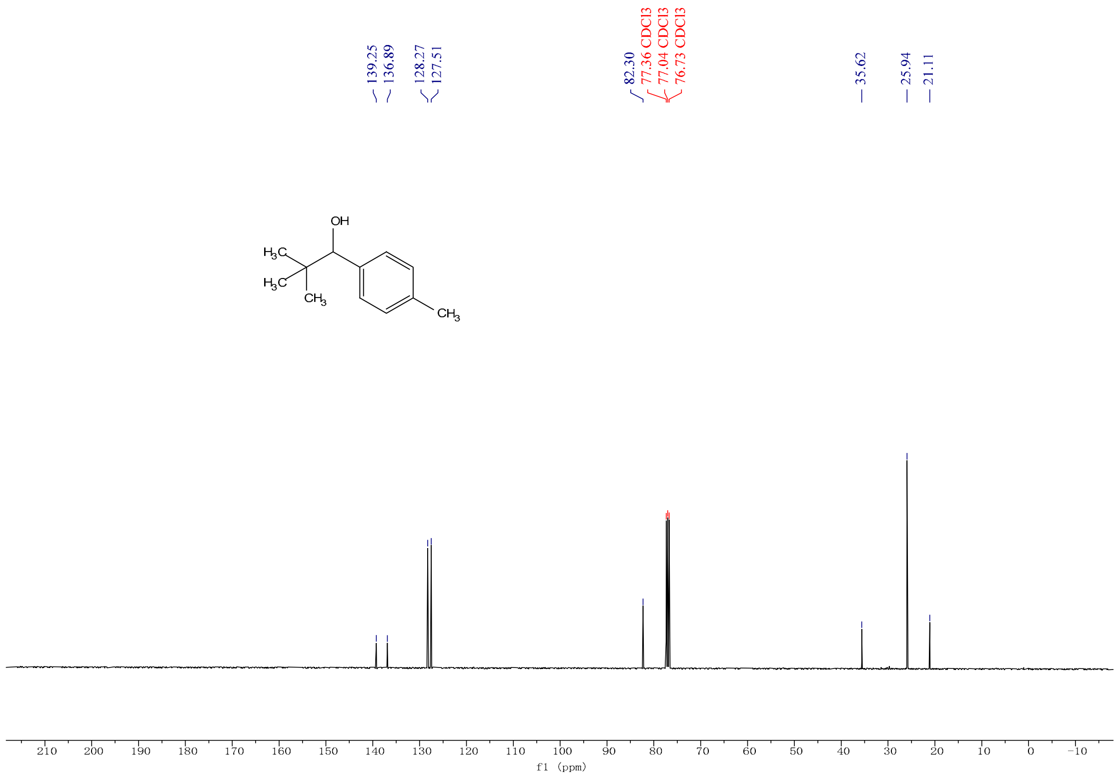

Figure S64: ^{13}CNMR spectrum of 2,2-dimethyl-1-(p-tolyl)propan-1-ol (3ha)

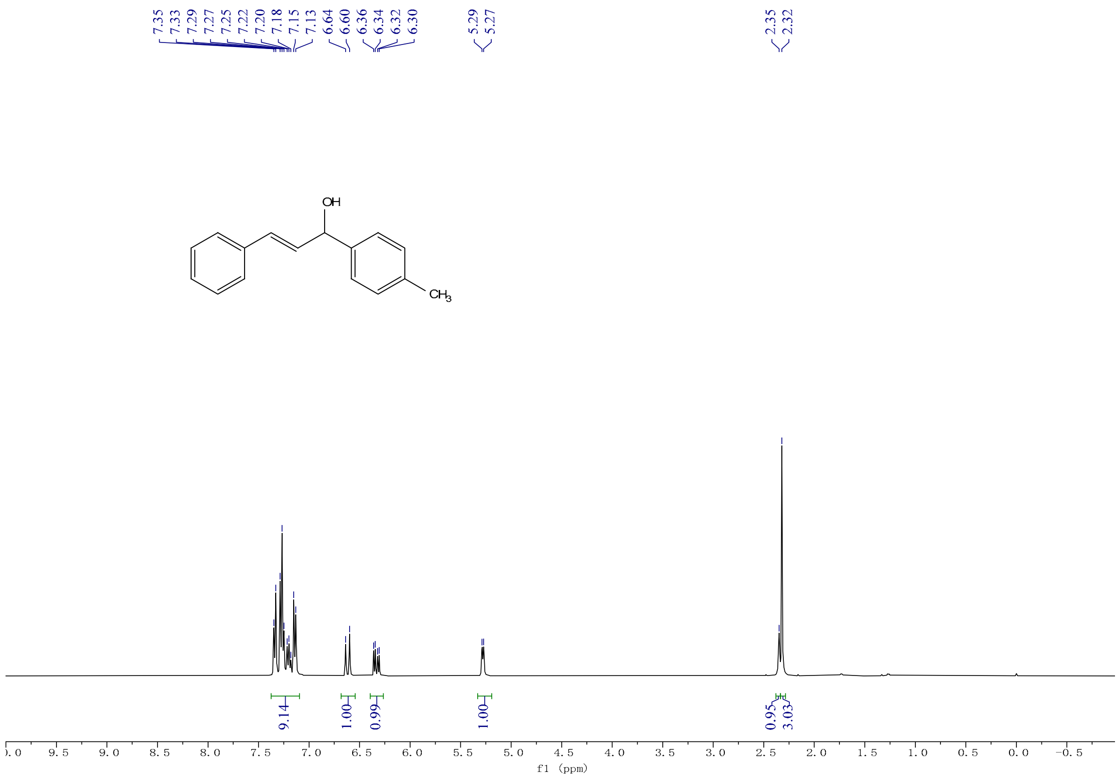

Figure S65: ^{1}H NMR spectrum of (*E*)-3-phenyl-1-(p-tolyl)prop-2-en-1-ol (3ia)

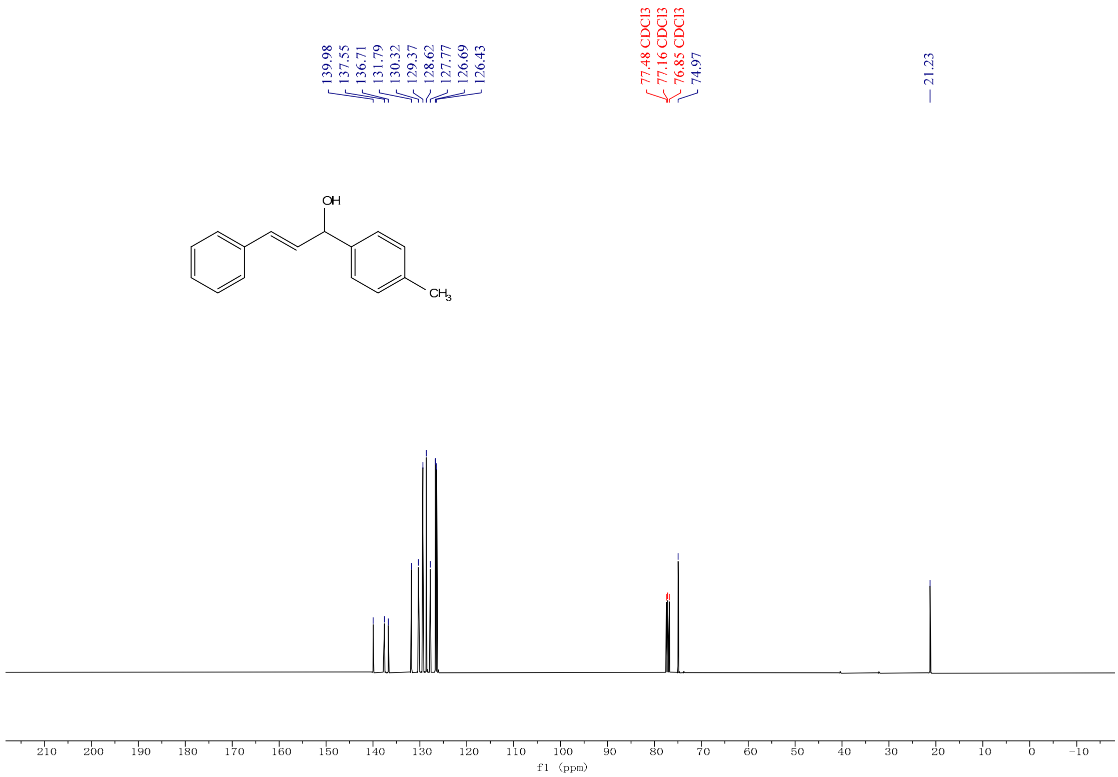

Figure S66: ^{13}CNMR spectrum of (*E*)-3-phenyl-1-(p-tolyl)prop-2-en-1-ol (3ia)

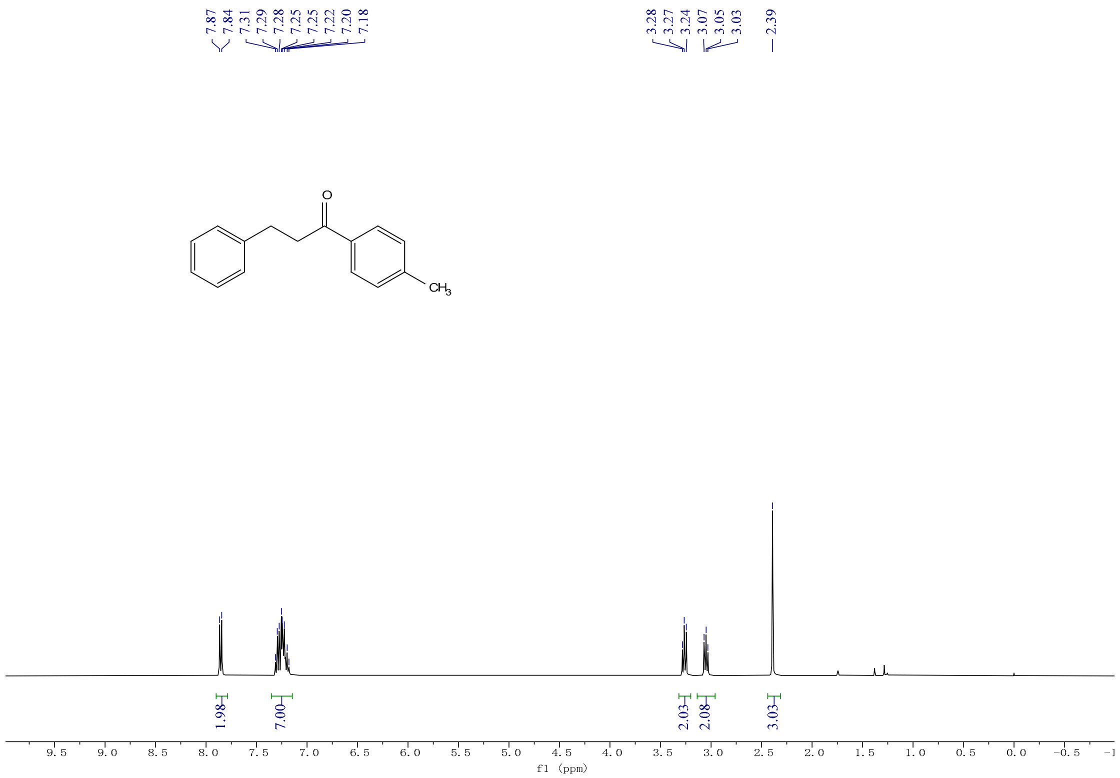

Figure S67: ^{1}H NMR spectrum of 4'-Methyl-3-phenylpropiophenone(4aa)

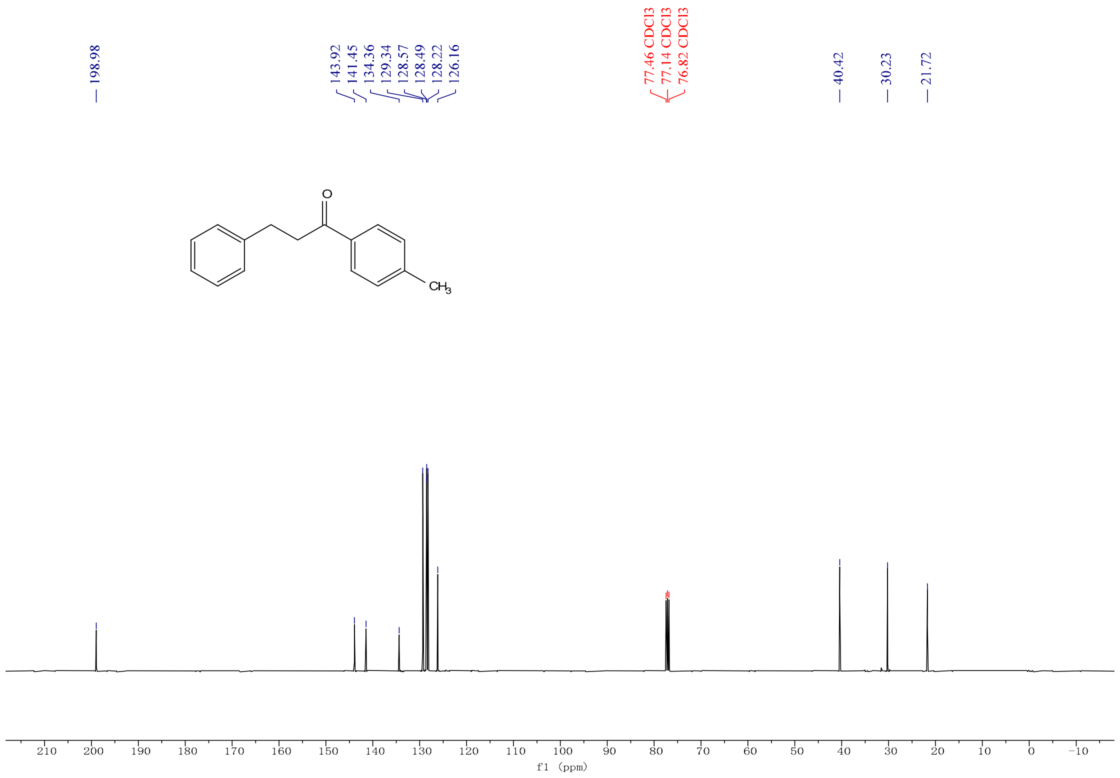

Figure S68: ^{13}C NMR spectrum of 4'-Methyl-3-phenylpropiophenone(4aa)

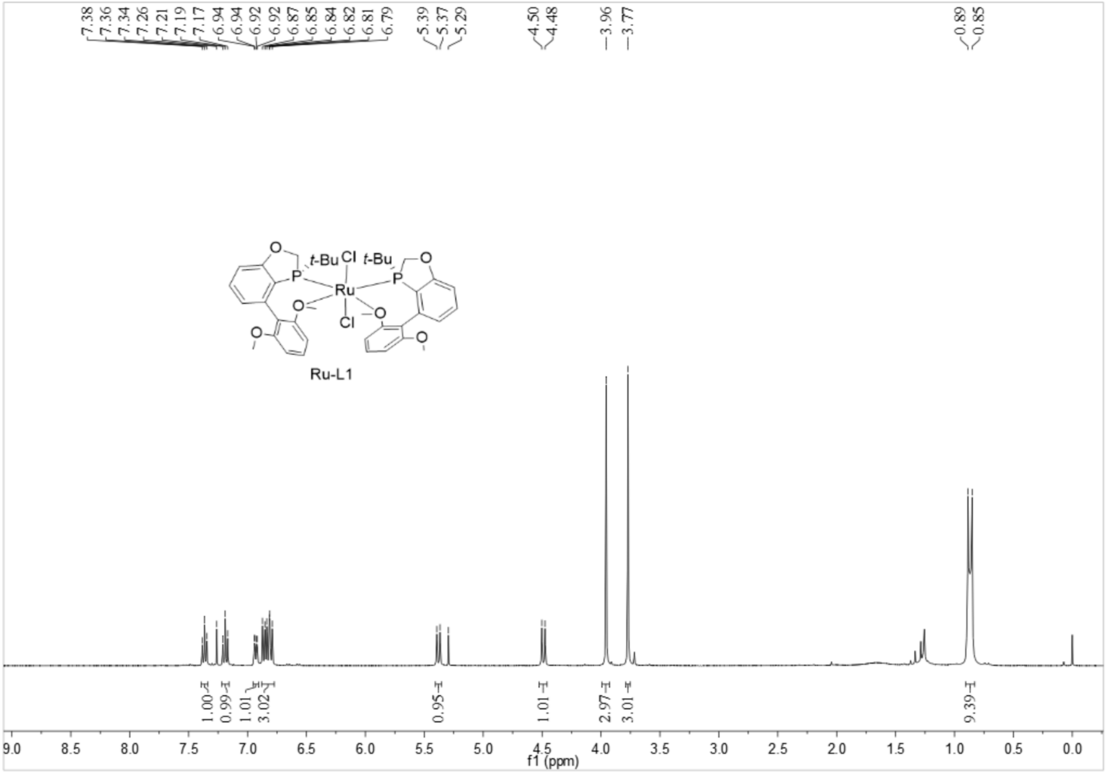

Figure S69: ^{1}H NMR spectrum of Ru-L1

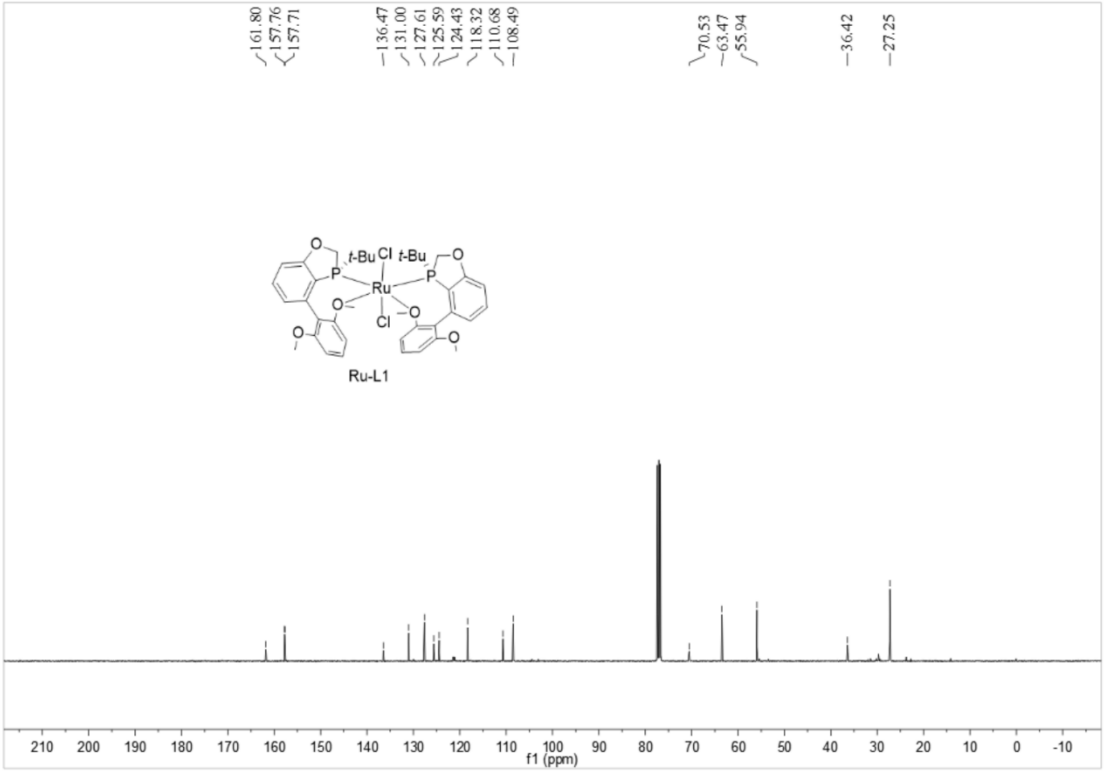

Figure S70: ^{13}C NMR spectrum of Ru-L1

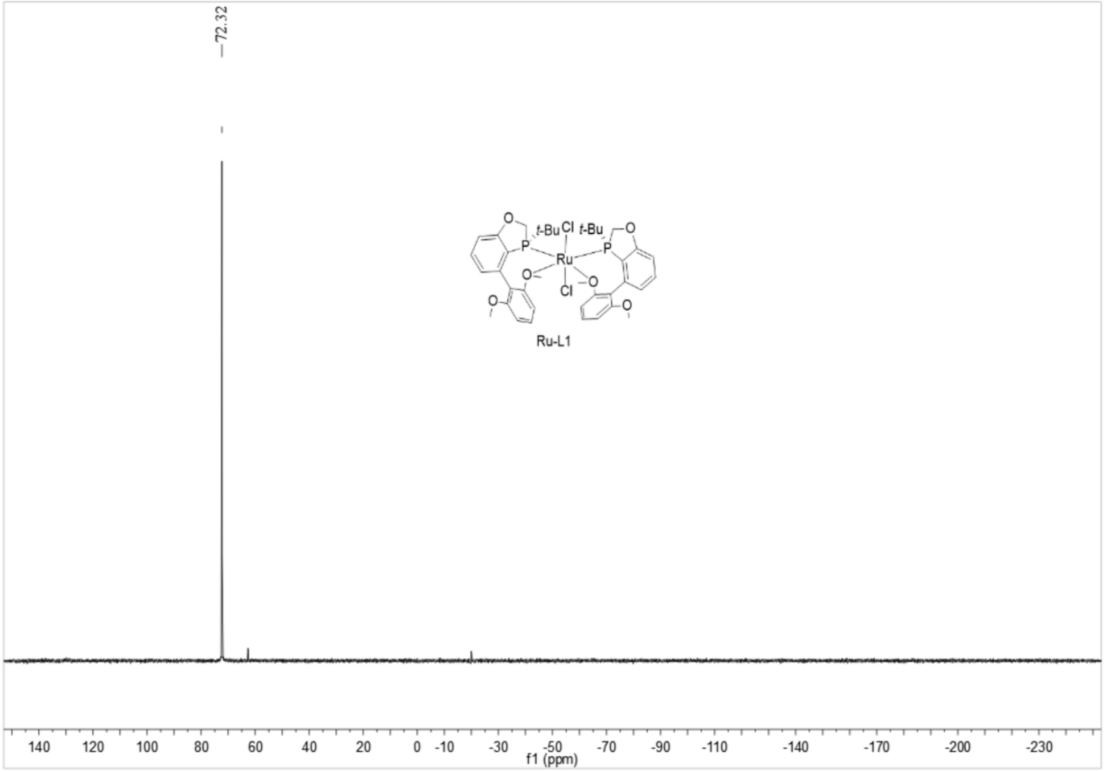

Figure S71: ^{31}P NMR spectrum of Ru-L1

H. HPLC Spectra

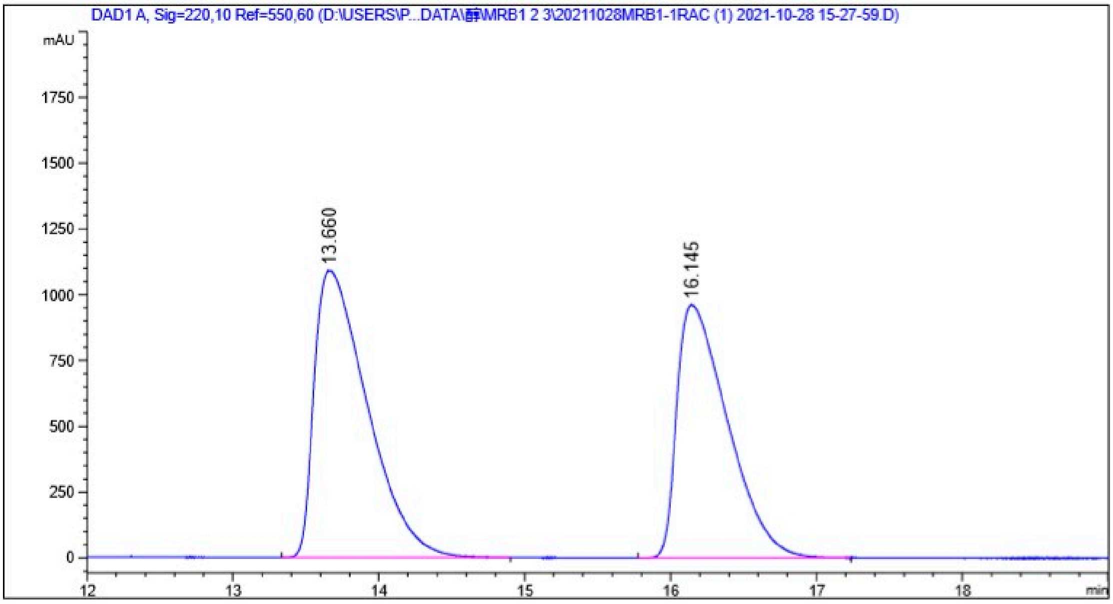

#	Time	Area	height	Width	Symmetry	Area%	Type
1	13.66	27506.2	1090.3	0.2963	0.378	54.233	BV R
2	16.145	23212.1	961.6	0.2916	0.403	45.767	BV R

Figure S72: Racemic product of 3-phenyl-1-(p-tolyl)propan-1-ol (3aa)

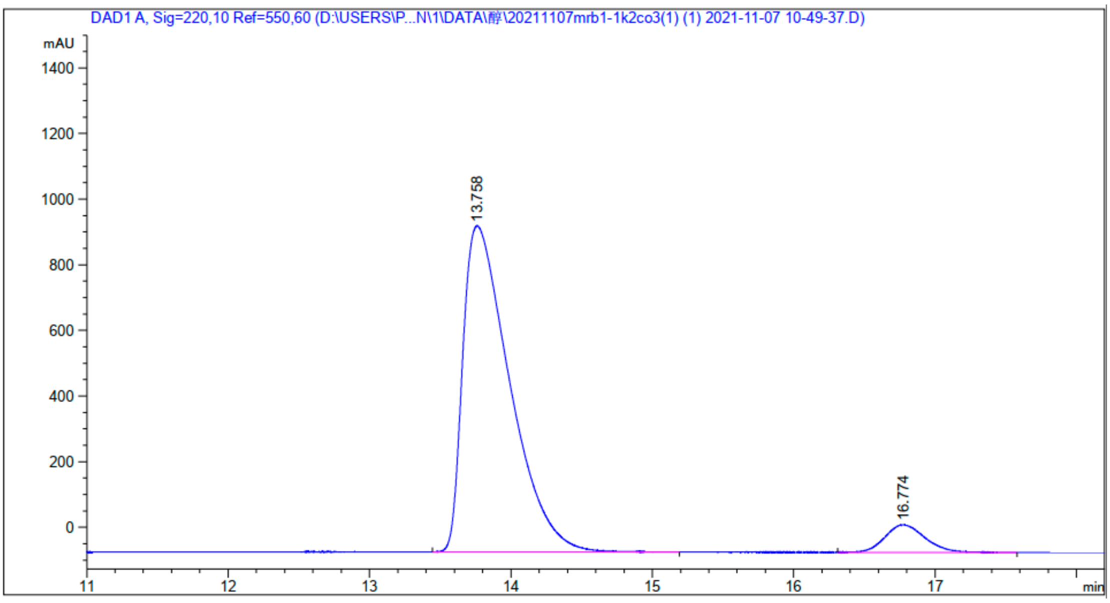

#	Time	Area	height	Width	Symmetry	Area%	Type
1	13.758	22869.5	993.8	0.301	0.387	93.250	BV R
2	16.774	1655.5	83.7	0.2353	0.811	6.750	VV R

Figure S73: Chiral product of 3-phenyl-1-(p-tolyl)propan-1-ol (3aa)

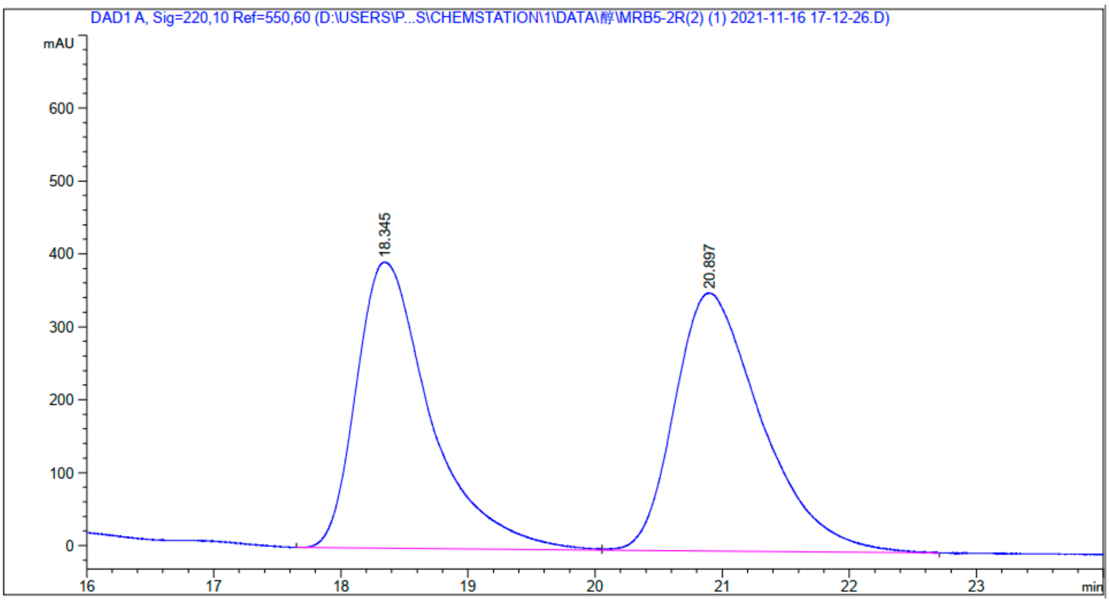

#	Time	Area	height	Width	Symmetry	Area%	Type
1	18.345	15876.2	392.6	0.4743	0.602	48.636	VV R
2	20.897	16766.4	353.8	0.5539	0.641	51.364	VV R

Figure S74: Racemic product of 1-(4-methoxyphenyl)-3-phenylpropan-1-ol (3ab)

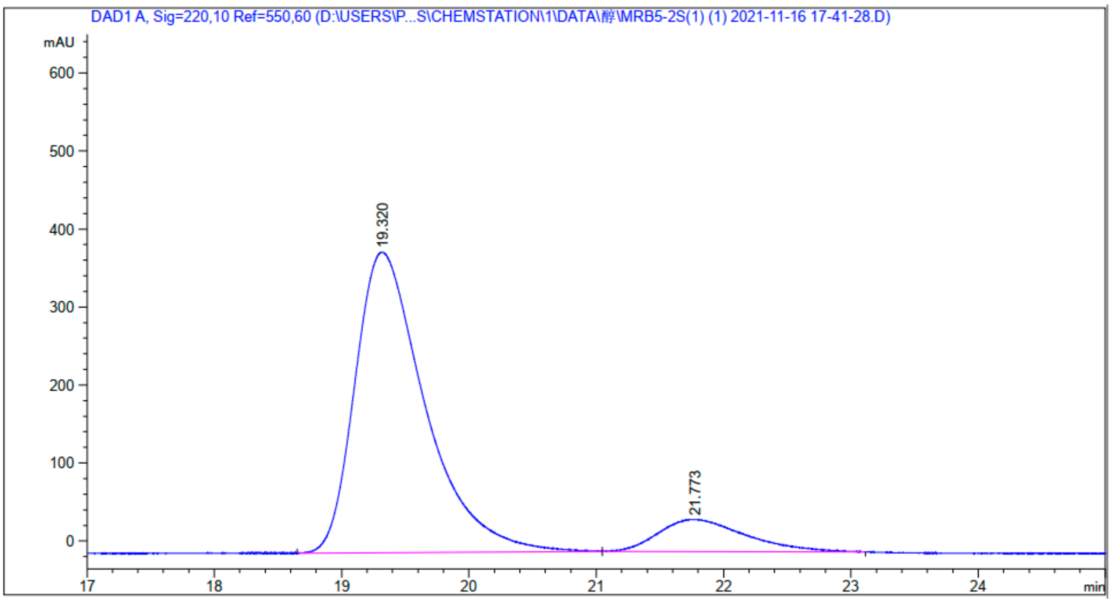

#	Time	Area	height	Width	Symmetry	Area%	Type
1	19.32	14723.9	385.2	0.4549	0.611	88.250	BV R
2	21.773	1960.5	41.1	0.558	0.684	11.750	BV R

Figure S75: Chiral product of 1-(4-methoxyphenyl)-3-phenylpropan-1-ol (3ab)

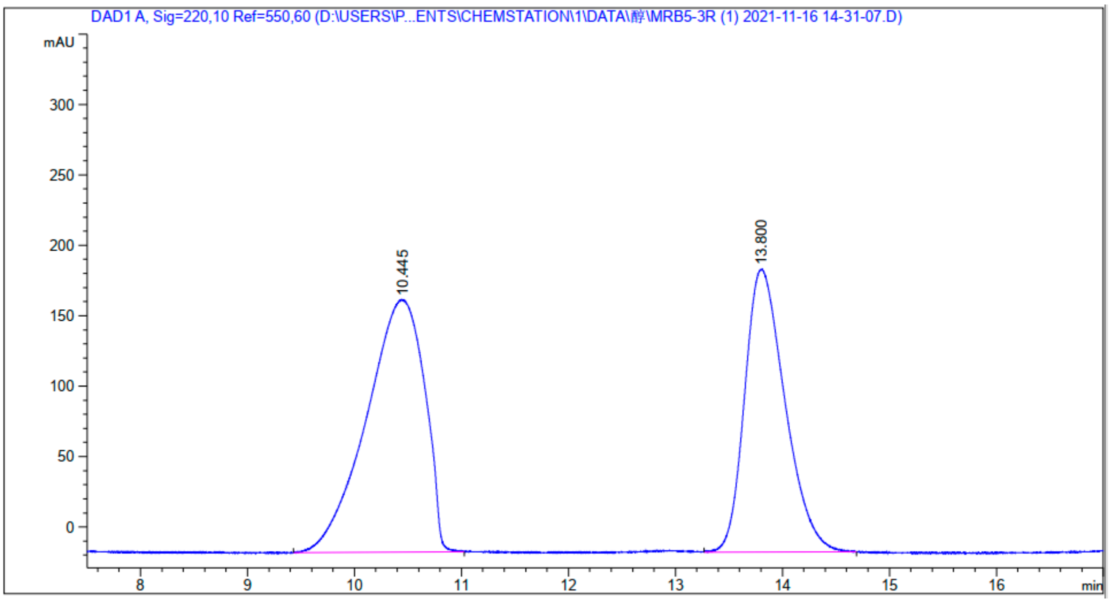

#	Time	Area	height	Width	Symmetry	Area%	Type
1	10.445	6762.8	179	0.4422	1.54	56.287	BB
2	13.8	5252	200.7	0.3063	0.666	43.713	BV R

Figure S76: Racemic product of 1-(4-(tert-butyl)phenyl)-3-phenylpropan-1-ol (3ac)

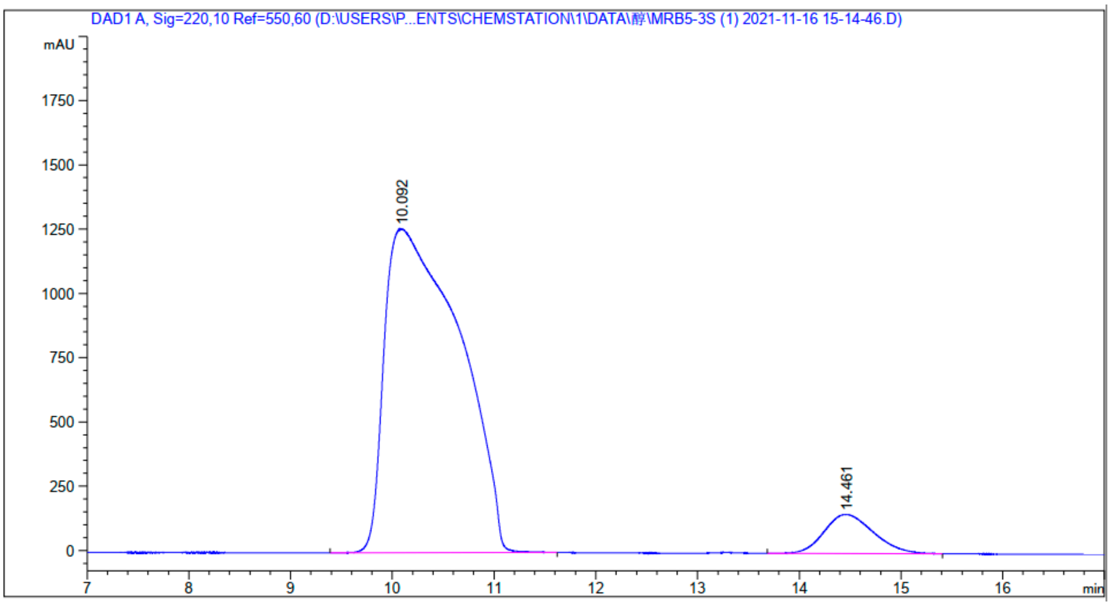

#	Time	Area	height	Width	Symmetry	Area%	Type
1	10.092	64930.2	1259.4	0.604	0.299	92.677	VV R
2	14.461	5130.3	152.2	0.3967	0.781	7.323	BB

Figure S77: Chiral product of 1-(4-(tert-butyl)phenyl)-3-phenylpropan-1-ol (3ac)

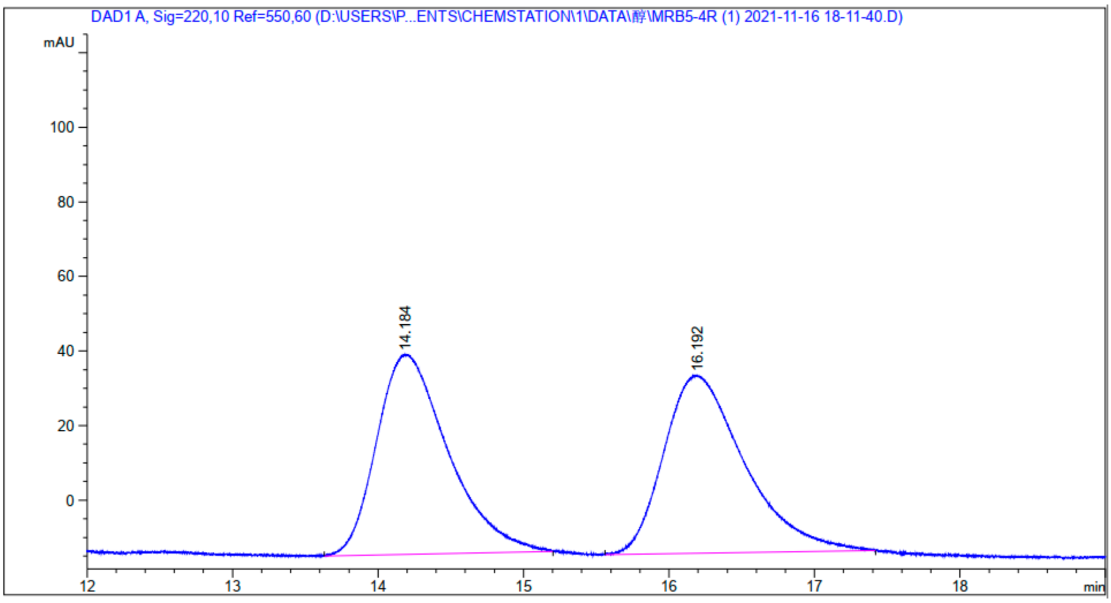

#	Time	Area	height	Width	Symmetry	Area%	Type
1	14.184	1833.1	53.6	0.4001	0.635	49.962	BV R
2	16.192	1835.9	47.7	0.4481	0.682	50.038	VV R

Figure S78: Racemic product of 1-(4-isopropoxyphenyl)-3-phenylpropan-1-ol (3ad)

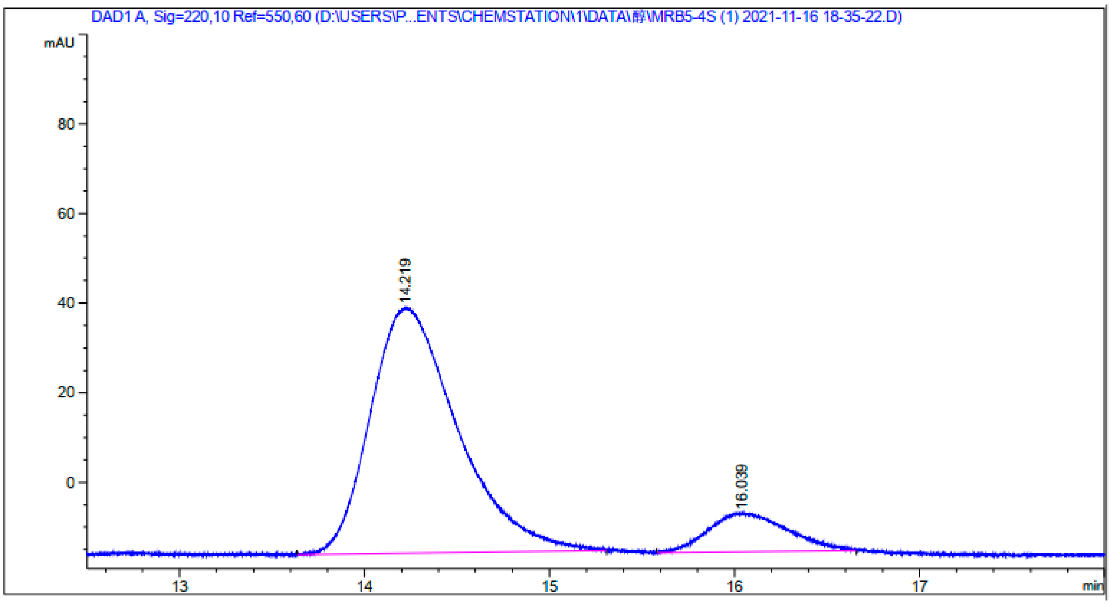

#	Time	Area	height	Width	Symmetry	Area%	Type
1	14.219	1800.4	54.7	0.3857	0.646	87.490	VV R
2	16.039	257.4	8.6	0.3509	0.718	12.510	VB R

Figure S79: Chiral product of 1-(4-isopropoxyphenyl)-3-phenylpropan-1-ol (3ad)

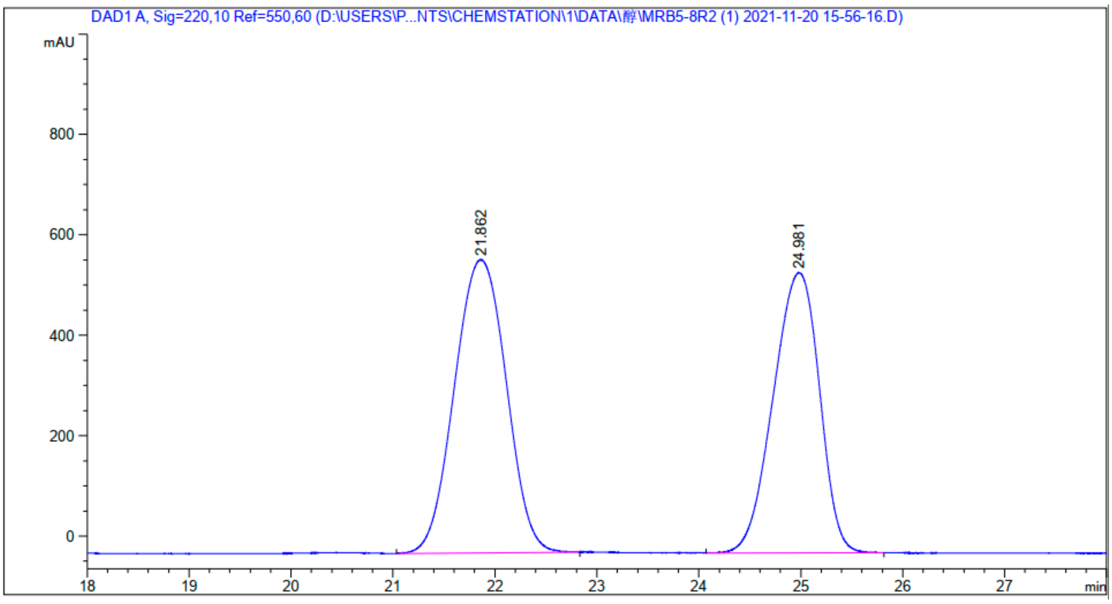

#	Time	Area	height	Width	Symmetry	Area%	Type
1	21.862	20594.6	583.7	0.4127	1.006	53.953	BV R
2	24.981	17577.1	557.9	0.3716	1.203	46.047	VV R

Figure S80: Racemic product of 1-(4-chlorophenyl)-3-phenylpropan-1-ol (3ae)

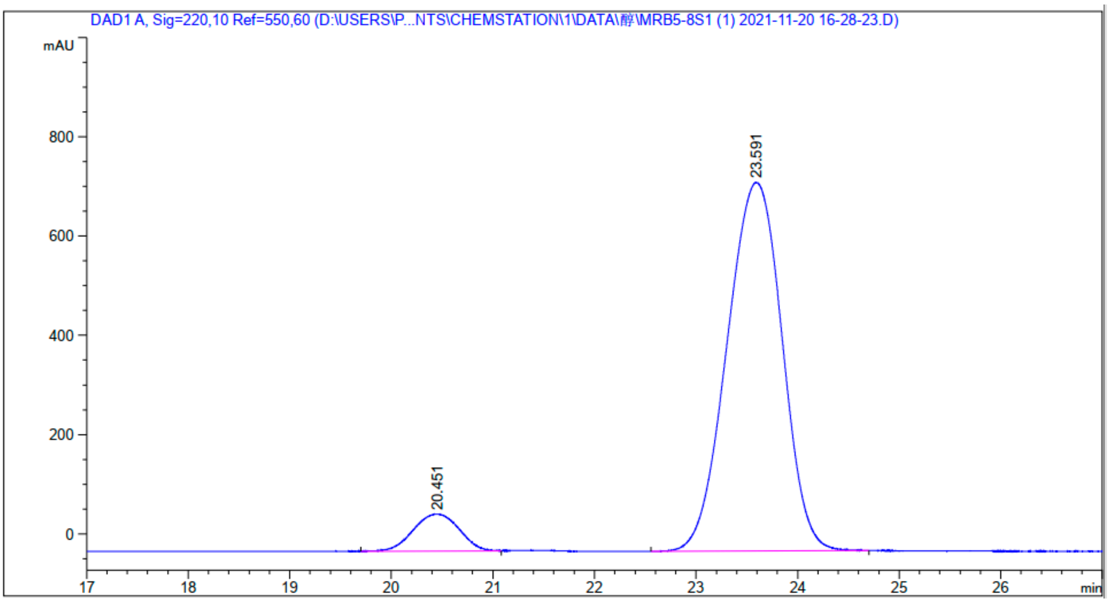

#	Time	Area	height	Width	Symmetry	Area%	Type
1	20.451	2325.4	74.4	0.3666	1.061	7.685	VB R
2	23.591	27932.3	742.1	0.4401	1.081	92.315	VV R

Figure S81: Chiral product of 1-(4-chlorophenyl)-3-phenylpropan-1-ol (3ae)

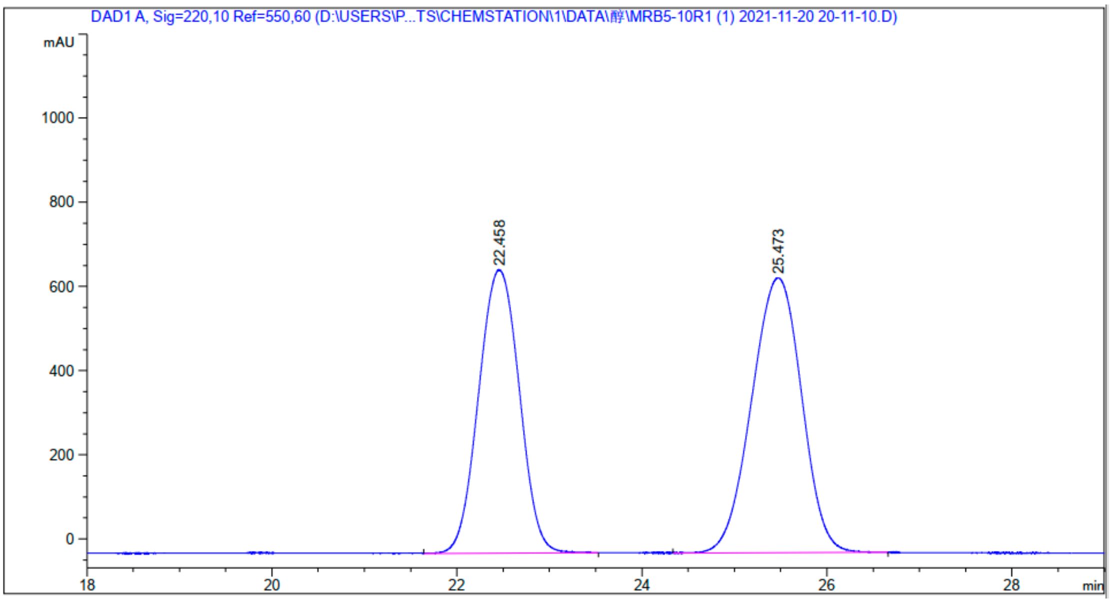

#	Time	Area	height	Width	Symmetry	Area%	Type
1	22.458	20531.7	672.9	0.3575	0.997	45.546	VV R
2	25.473	24547	653.1	0.44	1.094	54.454	VV R

Figure S82: Racemic product of 1-(4-bromophenyl)-3-phenylpropan-1-ol (3af)

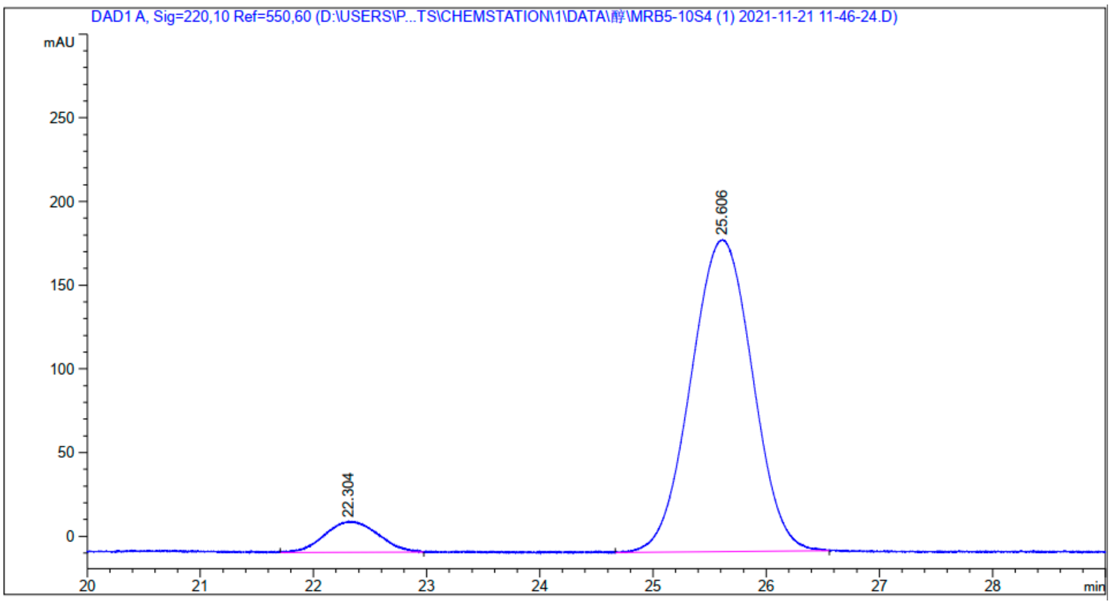

#	Time	Area	height	Width	Symmetry	Area%	Type
1	22.304	598.1	18.2	0.385	0.769	7.845	VB R
2	25.606	7025.6	186.1	0.4423	0.969	92.155	BV R

Figure S83: Chiral product of 1-(4-bromophenyl)-3-phenylpropan-1-ol (3af)

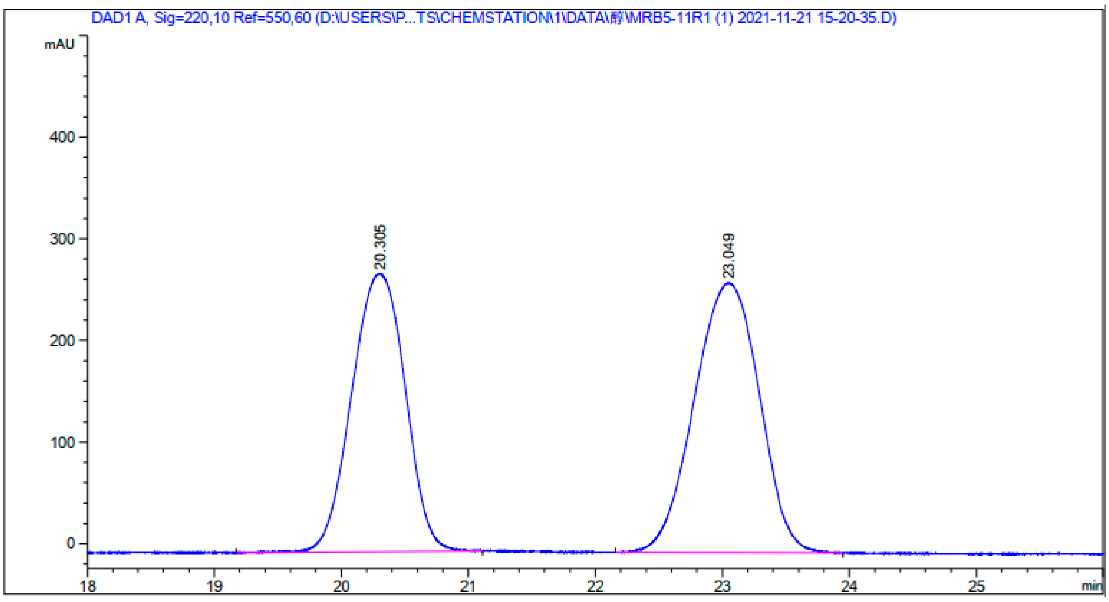

#	Time	Area	height	Width	Symmetry	Area%	Type
1	20.305	8027.6	273.6	0.3458	1.093	45.920	VV R
2	23.049	9454	265.2	0.4175	1.086	54.080	VV R

Figure S84: Racemic product of 1-(4-fluorophenyl)-3-phenylpropan-1-ol (3ag)

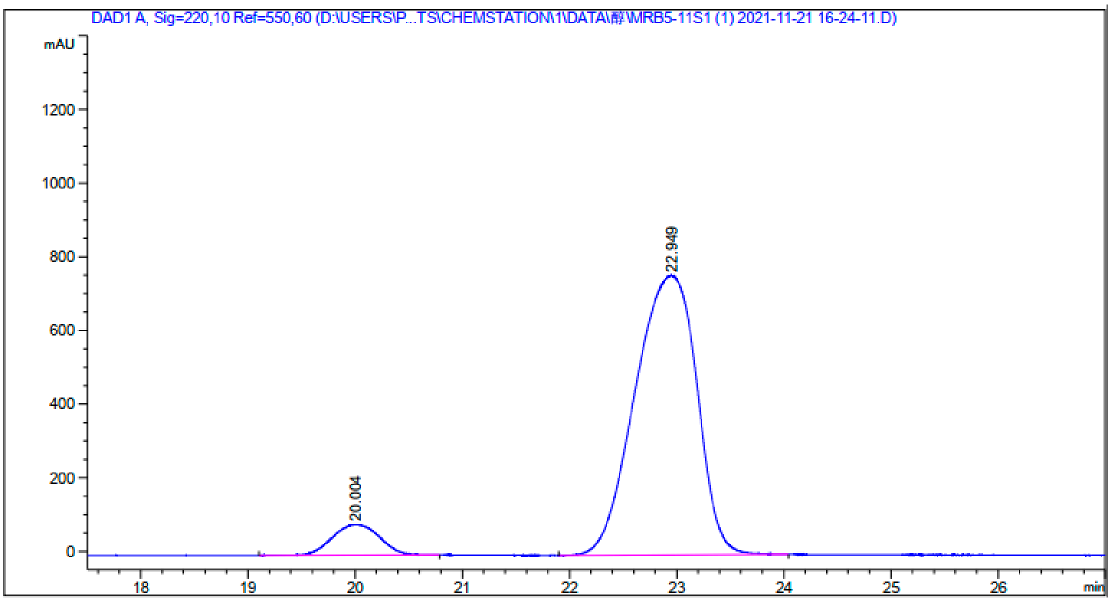

#	Time	Area	height	Width	Symmetry	Area%	Type
1	20.004	2544.7	82.6	0.3605	0.962	7.732	VV R
2	22.949	30365.2	757.3	0.4703	1.352	92.268	BV R

Figure S85: Chiral product of 1-(4-fluorophenyl)-3-phenylpropan-1-ol (3ag)

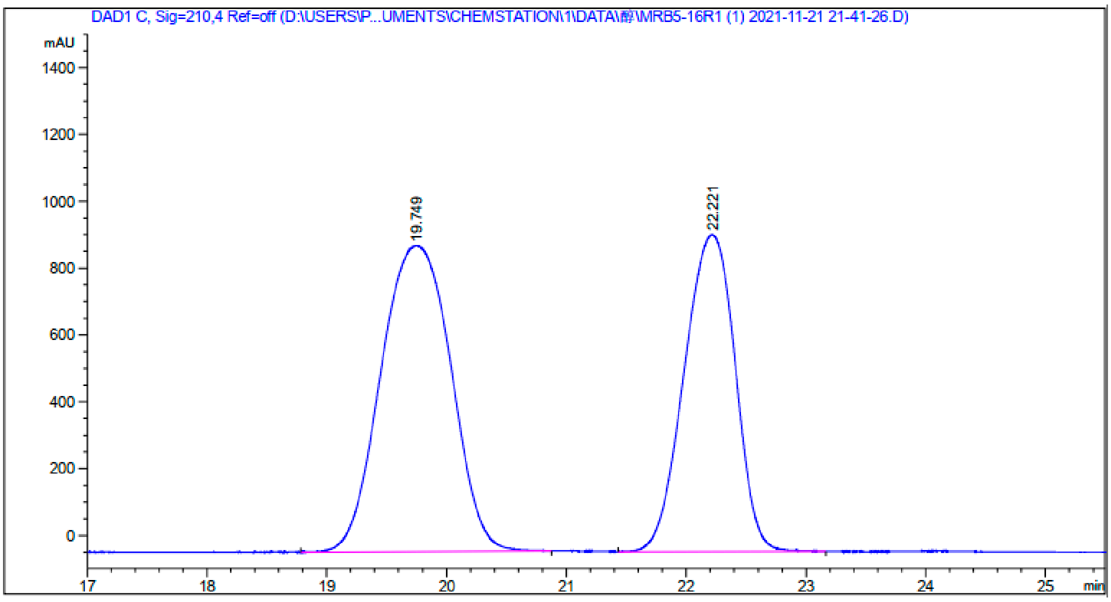

#	Time	Area	height	Width	Symmetry	Area%	Type
1	19.749	37201.5	915.5	0.4781	1.001	56.997	BV R
2	22.221	28067.7	948.5	0.3472	1.213	43.003	BV R

Figure S86: Racemic product of 3-phenyl-1-(4-(trifluoromethyl)phenyl)propan-1-ol (3ah)

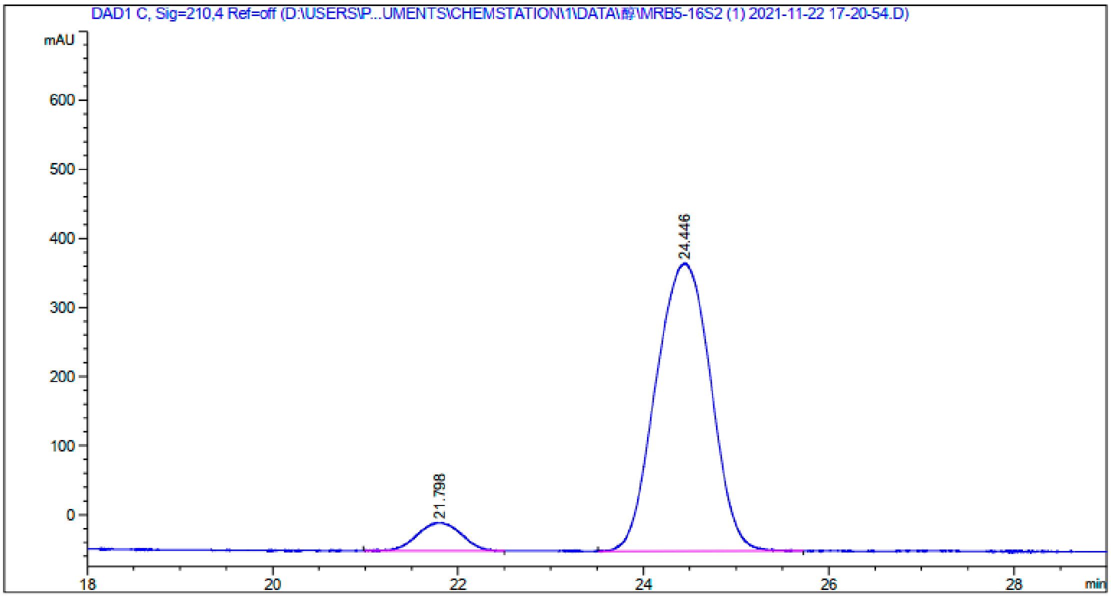

#	Time	Area	height	Width	Symmetry	Area%	Type
1	21.798	1349.8	40.4	0.394	0.976	7.309	VV R
2	24.446	17118.1	416	0.4831	1.063	92.691	VV R

Figure S87: Chiral product of 3-phenyl-1-(4-(trifluoromethyl)phenyl)propan-1-ol (3ah)

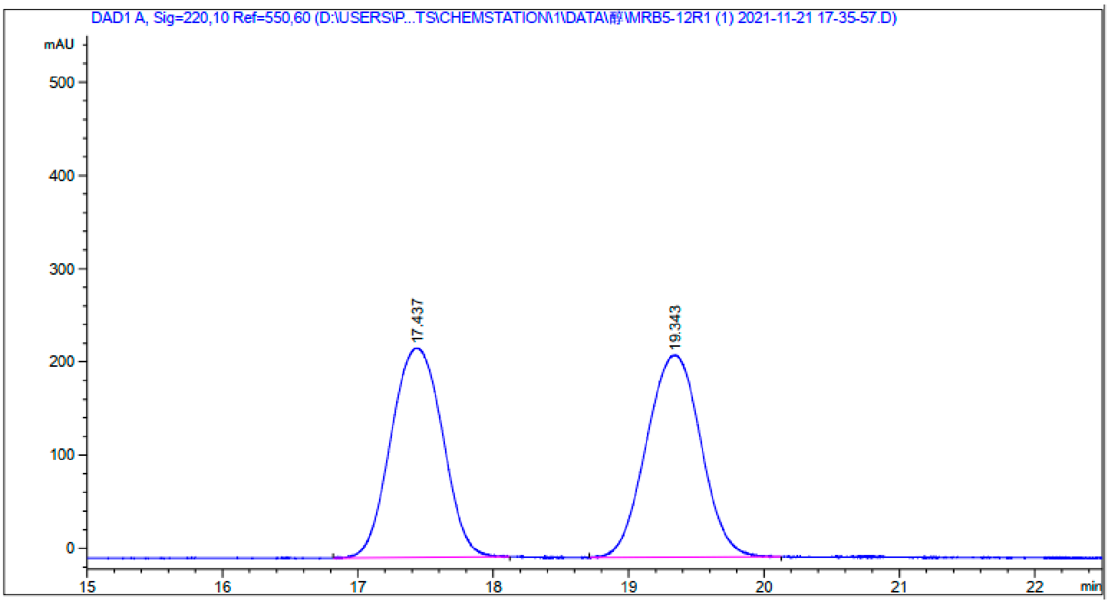

#	Time	Area	height	Width	Symmetry	Area%	Type
1	17.437	5928.5	224.3	0.3093	0.983	49.476	VV R
2	19.343	6054.2	216.8	0.3283	1.055	50.524	VV R

Figure S88: Racemic product of 1-(2-fluorophenyl)-3-phenylpropan-1-ol (3ai)

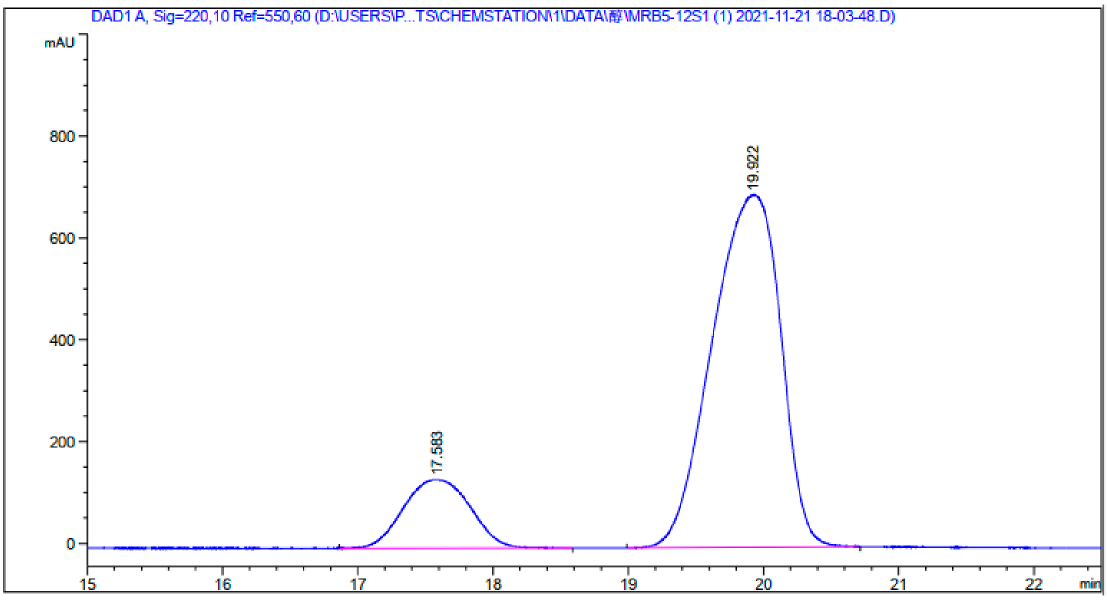

#	Time	Area	height	Width	Symmetry	Area%	Type
1	17.583	4650	135	0.4047	0.952	16.014	VV R
2	19.922	24387.7	691.8	0.4123	1.4	83.986	VB R

Figure S89: Chiral product of 1-(2-fluorophenyl)-3-phenylpropan-1-ol (3ai)

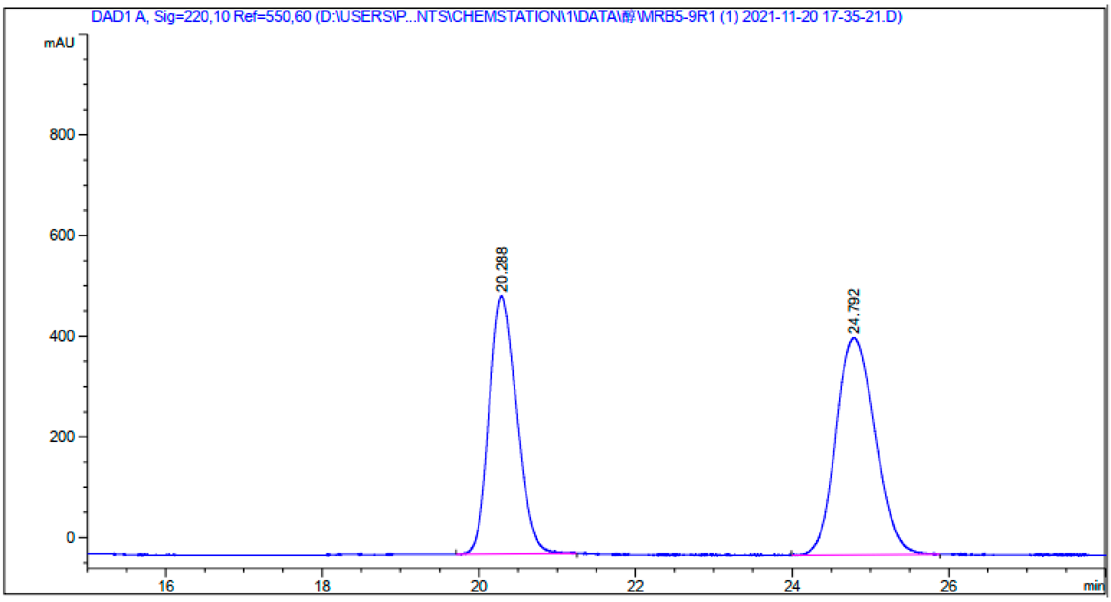

#	Time	Area	height	Width	Symmetry	Area%	Type
1	20.288	12517.6	511.2	0.2907	0.769	45.617	BV R
2	24.792	14923	430.8	0.4056	0.834	54.383	VV R

Figure S90: Racemic product of 1-(2-methoxyphenyl)-3-phenylpropan-1-ol (3aj)

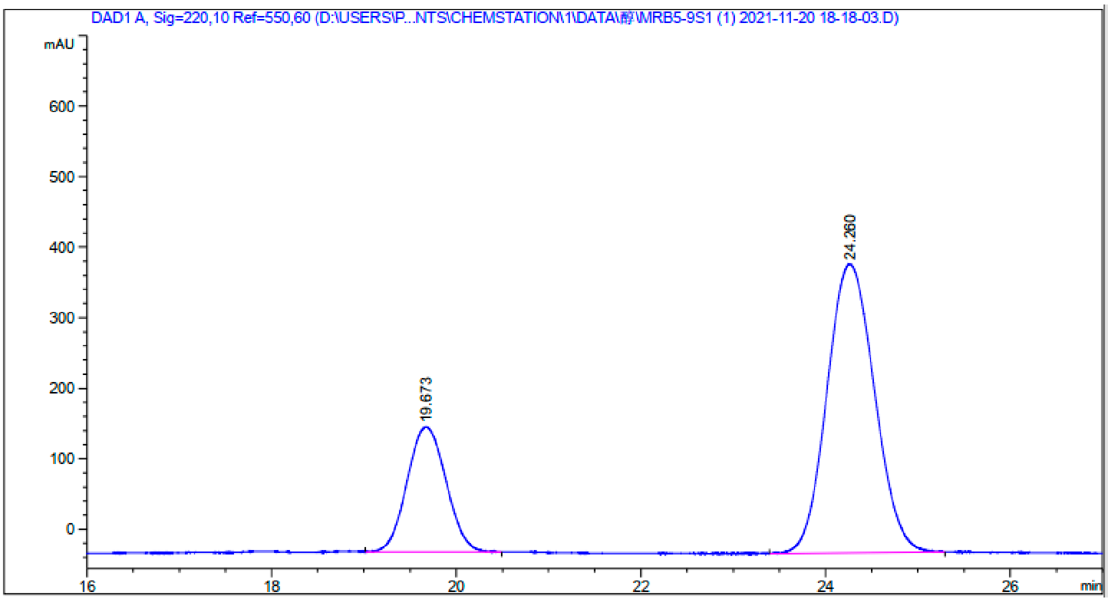

#	Time	Area	height	Width	Symmetry	Area%	Type
1	19.673	5191.7	177.2	0.3443	0.915	26.189	BV R
2	24.26	14632.3	409.7	0.4186	0.88	73.811	BB

Figure S91: Chiral product of 1-(2-methoxyphenyl)-3-phenylpropan-1-ol (3aj)

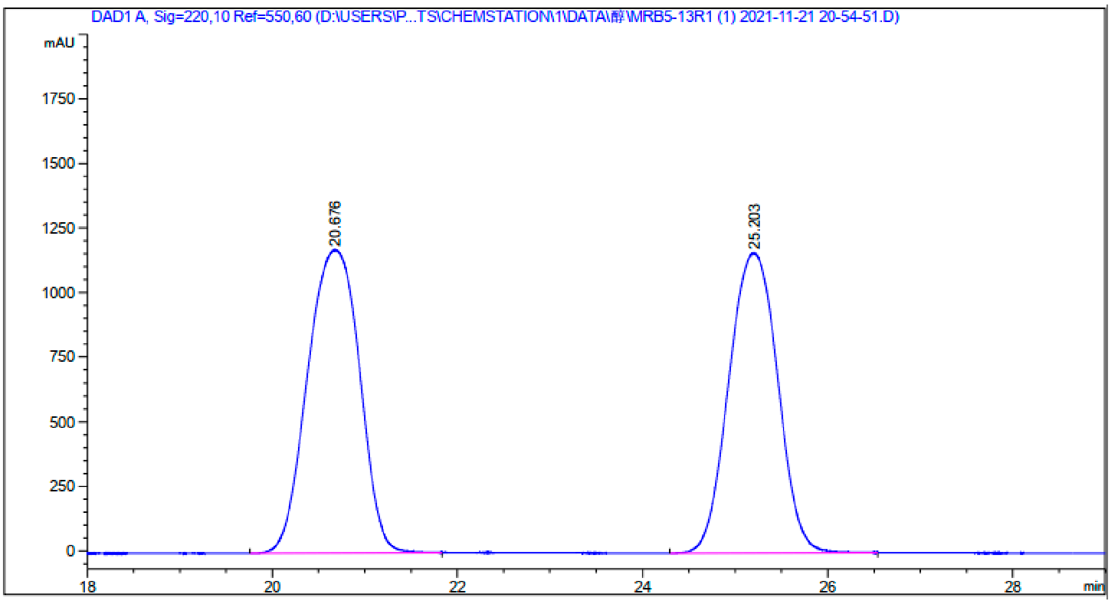

#	Time	Area	height	Width	Symmetry	Area%	Type
1	20.676	45737.8	1172.8	0.4565	1.071	51.818	VV R
2	25.203	42528.3	1160.1	0.4307	1.015	48.182	BV R

Figure S92: Racemic product of 1-(3-chlorophenyl)-3-phenylpropan-1-ol (3ak)

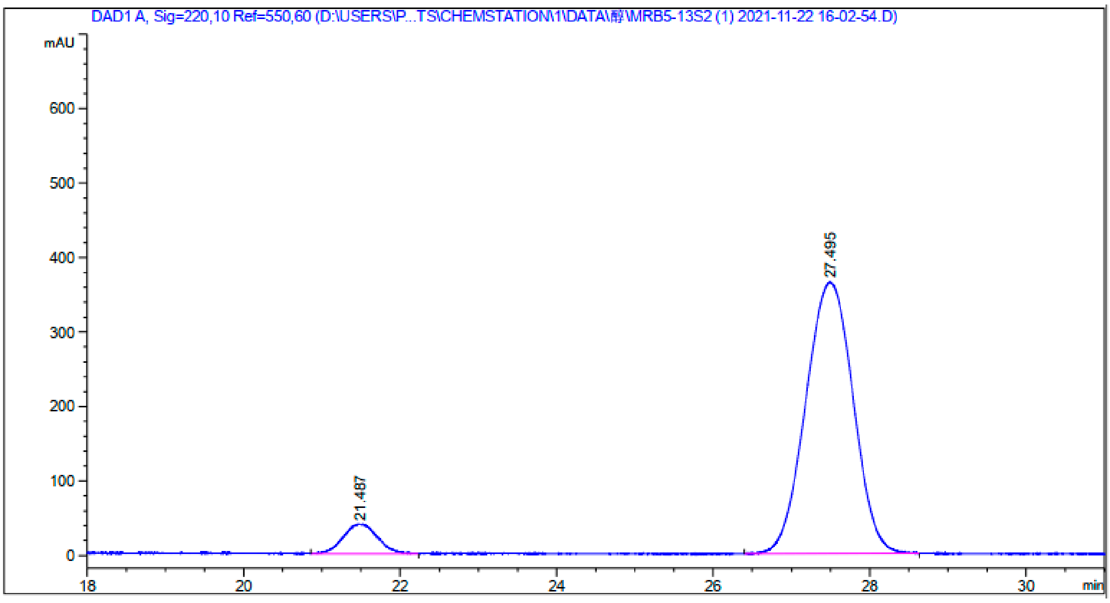

#	Time	Area	height	Width	Symmetry	Area%	Type
1	21.487	1190.7	39.4	0.3551	0.941	7.203	VV R
2	27.495	15340.8	364.4	0.4921	1.037	92.797	BV R

Figure S93: Chiral product of 1-(3-chlorophenyl)-3-phenylpropan-1-ol (3ak)

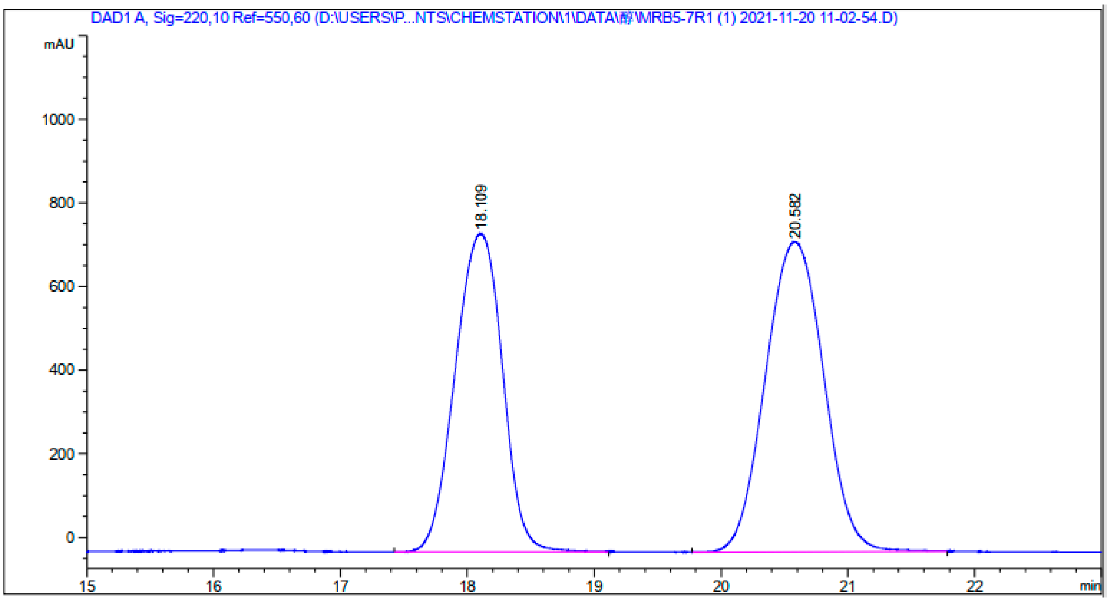

#	Time	Area	height	Width	Symmetry	Area%	Type
1	18.109	19686.5	760.6	0.303	1.159	45.445	VB R
2	20.582	23632.6	740.8	0.3736	0.985	54.555	BV R

Figure S94: Racemic product of 3-phenyl-1-(m-tolyl)propan-1-ol (3al)

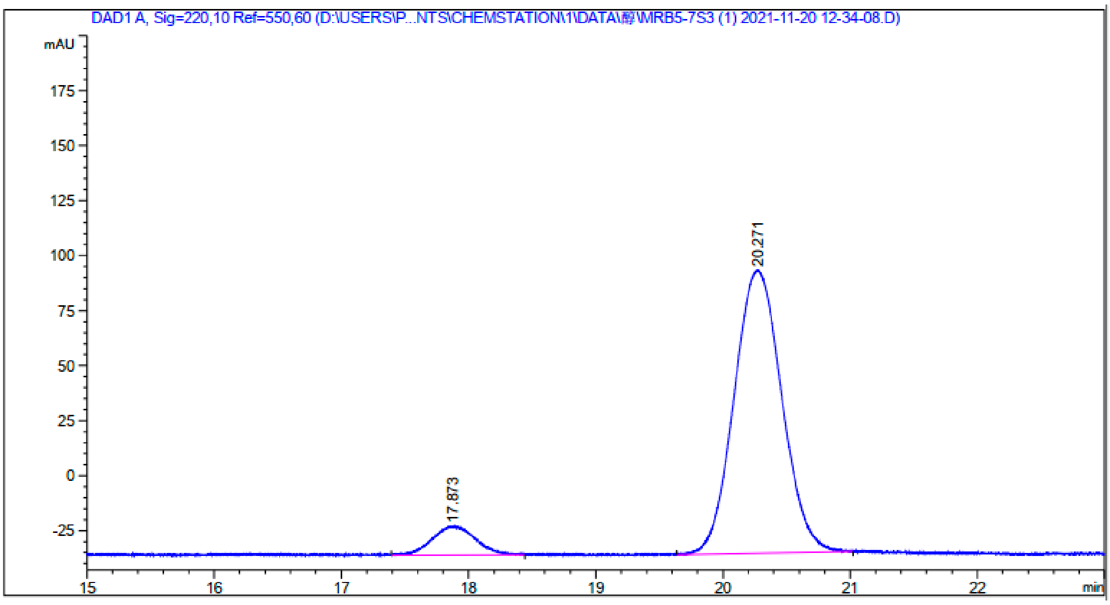

#	Time	Area	height	Width	Symmetry	Area%	Type
1	17.873	308.2	12.9	0.2823	0.863	8.420	VV R
2	20.271	3352.5	128.4	0.306	0.933	91.580	VV R

Figure S95: Chiral product of 3-phenyl-1-(m-tolyl)propan-1-ol (3al)

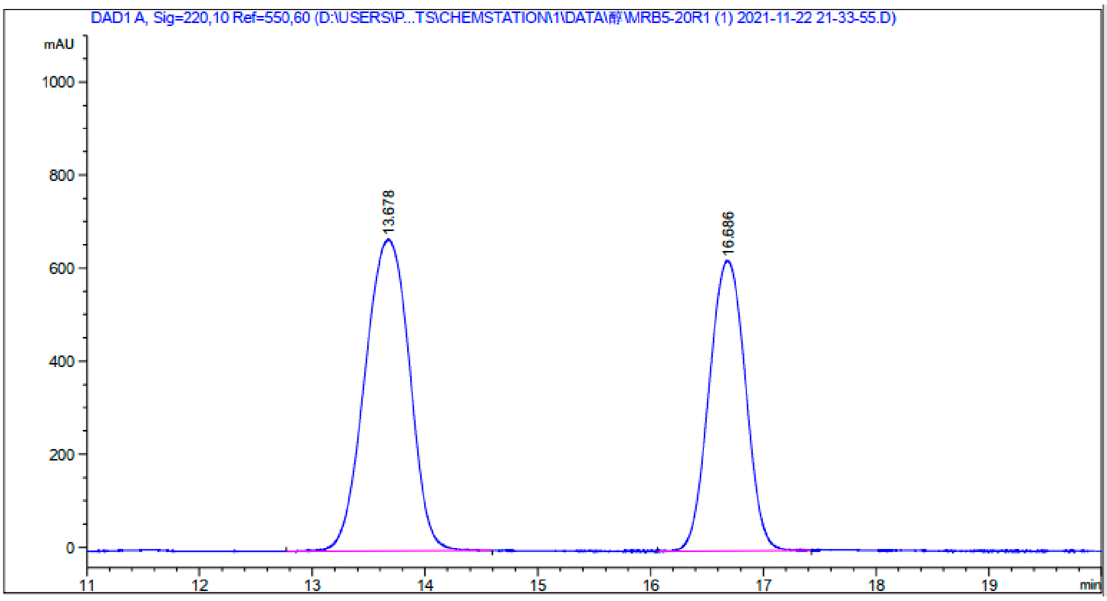

#	Time	Area	height	Width	Symmetry	Area%	Type
1	13.678	18443.7	669	0.3261	1.092	56.969	VB R
2	16.686	13931.4	623.4	0.2632	1.026	43.031	VV R

Figure S96: Racemic product of 1-(3-isopropylphenyl)-3-phenylpropan-1-ol (3am)

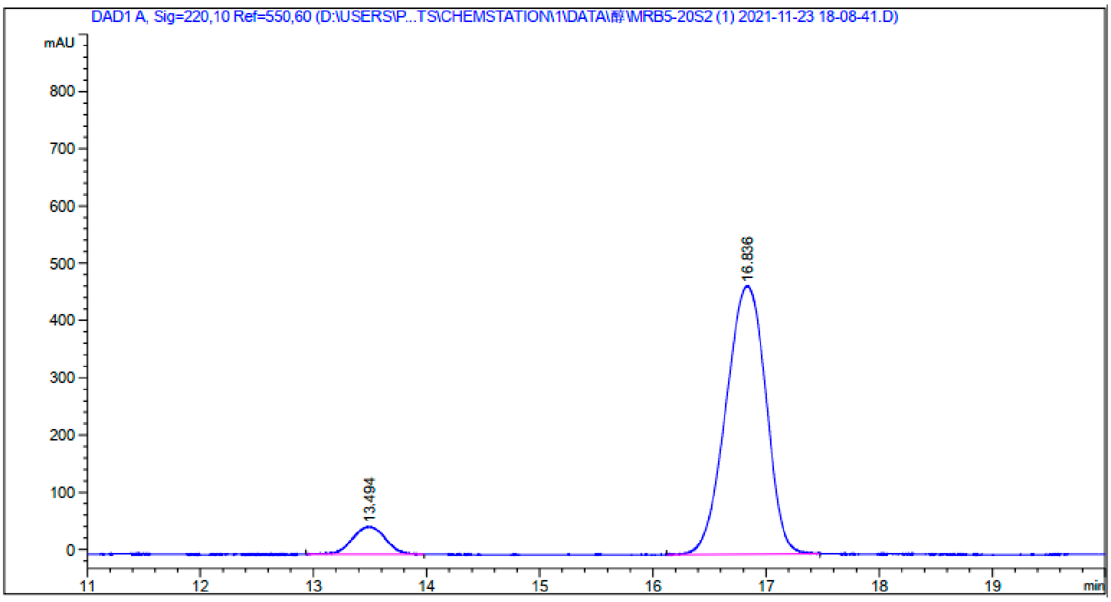

#	Time	Area	height	Width	Symmetry	Area%	Type
1	13.494	968.4	47.5	0.241	1.027	7.786	VV R
2	16.836	11470.6	468	0.2905	1.113	92.214	VV R

Figure S97: Chiral product of 1-(3-isopropylphenyl)-3-phenylpropan-1-ol (3am)

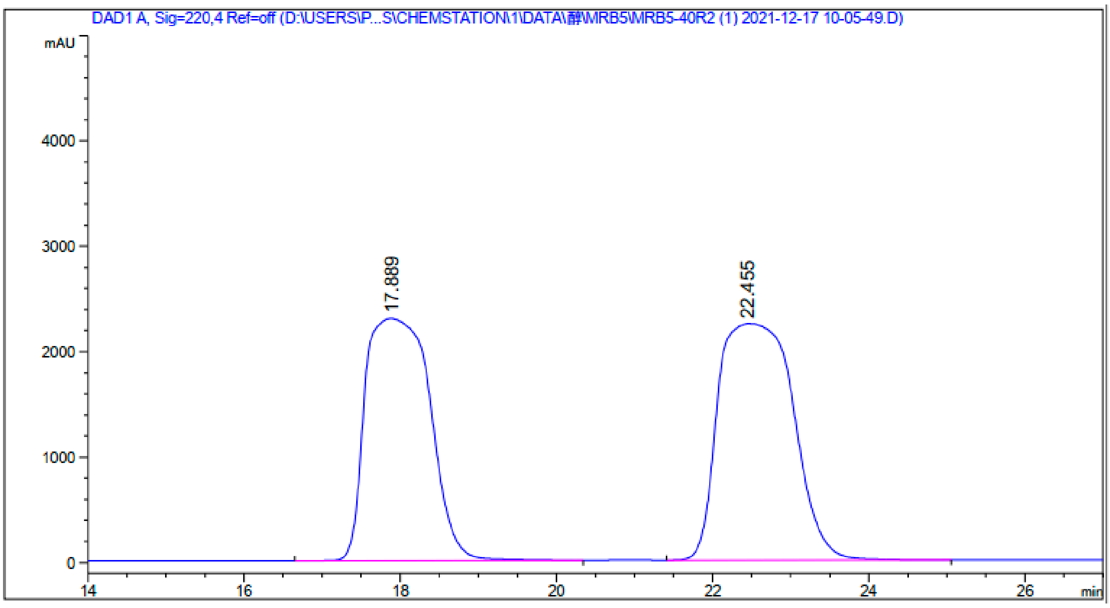

#	Time	Area	height	Width	Symmetry	Area%	Type
1	17.889	132271.5	2297.8	0.6976	0.663	46.848	BB
2	22.455	150070.1	2244.8	0.794	0.666	53.152	BB

Figure S98: Racemic product of 1-(naphthalen-2-yl)-3-phenylpropan-1-ol (3an)

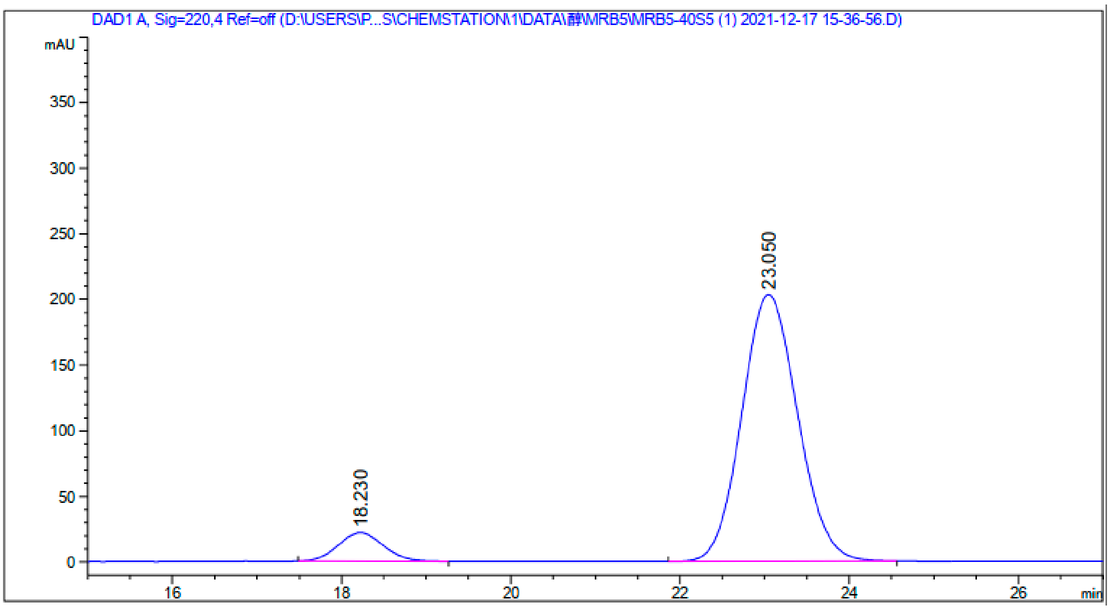

#	Time	Area	height	Width	Symmetry	Area%	Type
1	18.23	811.2	21.7	0.5674	0.95	8.010	BB
2	23.05	9316.2	203.2	0.7129	0.887	91.990	BB

Figure S99: Chiral product of 1-(naphthalen-2-yl)-3-phenylpropan-1-ol (3an)

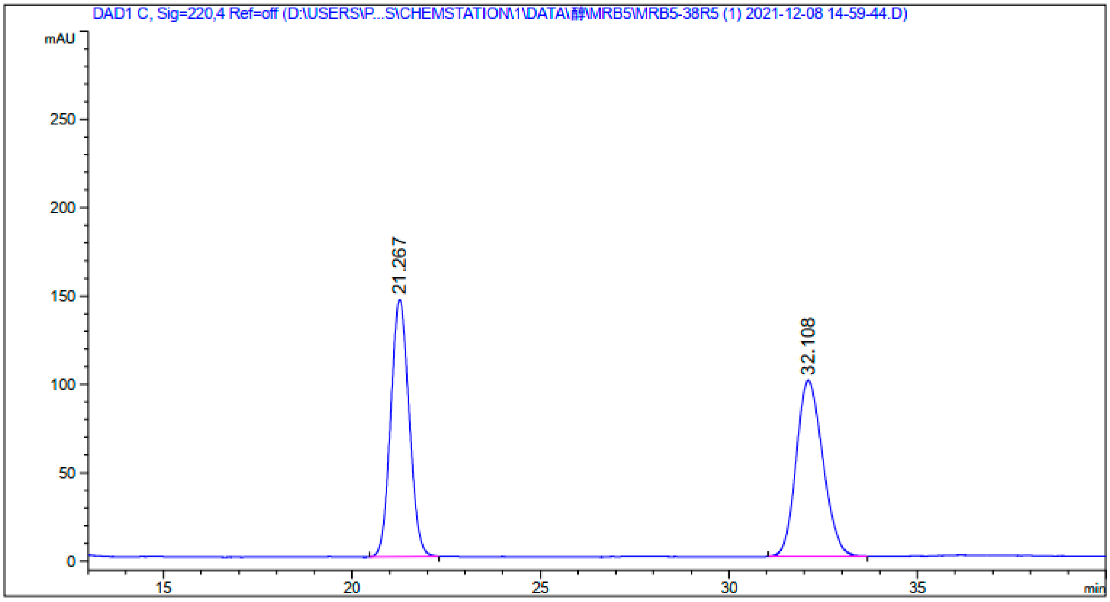

#	Time	Area	height	Width	Symmetry	Area%	Type
1	21.267	4923.2	145.4	0.5309	0.886	50.179	BB
2	32.108	4888.2	99.7	0.7579	0.8	49.821	BB

Figure S100: Racemic product of 3-phenyl-1-(thiophen-2-yl)propan-1-ol (3ao)

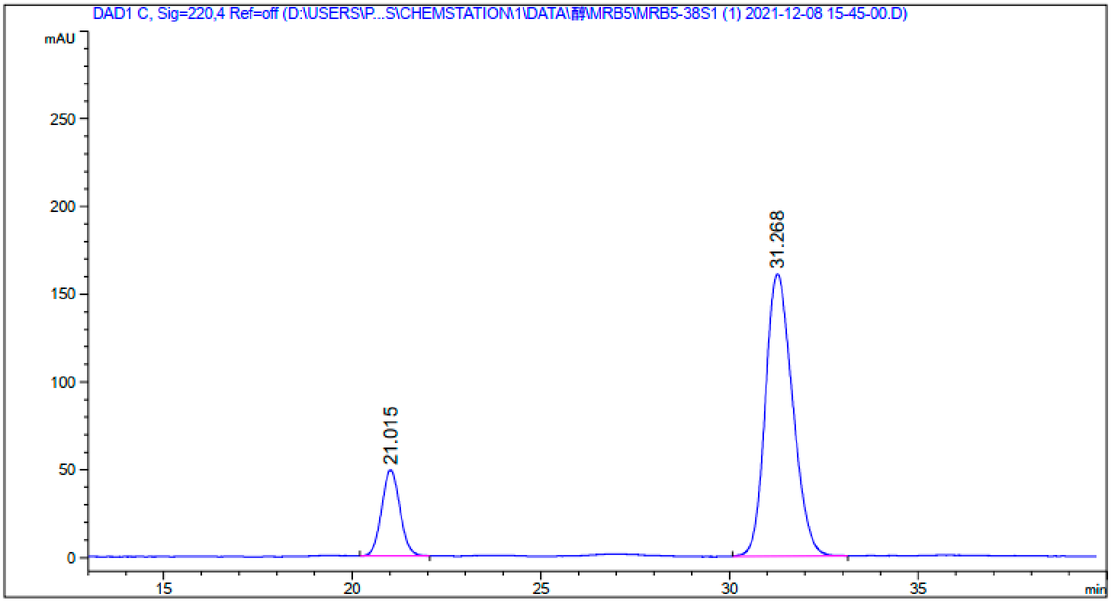

#	Time	Area	height	Width	Symmetry	Area%	Type
1	21.015	1650	48.8	0.5261	0.938	17.009	BB
2	31.268	8050.8	160.6	0.7688	0.764	82.991	BB

Figure S101: Chiral product of 3-phenyl-1-(thiophen-2-yl)propan-1-ol (3ao)

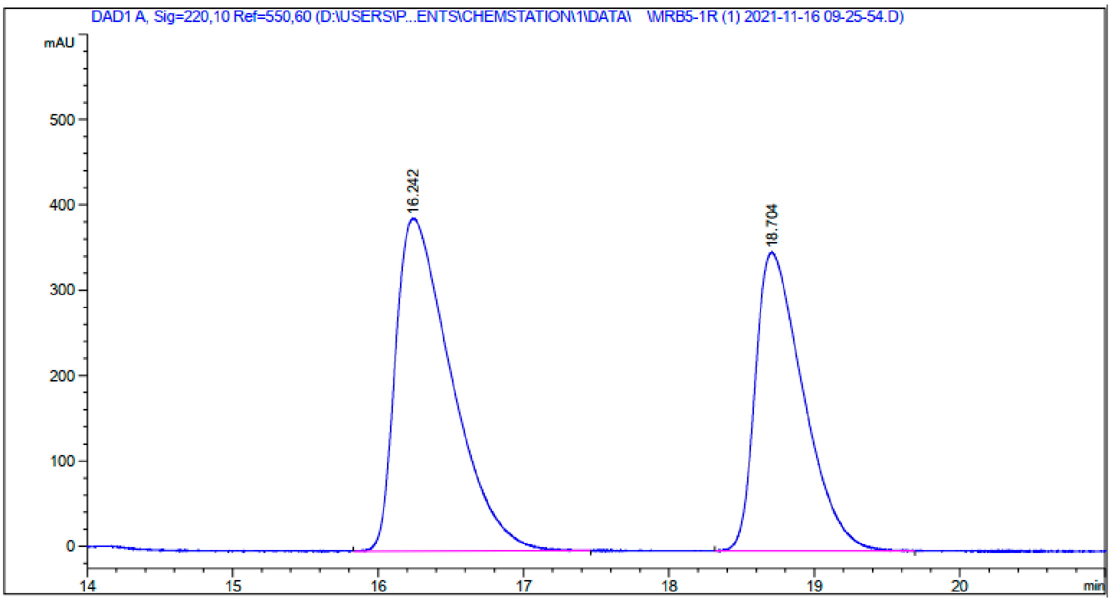

#	Time	Area	height	Width	Symmetry	Area%	Type
1	16.242	10383.2	389.7	0.3123	0.448	56.502	VV R
2	18.704	7993.4	349.6	0.2683	0.494	43.498	BV R

Figure S102: Racemic product of 1,3-diphenylpropan-1-ol (3ap)

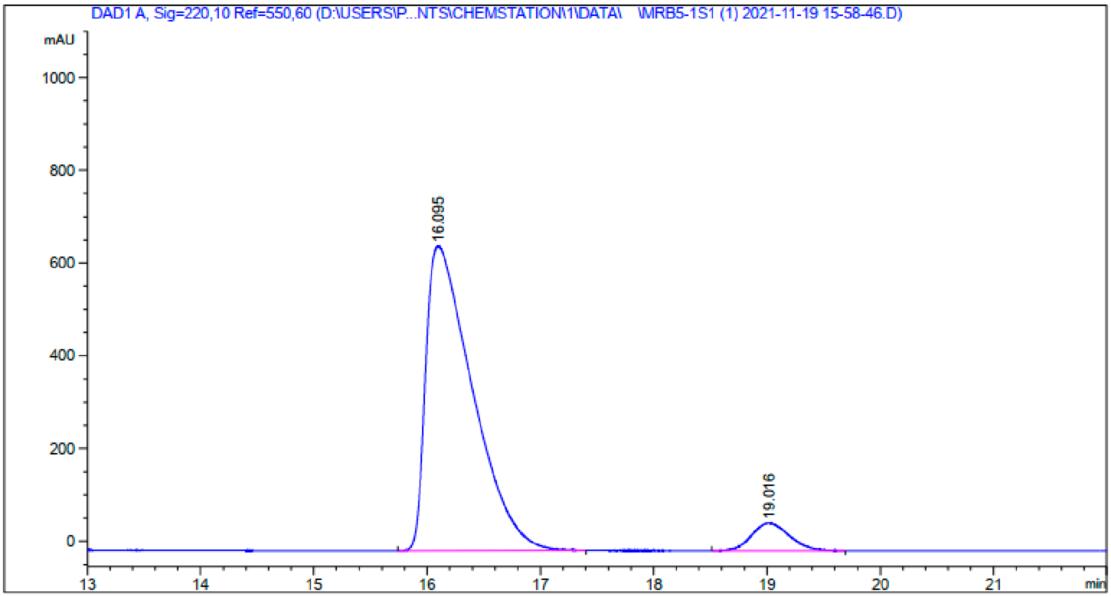

#	Time	Area	height	Width	Symmetry	Area%	Type
1	16.095	18775.3	656.7	0.3366	0.338	93.122	BV R
2	19.016	1386.7	59.8	0.2748	0.811	6.878	BB

Figure S103: Chiral product of 1,3-diphenylpropan-1-ol (3ap)

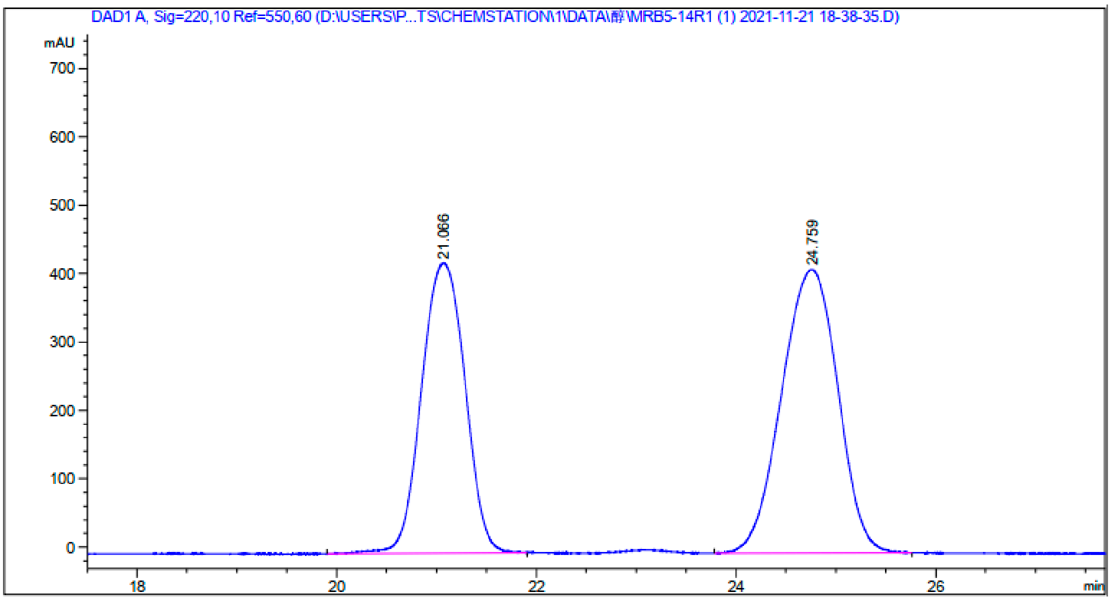

#	Time	Area	height	Width	Symmetry	Area%	Type
1	21.066	12828.6	423.9	0.3545	0.972	44.176	VV R
2	24.759	16211.4	414.1	0.4617	1.118	55.824	BV R

Figure S104: Racemic product of 1-(3-chloro-4-fluorophenyl)-3-phenylpropan-1-ol (3aq)

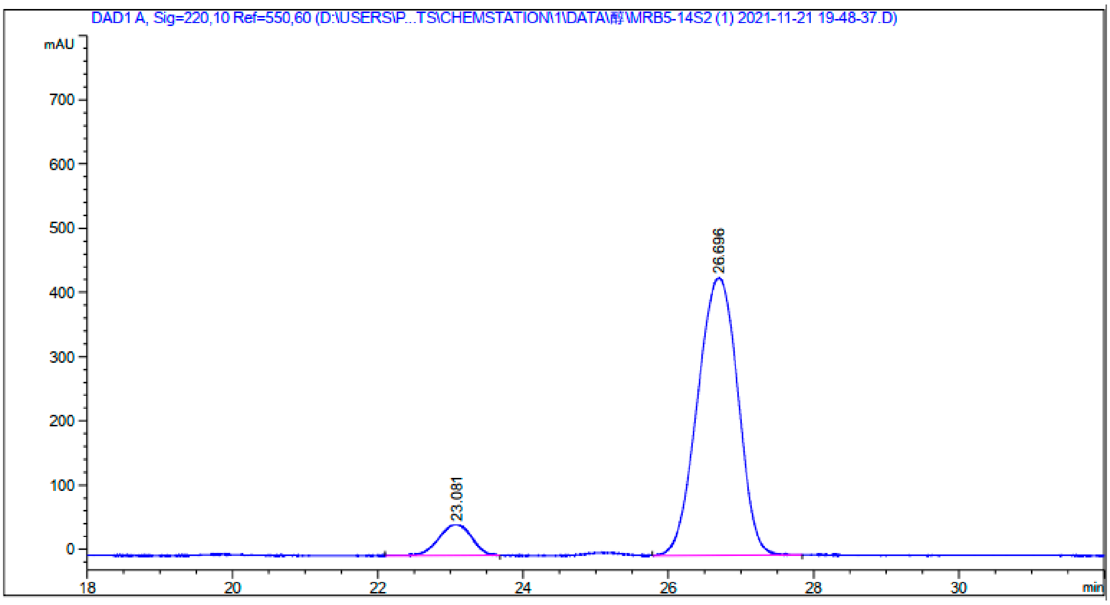

#	Time	Area	height	Width	Symmetry	Area%	Type
1	23.081	1508.4	47.9	0.3712	1.108	8.309	VV R
2	26.696	16644.8	431.6	0.4514	1.088	91.691	BV R

Figure S105: Chiral product of 1-(3-chloro-4-fluorophenyl)-3-phenylpropan-1-ol (3aq)

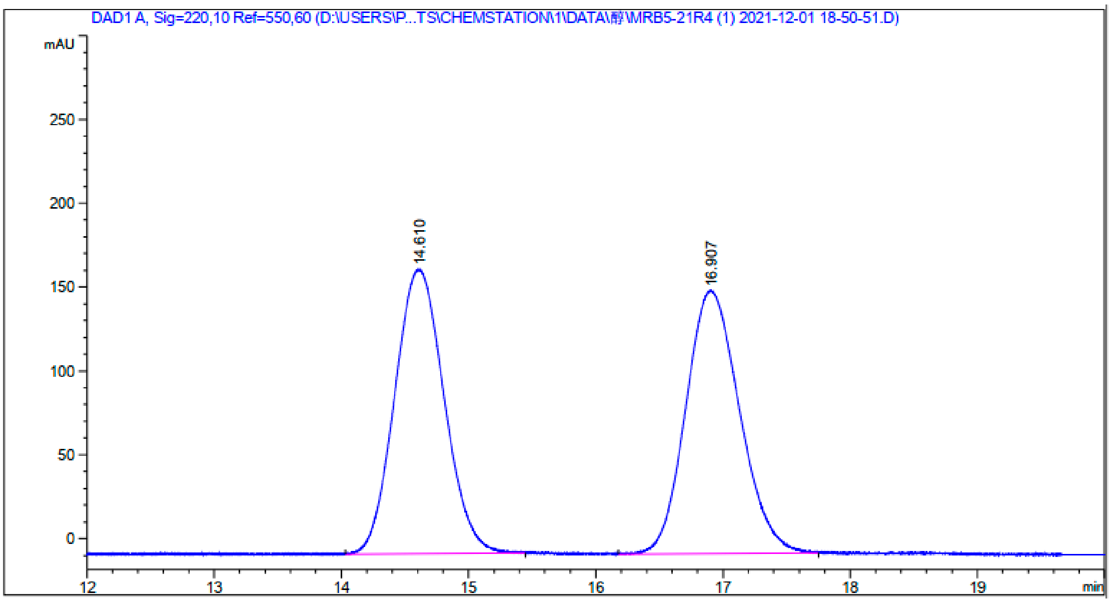

#	Time	Area	height	Width	Symmetry	Area%	Type
1	14.61	4575.4	169.5	0.3174	0.932	49.870	BB
2	16.907	4599.2	156.6	0.3467	0.87	50.130	VV R

Figure S106: Racemic product of 3-(4-methoxyphenyl)-1-(p-tolyl)propan-1-ol (3ba)

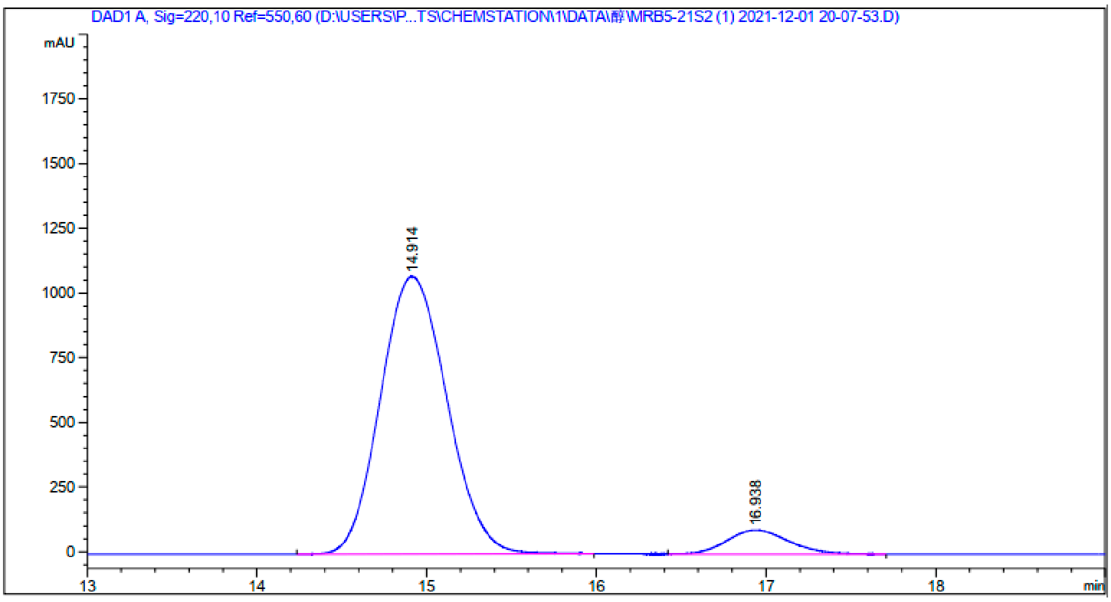

#	Time	Area	height	Width	Symmetry	Area%	Type
1	14.914	29195.2	1072	0.3192	0.875	92.589	BB
2	16.938	2336.8	92.3	0.2963	0.829	7.411	BV R

Figure S107: Chiral product of 3-(4-methoxyphenyl)-1-(p-tolyl)propan-1-ol (3ba)

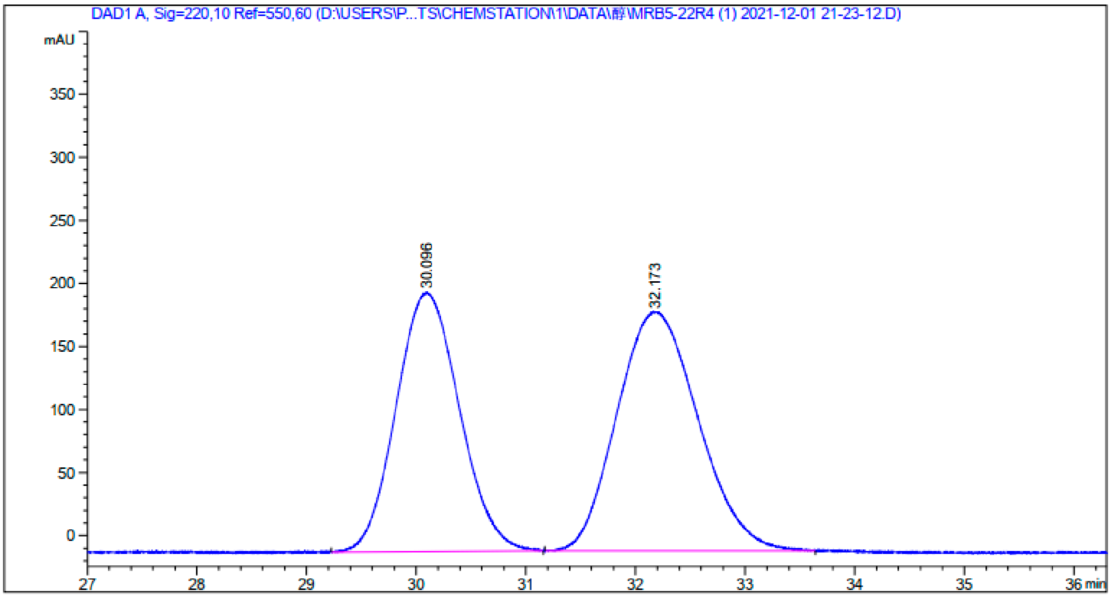

#	Time	Area	height	Width	Symmetry	Area%	Type
1	30.096	8228.9	204.8	0.4712	0.908	45.834	BV R
2	32.173	9724.7	189.1	0.6011	0.833	54.166	BV R

Figure S108: Racemic product of 1-(4-chlorophenyl)-3-(4-methoxyphenyl)propan-1-ol (3be)

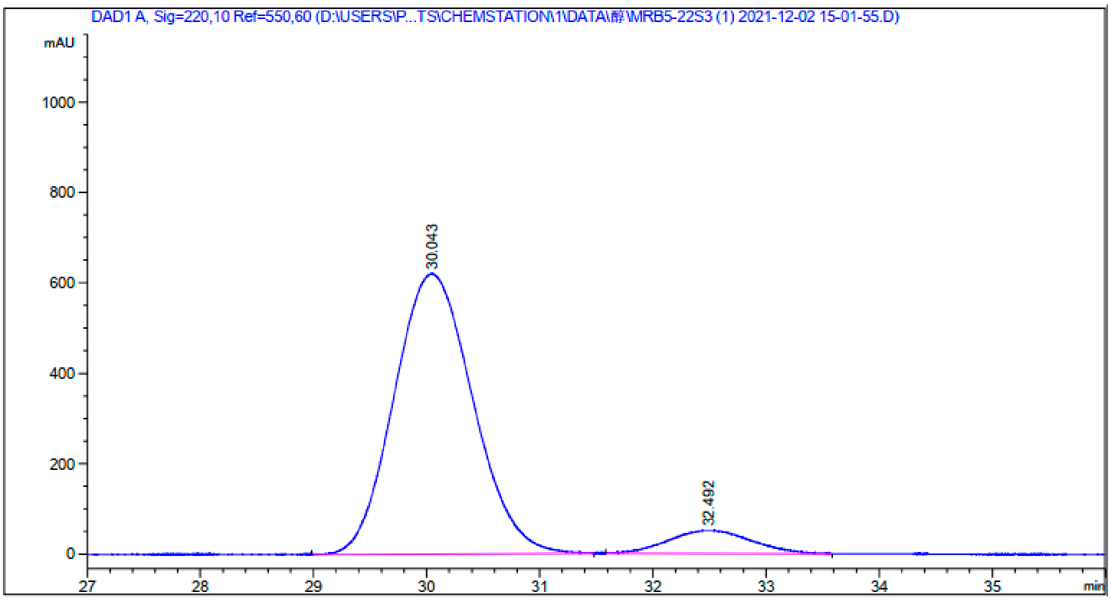

#	Time	Area	height	Width	Symmetry	Area%	Type
1	30.043	29840	618.5	0.5639	0.88	92.140	VV R
2	32.492	2545.4	50.4	0.5906	0.905	7.860	BV R

Figure S109: Chiral product of 1-(4-chlorophenyl)-3-(4-methoxyphenyl)propan-1-ol (3be)

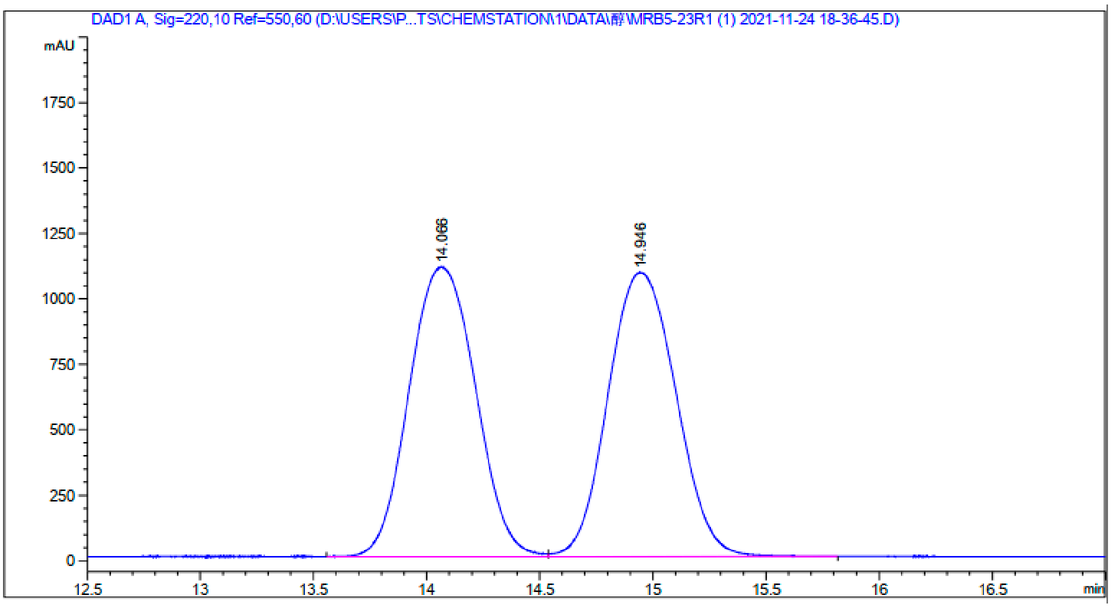

#	Time	Area	height	Width	Symmetry	Area%	Type
1	14.066	22538.8	1105.6	0.2413	0.92	49.906	BV
2	14.946	22623.8	1084.8	0.2538	0.895	50.094	VV R

Figure S110: Racemic product of 3-(5-methylfuran-2-yl)-1-(p-tolyl)propan-1-ol (3ca)

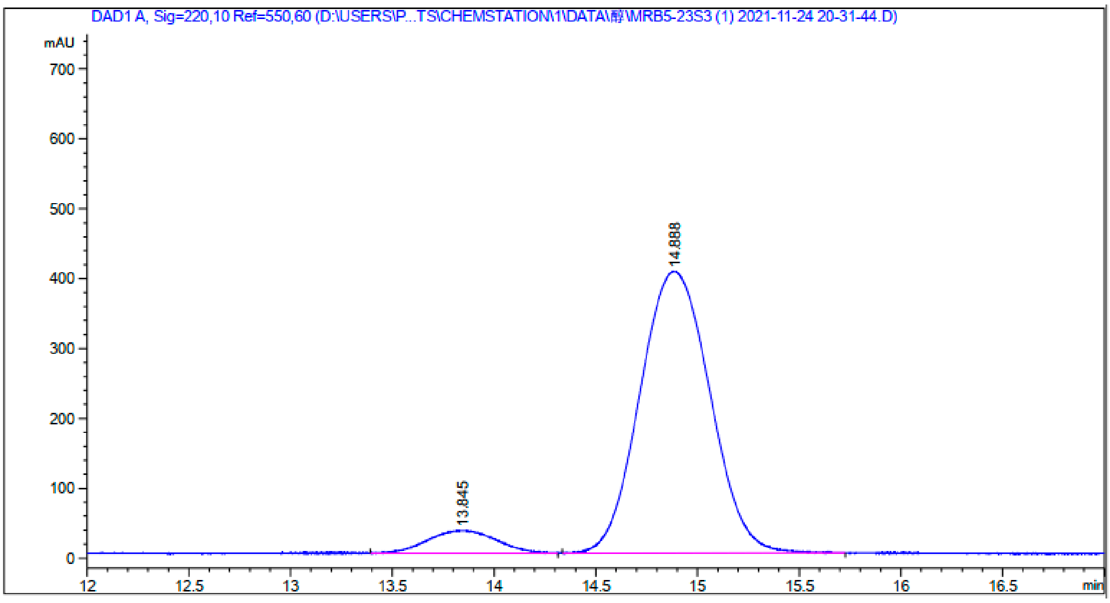

#	Time	Area	height	Width	Symmetry	Area%	Type
1	13.845	757	32.2	0.2766	1.001	7.276	BV R
2	14.888	9647.5	403	0.2853	0.952	92.724	BV R

Figure S111: Chiral product of 3-(5-methylfuran-2-yl)-1-(p-tolyl)propan-1-ol (3ca)

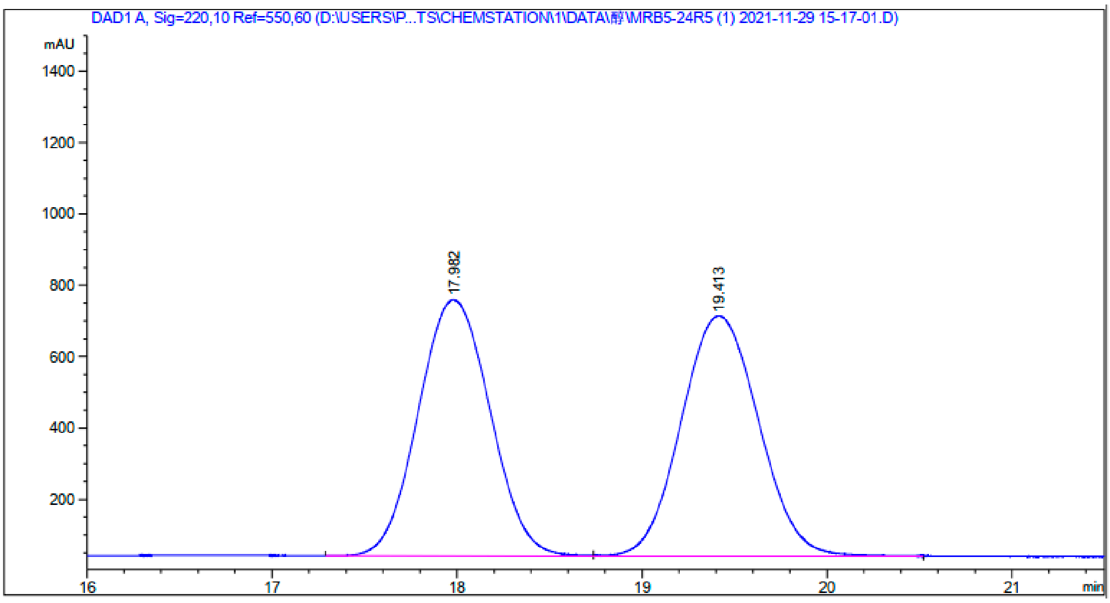

#	Time	Area	height	Width	Symmetry	Area%	Type
1	17.982	19339.4	717.6	0.3164	0.956	49.795	BV
2	19.413	19498.5	672.6	0.3407	0.907	50.205	VB

Figure S112: Racemic product of 1-(4-chlorophenyl)-3-(5-methylfuran-2-yl)propan-1-ol (3ce)

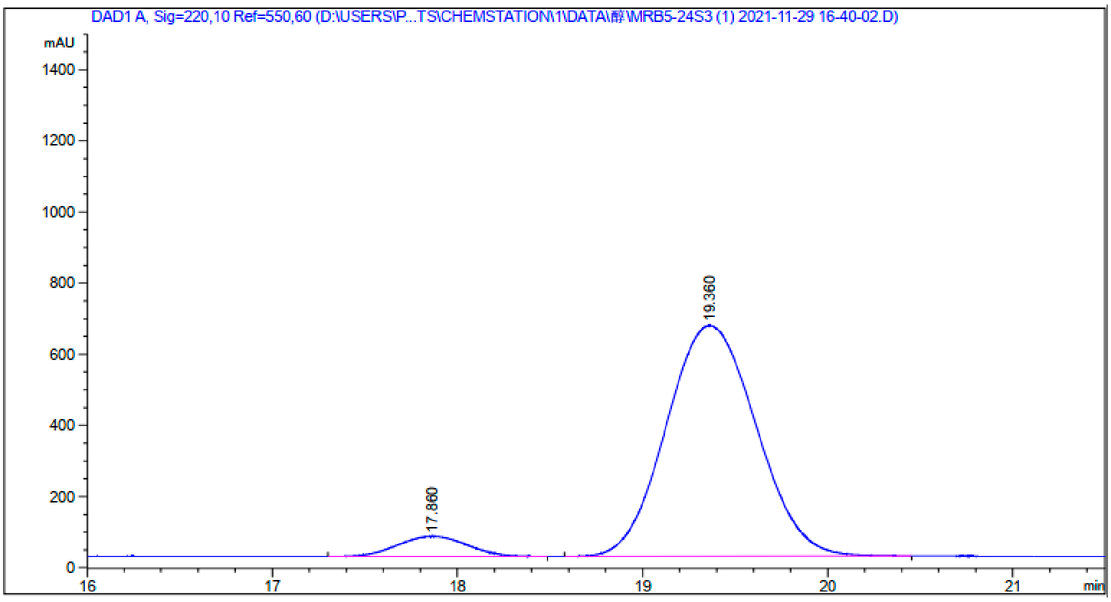

#	Time	Area	height	Width	Symmetry	Area%	Type
1	17.86	1484.1	56.7	0.3075	0.93	6.351	BV R
2	19.36	21883.7	648.8	0.3955	0.897	93.649	BV R

Figure S113: Chiral product of 1-(4-chlorophenyl)-3-(5-methylfuran-2-yl)propan-1-ol (3ce)

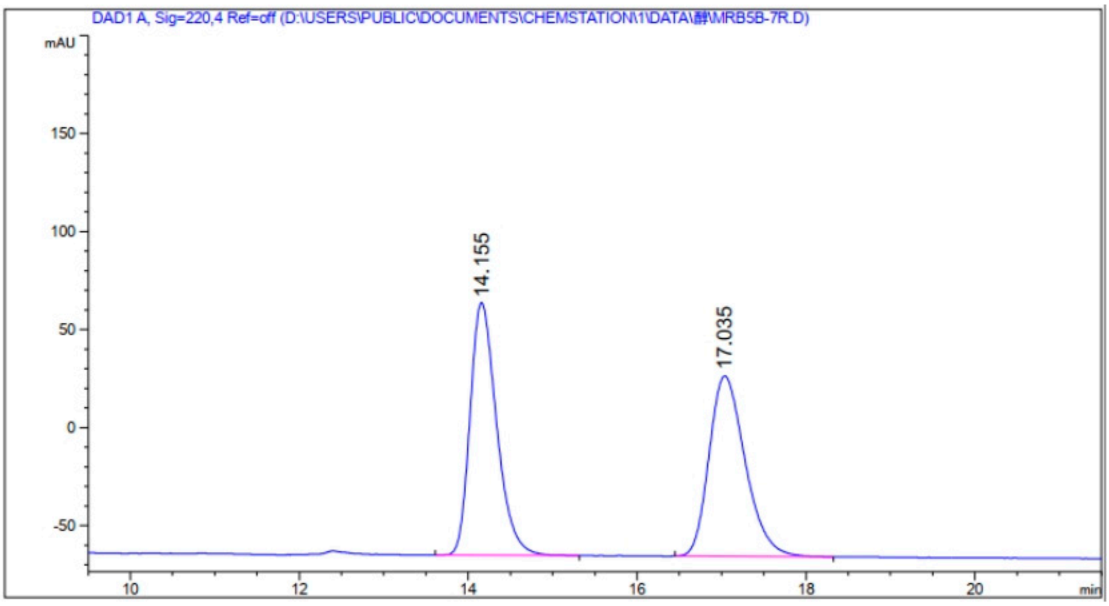

#	Time	Area	height	Width	Symmetry	Area%	Type
1	14.155	2812.3	128.8	0.3368	0.703	50.675	BB
2	17.035	2737.3	91.9	0.4574	0.719	49.325	BB

Figure S114: Racemic product of 3-(4-fluorophenyl)-1-(*p*-tolyl)propan-1-ol (3ja)

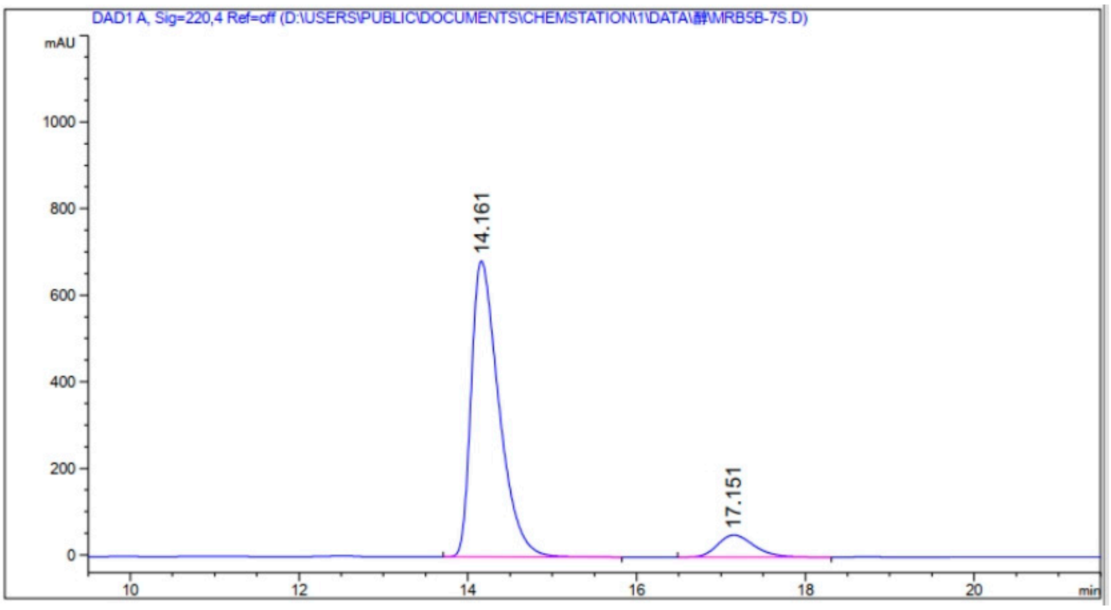

#	Time	Area	height	Width	Symmetry	Area%	Type
1	14.161	15580.1	683.8	0.3478	0.537	91.027	BB
2	17.151	1535.8	50.9	0.4639	0.753	8.973	BB

Figure S115: Chiral product of 3-(4-fluorophenyl)-1-(*p*-tolyl)propan-1-ol (3ja)

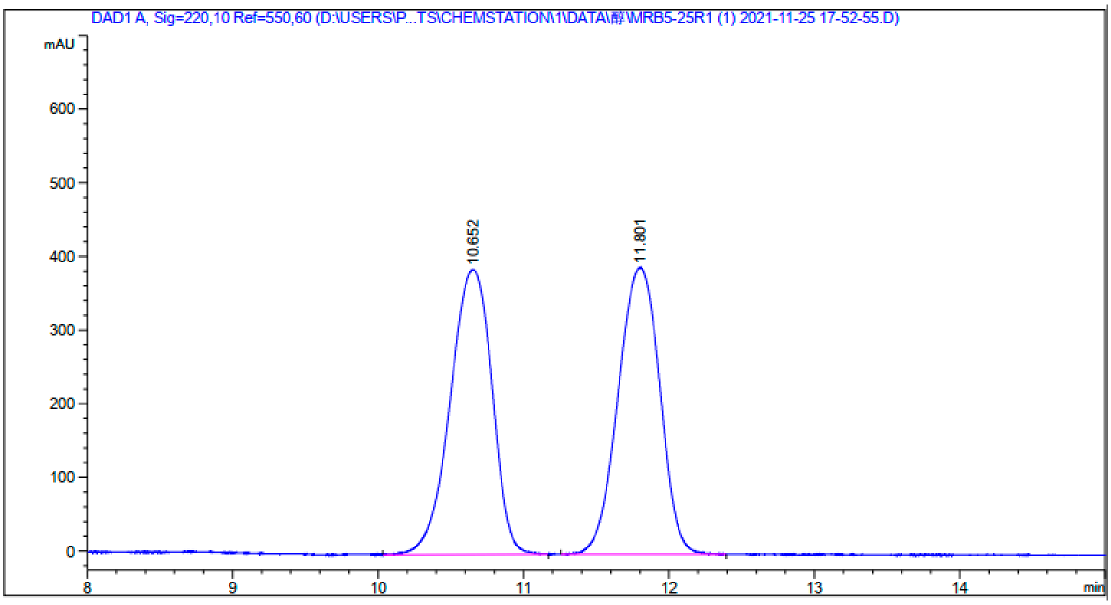

#	Time	Area	height	Width	Symmetry	Area%	Type
1	10.652	7724.1	386.8	0.238	1.142	49.902	VV R
2	11.801	7754.5	389.3	0.2354	1.06	50.098	BV R

Figure S116: Racemic product of cyclohexyl(p-tolyl)methanol (3da)

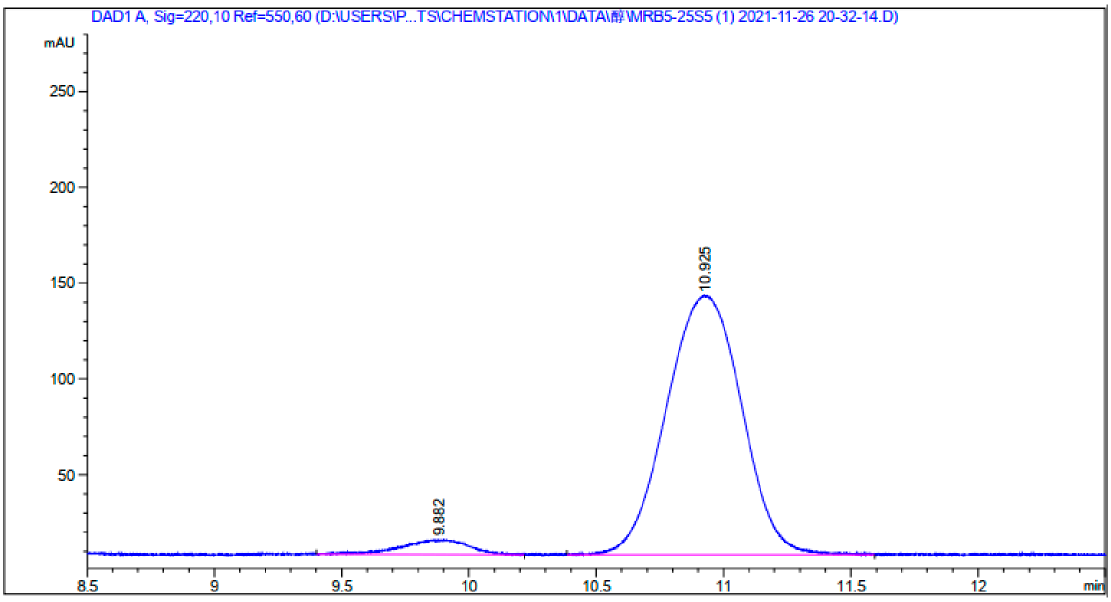

#	Time	Area	height	Width	Symmetry	Area%	Type
1	9.882	141.6	7.5	0.2226	1.23	4.857	VV R
2	10.925	2772.7	135.1	0.241	1.005	95.143	VV R

Figure S117: Chiral product of cyclohexyl(p-tolyl)methanol (3da)

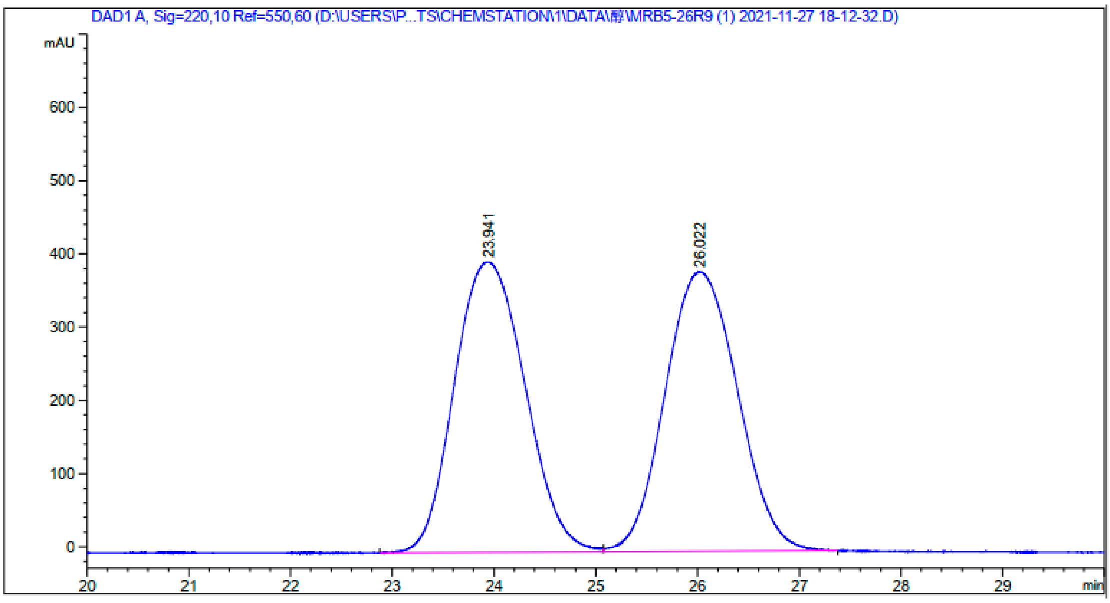

#	Time	Area	height	Width	Symmetry	Area%	Type
1	23.941	19120	396.2	0.5665	0.873	50.301	BV R
2	26.022	18891.2	381.4	0.5789	0.89	49.699	VB

Figure S118: Racemic product of (4-chlorophenyl)(cyclohexyl)methanol (3de)

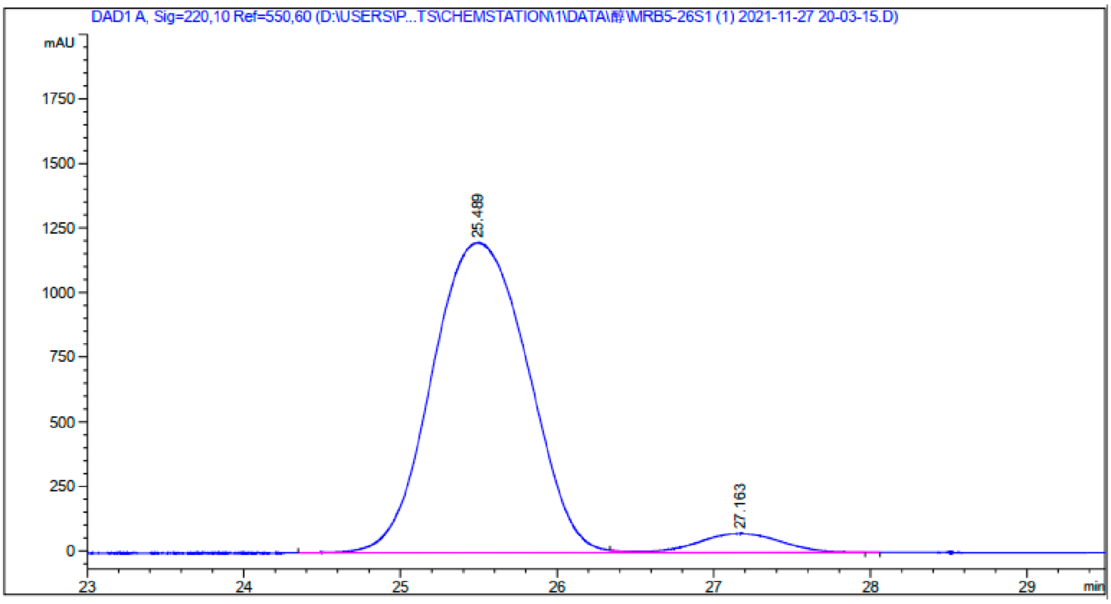

#	Time	Area	height	Width	Symmetry	Area%	Type
1	25.489	50948.5	1200	0.4978	0.871	94.971	VV R
2	27.163	2698	73.4	0.4311	0.957	5.029	VV E

Figure S119: Chiral product of (4-chlorophenyl)(cyclohexyl)methanol (3de)

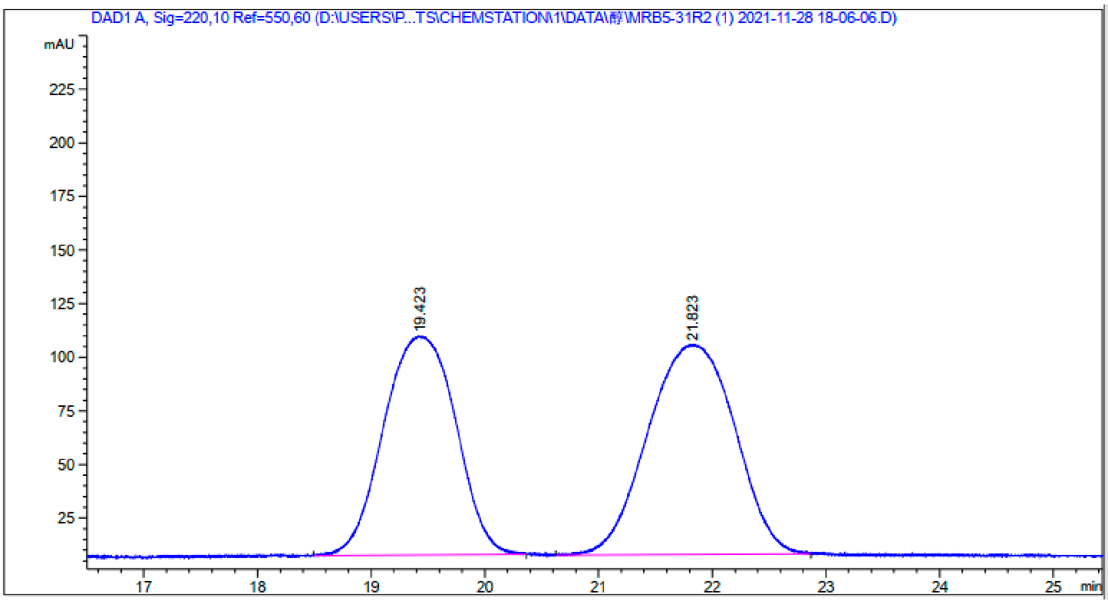

#	Time	Area	height	Width	Symmetry	Area%	Type
1	19.423	4507.1	101.9	0.5182	0.956	46.444	BV R
2	21.823	5197.2	97.6	0.6226	1.017	53.556	VV R

Figure S120: Racemic product of cyclohexyl(4-(trifluoromethyl)phenyl)methanol (3dh)

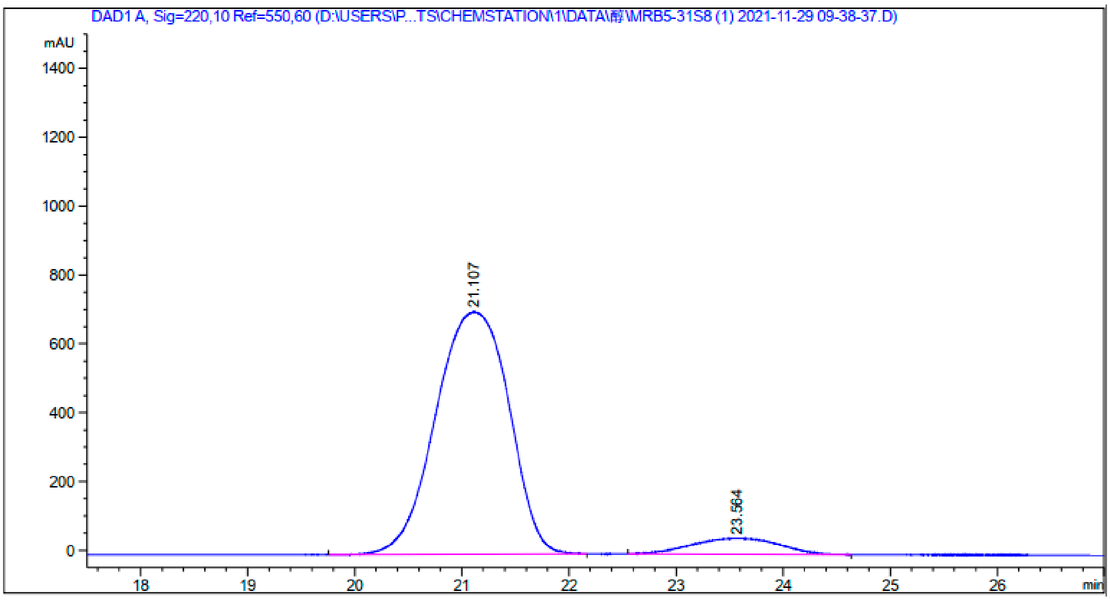

#	Time	Area	height	Width	Symmetry	Area%	Type
1	21.107	33505.3	703.4	0.5572	1.038	92.829	VB R
2	23.564	2588.1	45.6	0.6662	1.007	7.171	VV R

Figure S121: Chiral product of cyclohexyl(4-(trifluoromethyl)phenyl)methanol (3dh)

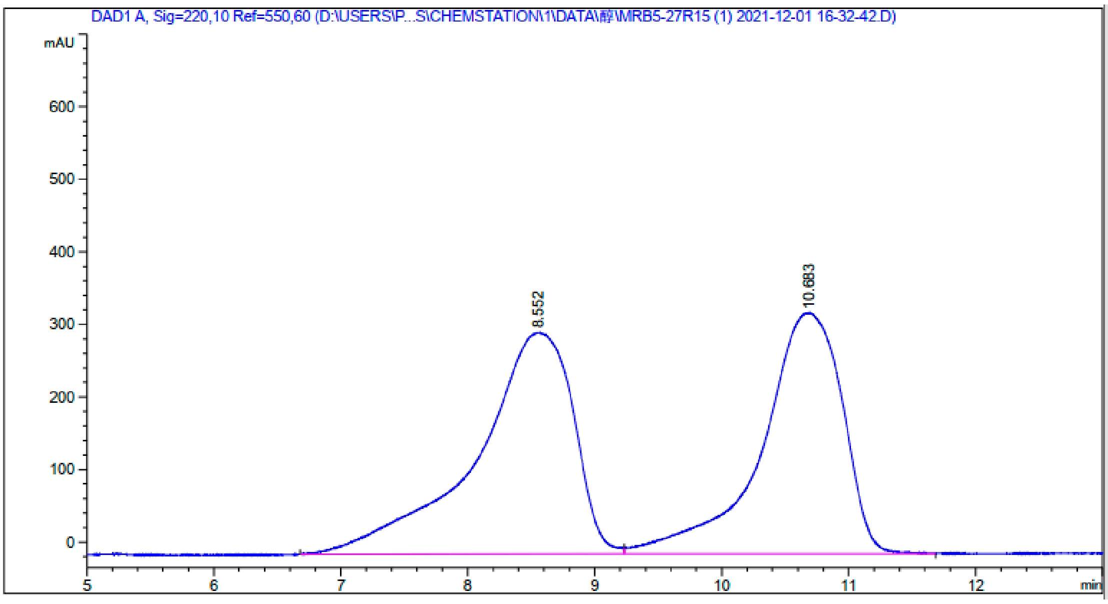

#	Time	Area	height	Width	Symmetry	Area%	Type
1	8.552	16302.1	305.3	0.6239	1.721	52.510	BV R
2	10.683	14743.6	331.7	0.5204	1.373	47.490	VV R

Figure S122: Racemic product of 1-(p-tolyl)heptan-1-ol (3ea)

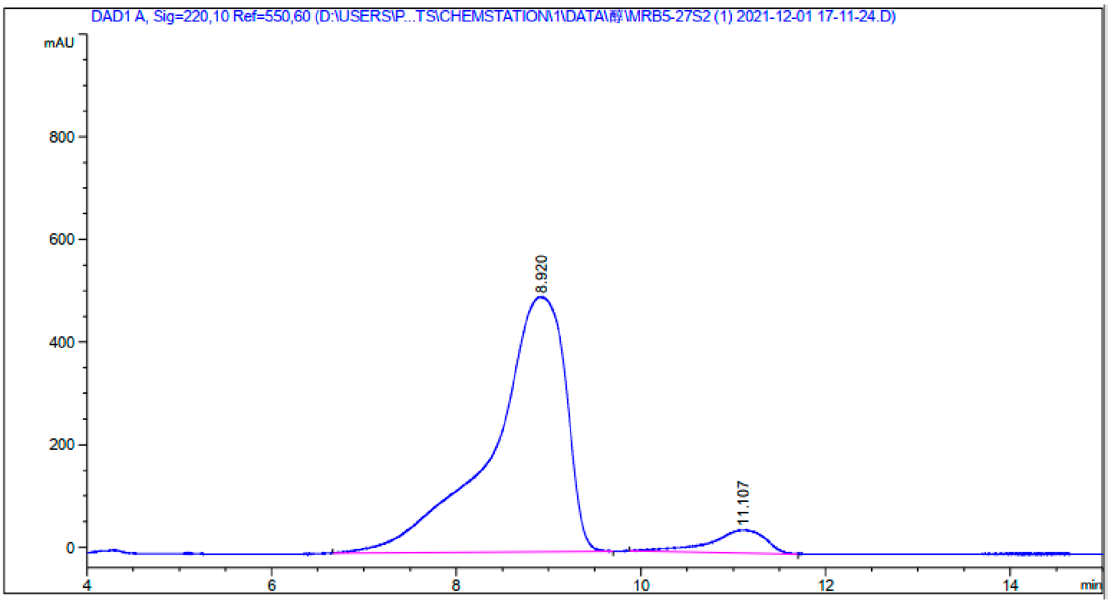

#	Time	Area	height	Width	Symmetry	Area%	Type
1	8.92	27379.8	497	0.6458	1.943	93.626	BV R
2	11.107	1863.9	44.5	0.4899	1.468	6.374	VB R

Figure S123: Chiral product of 1-(p-tolyl)heptan-1-ol (3ea)

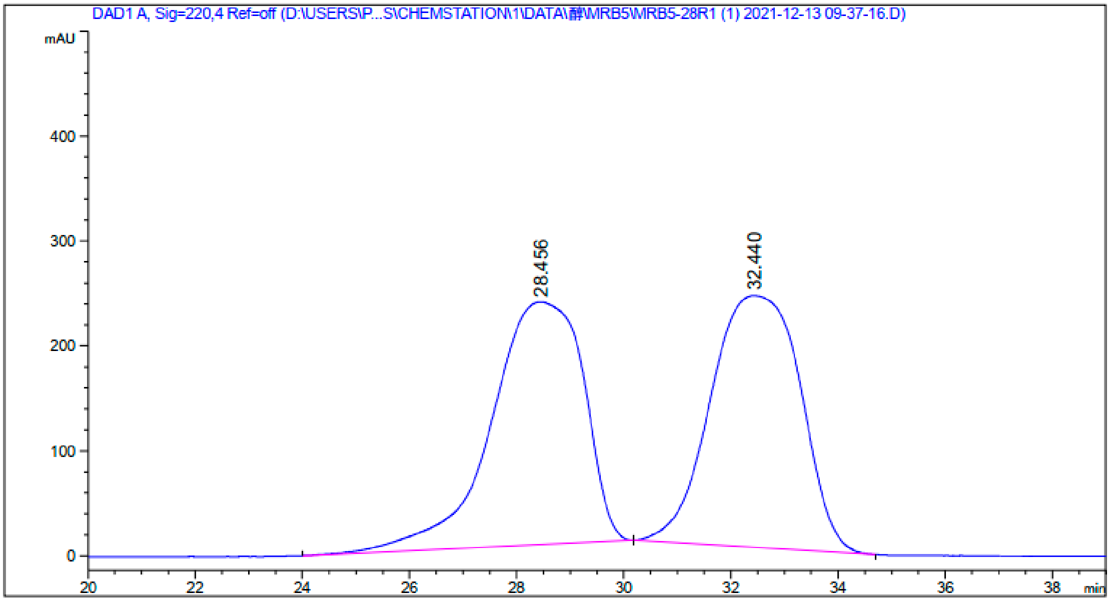

#	Time	Area	height	Width	Symmetry	Area%	Type
1	28.456	27825.9	231.1	1.6726	1.184	49.837	BB
2	32.44	28008	239.9	1.3879	0.932	50.163	BB

Figure S124: Racemic product of 1-(4-chlorophenyl)heptan-1-ol (3ee)

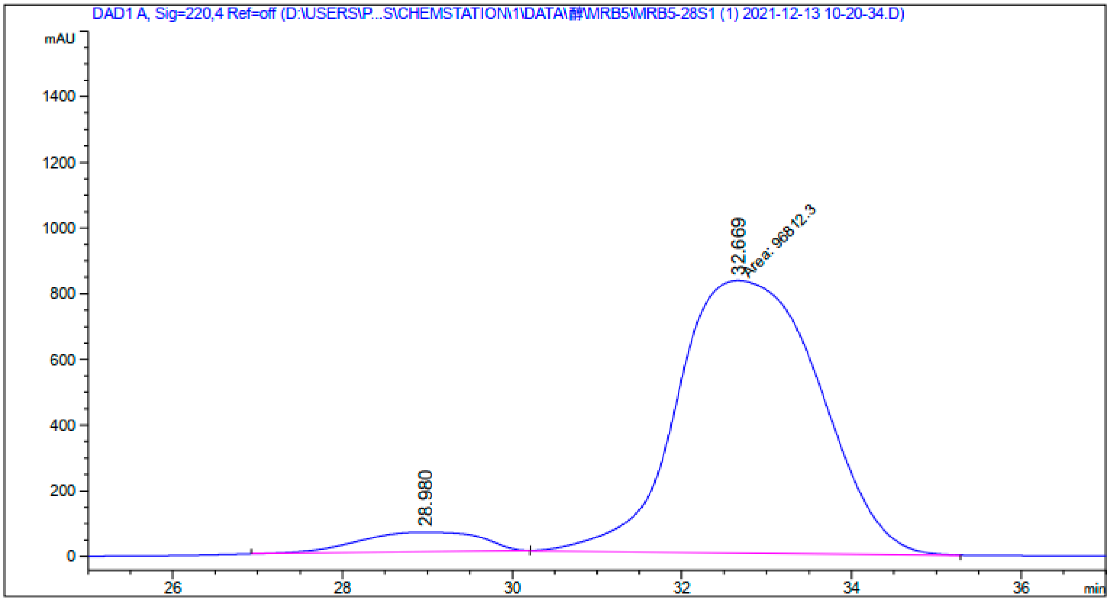

#	Time	Area	height	Width	Symmetry	Area%	Type
1	28.98	5922.9	59.5	1.1843	1.228	5.765	BB
2	32.669	96812.3	829.7	1.9447	0.741	94.235	MM

Figure S125: Chiral product of 1-(4-chlorophenyl)heptan-1-ol (3ee)

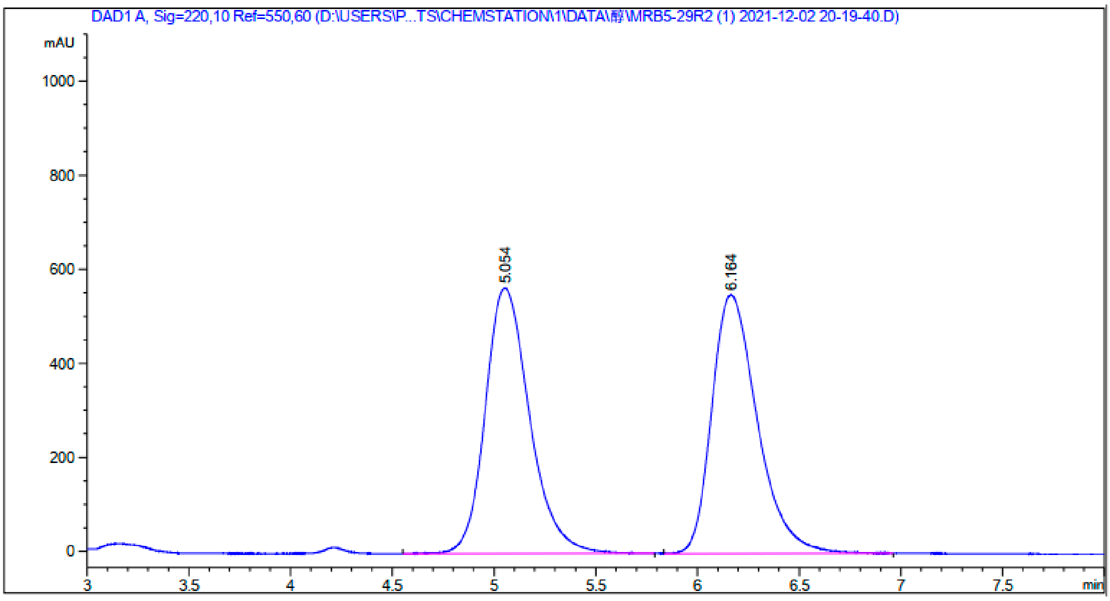

#	Time	Area	height	Width	Symmetry	Area%	Type
1	5.054	8569.9	564.4	0.2212	0.767	50.120	VV R
2	6.164	8528.8	549.1	0.2227	0.686	49.880	BV R

Figure S126: Racemic product of 3-methyl-1-(p-tolyl)butan-1-ol (3fa)

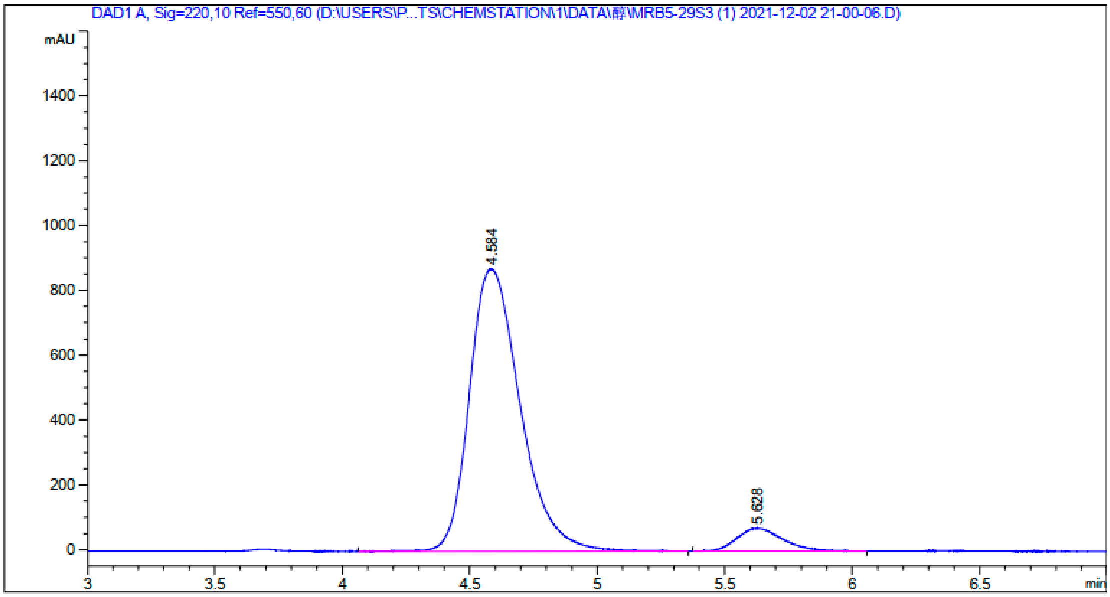

#	Time	Area	height	Width	Symmetry	Area%	Type
1	4.584	12098.7	871.2	0.172	0.717	93.121	BV R
2	5.628	893.8	70.3	0.1528	0.773	6.879	BV R

Figure S127: Chiral product of 3-methyl-1-(p-tolyl)butan-1-ol (3fa)

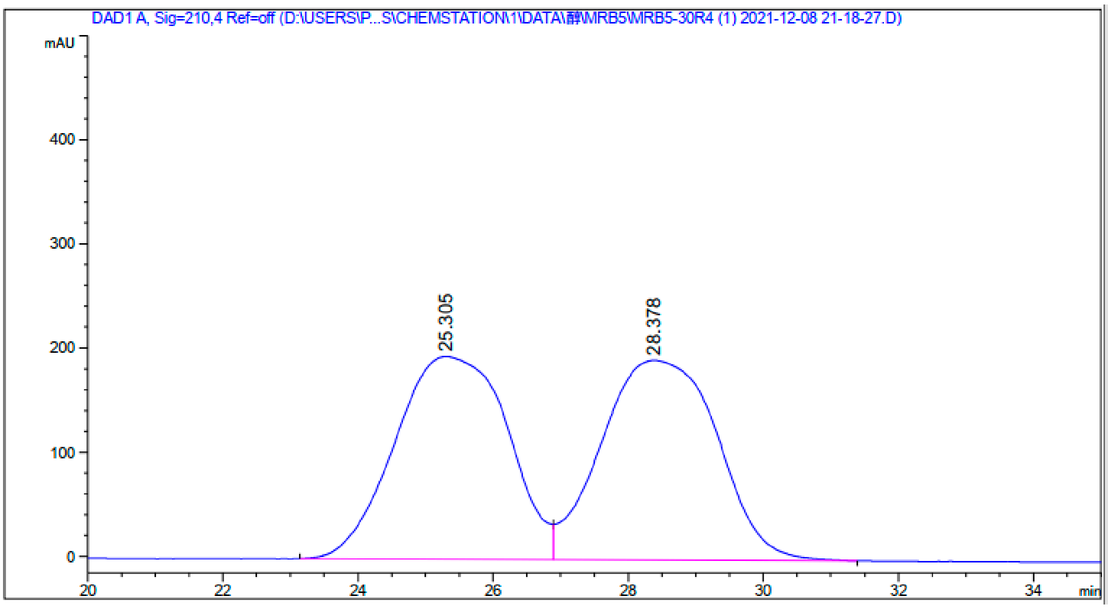

#	Time	Area	height	Width	Symmetry	Area%	Type
1	25.305	22603.1	194.6	1.445	0.83	49.639	BV
2	28.378	22932.3	191.7	1.6754	0.804	50.361	VB

Figure S128: Racemic product of 1-(4-chlorophenyl)-3-methylbutan-1-ol (3fe)

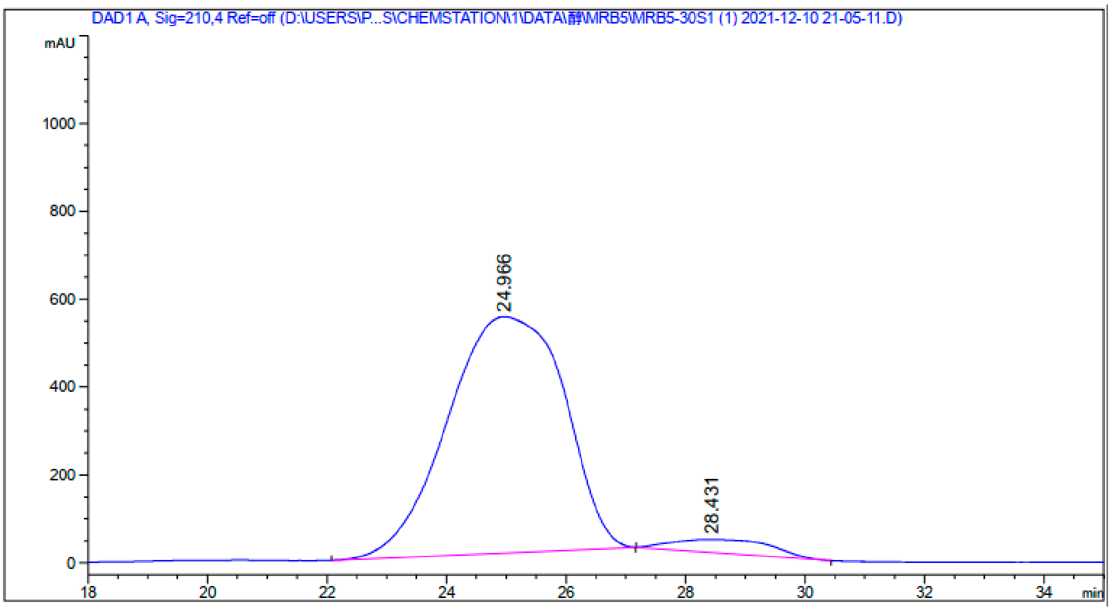

#	Time	Area	height	Width	Symmetry	Area%	Type
1	24.966	72832.2	537.4	1.8672	0.924	95.363	BB
2	28.431	3541.8	29.8	1.4074	0.543	4.637	BB

Figure S129: Chiral product of 1-(4-chlorophenyl)-3-methylbutan-1-ol (3fe)

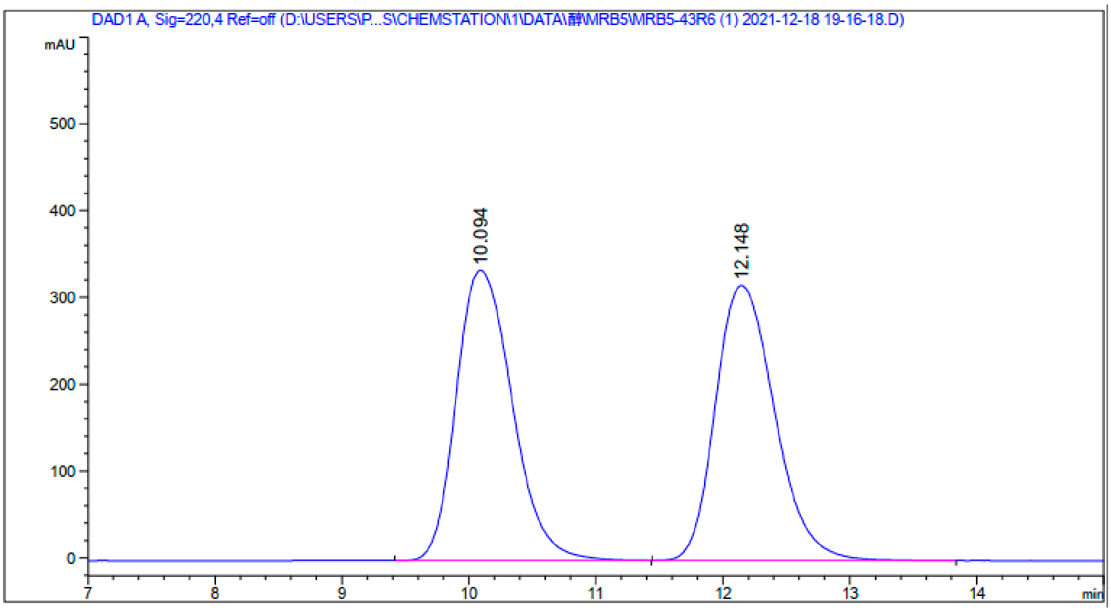

#	Time	Area	height	Width	Symmetry	Area%	Type
1	10.094	10263.9	334.3	0.4862	0.729	49.980	BB
2	12.148	10272.2	316.5	0.5106	0.741	50.020	BB

Figure S130: Racemic product of 1-(p-tolyl)propan-1-ol (3ga)

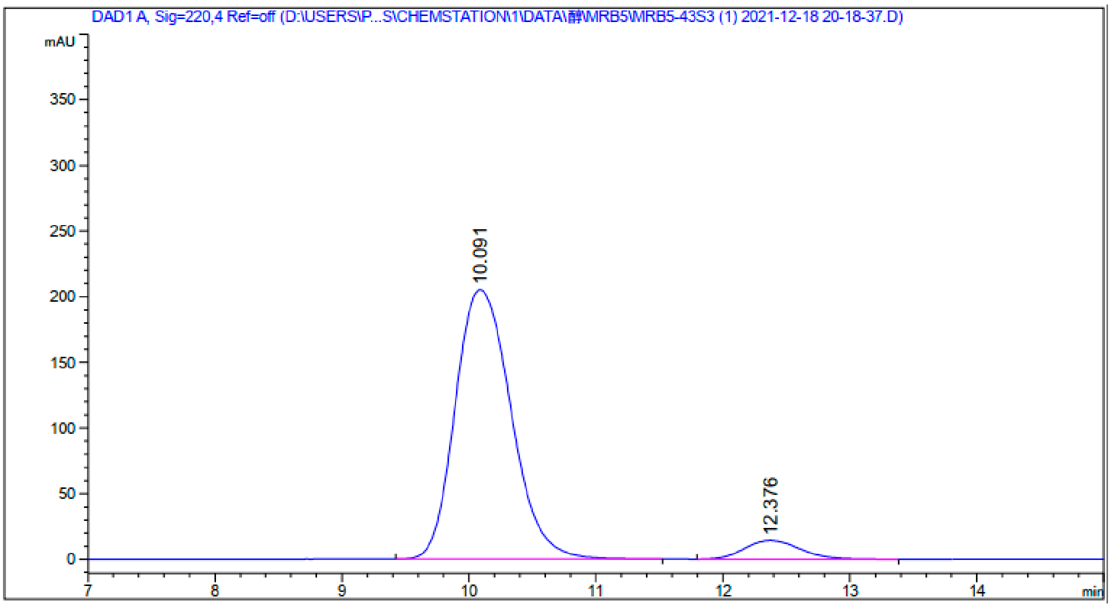

#	Time	Area	height	Width	Symmetry	Area%	Type
1	10.091	6263.2	205	0.4884	0.769	93.454	BB
2	12.376	438.7	14.3	0.425	0.821	6.546	BB

Figure S131: Chiral product of 1-(p-tolyl)propan-1-ol (3ga)

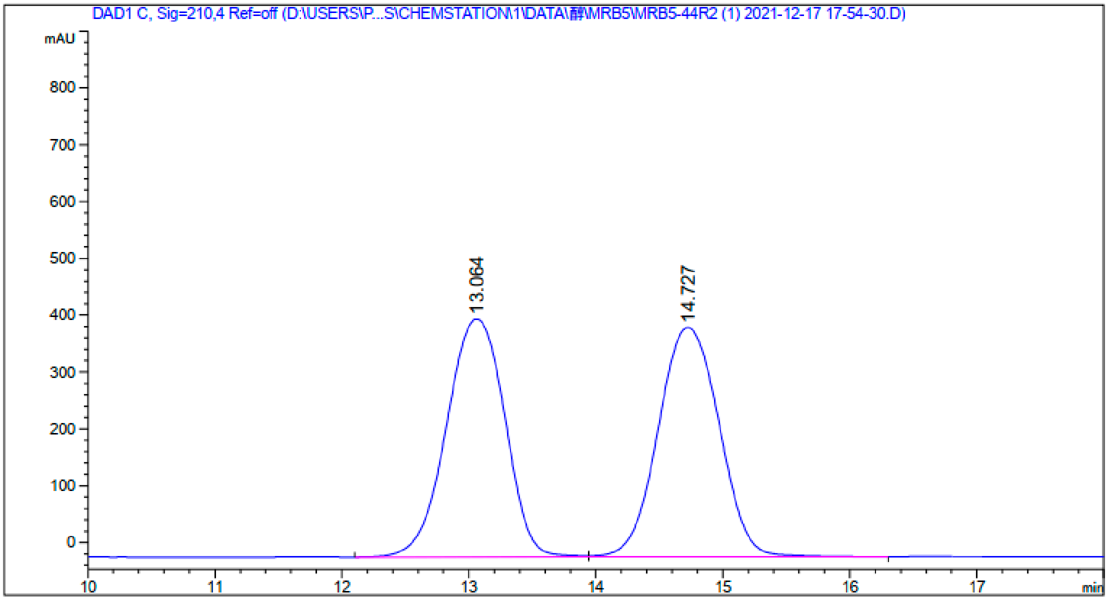

#	Time	Area	height	Width	Symmetry	Area%	Type
1	13.064	13377.9	418.7	0.5148	1.047	49.838	BV
2	14.727	13464.9	403.7	0.5351	0.924	50.162	VB

Figure S132: Racemic product of 1-(4-chlorophenyl)propan-1-ol (3ge)

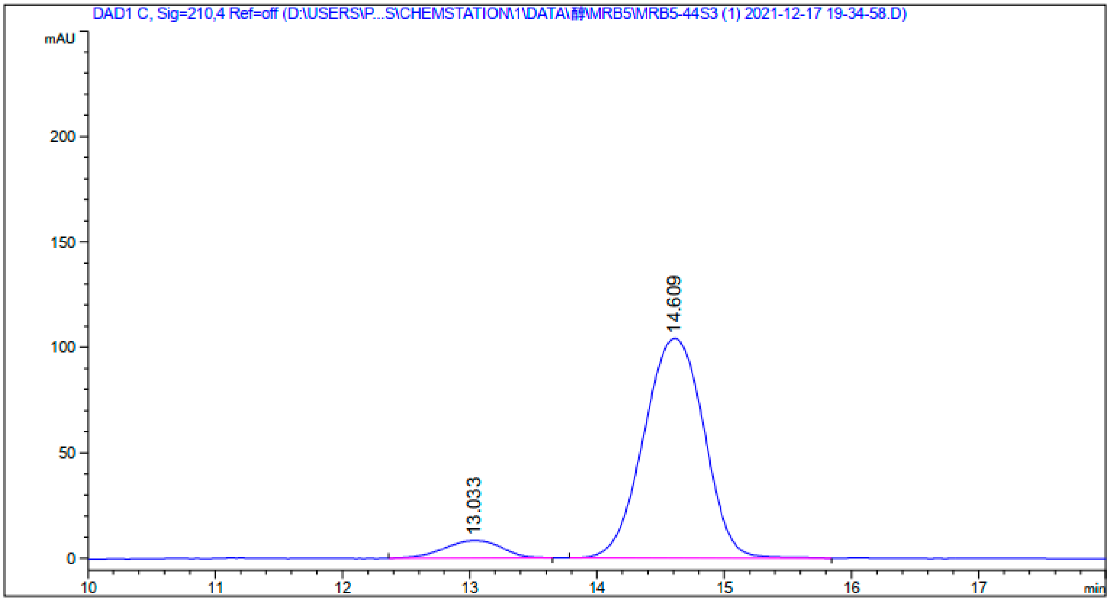

#	Time	Area	height	Width	Symmetry	Area%	Type
1	13.033	259.6	8.2	0.5063	1.106	7.044	BB
2	14.609	3425.9	104.2	0.5277	1.032	92.956	BB

Figure S133: Chiral product of 1-(4-chlorophenyl)propan-1-ol (3ge)

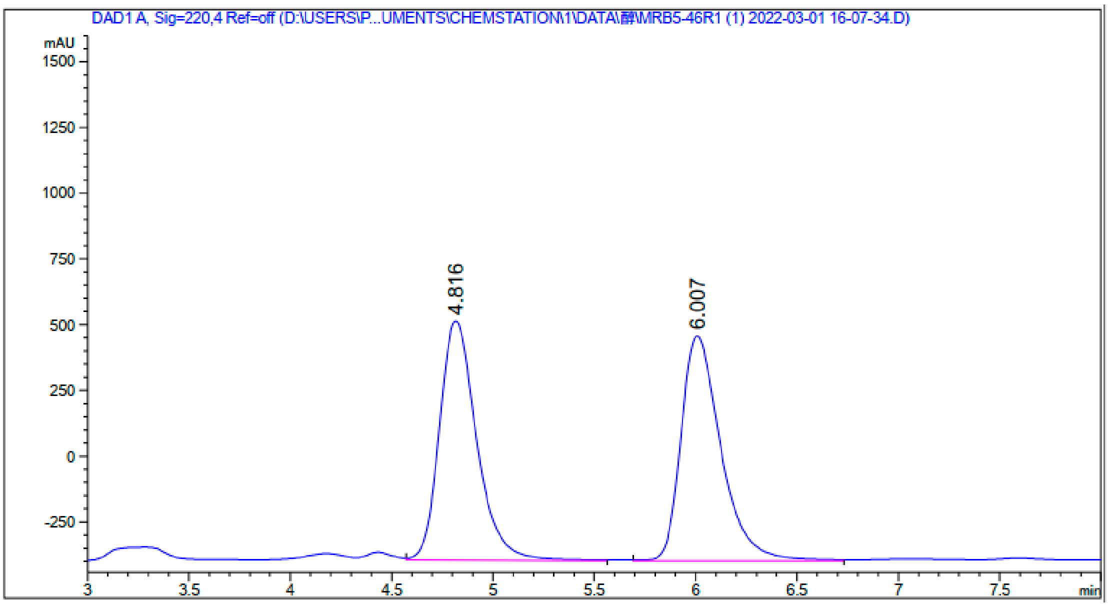

#	Time	Area	height	Width	Symmetry	Area%	Type
1	4.816	11603.4	910.1	0.2125	0.746	49.786	MM R
2	6.007	11703	854.7	0.2282	0.654	50.214	MM R

Figure S134: Racemic product of 2,2-dimethyl-1-(p-tolyl)propan-1-ol (3ha)

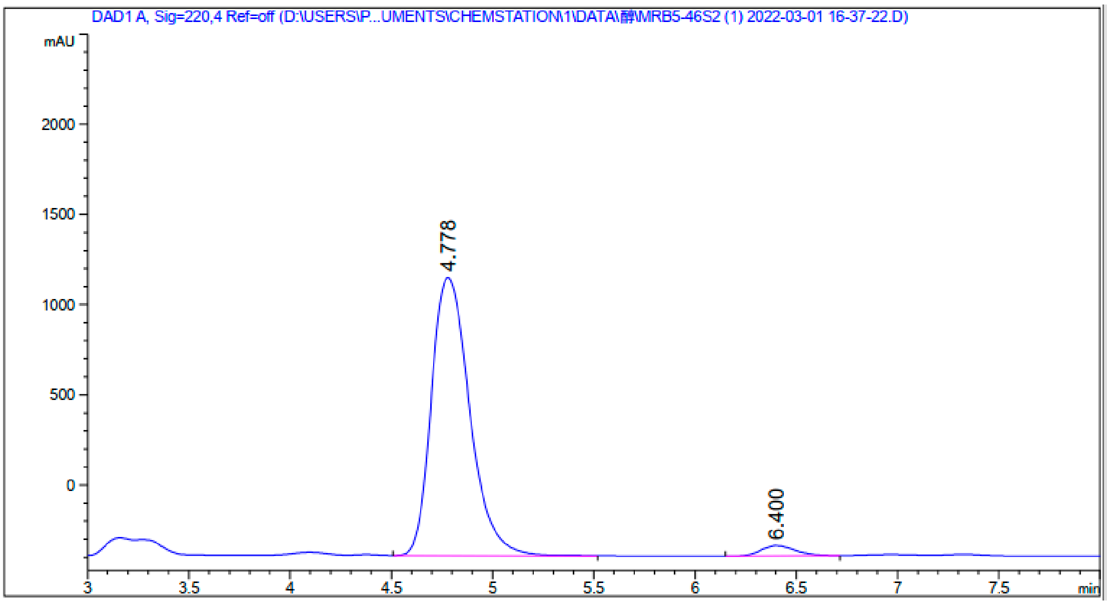

#	Time	Area	height	Width	Symmetry	Area%	Type
1	4.778	20376.5	1541.6	0.2203	0.733	96.769	MM R
2	6.4	680.4	57.3	0.1846	0.791	3.231	BB

Figure S135: Chiral product of 2,2-dimethyl-1-(p-tolyl)propan-1-ol (3ha)

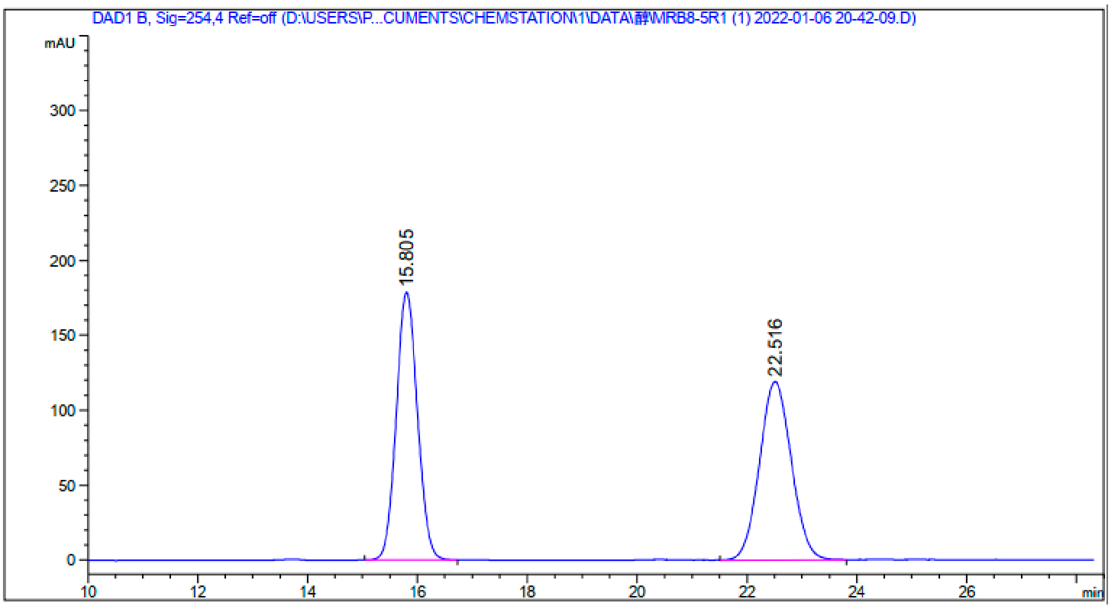

#	Time	Area	height	Width	Symmetry	Area%	Type
1	15.805	4784.5	178.6	0.4166	0.909	49.952	BB
2	22.516	4793.7	119.1	0.6296	0.931	50.048	BB

Figure S136: Racemic product of (*E*)-3-phenyl-1-(p-tolyl)prop-2-en-1-ol (3ia)

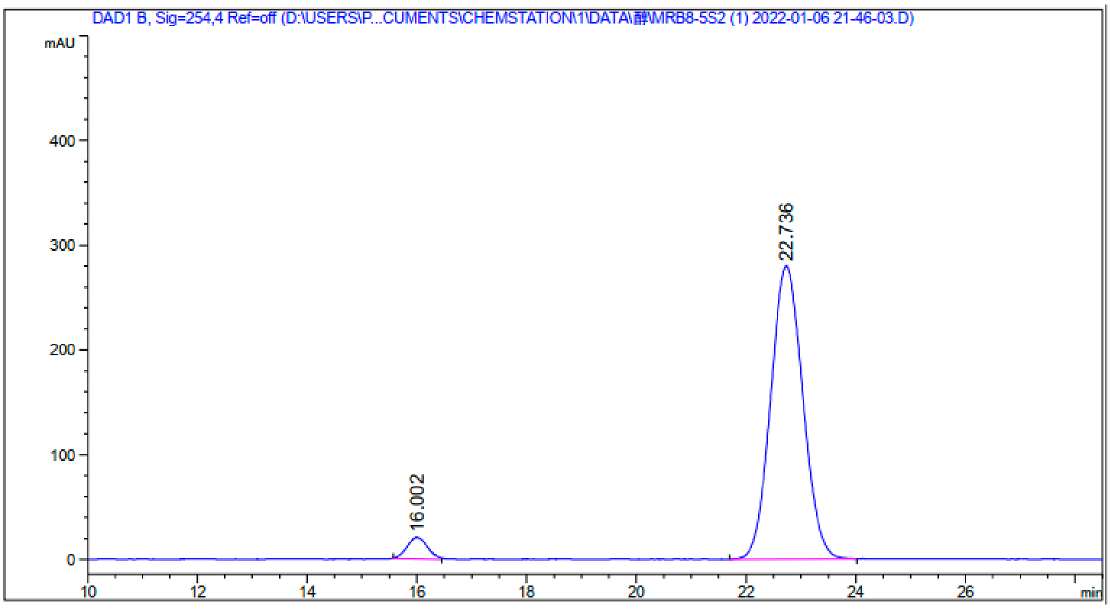

#	Time	Area	height	Width	Symmetry	Area%	Type
1	16.002	537	20.6	0.4336	0.898	4.503	MM R
2	22.736	11388.2	279.5	0.6354	0.906	95.497	BB

Figure S137: Chiral product of (*E*)-3-phenyl-1-(p-tolyl)prop-2-en-1-ol (3ia)

I. HRMS-ESI Spectra

Elemental Composition Report **Page 1**

Single Mass Analysis
Tolerance = 10.0 PPM / DBE: min = -1.5, max = 50.0
Element prediction: Off
Number of isotope peaks used for i-FIT = 3

Monoisotopic Mass, Even Electron Ions
123 formula(e) evaluated with 1 results within limits (up to 50 closest results for each mass)
Elements Used:
C: 18-18 H: 0-100 N: 0-8 O: 0-20

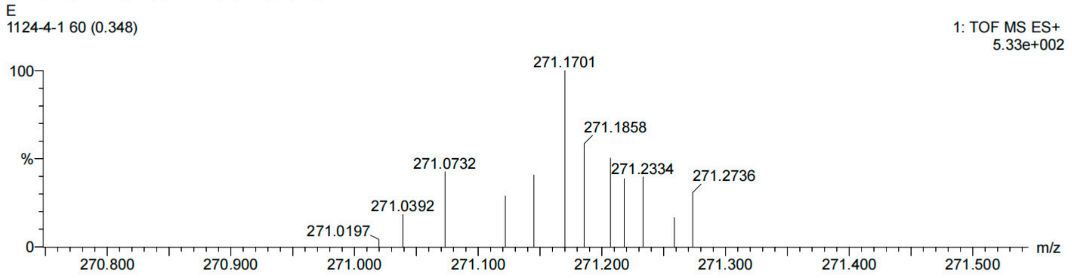

	Mass	Calc. Mass	mDa	PPM	DBE	i-FIT	Norm	Conf(%)	Formula
Minimum:					-1.5				
Maximum:			5.0	10.0	50.0				
	271.1701	271.1698	0.3	1.1	7.5	85.0	n/a	n/a	C18 H23 O2

Figure S138: HRMS of 1-(4-isopropoxyphenyl)-3-phenylpropan-1-ol (3ad)

Elemental Composition Report **Page 1**

Single Mass Analysis
Tolerance = 10.0 PPM / DBE: min = -1.5, max = 50.0
Element prediction: Off
Number of isotope peaks used for i-FIT = 3

Monoisotopic Mass, Even Electron Ions
114 formula(e) evaluated with 1 results within limits (up to 50 closest results for each mass)
Elements Used:
C: 18-18 H: 0-100 N: 0-8 O: 0-20 Na: 1-1

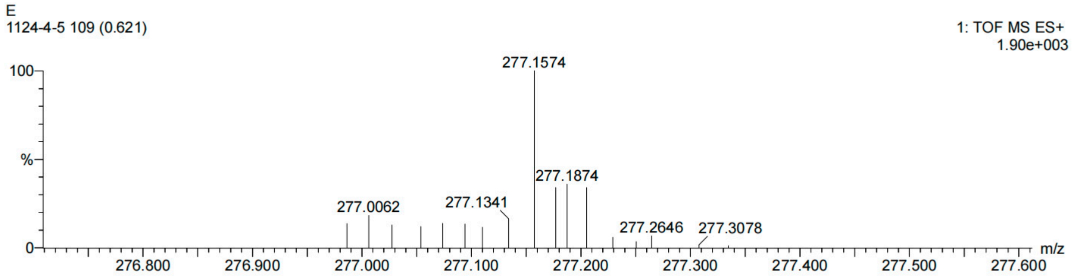

	Mass	Calc. Mass	mDa	PPM	DBE	i-FIT	Norm	Conf(%)	Formula
Minimum:					-1.5				
Maximum:			5.0	10.0	50.0				
	277.1574	277.1568	0.6	2.2	7.5	104.2	n/a	n/a	C18 H22 O Na

Figure S139: HRMS of 1-(3-isopropylphenyl)-3-phenylpropan-1-ol (3am)

Elemental Composition Report

Page 1

Single Mass Analysis
Tolerance = 10.0 PPM / DBE: min = -1.5, max = 50.0
Element prediction: Off
Number of isotope peaks used for i-FIT = 3

Monoisotopic Mass, Even Electron Ions
426 formula(e) evaluated with 1 results within limits (up to 50 closest results for each mass)
Elements Used:
C: 15-15 H: 0-100 N: 0-8 O: 0-20 Cl: 1-2 F: 1-3

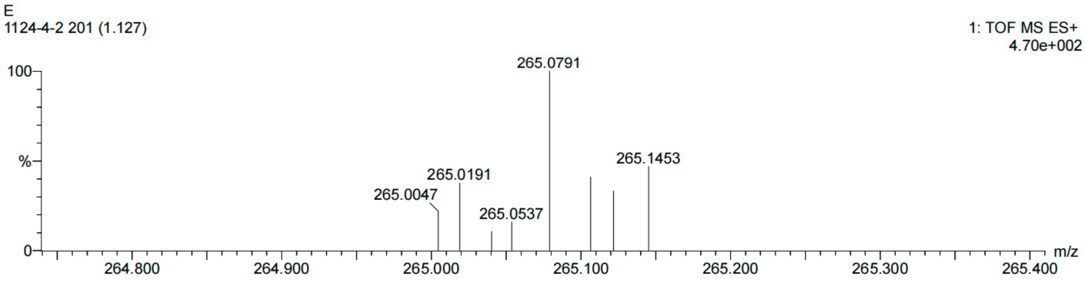

Minimum:				-1.5				
Maximum:		5.0	10.0	50.0				
Mass	Calc. Mass	mDa	PPM	DBE	i-FIT	Norm	Conf(%)	Formula
265.0791	265.0795	-0.4	-1.5	7.5	53.4	n/a	n/a	C15 H15 O Cl F

Figure S140: HRMS of 1-(3-chloro-4-fluorophenyl)-3-phenylpropan-1-ol (3aq)

Elemental Composition Report

Page 1

Single Mass Analysis
Tolerance = 10.0 PPM / DBE: min = -1.5, max = 50.0
Element prediction: Off
Number of isotope peaks used for i-FIT = 3

Monoisotopic Mass, Even Electron Ions
102 formula(e) evaluated with 1 results within limits (up to 50 closest results for each mass)
Elements Used:
C: 15-15 H: 0-100 N: 0-8 O: 0-20

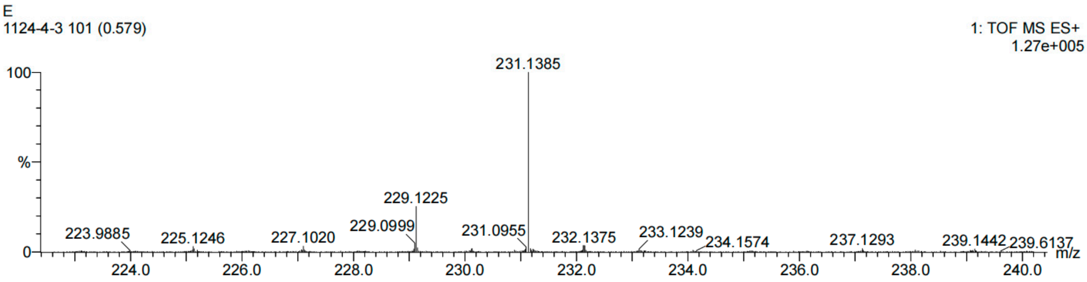

Minimum:				-1.5				
Maximum:		5.0	10.0	50.0				
Mass	Calc. Mass	mDa	PPM	DBE	i-FIT	Norm	Conf(%)	Formula
231.1385	231.1385	0.0	0.0	6.5	1091.6	n/a	n/a	C15 H19 O2

Figure S141: HRMS of 3-(5-methylfuran-2-yl)-1-(p-tolyl)propan-1-ol (3ca)

Elemental Composition Report

Page 1

Single Mass Analysis

Tolerance = 10.0 PPM / DBE: min = -1.5, max = 50.0
Element prediction: Off
Number of isotope peaks used for i-FIT = 3

Monoisotopic Mass, Even Electron Ions
169 formula(e) evaluated with 1 results within limits (up to 50 closest results for each mass)
Elements Used:
C: 14-14 H: 0-100 N: 0-8 O: 0-20 Cl: 1-2

E
1124-4-4 96 (0.553)

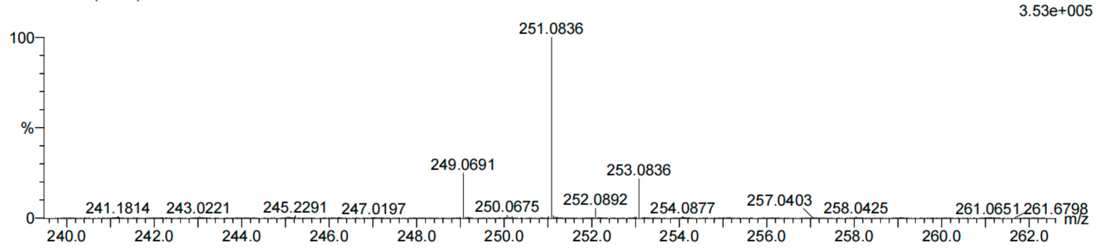

Minimum:				-1.5					
Maximum:		5.0	10.0	50.0					
Mass	Calc. Mass	mDa	PPM	DBE	i-FIT	Norm	Conf(%)	Formula	
251.0836	251.0839	-0.3	-1.2	6.5	1088.6	n/a	n/a	C14 H16 O2 Cl	

Figure S142: HRMS of 1-(4-chlorophenyl)-3-(5-methylfuran-2-yl)propan-1-ol (3ce)

Elemental Composition Report

Page 1

Single Mass Analysis

Tolerance = 5.0 PPM / DBE: min = -1.5, max = 50.0
Element prediction: Off
Number of isotope peaks used for i-FIT = 3

Monoisotopic Mass, Even Electron Ions
517 formula(e) evaluated with 1 results within limits (up to 50 closest results for each mass)
Elements Used:
C: 16-16 H: 0-39 N: 0-50 O: 0-50 F: 1-6

16
0611-1--01 72 (0.443)

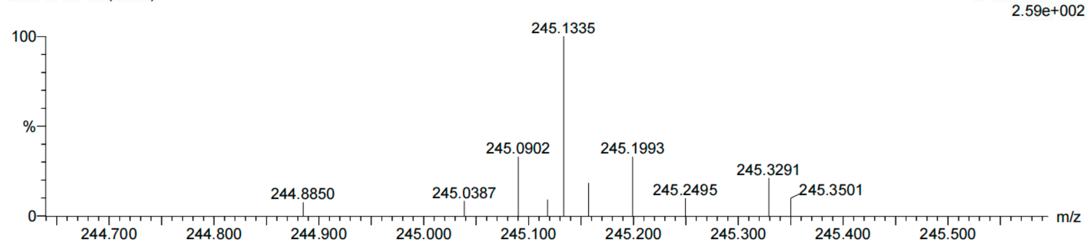

Minimum:				-1.5				
Maximum:		5.0	5.0	50.0				
Mass	Calc. Mass	mDa	PPM	DBE	i-FIT	Norm	Conf(%)	Formula
245.1335	245.1342	-0.7	-2.9	7.5	58.1	n/a	n/a	C16 H18 O F

Figure S143: HRMS of 3-(4-fluorophenyl)-1-(p-tolyl)propan-1-ol (3ja)

Single Mass Analysis

Tolerance = 50.0 PPM / DBE: min = -1.5, max = 50.0
Element prediction: Off
Number of isotope peaks used for i-FIT = 3

Monoisotopic Mass, Even Electron Ions
15 formula(e) evaluated with 1 results within limits (up to 50 closest results for each mass)
Elements Used:
C: 38-38 H: 0-50 N: 0-8 O: 6-6 Cl: 2-2 Ru: 0-1 P: 2-2

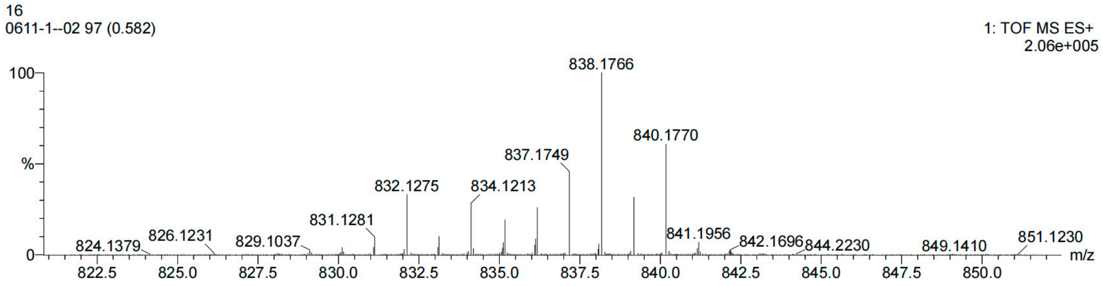

	Calc. Mass	mDa	PPM	DBE	i-FIT	Norm	Conf(%)	Formula
Minimum:				-1.5				
Maximum:		5.0	50.0	50.0				
**Mass**	**Calc. Mass**	**mDa**	**PPM**	**DBE**	**i-FIT**	**Norm**	**Conf(%)**	**Formula**
833.1254	833.1268	-1.4	-1.7	15.5	630.5	n/a	n/a	C38 H47 O6 Cl2 Ru P2

Figure S144: HRMS of Ru-L1
